# Synthesis and Biological Evaluation of 1-(Diarylmethyl)-1*H*-1,2,4-triazoles and 1-(Diarylmethyl)-1*H*-imidazoles as a Novel Class of Anti-Mitotic Agent for Activity in Breast Cancer

**DOI:** 10.3390/ph14020169

**Published:** 2021-02-22

**Authors:** Gloria Ana, Patrick M. Kelly, Azizah M. Malebari, Sara Noorani, Seema M. Nathwani, Brendan Twamley, Darren Fayne, Niamh M. O’Boyle, Daniela M. Zisterer, Elisangela Flavia Pimentel, Denise Coutinho Endringer, Mary J. Meegan

**Affiliations:** 1 School of Pharmacy and Pharmaceutical Sciences, Trinity College Dublin, Trinity Biomedical Sciences Institute, 152-160 Pearse Street, Dublin 2, DO2R590 Dublin, Ireland; anag@tcd.ie (G.A.); kellyp9@tcd.ie (P.M.K.); noorani87@gmail.com (S.N.); NIOBOYLE@tcd.ie (N.M.O.); 2 Department of Pharmaceutical Chemistry, College of Pharmacy, King Abdulaziz University, Jeddah 21589, Saudi Arabia; amelibary@kau.edu.sa; 3 School of Biochemistry and Immunology, Trinity College Dublin, Trinity Biomedical Sciences Institute, 152-160 Pearse Street, Dublin 2, DO2R590 Dublin, Ireland; seema.nathwani@outlook.com (S.M.N.); FAYNED@tcd.ie (D.F.); dzistrer@tcd.ie (D.M.Z.); 4 School of Chemistry, Trinity College Dublin, Dublin 2, DO2R590 Dublin, Ireland; TWAMLEYB@tcd.ie; 5 Department of Pharmaceutical Sciences, University Vila Velha, Av. Comissário José Dantas de Melo, n°21, Boa Vista Vila Velha—Espírito Santo, Vila Velha 29102-920, Brazil; eflapim@hotmail.com (E.F.P.); endringe@gmail.com (D.C.E.)

**Keywords:** phenstatin, letrozole, tubulin polymerisation inhibitor, aromatase inhibitor, breast cancer, hybrid molecule, dual-targeting molecule, apoptosis, designed multiple ligand

## Abstract

We report the synthesis and biochemical evaluation of compounds that are designed as hybrids of the microtubule targeting benzophenone phenstatin and the aromatase inhibitor letrozole. A preliminary screening in estrogen receptor (ER)-positive MCF-7 breast cancer cells identified 5-((2*H*-1,2,3-triazol-1-yl)(3,4,5-trimethoxyphenyl)methyl)-2-methoxyphenol **24** as a potent antiproliferative compound with an IC_50_ value of 52 nM in MCF-7 breast cancer cells (ER+/PR+) and 74 nM in triple-negative MDA-MB-231 breast cancer cells. The compounds demonstrated significant G_2_/M phase cell cycle arrest and induction of apoptosis in the MCF-7 cell line, inhibited tubulin polymerisation, and were selective for cancer cells when evaluated in non-tumorigenic MCF-10A breast cells. The immunofluorescence staining of MCF-7 cells confirmed that the compounds targeted tubulin and induced multinucleation, which is a recognised sign of mitotic catastrophe. Computational docking studies of compounds **19e**, **21l**, and **24** in the colchicine binding site of tubulin indicated potential binding conformations for the compounds. Compounds **19e** and **21l** were also shown to selectively inhibit aromatase. These compounds are promising candidates for development as antiproliferative, aromatase inhibitory, and microtubule-disrupting agents for breast cancer.

## 1. Introduction

Designing single agents that act against multiple biological targets is of increasing interest and prominence in medicinal chemistry [1,2,3,4]. Dual-targeting drugs are designed with the potential to be more potent and efficient and overcome many of the disadvantages of single drugs such as low solubility, side effects [5], and multidrug resistance (MDR). While the molecular mechanisms of resistance to chemotherapeutics have been identified, MDR is known to be a key factor in the failure of breast cancer chemotherapy [6]. Traditionally, drugs have been designed to target a single biological target (protein), aiming for high selectivity and thus avoiding unwanted effects due to off-target events. The interaction of a drug with multiple target proteins has been regarded as potentially associated with adverse side effects. However, for complex diseases such as cancer, it is now recognised that a single-target drug may not achieve the optimum therapeutic effect. Molecules that are effective at more than one target protein may overcome incomplete efficacy and demonstrate an increased safety profile compared to single-targeted ones [2]. Dual-targeting strategies may offer a more favourable outcome of cancer treatment.

A possible strategy to improve the outcome for postmenopausal breast cancer patients is to design compounds with dual aromatase and tubulin targeting activities, which may offer the potential benefits of improved efficacy and fewer side effects [7,8]. The objective of our research is to investigate a new series of 1-(diarylmethyl)-1*H*-1,2,4-triazoles and 1-(diarylmethyl)-1*H*-imidazoles as a novel class of antimitotic compounds with an interesting biochemical profile particularly as tubulin-targeting agents and aromatase inhibitors for the treatment of breast cancer. 

Breast cancer is the most commonly diagnosed cancer in women; it is estimated that approximately one in eight women will develop breast cancer during their lifetime, and it is the most frequent cause of death for women in the age group 35–55 [9]. There were over two million new cases in 2018 [10], and the number of cases is predicted to rise due to an ageing population [11,12]. Mortality has decreased due to improved screening and early detection together with the use of adjuvant therapy [13]. Approximately 70–80% of breast cancers are hormone-dependent; their growth is stimulated in response to the hormone estrogen, with the majority of these estrogen receptor positive (ER+) cancers also expressing the progesterone receptor (ER+/PR+ cancers). Upregulation of the gene encoding the PR is directly mediated by ER, and PR modulates ERα action in breast cancer [14].

**Figure 1:**
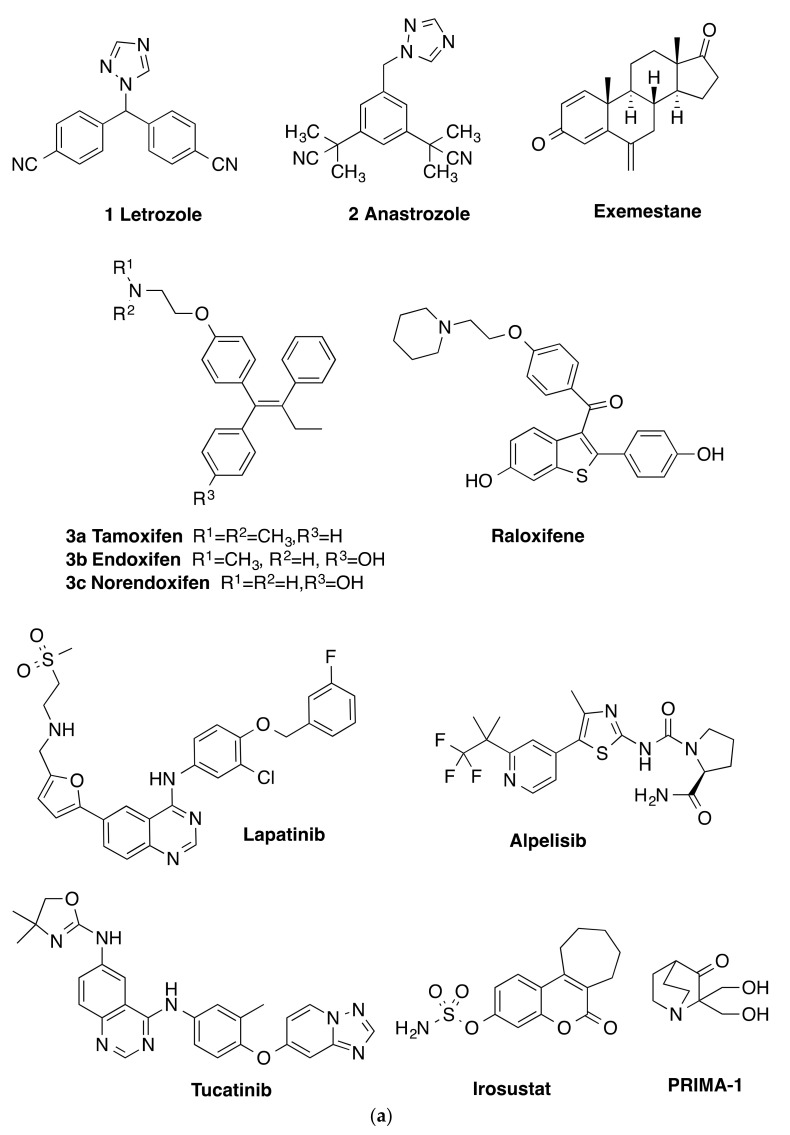

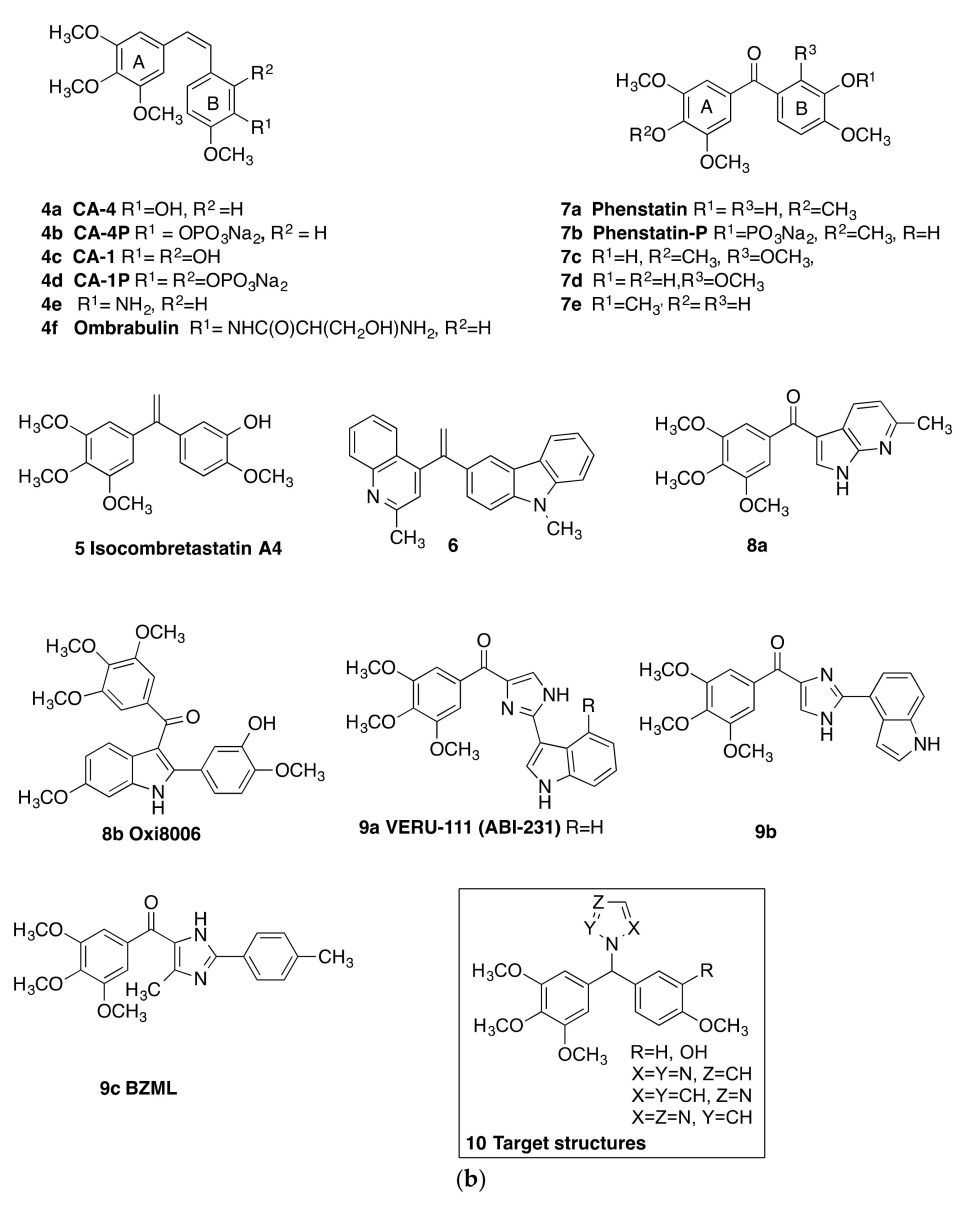
(**a**) Aromatase inhibitors (letrozole, anastrozole, Exemestane), SERMs (Tamoxifen, Endoxifen, Norendoxifen, Raloxifene), kinase inhibitors Lapatinib, Alpelisib, Tucatinib, steroid sulfatase inhibitor Irosustat and mutant p53 inhibitor PRIMA-1, (**b**) Combretastatins **4a–f**, Isocombretastatins **5**,**6**, phenstatins **7a–e,** colchicine binding site inhibitors **8a**,**b**, **9a–c** and target structures **10**.

Aromatase (CYP19A1), a member of the cytochrome P-450 enzyme superfamily, catalyses the aromatisation of C-19 androgens to C-18 estrogens in the final step in estrogen biosynthesis, and it is an attractive target for selective inhibition [15,16,17]. Estrogen deprivation is an effective therapeutic intervention for hormone-dependent breast cancer (HDBC) and has been clinically established by the inhibition of the aromatase enzyme. The aromatase inhibitors (AIs), e.g., letrozole **1** [18], anastrozole **2** [19], and exemestane [20] (Figure 1a), prevent the stimulating effects of estrogen in breast tissue [19], and they are approved in the treatment of a wide spectrum of breast cancers [21]. These AIs have demonstrated superior efficacy in postmenopausal women and have few associated risks apart from reduction in bone density [8,21,22,23], and emerging resistance [24,25]. 

The selective estrogen receptor modulator (SERM) tamoxifen **3a** (Figure 1a) is effective for the treatment of ER+ breast cancer [13]; however, resistance is a clinical problem [26] together with a small increase in incidences of blood clots and endometrial cancers for postmenopausal women [27,28]. The potential advantage of the tamoxifen metabolites endoxifen (**3b**) and norendoxifen (**3c**) in endocrine-refractory metastatic breast cancer is reported [29]. Breast cancers that are (ER+/PR+) are likely to respond to hormone therapy such as tamoxifen and anastrozole [23], while the prophylatic use of tamoxifen, raloxifene, or anastrozole is recommended for postmenopausal women at high risk of developing breast cancer [30,31]. Approximately 20% of breast cancers overexpress the human epidermal growth factor receptor 2 (HER2), which promotes the growth of cancer cells. 

Effective treatments for HER2+ breast cancers include the monoclonal antibody trastuzumab [32], the antibody–drug conjugate ado-trastuzumab emtansine [33], and the dual tyrosine kinase inhibitor lapatinib which targets both the HER/neu and the epidermal growth factor receptor (EGFR) [34]. Breast cancers are classified as triple negative (TNBC) when their growth is not supported by estrogen and progesterone nor by the presence of HER2 receptors. The clinical options for treatment of TNBC are limited due to poor response to hormonal therapy, resulting in low 5-year survival rates [35]. There is extensive diversity among breast cancer patients, and each sub-type of breast cancer has unique characteristics. The identification of sub-type-specific network biomarkers can be useful in predicting the survivability of breast cancer patients [36].

FDA-approved drugs for breast cancer in 2019 include the antibody–drug conjugate Fam-trastuzumab deruxtecan [37] (HER2-directed antibody and topoisomerase inhibitor) for the treatment of unresectable or metastatic HER2-positive breast cancer [38], the phosphoinositide-3-kinase (PI3Kα) inhibitor alpelisib [39] for the treatment of HER2-negative, PIK3CA-mutated, advanced or metastatic breast cancer [40] and in 2020, tucatinib, an orally bioavailable, small molecule tyrosine kinase inhibitor for patients with HER2-positive metastatic breast cancer [41]. The microtubule-stabilising drugs paclitaxel, docetaxel, and the epothilone ixabepilone were approved for use in patients with metastatic breast cancer (MBC), alongside the microtubule destabilising vinca alkaloid eribulin [42,43]. The FDA recently granted accelerated approval to the antibody–drug (topoisomerase inhibitor) conjugate sacituzumab govitecan (Trodelvy) for previously treated metastatic TNBC [44], while ladiratuzumab vedotin (a LIV-1-targeted antibody linked to the microtubule-disrupting agent monomethyl auristatin E (MMAE)) is in clinical trials for locally advanced or metastatic triple-negative breast cancer [45]. The steroid sulfatase inhibitor (STS) e.g., STX64 (Irosustat) has entered clinical trials for ER+ locally advanced or metastatic breast cancer [46], while inhibitors of mutant p53, e.g., PRIMA-1 and PRIMA-1^MET^, overexpressed in TNBC have been demonstrated to be effective in vitro [47].

Combretastatins CA-4 **4a**, (phosphate prodrug **4b**), CA-1 **4c** (phosphate prodrug **4d**), **4e**, and serine prodrug ombrabulin **4f** (Figure 1b) have demonstrated impressive antiproliferative potency with microtubule destabilising and anti-vascular effects in many cancers, including breast cancer [48,49,50]. While many structurally related colchicine binding site inhibitors have been reported [51,52,53], problems associated with the poor water solubility and isomerisation causing an extensive loss of potency have hampered the progression of combretastatins in clinical trials [54,55]. We have previously reported the synthesis of a series of CA-4 analogues with structures based on the conformationally constrained 2-azetidinone ring, demonstrating potent activity in breast cancer cells [56]. Triazole [57,58] imidazole [59,60], and pyridine-containing analogs [61] of CA-4 are also reported with antiproliferative activity in human cancer cell lines. *Iso*combretastatin A4 **5** [62] and 1,1-diheterocyclic ethylenes derived from quinaldine and carbazole e.g., **6** [63] and related conjugates [64] display potent antiproliferative effects in cancer cells and induce G_2_/M cell cycle arrest [65]. The related benzophenone phenstatin **7a** (Figure 1b) [66], together with its sodium phosphate prodrug (**7b**) and metabolites **7c–e** [67], show potent activity in cancer cells and microtubule destabilising activity. The imidazole and indole heterocycles are widely recognised as nuclei of great interest in the design of molecules with anti-tumour activity [68,69]. Related fused-ring heterocyclic structures such as imidazo[2,1-*b*][1,3,4]thiadiazoles with potent antiproliferative activity have also been reported [70]. A variety of compounds structurally related to phenstatin, which contain the heterocycles indole and imidazole, have been synthesised and subsequently evaluated for antimitotic effects and vascular-disrupting effects in cancer cells [71,72]. The azaindole **8a** and 2-aryl-3-aroylindole (OXi8006) **8b** are cytotoxic against selected human cancer cell lines and strongly inhibit tubulin assembly [73,74]. Examples of novel imidazole and indole containing compounds e.g., **9a** [75], **9b** [76], and **9c** (BZML) [77] have been developed as potent tubulin polymerisation-targeting antiproliferative agents. The imidazole derivative BZML **9c** is a novel colchicine binding site inhibitor, which also overcomes multidrug resistance by inhibiting P-gp function and inducing mitotic catastrophe [77]. Compounds such as **9a** (VERU-111), **9b**, and **9c** (BZML) containing both imidazole and indole nuclei exhibit potent activity against a panel of cancer cell lines, are not substrates of P-glycoprotein, and inhibit tumour growth in paclitaxel-resistant cell lines. **9c** inhibits tumour growth and metastasis in vivo [75,76,77].

A number of approaches to the design of dual targeting breast cancer agents have been reported e.g., ER/tubulin [78], tubulin/HSP90 [79], tubulin/HSP27 [80], ER/AI e.g., norendoxifen [81,82], and endoxifen [83], sulfatase/AI [84], tubulin/sulfatase [85,86] and tubulin/angiogenesis (vascular endothelial growth factor receptor-2 (VEGFR2) [87]. We now report the synthesis and biological evaluation of a series of 1-(diarylmethyl)-1*H*-1,2,4-triazoles, 1-(diarylmethyl)-2*H*-1,2,3-triazoles, and 1-(diarylmethyl)-1*H*-imidazoles, which are designed as hybrid scaffolds derived from the benzophenone structure of the tubulin targeting phenstatin **7a**, together with the 1,2,4-triazole of the aromatase inhibitor letrozole **2** [88]. These compounds are designed to provide a selective anti-tumour effect by targeting tubulin polymerisation and also would be effective by inhibiting estrogen production. Although aromatase inhibitors such as letrozole are widely used in the treatment of breast cancer, dual tubulin–aromatase inhibitors have not been reported to date. 1-(Diarylmethyl)-1*H*-1,2,4-triazole and 1-(diarylmethyl)-1*H*-1,2,4-imidazole derivatives have been previously investigated as dual aromatase-steroid sulfatase inhibitors [89]. The target structures **10** are shown in Figure 1b. In addition, a number of related compounds containing the cyclic amines pyrrolidine, piperidine, and piperazine are investigated. We wished to develop this strategic approach with the aim of targeting dual tubulin–aromatase inhibition and have investigated a series of dual-targeting inhibitors.

## 2. Results and Discussion

### 2.1. Chemistry

**Scheme 1:**
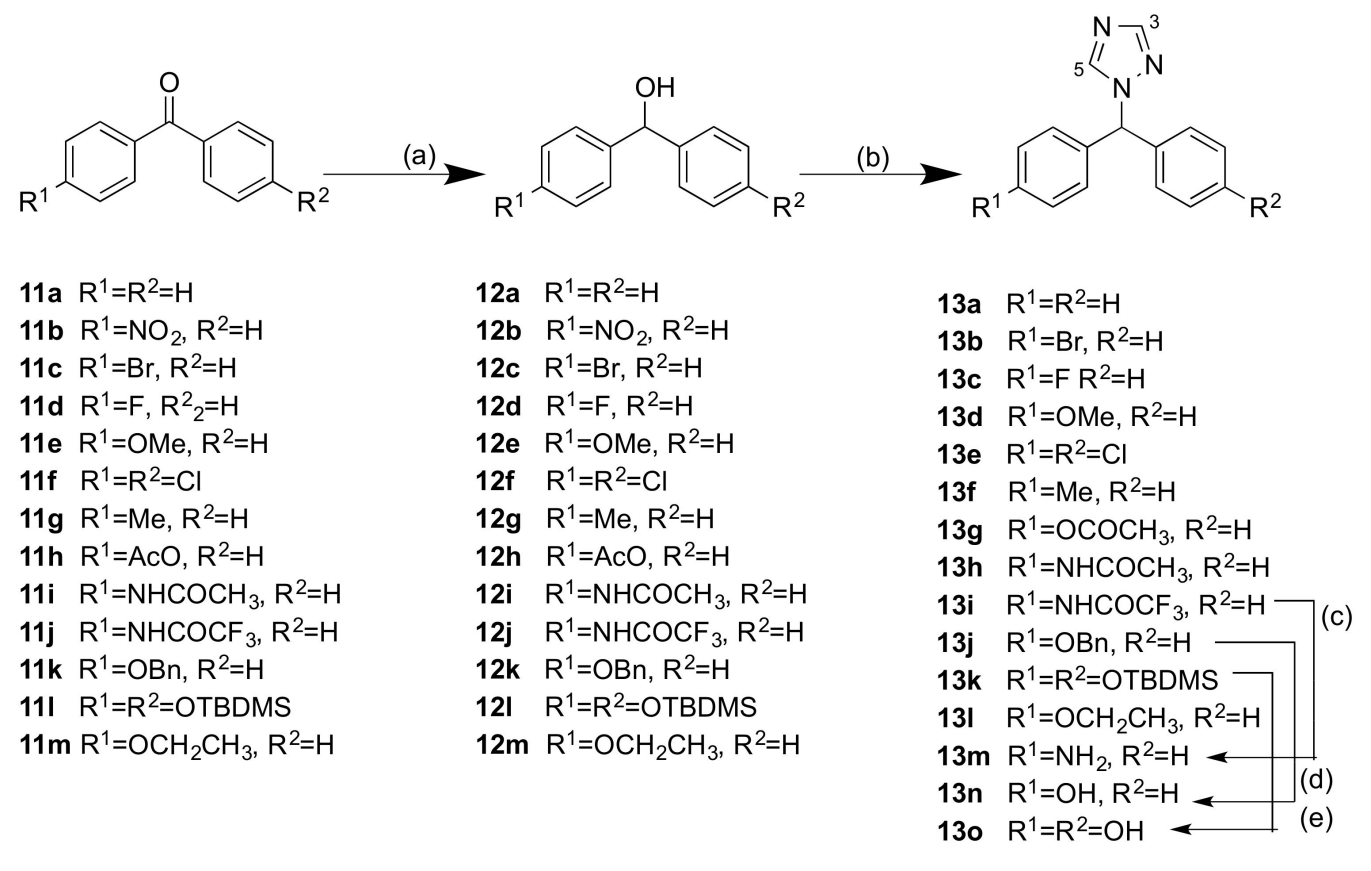
Synthesis of compounds **13a–13o** (Series 1). Reagents and conditions: (**a**) NaBH_4,_ MeOH, 0 °C, (38–100%); (b) 1,2,4-triazole, *p*-TSA, toluene, 4 h, 120 ^o^C, microwave open vessel, (3–98%); (**c**) K_2_CO_3_, MeOH, H_2_O, 20 °C, 72 h, (16%); (**d**) H_2_, Pd(OH)_2_, ethyl acetate, 20 °C, (67%); (**e**) TBAF, THF, 0 °C, (90%) [TBDMS, *tert*-butyldimethylsilyl; Bn, CH_2_C_6_H_5_].

**Scheme 2:**
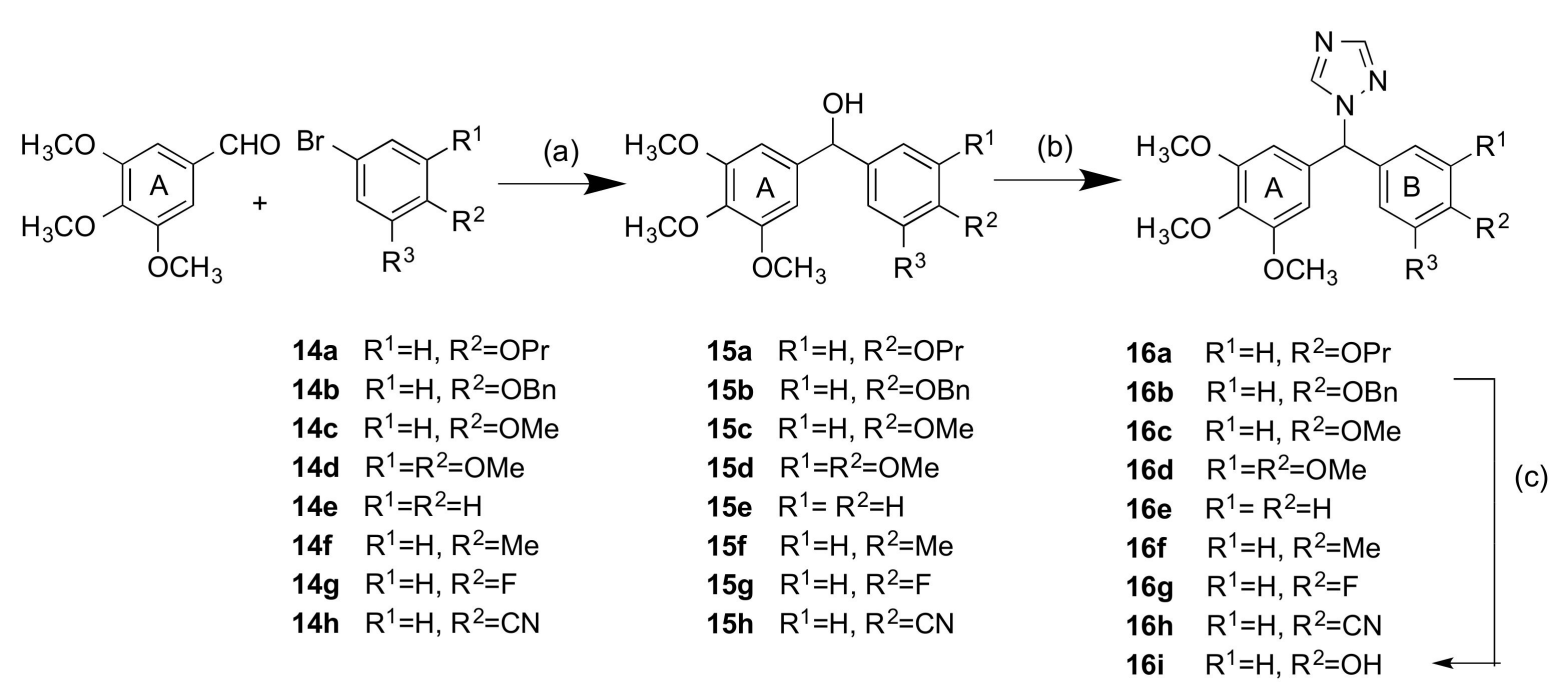
Synthesis of letrozole-phenstatin hybrid compounds **16a–16i** (Series 2a). Reagents and conditions: (**a**) *n*-BuLi, THF, −78 °C, 1.5 h, (21–89%); (**b**) 1,2,4-triazole, *p*-TSA, toluene, 4 h, 120 °C, microwave open vessel, (34–93%); (**c**) Pd(OH)_2_, H_2_, ethyl acetate, 20 °C, (49%). [Bn, CH_2_C_6_H_5_].

**Scheme 3:**
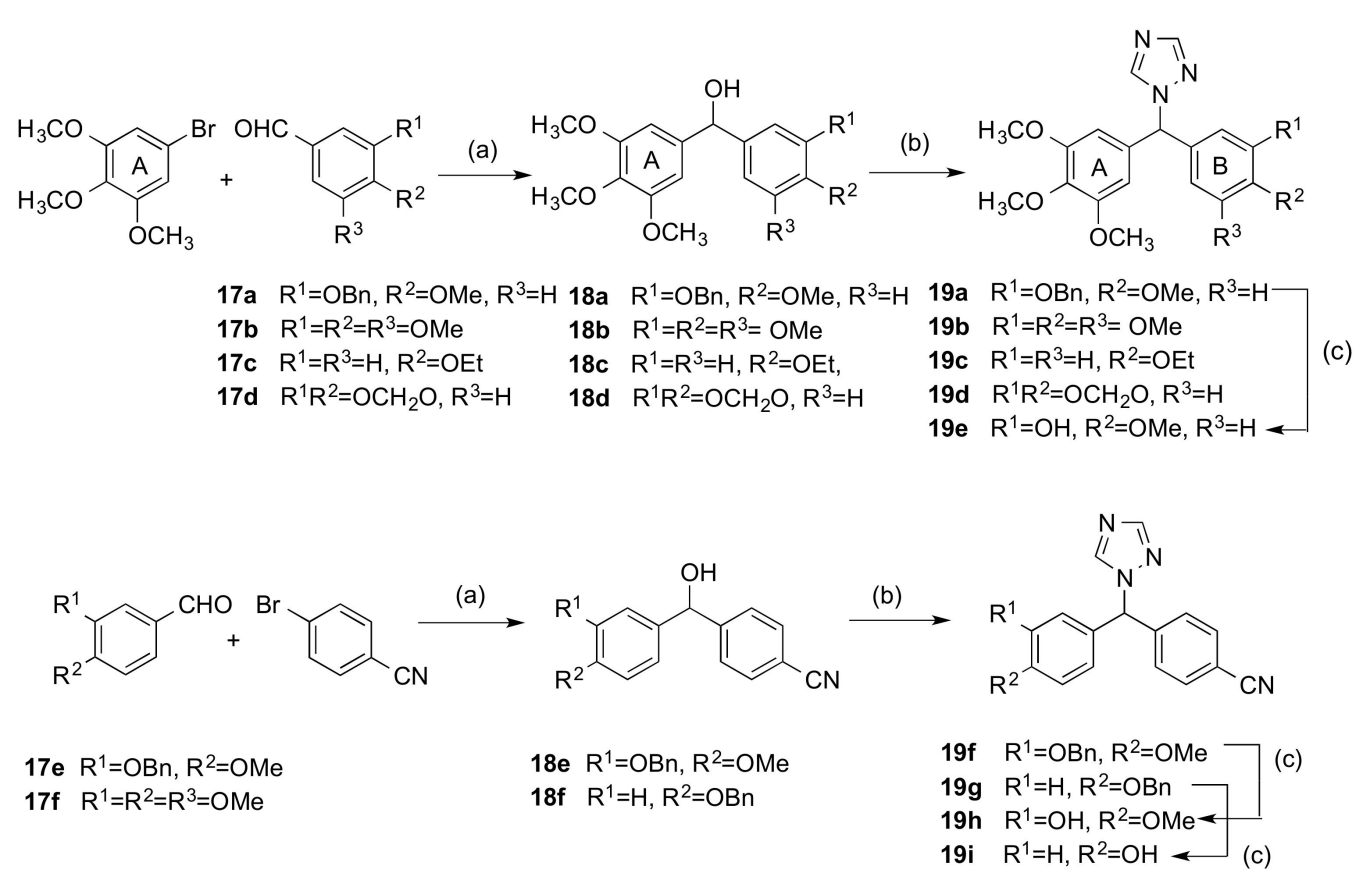
Synthesis of letrozole-phenstatin hybrid compounds **19a-19e** (Series 2a) and 19f–19i (Series 2b). Reagents and conditions: (**a**): *n*-BuLi, dry THF, −78 °C,1.5 h, (16–88%); (**b**) 1,2,4-triazole, *p*-TSA, microwave open vessel, 4h, (64–95%); (**c**) Pd(OH)_2_, H_2_, (66–82%). [Bn: CH_2_C_6_H_5_].

**Scheme 4:**
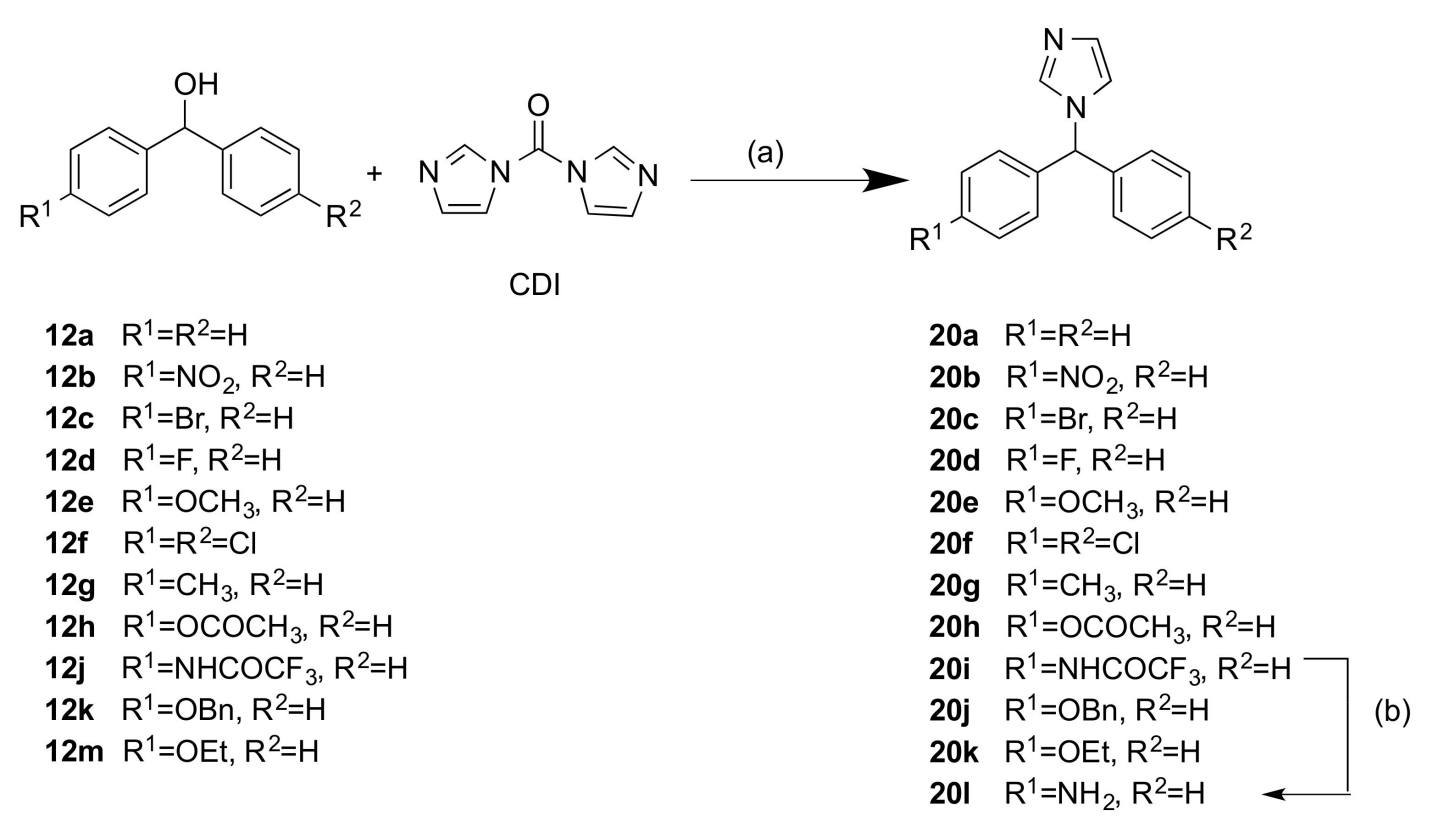
Synthesis of hybrid imidazole-phenstatin compounds **20a–20l** (Series 3). Reagents and conditions: (**a**) CDI, CH_3_CN, reflux, 3 h, (10–64%); (**b**) K_2_CO_3_, MeOH, H_2_O, 20 °C, 72, (50%). [Bn: CH_2_C_6_H_5_].

**Scheme 5:**
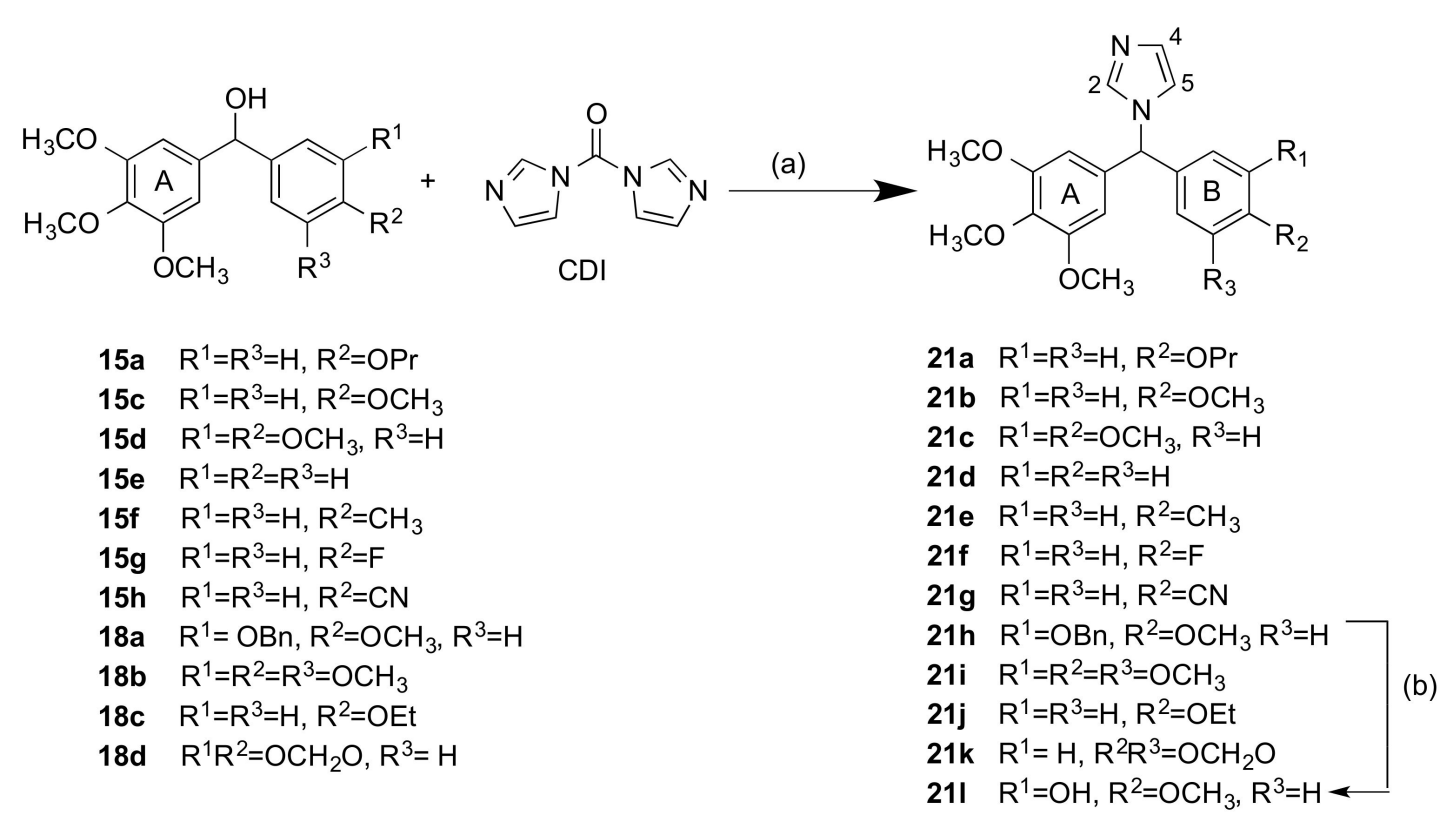
Synthesis of hybrid imidazole-phenstatin compounds **21a–21l**, (Series 4). Reagents and conditions: (**a**) CDI, CH_3_CN, reflux, 3 h, (30–100%). (**b**) H_2_, Pd(OH)_2_, ethyl acetate, 20 °C, (93%). [Bn: CH_2_C_6_H_5_].

**Scheme 6:**
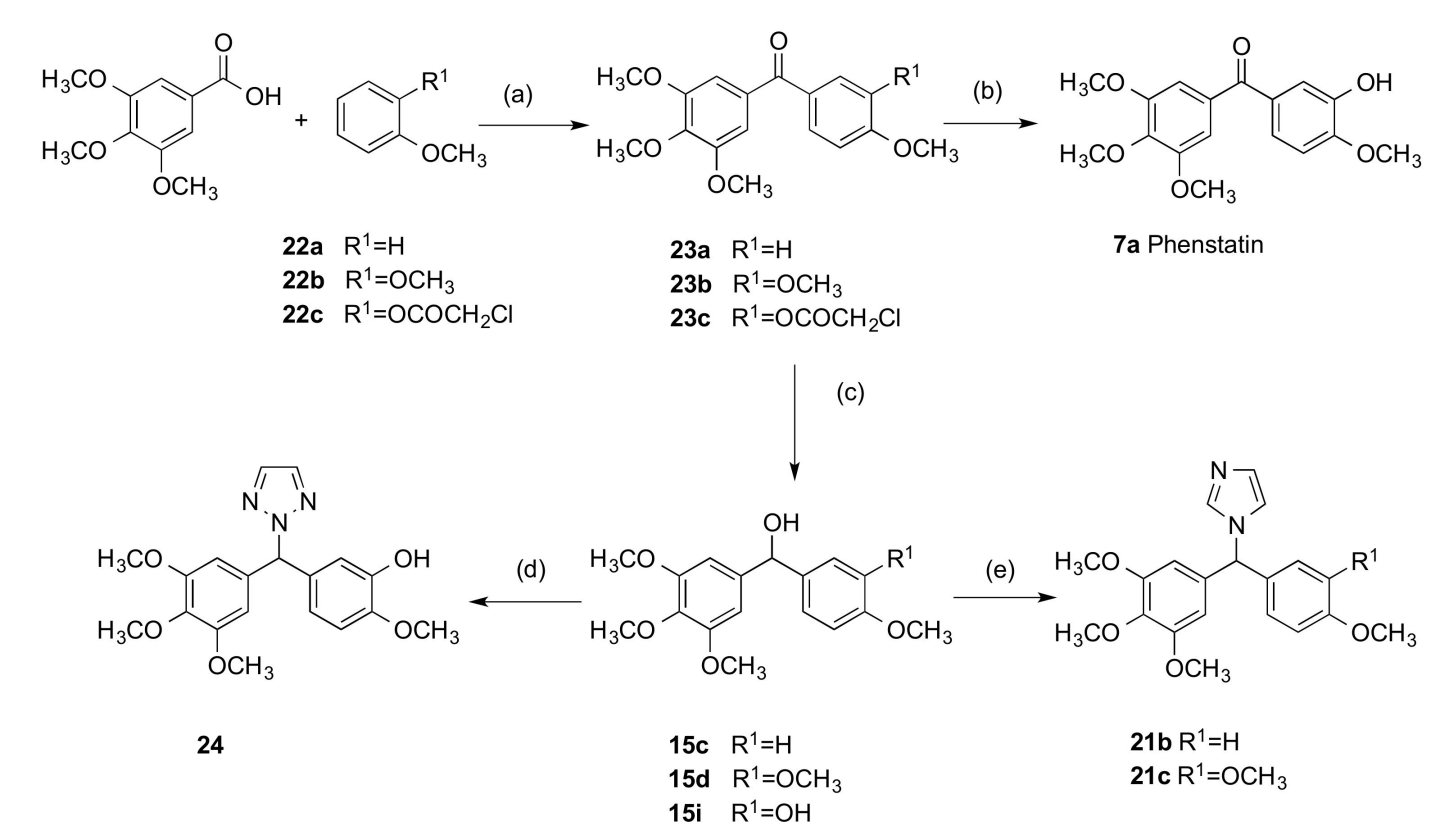
Synthesis of phenstatin 7a and phenstatin hybrids **21b**, **21c**, **24**, (Series 4). Reagents and conditions: (**a**) Eaton’s reagent (P_2_O_5_, CH_3_SO_3_H), 60 °C, 3 h, [**23a** (60%), **23b** (57%), **23c** (17%]); (**b**) sodium acetate, methanol, refux, 2 h, (89%); (**c**) NaBH_4,_ MeOH, 0 ^o^C, [**15c** (50%), **15d** (89%),**15i** (96%)]; (**d**) 1,2,3-triazole, *p*-TSA, toluene, 4 h, 120 °C, microwave open vessel, (77%); (**e**) CDI, CH_3_CN, reflux, 3 h, [**21b** (39%), **21c** (67%)].

**Scheme 7:**
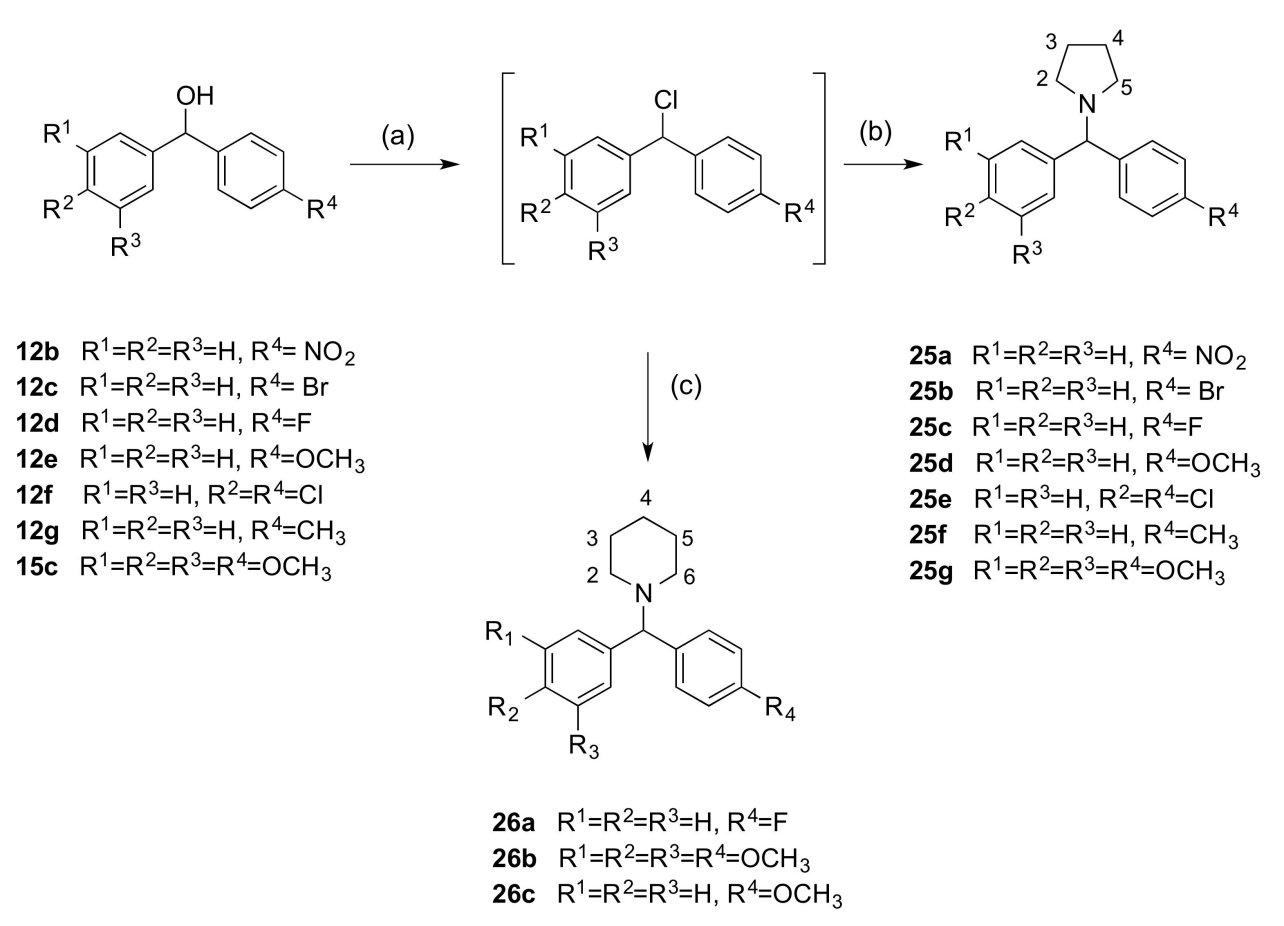
Synthesis of pyrrolidine **25a–g** and piperidine derivatives **26a–c**, (Series 5). Reagents and conditions: (**a**) SOCl_2_,CH_2_Cl_2_, 12 h, 20 ^o^C; (**b**) pyrrolidine, acetonitrile,12 h, reflux (23–93%); (**c**) piperidine, acetonitrile,12 h, reflux (84–91%).

**Scheme 8:**
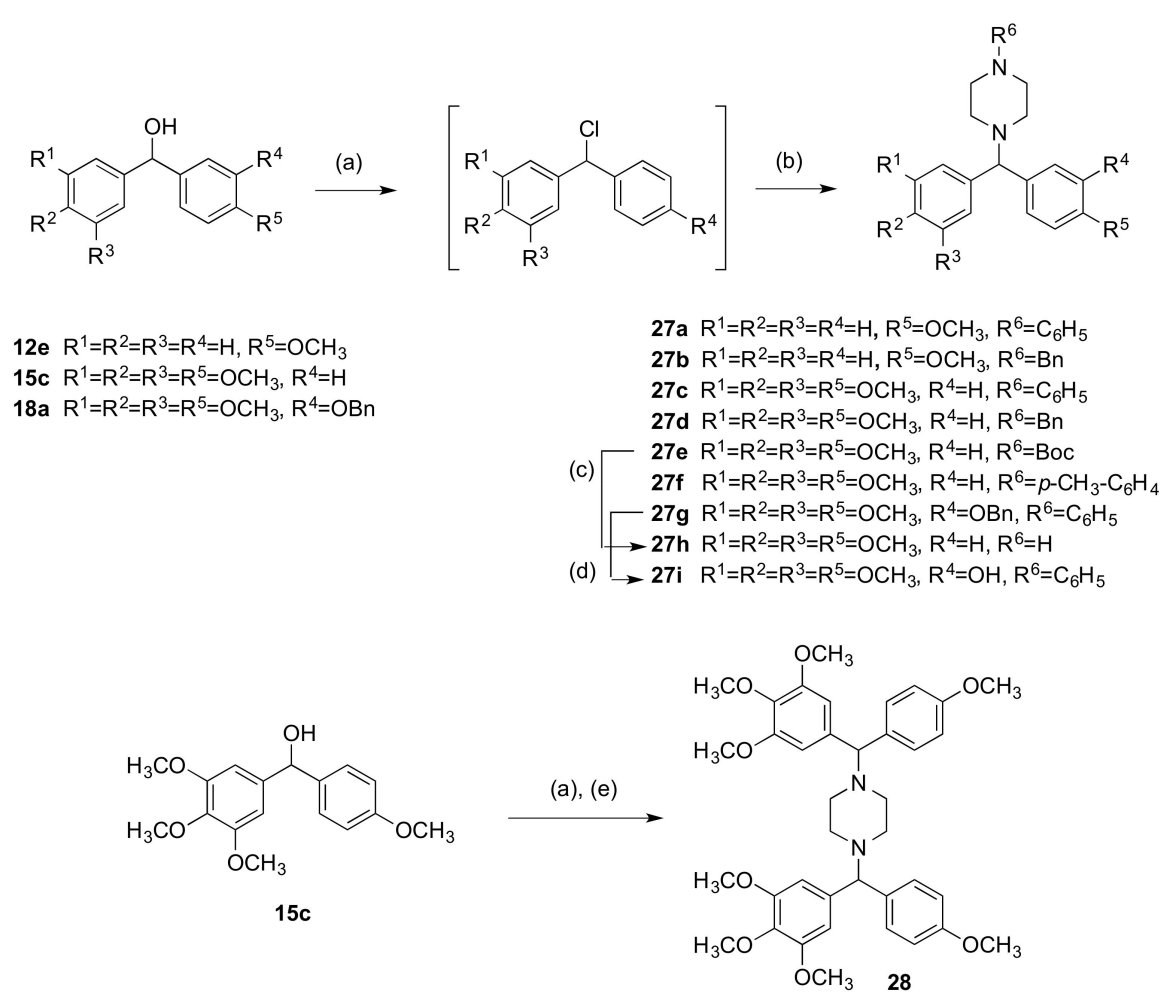
Synthesis of piperazine-phenstatin compounds **27a–g**, 28, (Series 5). Reagents and conditions: (**a**) SOCl_2_, CH_2_Cl_2_,12 h, 20 ^o^C; (**b**) N-phenylpiperazine, N-benzylpiperazine, N-Boc-piperazine or *p*-methoxyphenylpiperazine, acetonitrile, reflux, 12 h (6–80%) (**c**) TFA, CH_2_Cl_2_, 30 min, 20 ^o^C (42%); (**d**) H_2_, Pd(OH)_2_ (45%); (**e**): piperazine, ACN, acetonitrile, reflux, 12 h (12%). [Boc:*tert*-Butoxycarbony; Bn: CH_2_C_6_H_5_].

A series of benzophenone-like compounds related in structure to phenstatin **7a** were first prepared (**11a–11m**). The carbonyl group of the benzophenone was subsequently reduced to afford a benzhydryl alcohol; the heterocycles 1,2,4-triazole, 1,2,3-triazole, imidazole, piperidine, pyrrolidine, and piperazine were introduced in order to afford the N-benzhydryl-heterocyclic products (Scheme 1, Scheme 2, Scheme 3, Scheme 4, Scheme 5, Scheme 6, Scheme 7 and Scheme 8). These compounds were investigated as potential dual-active hybrids: the benzophenone scaffold was designed to interact with tubulin, while the heterocyclic ring was incorporated to target the aromatase enzyme. 

The target compounds are arranged as follows:

Series 1: 1-(Diarylmethyl)-1*H*-1,2,4-triazoles **13a–13o**

Series 2a: 1-(Aryl-(3,4,5-trimethoxyphenyl)methyl)-1*H*-1,2,4-triazoles **16a–i, 19a–e,** 1-(aryl-(3,4,5-trimethoxyphenyl)methyl)-1*H*-1,2,3-triazole **24**

Series 2b: 4-(Aryl-(1*H*-1,2,4-triazol-1-yl)methyl)benzonitriles **19f–i**

Series 3: 1-(Diarylmethyl)-1*H*-imidazoles **20a–l**

Series 4: 1-(Aryl-(3,4,5-trimethoxyphenyl)methyl)-1*H*-imidazoles **21a–l,**

Series 5: 1-(Diarylmethyl)pyrrolidines **25a–g**, 1-(diarylmethyl)piperidines **26a–c** and 1-(diarylmethyl)piperazines **27a–i**, **28**

#### 2.1.1. 1-(Diarylmethyl)-1*H*-1,2,4-triazoles (Series 1 and 2)

The general reaction scheme for the preparation of the 1-(diarylmethyl)-1*H*-1,2,4-triazoles **13a–o** (Series 1) heterocyclic derivatives of benzophenones is shown in Scheme 1. These initial compounds carry a single substituent at the *para* position on one or both of the aryl rings (Cl, F, Br, OH, OCH_3_, CH_3_ etc.). The benzophenones (**11a–m**) were reduced with sodium borohydride to afford the secondary alcohols **12a–m**, in good yields, Scheme 1. Coupling of 1,2,4-triazole with the secondary alcohols **12a–12m** to afford the benzhydryl-1*H*-1,2,4-triazoles **13a–l** was achieved using *p*-TSA as a catalyst in an open-vessel microwave reactor. Amine **13m** was obtained on treatment of amide **13i** with potassium carbonate, while the phenol **13n** was obtained by hydrogenolysis of the benzyl ether **13j** with palladium hydroxide. Deprotection of the silyl ether **13k** with TBAF afforded the diphenol **13o**. In the ^1^H-NMR spectrum of compound **13o**, the singlets (8.41 and 7.97 ppm) were identified as the triazole H5 and H3 respectively, while the singlet at 6.75 ppm corresponds to the methine proton. In the ^13^C-NMR spectrum of **13o**, the signal at 65.12 ppm was assigned to the tertiary carbon, while the signals at 151.52 and 143.93 ppm are assigned to C3 and C5 of the triazole (see Supplementary Information).

Since the potent tubulin-inhibiting activity of the 3,4,5-trimethoxyaryl function is very well documented in colchicine-binding site inhibitors [90], the 1,2,4-triazole heterocycle was next reacted with several phenstatin-type 3,4,5-trimethoxyaryl substituted benzhydryl alcohols in order to maximise the potential tubulin activity in the scaffold structures with aromatase-inhibiting action (Series 2). It was decided to retain in most compounds the 3,4,5-trimethoxyaryl group substitution (ring A) and introduce alternative substituents on the second ring (ring B). A modified synthetic procedure allowing access to the desired benzhydryl alcohol intermediates **15a–h** and **18a–f** is shown in Scheme 2 and Scheme 3 (step *a*) [91]. Scheme 2 shows the alcohols (**15a–h**) obtained by treatment of the appropriate aryl bromides **14a–h** with *n*-butyllithium followed by reaction with 3,4,5-trimethoxybenzaldehyde (A ring) to afford the alcohols **15a–h** in yields of 21–89%. For the preparation of compounds (**18a–d**) (Scheme 3), the A ring was derived from 3,4,5-trimethoxybromobenzene followed by reaction with the appropriate aldehyde **17a–d**. The nitrile-containing compounds **18e**,**f** were similarly obtained from the aldehydes **17e**,**f** and 4-bromobenzonitrile (Scheme 3). The benzhydryl compounds were obtained in good yield after purification via flash column chromatography and the presence of the hydroxyl group was confirmed from IR (ν 3200–3600 cm^−1^). 

Then, the secondary alcohols **15a–h** and **18a–f** were reacted with 1,2,4-triazole to afford the hybrid phenstatin/letrozole compounds **16a–h** and **19a–d**,**f**,**g** as racemates, except for **19b**, (Scheme 2 and Scheme 3, step *b*). The phenolic compounds **16i**, **19e**, **19h**, and **19i** were obtained by hydrogenolysis over palladium hydroxide of the benzyl ethers **16b**, **19a**, **19f**, and **19g** respectively. From the ^1^H-NMR spectrum of compound **16c**, the singlet at 6.62 ppm was assigned the tertiary aliphatic proton. The singlets at 7.91 and 8.01 ppm were assigned to the triazole H-3 and H-5. In the ^13^C-NMR spectrum, the tertiary CH signal was identified at 67.4 ppm, while the triazole ring C3 and C5 signals were identified at 143.5 and 152.3 ppm, respectively.

**Figure 2:**
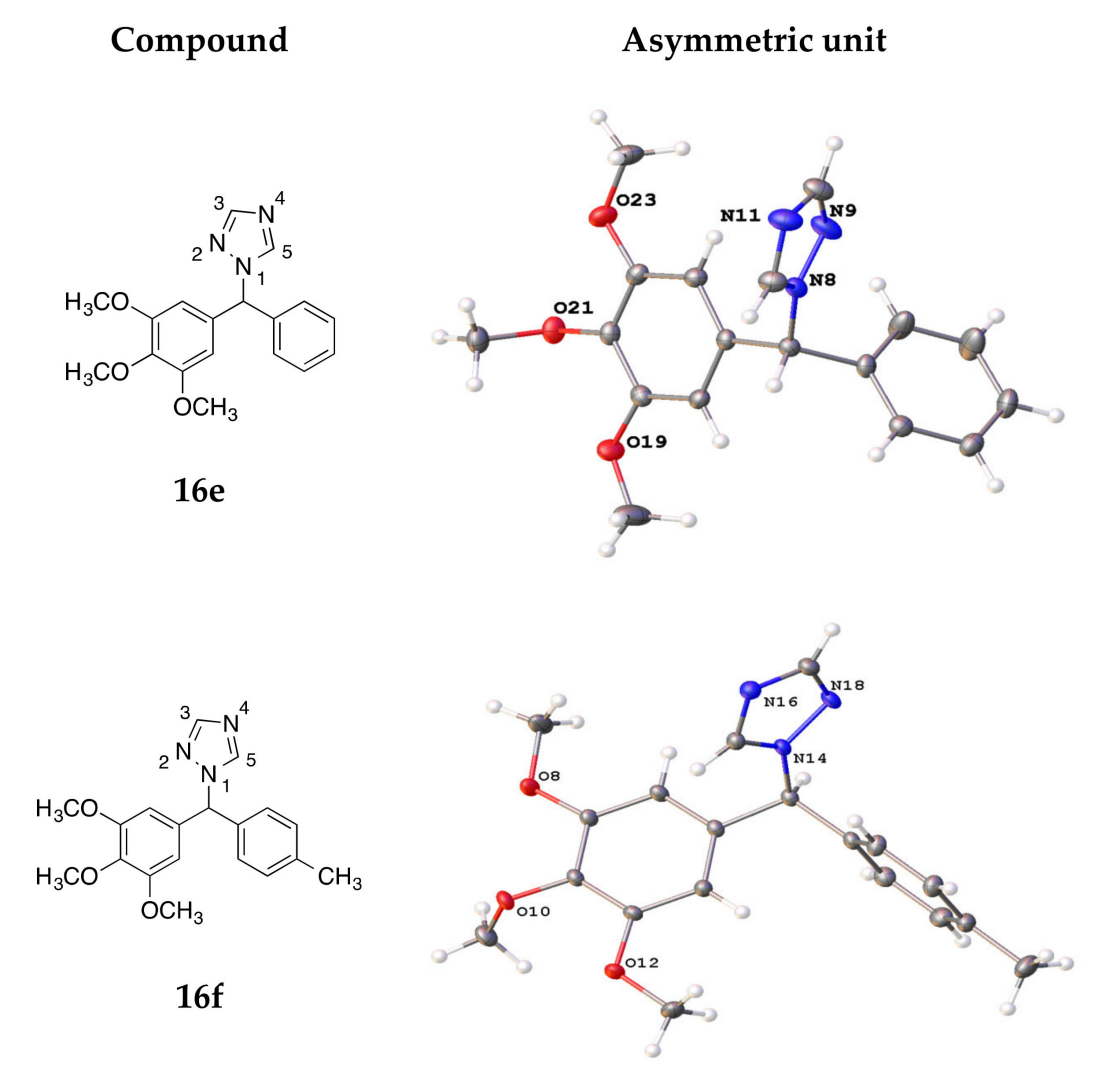

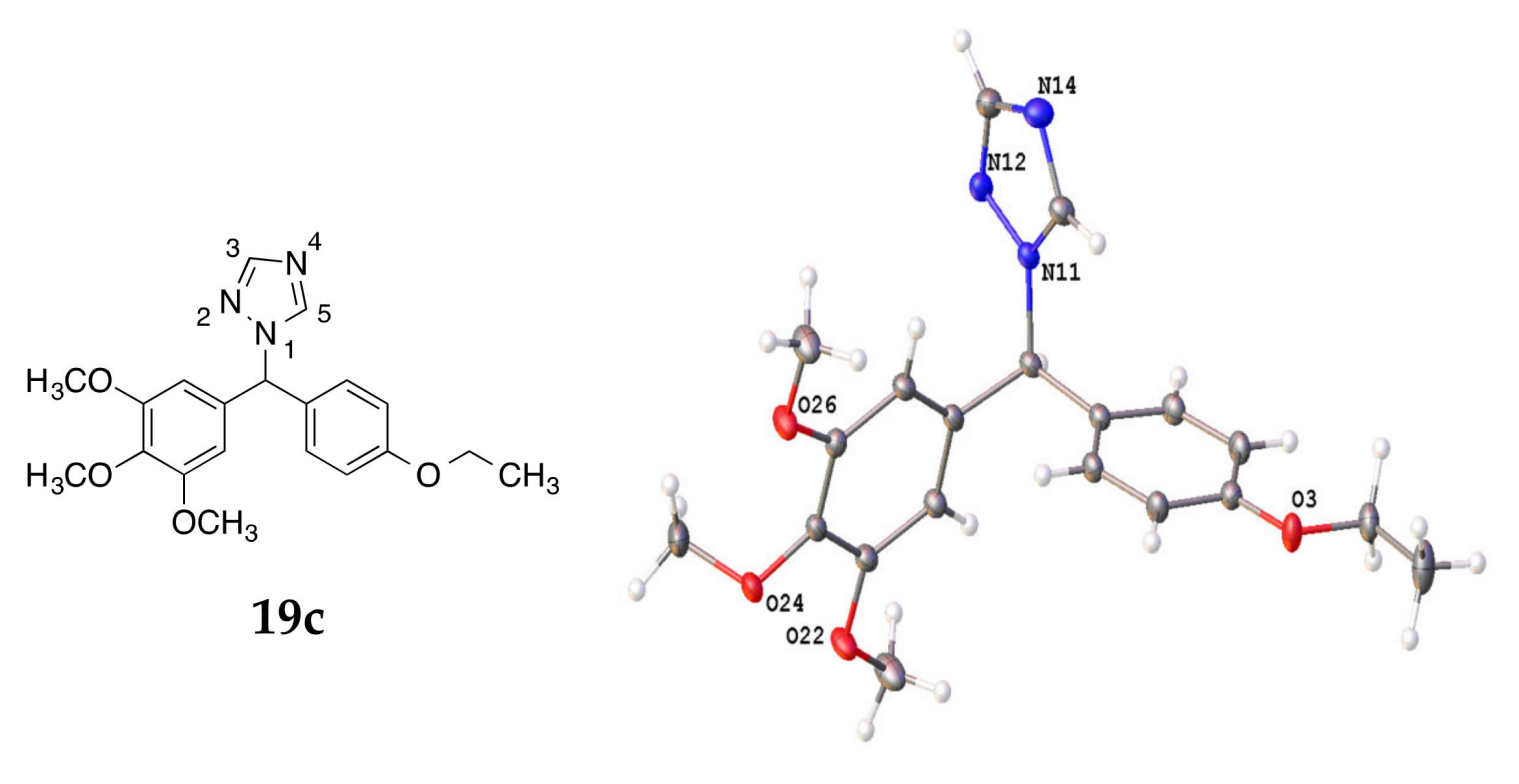
X-ray crystallography structures of **16e**, **16f**, and **19c** with heteroatoms labelled and displacement shown at 50% probability.

X-ray crystal structures of the triazole compounds **16e**, **16f**, and **19c** (recrystallised from dichloromethane/*n*-hexane) are displayed in Figure 2, while the crystal data and structure refinement are displayed in Table 1. The length of the C-N bond between the methine carbon and the triazole N-1 for compounds **16e**, **16f**, and **19c** was measured at 1.470, 1.471, and 1.479 Å, respectively. The N1-N2 bond length was 1.366 Å (**16e)**, 1.363 Å (**16f**), and 1.365 Å (**19c**). The N1-C5 bond length of the triazole ring was observed as 1.334 Å (**16e**), 1.342 Å (**16f**), and 1.343 Å (**19c**). The angle between the methine carbon and the two aromatic rings (Ar-C1-Ar) was measured as 112.51°, 115.08°, and 113.53° respectively for compounds **16e**, **16f**, and **19c**. The corresponding value for the letrozole structure is 114.0°, while the C-N bond between the methine carbon and the triazole N-1 was 1.46 Å [92]. 

#### 2.1.2. 1-(Diarylmethyl)-1*H*-Imidazoles (Series 3 and 4) 

A series of related imidazole-containing compounds were also prepared **20a–l** (Series 3) and **21a–k** (Series 4). The secondary alcohols **12a–h**, **j**, **k**, and **m** were coupled to imidazole using CDI (carbonyldiimidazole) [93] to afford products **20a–k**, Series 3, (Scheme 4, step *a*). The associated carbamate derivatives were not isolated in our reactions [94]. The hydrolysis of **20i** afforded the amine **20l** in 50% yield (Scheme 4, step *b*). Structures were optimised with variations in electron-releasing and electron-withdrawing substituents on the aryl rings. A further series of compounds containing the ring A type 3,4,5-trimethoxyaryl substituents was prepared by reacting alcohols **15a**,**c–h**, and **18a–d** with CDI to afford imidazole products **21a–k**, Series 4, (Scheme 5, step *a*). The benzyl ether **21h** was treated with Pd(OH)_2_ to afford the phenol **21l** as a racemate in 93% yield (Scheme 5, step *b*). In the ^1^H NMR spectrum of compound **21i**, the imidazole H4 was observed as a singlet at 6.88 ppm, while the H2 and H5 were observed at 7.44, and 7.12 ppm, respectively. The singlet at 6.38 ppm was assigned to the tertiary aliphatic CH. From the ^13^C-NMR spectrum, the aliphatic tertiary CH was identified at 65.2, while the signals at 138.0, 129.4, and 119.4 ppm were assigned to the imidazole C2, C4, and C5, respectively.

**Figure 3:**
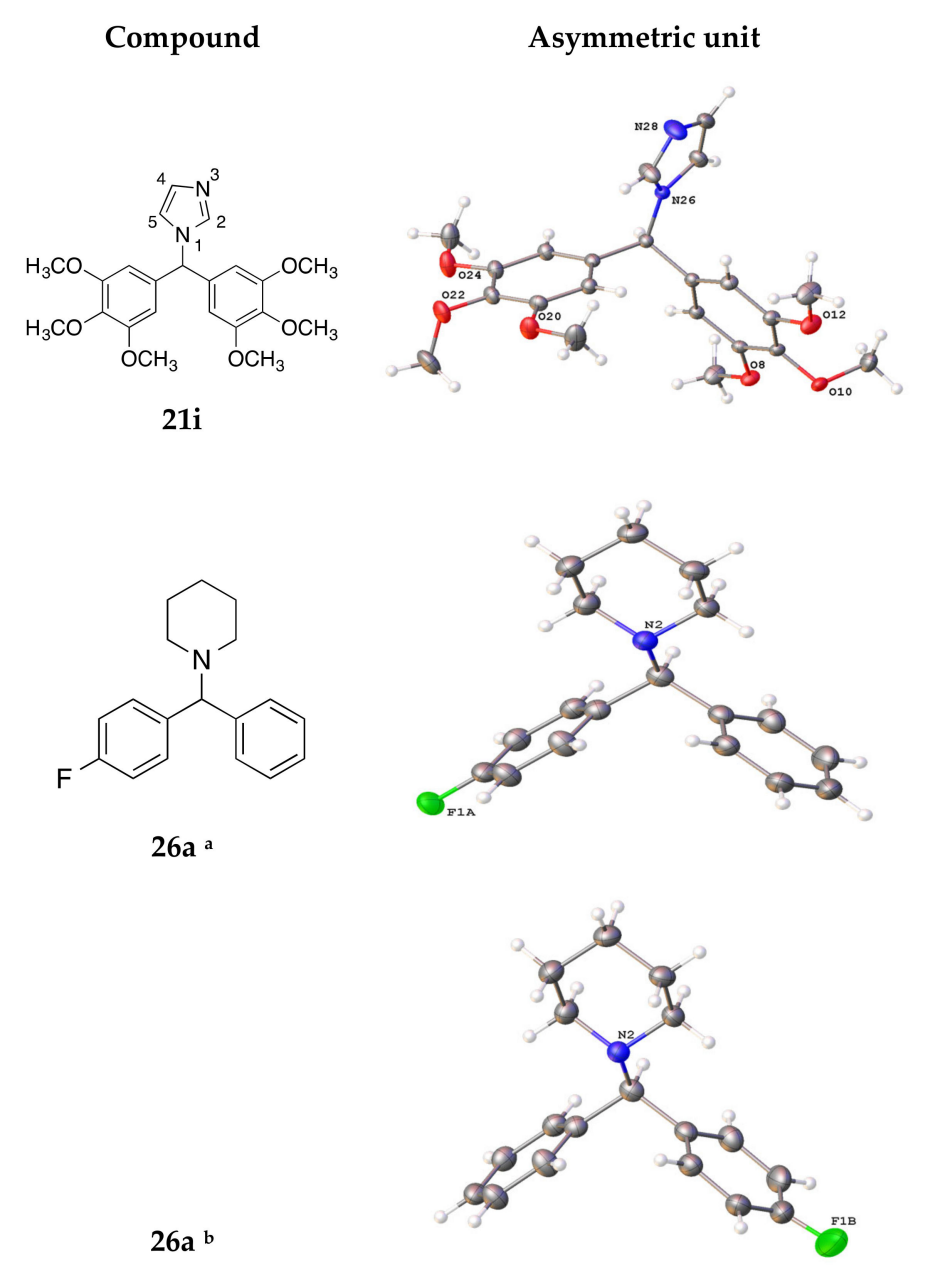
X-ray crystallography of compounds **21i** and **26a** with heteroatoms labelled and displacement shown at 50% probability. ^a^ Disordered molecular structure of **26a** showing the major occupied moiety F1a. ^b^ Disordered molecular structure of **26a** showing the minor occupied moiety F1B.

Single crystal X-ray analysis was obtained for compound **21i** (recrystallised from dichloromethane/*n*-hexane), and the crystal structure is shown in Figure 3. The crystal data and structure refinement for compound **21i** are displayed in Table 1. The angle between the methine carbon and the aryl rings (114.16°) and also the bond length between the methine carbon and the N-1 imidazole nitrogen (1.471 Å) were similar to the corresponding values obtained for the triazole compounds **16e**, **16f**, and **19c** (Table 1). The bond angles between the aryl rings and the imidazole ring were determined as 111.36° and 111.80°, also similar to the corresponding values of 109.99° and 112.6° reported for letrozole [92].

An alternative approach for the preparation of phenstatin and related azole compounds using a Friedel–Crafts acylation with Eaton’s reagent was also investigated (Scheme 6) [67]. 3,4,5-Trimethoxybenzoic acid was reacted with anisole (**22a**), 1,2-dimethoxybenzene (**22b**), or compound **22c** (prepared by the protection of 2-methoxyphenol with chloroacetyl chloride) using Eaton’s reagent (readily prepared from phosphorus pentoxide and methanesulfonic acid) to afford respectively benzophenones **23a**, **23b**, and **23c** (Scheme 6, Step a). Then, these benzophenones were reduced to the benzhydryl alcohols **15c**, **15d**, and **15i**, respectively with sodium borohydride (Scheme 6, step a), with the concomitant removal of the chloroacetyl protecting group of **23c**. Although requiring an additional step, this method was followed after the reaction of the aryl bromide with the aldehyde to afford the alcohol as shown in Scheme 2 and Scheme 3 was not successful or did not afford a sufficient quantity of product for the next step e.g., for compound **15d**, the overall yield increased to 51% compared with 30%. Then, compounds **15c** and **15d** were treated with CDI azole to afford the imidazole-containing products **21b** and **21c** (Scheme 6, step e).

The phenol **15i** was also reacted with 1,2,3-triazole to afford the product **24** in 77% yield, Series 4, (Scheme 6, step d). Compound **24** is the only phenstatin derivative substituted with 1,2,3-triazole synthesised in this project and was investigated for comparison with the 1,3,4-triazole compound series. In the ^1^H NMR spectrum of **24**, the signal at 6.99 ppm was assigned to the tertiary CH. Interestingly, the two protons of the 1,2,3-triazole ring were observed as a singlet with an integration of 2H at 7.83 ppm, while the signal at 134.9 ppm in the ^13^C-NMR spectrum of **24** was assigned to the C4 and C5 of the triazole ring, indicating that alkylation occurred at N2 of the 1,2,3-triazole [95]. The alkylation of 1,2,3-triazoles may result in the formation of regioisomers depending on the reaction conditions e.g., solvent, temperature, and catalyst used [96]. The signal for the tertiary CH was observed at 71.0 ppm. The benzophenone **23c** was also used in the preparation of phenstatin **7a [67]**; the deprotection of **23c** by reaction with sodium acetate afforded **7a** in 89% yield (Scheme 6, step *b)*, which was used as a positive control in the cell viability tests. 

#### 2.1.3. 1-(Diarylmethyl)Pyrrolidines, 1-(Diarylmethyl)Piperidines, and 1-(Diarylmethyl) Piperazines (Series 5)

The preparation of a series of benzhydryl derivatives substituted on the tertiary carbon with the heterocycles pyrrolidine, piperidine, and piperazine was next investigated (Series 5, Scheme 7 and Scheme 8). These products allow a comparison of biochemical activity with the related imidazole and triazole compounds from Series 1–4. The advantages of incorporating such heterocyclic rings into drugs are well known; i.e., they can increase the lipophilicity, polarity, and aqueous solubility of the drug [97]. In particular, piperazine is ranked 3^rd^ among the 25 most common heterocycles contained in FDA-approved drugs [98]. In the present work, the corresponding secondary benzhydryl chloride was prepared from the secondary alcohols **12b–12g**, **15c**, and **18a** using thionyl chloride (Scheme 7 and Scheme 8, step *a*) [93]. The intermediate alkyl chlorides were reacted with piperidine to afford products **26a–c** (Scheme 7, step *c*), while reaction with pyrrolidine yielded derivatives **25a–g** (Scheme 7, step *b*). 

An alternative synthesis of 1-(diarylmethyl)piperidines is reported using a copper(I)-catalysed coupling reaction of aryl boronic acids with N,O-acetals and N,N-aminals [99]. All compounds are racemates apart from compound **25e** and were obtained in moderate yields (23–93%). In the ^1^H-NMR spectrum of compound **25b**, the multiplets at 1.71–1.80 and 2.35–2.43 ppm were assigned to the pyrrolidine methylene protons at H-3,4 and H-2,5 respectively, while the tertiary CH was observed as a singlet at 4.11 ppm. In the ^13^C-NMR spectrum, the pyrrolidine C-3 and C-2 signals were at 23.5 and 53.6 ppm, respectively. The signal at 75.7 ppm was assigned to the tertiary carbon. Single crystal X-ray analysis for compound **26a** is shown below in Figure 3 (obtained by crystallisation in dichloromethane/*n*-hexane). The crystal data and structure refinement for compound **26a** are displayed in Table 1. In **26a**, the disordered fluorine was modelled in two positions with occupancies of 84% and 16%. The C1-N bond length was observed as 1.473 Å and the central C_14_-C_1_-C_8_ and C_14_-C_1_-N_2_ angles were observed as 109.28° and 112.09°, respectively. The piperidine ring bond lengths were 1.471 Å (N2-C3), 1.474 Å (N2-C7), and 1.514 Å (C3-C4), which differ from the N1-C bond length of the triazole ring 1.334 Å due to unsaturation.

As a further extension of this research, a related series of piperazine-containing compounds was prepared by coupling selected secondary alcohols with the appropriate piperazine derivative (Series 5, Scheme 8). The preparation of diarylmethylamines has been reported by Le Gall et al. by reaction of the aldehyde and piperidine derivative to a solution of the organozinc reagent in acetonitrile in a Mannich-type reaction [100,101]. The secondary alcohols **12e**, **15c**, and **18a** were treated with thionyl chloride (Scheme 8, step *a*), and the resulting alkyl chloride was used immediately for the next reaction step (step *b*) by addition of the appropriate piperazine (N-phenylpiperazine, N-benzylpiperazine, *p*-methoxyphenylpiperazine, or N-Boc-piperazine) to afford the products **27a–g** in yields up to 80%. For the preparation of compound **27e**, Boc-protected piperazine was used to avoid the possible formation of the dimer. In the ^1^H-NMR spectrum of compound **27d**, the broad signal at 2.47 ppm is assigned to piperazine methylene protons; the singlet at 3.50 ppm is assigned to the benzyl methylene, while the singlet at 4.09 ppm corresponds to the tertiary C-1 proton. The ^13^C-NMR spectrum of compound **27d** further confirms the proposed structure. The signals at 51.8 and 53.3 ppm were characteristic of the piperazine ring protons, the signals at 63.0 ppm and 75.6 ppm are assigned to the benzyl methylene and tertiary CH, respectively. The deprotection of compound **27e** with TFA afforded compound **27h** as a yellow oil (42%), (Scheme 8, step *c*). A palladium-catalysed hydrogenolysis of **27g** afforded the phenolic compound **27i** in 45% yield (step *d*), which is the phenylpiperazine derivative of phenstatin. Its formation was confirmed by IR spectroscopy (3475 cm^−1^). 

When the secondary alcohol **15c** was treated with thionyl chloride followed by an excess of piperazine (5 equivalents), the product obtained was a piperazine dimer **28** (Scheme 8). In the ^1^H-NMR spectrum of the dimer **28**, the broad signal (2.40 ppm) is characteristic of the piperazine methylene protons, while the signal at 4.08 ppm integrating for 2H was assigned to the two tertiary CH protons. In the ^13^C-NMR spectrum, the signal at 52.0 ppm was assigned to the piperazine ring carbons; the signal at 75.7 ppm was assigned to the CH, while the duplication of the aromatic signals confirmed the formation of the product.

### 2.2. Stability Studies

HPLC stability studies were performed on representative compounds **21l** and **24** to establish their stability at different pH systems, which mimic in vivo conditions. Compound **21l** was chosen among these imidazole compounds for HPLC stability studies at three different pH systems; acidic pH 4, pH 7.4, and basic pH 9 (acid pH found in the stomach, basic found in the intestine, and pH 7.4 in the plasma). The degradation of compound **21l** was minimal with 80% of **23l** remaining at both pH 7.4 and pH 9 and 90% at pH 4 after 24 h. The 1,2,3-triazole compound **24** was observed to be most stable at pH 4 with 65% remaining after 24 h compared to 60% at pH 9 and 50% at pH 7.4.

## 3. Biochemical Results and Discussion

### 3.1. In Vitro Antiproliferative Activity in MCF-7 Breast Cancer Cells 

**Figure 4:**
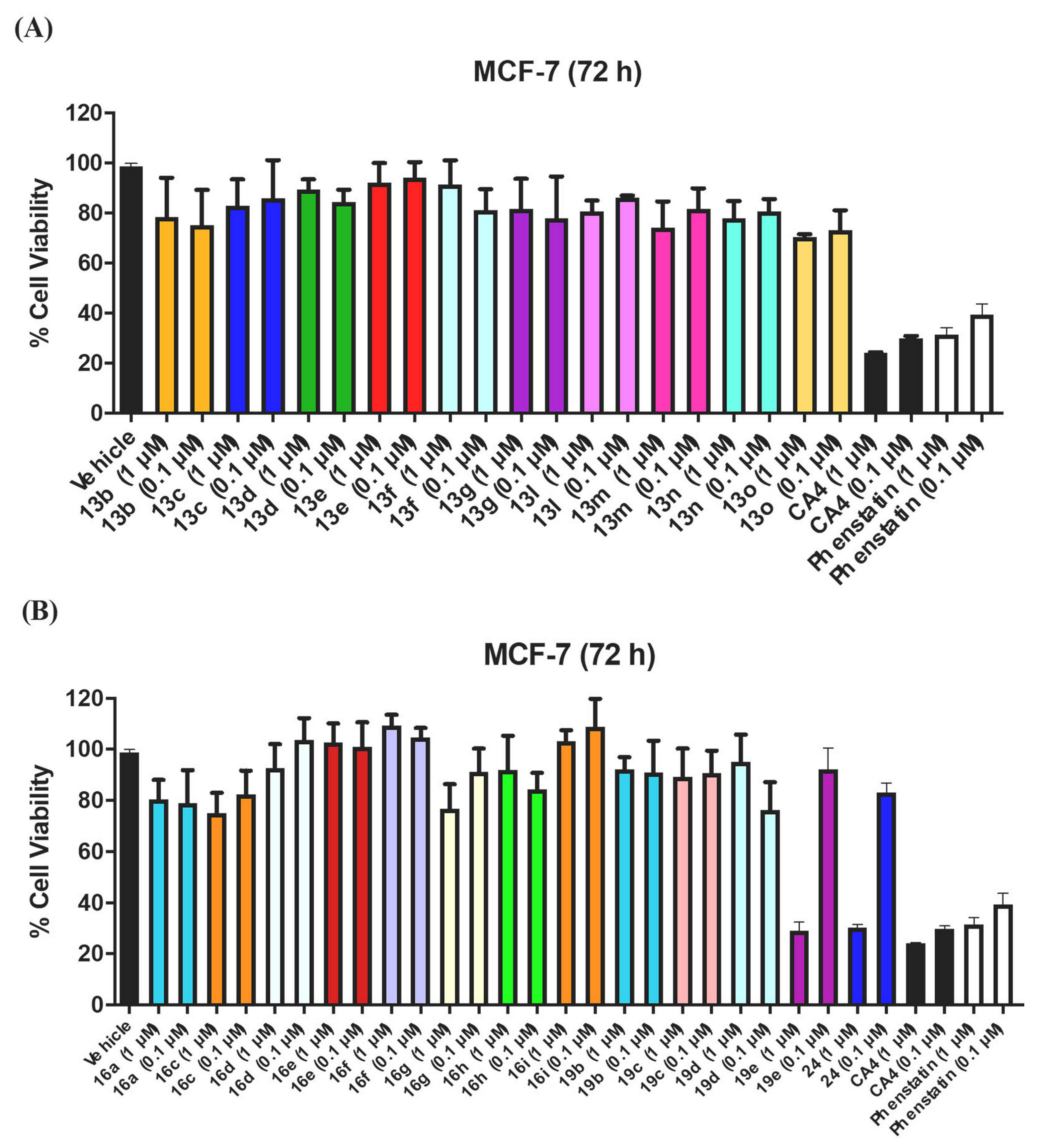
Preliminary cell viability data for (**A**) Series 1: 1-(diarylmethyl)-1*H*-1,2,4-triazoles **13b–g**, **l–o** (**B**) Series 2: 1-(diarylmethyl)-1*H*-1,2,4-triazoles **16a**, **c–i**, **19b–e**, 1-(diarylmethyl)-2*H*-1,2,3-triazole **24** in MCF-7 breast cancer cells. Cell proliferation of MCF-7 cells was determined with an alamarBlue assay (seeding density 2.5 × 104 cells/mL per well for 96-well plates). Compound concentrations of either 1 or 0.1 μM for 72 h were used to treat the cells (in triplicate) with control wells containing vehicle ethanol (1% *v*/*v*). The mean value + SEM for three independent experiments is shown. The positive controls used are CA-4 and phenstatin.

**Figure 5:**
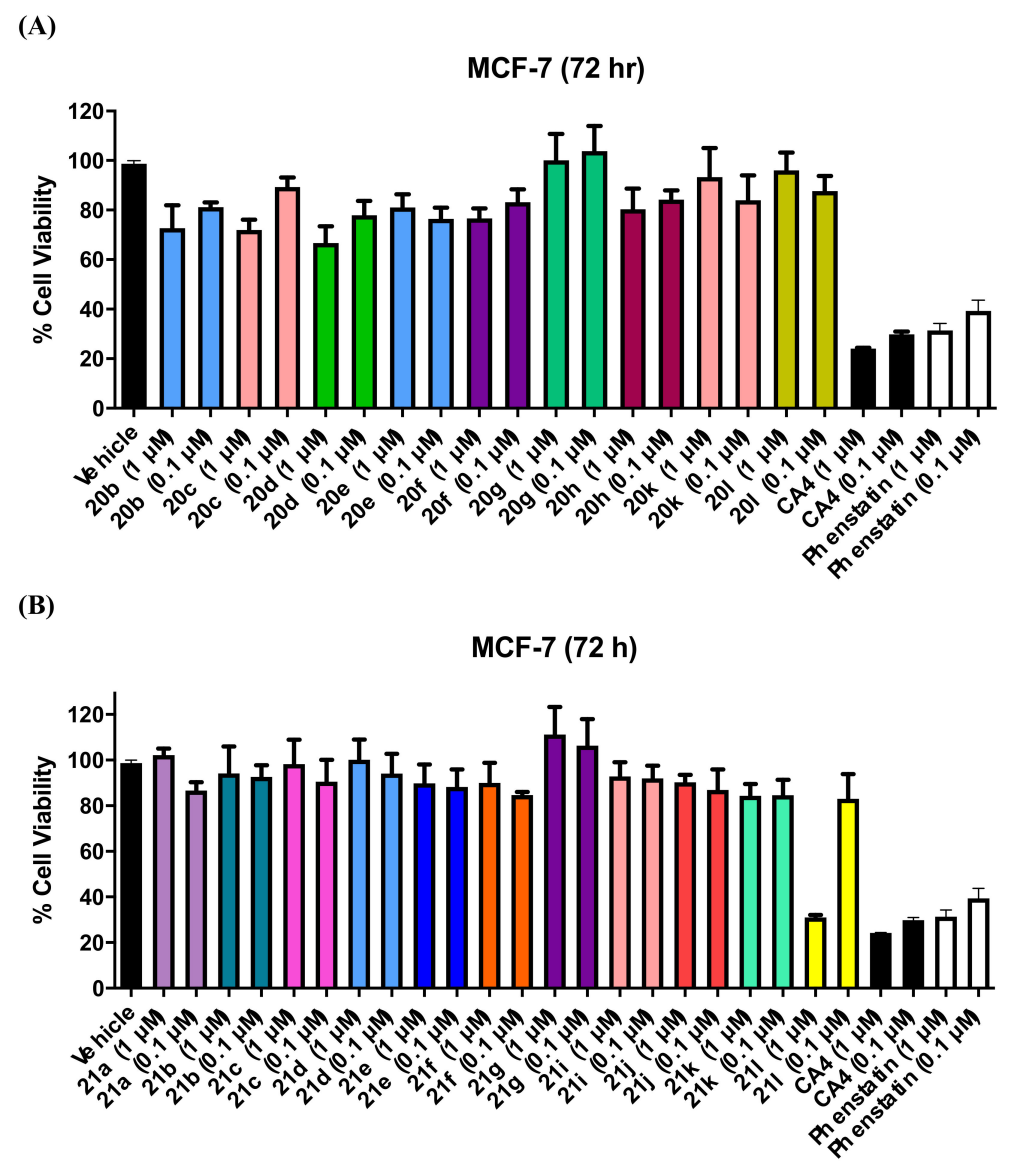
Preliminary cell viability data for (**A**) **Series 3:** 1-(diarylmethyl)-1*H*-imidazoles **20b–h, k, l** and (**B**) **Series 4:** 1-(diarylmethyl)-1*H*-imidazoles **21a–g, i–l** in MCF-7 breast cancer cells. Cell proliferation of MCF-7 cells was determined with an alamarBlue assay (seeding density 2.5 × 10^4^ cells/mL per well for 96-well plates). Compound concentrations of either 1 or 0.1 μM for 72 h were used to treat the cells (in triplicate) with control wells containing vehicle ethanol (1% *v*/*v*). The mean value ± SEM for three independent experiments is shown. The positive controls used are CA-4 and phenstatin.

**Figure 6:**
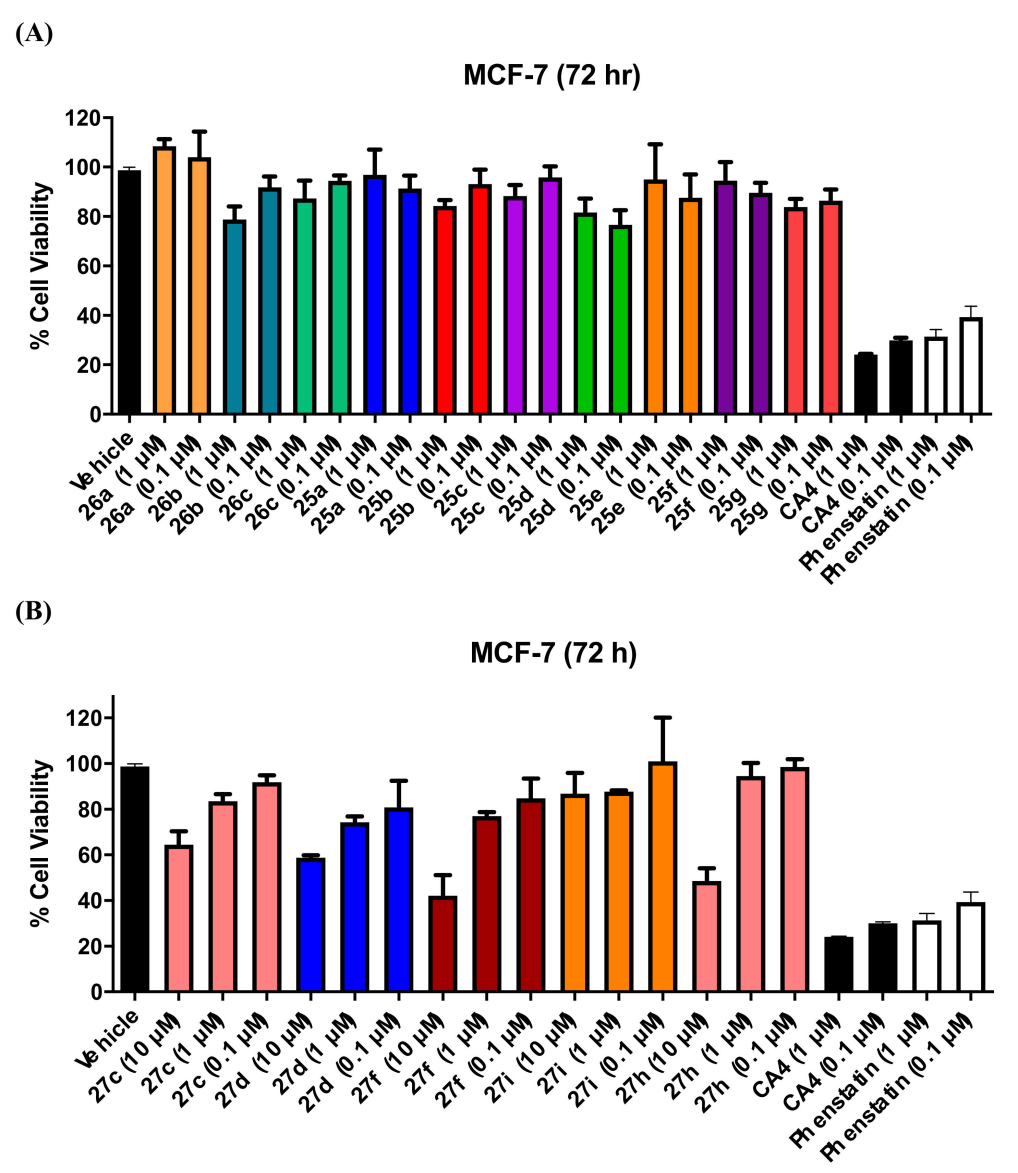
Preliminary cell viability data for (**A**) Series 5: 1-(diarylmethyl)pyrrolidines and 1-(diarylmethyl)piperidines **25a–g, 26a–c** and (**B**) Series 5: 1-(diarylmethyl)piperazines **27c,d,f,i**, and **h** in MCF-7 breast cancer cells. Cell proliferation of MCF-7 cells was determined with an alamarBlue assay (seeding density 2.5 × 10^4^ cells/mL per well for 96-well plates). Compound concentrations of either 10, 1, or 0.1 μM for 72 h were used to treat the cells (in triplicate) with control wells containing vehicle ethanol (1% *v*/*v*). The mean value ± SEM for three independent experiments is shown. The positive controls used are CA-4 and phenstatin.

The antiproliferative activity of the panel of hybrid compounds 1-(diarylmethyl)-1*H*-1,2,4-triazoles (Series 1 and 2) and 1-(diarylmethyl)-1*H*-imidazoles (Series 3 and 4) was initially evaluated in the MCF-7 human breast cancer cell using the standard alamarBlue assay. In addition, a number of related compounds containing the aliphatic amines pyrrolidine, piperidine, and piperazine were investigated (Series 5). The MCF-7 human breast cancer cell line is estrogen receptor (ER)-positive, progesterone receptor (PR)-positive, and HER2 negative. Compounds were initially screened at two concentrations (1 and 0.1 μM) for antiproliferative activity in MCF-7 cells to determine the structure–activity relationship for these hybrid compounds and to identify the most potent compounds for further investigation. Compounds that were synthetic intermediates for the final compound were not screened, as they were not considered as potential actives in the study. The results obtained from this preliminary screen are displayed in Figure 4, Figure 5 and Figure 6. Then, those compounds showing potential activity (cell viability <50%) were selected for further evaluation at different concentrations and in other cell lines. CA-4 (**4a**) (24% viable cells at 1 μM) and phenstatin (**7a**) (30% viable cells at 1 μM) induced a potent antiproliferative effect and were used as positive controls. Ethanol (1% *v*/*v*) was used as the vehicle control (with 99% cell viability). The preliminary results obtained for these novel compounds (Series 1–5) are discussed by structural type.

#### 3.1.1. Series 1: 1-(Diarylmethyl)-1H-1,2,4-Triazoles **13b–g**, **l–o**

The first class of compounds tested 1-(diarylmethyl)-1*H*-1,2,4-triazoles (**13b–g**, **13l–o**, Figure 4A) were weakly active, with 68–90% viability for the two concentrations tested (1 μM and 0.1 μM). These compounds carry a single substituent at the *para* position on one or both aryl rings (Cl, F, Br, OH, OCH_3_, CH_3_, etc.) indicating that the triazole ring alone is not sufficient for the induction of antiproliferative activity in MCF-7 cells. The most active compounds were the diphenolic derivative **13o** with 68% viability (1 μM) and the amino compound **13m** (72% viability 1 μM). It appears that specific substituents are required on both the A and B rings of the benzophenone for activity, as also observed for phenstatin and analogues [67]. 

#### 3.1.2. Series 2: 1-(Aryl-(3,4,5-Trimethoxyphenyl)Methyl)-1*H*-1,2,4-Triazoles **16a**,**c–i**, **19b–e**, **19h**, **19i** and 1-(Phenyl(3,4,5-Trimethoxyphenyl)Methyl)-1*H*-1,2,3-Triazole **24**

Since the potent tubulin inhibiting activity of the 3,4,5-trimethoxyaryl function is very well documented [90], the preliminary screening in MCF-7 cells of the panel of 1,2,4-triazole containing compounds (**16a, c–i**, **19b–e**) synthesised having the 3,4,5-trimethoxyphenyl motif (A ring) together with various substituents on the B ring was next investigated (Figure 4B, two concentrations of 1 μM and 0.1 μM). The most potent compound was identified as **19e** having the characteristic 3-hydroxy-4-methoxyaryl B ring as in phenstatin and CA-4 (29% viability at 1 μM), while the ethanol control (1% *v*/*v*) resulted in 99% viability. Two compounds with moderate activity were identified as **16c** (4-methoxy group in the B ring) with 75% cell viability at 1 μM and **16g** (4-fluoro in B ring) with 77% viable cells at 1 μM. The remaining 3,4,5-trimethoxyphenylmethyl-1*H*-1,2,4-triazole compounds investigated having various substituents on the B ring e.g., 4-F, 4-CN, 4-OH, 4-CH_3_ were not as potent as the lead compound with viability >80% at 1 μM, while compounds **19h** and **19i** were found to be inactive with half maximal inhibitory concentration (IC_50_) values greater than 100 μM. This result demonstrated that even small changes to the phenstatin scaffold were unfavourable for antiproliferative activity. From the initial screening results, it was concluded that the 1,2,4-triazole heterocycle alone was not sufficient to improve activity in the benzhydryl compounds compared to phenstatin. 

The IC_50_ value for the most potent triazole–phenstatin hybrid compound **19e** was determined in MCF-7 as 0.42 ± 0.07 μM at 72 h (Table 2). **19e** is a hybrid of phenstatin with the 3,4,5-trimethoxyaryl motif (ring A) and the 3-hydroxy-4-methoxyaryl B ring, but it is also related to the aromatase inhibitor letrozole due to the 1,2,4-triazole heterocycle. The hybrid structure suggests a potential for dual tubulin/aromatase activity, and therefore, this compound was selected for aromatase inhibition assay. 

Compound **24** is the only example synthesised containing the 1,2,3-triazole heterocycle and is also a direct analogue of phenstatin because of the presence of the 3,4,5-trimethoxyaryl motif (ring A) and the 3-hydroxy-4-methoxyaryl B ring. This structure showed excellent activity in MCF-7 cells with 27% cell viability at 1 μM and the IC_50_ value for the compound was determined as 52 nM (Table 2), which compares with CA-4 (IC_50_ = 3.9 nM) [102,103]. **24** was selected for further studies on different cell lines and for cell cycle analysis. Phenstatin (**7a**) was synthesised in our laboratory for use as a positive control (IC_50_ = 1.61 ± 2.7 nM) [104]. 

#### 3.1.3. Series 3: 1-(Diarylmethyl)-1*H*-Imidazoles **20b–h**,**k**,**l**

The results obtained from the preliminary screening of the benzhydryl imidazole derivatives, **20b–k** and **l** are shown in Figure 5A. These compounds carry a single substituent at the *para* position on one or both aryl rings (Cl, F, Br, OH, OCH_3_, CH_3_, etc). This library of compounds did not show any significant activity, with cell viability of 67–90% at concentrations of 1 and 0.1 μM, as observed for the Series 1 1,2,4-triazole derivatives **13b**–**g** and **l–o**, indicating that the imidazole ring alone is not sufficient for antiproliferative activity. The most active compounds in this panel were identified as the 4-nitro derivative **20b** and the 4-fluoro substituted compound **20d** (73% and 67% cell viability respectively at 1 μM).

#### 3.1.4. Series 4: 1-(Aryl-(3,4,5-Trimethoxyphenyl)Methyl)-1H-Imidazoles **21a-g**, **i-l**

The results obtained from the preliminary screening of the panel of phenstatin hybrid compounds carrying imidazole as the heterocyclic ring (**21a–g**, **i–l**) in MCF-7 cells are shown in Figure 5B. From the library of 3,4,5-trimethoxydiphenylmethyl-1*H*-imidazole derivatives (**21a–g**, **i–l**), compound **21l** was significantly the most active (31% viable cells at 1 μM), confirming the observation that the phenstatin scaffold is required for optimum activity. The remaining compounds in the series demonstrated weak activity, with viability >80% at 1 μM. The IC_50_ value of the most potent imidazole containing compound **21l** was determined as 0.132 ± 0.007 μM in MCF-7 cells (Table 2), and this compound was selected for further evaluations in other cancer cell lines and cell cycle analysis in MCF-7 cells. 

#### 3.1.5. Series 5: 1-(Diarylmethyl)Pyrrolidines **25a–g** and 1-(Diarylmethyl)Piperidines **26a–c**, and 1-(Diarylmethyl)Piperazines **27a–g**, **28**

The results of preliminary evaluation of the panel of pyrrolidine and piperidine derivatives **25a–g** and **26a–c** in MCF-7 cells are shown Figure 6A. These compounds were not sufficiently active when compared to the positive controls CA-4 and phenstatin (**7a**). The most potent examples were identified as the piperidine derivative **26b**, showing the lowest percentage of viable cells (78%) at 1 μM and containing the 3,4,5-trimethoxyphenyl (ring A) and 4-methoxyphenyl (Ring B), together with the corresponding pyrrolidine containing compounds **25g** and **25d** (82% and 80% viability at 1 μM). The (3,4,5-trimethoxyphenyl)(methyl)piperazine derivatives (**27c**,**d**,**f**,**h**,**i**) were screened at three concentrations (10, 1, 0.1 μM) (Figure 6B). Compound **27f** was identified as the most active, with a percentage of viable cells of 42% at 10 μM, 76% at 1 μM and 84% at 0.1 μM. Benzylpiperazine **27i**, which is more closely related in structure to phenstatin, displayed promising antiproliferative activity at 10 μM (48% cell viability).

From the results obtained above, it is interesting to see that inclusion of the triazole heterocycle on the phenstatin scaffold (as in compounds **21l** and **24**) results in greater antiproliferative effects in the MCF-7 cell line than the corresponding imidazole compound (**19e**). By comparison, replacement of the azole with pyrrolidine, piperidine, or piperazine resulted in decreased antiproliferative activity. The antiproliferative activity of the most potent azole compounds **19e**, **21l**, and **24** may be correlated to the logP values (see Supplementary Information). The imidazole compound **19e** has a lower logP (2.41) when compared to the 1,2,4-triazole compound **21l** (logP of 2.91) and the 1,2,3-triazole compound **24** (logP 3.50); the antiproliferative activity of the compounds **19e, 21l**, and **24** in MCF-7 cells are determined as IC_50_ = 0.42, 0.13, and 0.052 μM, respectively. In addition, the total polar surface (TPSA) area for these compounds is in the range 74.22–87.86 Å^2^ < 140 Å^2^. However, compounds with higher logP values e.g., the piperazine compounds **27f** (5.50) and **27d** (4.67) display poor activity. 

### 3.2. Antiproliferative Activity of Selected Analogues in MDA-MB-231 and HL60 Cell Lines

A number of the more potent compounds synthesised were evaluated in the triple-negative MDA-MB-231 cell line with 72 h incubation time (see Table 2). For the triazole compound **19e**, an IC_50_ value of 0.98 μM was obtained in MDA-MB-231 cells, although this is not as potent as observed in the MCF-7 cells (IC_50_ = 0.42 μM, Table 2). The lower IC_50_ value for the imidazole compound **21l** (0.237 μM) indicates that the imidazole heterocycle in **21l** contributes to the antiproliferative activity more effectively than the 1,2,4-triazole ring in compound **19e**. The novel 1,2,3-triazole compound **24** was the best of all analogues tested in MCF-7 cells (IC_50_ = 0.052 μM). The result obtained for **24** in the MDA-MB-231 cell line was also very promising (IC_50_ = 0.074 μM), Table 2, and compares very favourably with the reported activity of phenstatin in MDA-MB-231 cells (IC_50_ = 1.5 μM [105]), indicating that the 1,2,3-triazole has very potent antiproliferative effects compared to imidazole or 1,2,4-triazole present in the related compounds **21l** and **19e**. Since the antiproliferative effects of 1,2,3-triazole–phenstatin hybrid compounds has not previously been investigated, this heterocycle is especially interesting for further development. 

In a further study, the antiproliferative effects of the novel imidazole compound **21l,** 1,3,4-triazole compound **19e**, and the 1,2,3-triazole compound **24** (structurally related to letrozole and phenstatin) in HL-60 leukaemia cells was also investigated. HL-60 leukaemia cells were used as an in vitro model for acute myeloid leukaemia. Both MCF-7 and HL-60 cell lines are CA-4 sensitive are highly susceptible to the effects of tubulin-targeting compounds [102]. The IC_50_ value of 0.156 μM obtained for imidazole compound **21l** identifies it as a lead compound for future development. The 1,2,3-triazole compound **24** was also potent in the leukaemia HL-60 cell line with an IC_50_ value of 0.173 μM, while **19e** was less potent, IC_50_ = 261 μM. (IC_50_ value for phenstatin = 0.031 μM [106]). This experiment demonstrated the selective effect of interchanging the imidazole, 1,3,4-triazole, and 1,2,3-triazole heterocycles on cell viability in HL-60 cells.

### 3.3. NCI Cell Line Screening for **19e**, **21l**, **25g**, **26b**, and **27d**

Five novel substituted phenstatin compounds from the present work ((**19e**, (Series 2) **21l**, (Series 4), **25g**, **26b** and **27d** (Series 5)) were selected for evaluation in the NCI 60 cell line screen [107] following initial analysis of the Lipinski (drug-like) properties from the Tier-1 profiling screen, together with predictions of the relevant absorption, distribution, metabolism, excretion, and toxicity (ADMET) properties e.g., metabolic stability, permeability, blood–brain barrier partition, plasma protein binding, and human intestinal absorption properties (see Tables S1 and S2 Supplementary Information). The compounds are predicted to be moderately lipophilic–hydrophilic, revealing their drug-like pharmacokinetic profiles and are potentially suitable candidates for further investigation. 

The results obtained for the triazole compound **19e** in the NCI 60 cancer cell line screening (GI_50_ values, five doses) [107] are shown in Table 3. (GI_50_ is defined as the concentration for 50% of maximal inhibition of cell proliferation). In general, **19e** showed good activity on most of the cell lines with GI_50_ values in the sub-micromolar range. The activity was particularly potent in all of the leukaemia, CNS, and prostate cancer cell lines. The activity in MCF-7 cells (GI_50_ = 0.347 μM) was in close agreement with the value obtained from our in-house viability assay of 0.424 μM. The compound displayed significant activity in the TNBC cell lines HS-578T (GI_50_ = 0.548 μM) and MDA-MB-468 (GI_50_ = 0.371 μM) and in the BT-549 invasive ductal carcinoma cell line (GI_50_ = 0.618 μM). Potent anti-cancer activity was also observed against the ovarian cancer cells, e.g., OVOCAR-3 cell line (0.323 μM) and colon cancer, e.g., chemoresistant HT-29 cells (GI_50_ = 0.330 μM). The best activity for **19e** among all of the 60 cell lines tested was the melanoma cell line MDA-MB-435 in which the GI_50_ value was 0.181 μM. The MID GI_50_ was calculated as 0.243 μM over all 60 cell lines. The MID value obtained for TGI (total growth inhibition) was 53.7 μM, and for LC_50_, it was 97.7 μM, indicating that the lethal concentration of the drug is very high and well above the GI_50_ value, indicating that **19e** has low toxicity. The results of the NCI COMPARE analysis for compound **19e** are shown in Table 4. Based on the GI_50_ mean graph and on the TGI mean graph, the compound with the highest rank was vinblastine sulphate with *r* values of 0.586 and 0.737, respectively. Correlation values (*r*) are Pearson correlation coefficients. 

The National Cancer Institute (NCI) screening of imidazole compound **21l** also demonstrated very good results showing that the compound not only is active against breast cancer cells but also against other types of cancer (see Table 2). Compound **21l** proved active against all of the leukaemia cell lines; in particular, very promising activity was measured in SR cells (GI_50_ = 0.182 μM) and HL60 (GI_50_ = 0.229 μM), confirming our in-house evaluation. The activity against CNS cancer varied in a range between GI_50_= 0.192 and 0.731 μM. Particularly good was also the activity against the breast cancer panel with GI_50_ values in the range of 0.306–0.664 μM, including the TNBC cell line MDA-MB-468 (GI_50_ = 0.316 μM). Of all the cell lines evaluated in the panel, compound **21l** was most potent against melanoma MDA-MB-435 cells with GI_50_ = 0.119 μM. The MID GI_50_ value for the 60 cell line panel was 0.234 μM. MID TGI and LC_50_ values of 40.7 and 100 μM respectively are an indication of the low toxicity of the compound, as the median lethal dose is very high compared to the GI_50_ values. From the COMPARE analysis results shown in Table 3, it was observed that based on the mean GI_50_ value, the activity of our **21l** is most closely related to paclitaxel (*r* = 0.587). Based on TGI values, the compound with the highest ranking was maytansine (*r* = 0.775); both are tubulin-targeting agents. Correlation values (*r*) are Pearson correlation coefficients and LC_50_ values all >0.1 mM.

Compounds **25g**, **26b**, and **27d** were also selected for evaluation in the NCI 60 cell line one-dose screen (see Table S4, Supplementary Information). The mean growth percentages for **25g**, **26b**, and **27d** were 73.1%, 34.2%, and 65.5% over the 60 cell line panel at 10 μM. Interestingly, the piperidine compound **26b** displayed significant potency in the breast cancer panel, with mean growth of 30.3% over this cell panel, with notable potency in the triple negative breast cancer cell lines HS587T (16.6% growth) and MDA-MB-468 (9.3% growth). In the leukaemia panel, the mean growth obtained is 23.5% over this 60 cell panel and significantly 4.36% growth for the acute myeloid leukaemia HL-60 cell line. Compound **26b** also displayed notable potency in the CA-4 resistant colon cancer cell line HT-29 with 7.93% growth recorded. 

### 3.4. Evaluation of Toxicity in MCF-10A Cells

**Figure 7:**
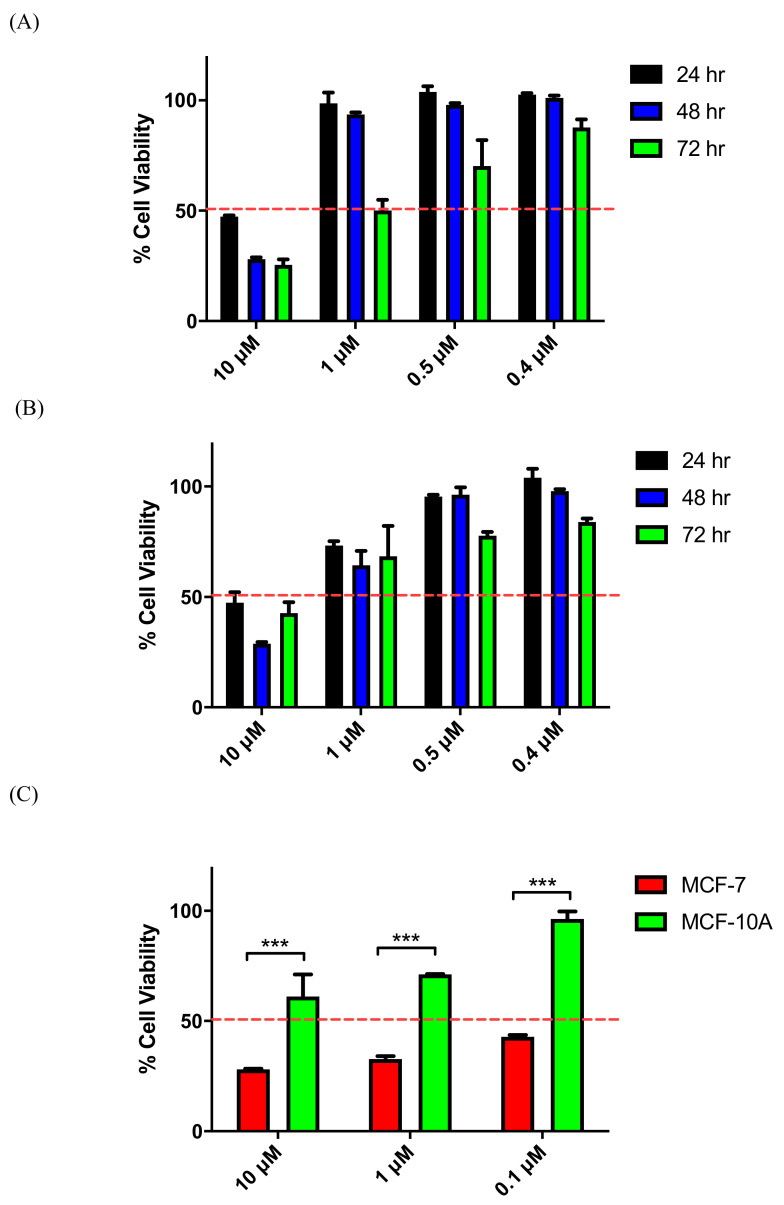
Screening of phenstatin derivatives **19e** (**A**) and **21l** (**B**) in MCF-10A cells at 24, 48, 72 h and (**C**) **24** in MCF-10A and MCF-7 cells at 72 h. (**A**,**B**) Effect of compounds **19e** and **21l** on the viability of non-tumorigenic MCF-10A human mammary epithelial cells. Cells were treated with the indicated concentrations for 21, 48, or 72 h. Cell viability was expressed as a percentage of vehicle control (ethanol 1% (*v*/*v*)) and was determined by alamarBlue assay (average + SEM of three independent experiments). (**C**) Effect of compound **24** on the viability of non-tumorigenic MCF-10A human mammary epithelial cells and MCF-7 breast cancer cells. Cells were treated with the indicated concentrations for 72 h. Cell viability was expressed as a percentage of vehicle control (ethanol 1% (*v*/*v*)) and was determined by alamarBlue assay (average ± SEM of three independent experiments). Statistical analysis was performed using two-way ANOVA (***, *p* < 0.001).

The potent phenstatin derivatives **19e**, **21l**, and **24** were selected for toxicity evaluation in the non-tumorigenic MCF-10A epithelial breast cancer cell line. The human mammary epithelial cell line MCF10A is widely used as an in vitro model for normal breast cell function and transformation [108]. The viability of the MCF-10A cells was determined after treatment with compounds **19e** and **21l** at four different concentrations of 10, 1, 0.5, and 0.4 μM for 24 h (Figure 7A,B). It was observed that at the highest concentration (10 μM), compounds **19e** and **21l** show a cell death of approximately 50%. At 1 μM concentration, compound **19e** does not show any loss in cell viability (99% viability), while compound **21l** resulted in 73% cell viability, still above the IC_50_ values of 0.42 μM (**19e**) and 0.13 μM (**21l**) in MCF-7 cells. When the experiment was repeated with an increased incubation time of 48 h, it was observed that the percentage of viable cells at 10 μM concentration decreased for compounds **19e** and **21l** to approximately 30% (Figure 7A,B). The percentage of viable cells at 1 μM decreased to 64% for compound **21l**, while it did not change significantly for **19e** (>94%). For both compounds, viability at 0.5 μM and 0.4 μM is close to 100%, which means that the compounds are not toxic toward healthy cells at lower concentrations corresponding to their IC_50_ values. The third screening for **19e** and **21l** was performed at 72 h, which is the incubation time used through all the screenings in MCF-7 (Figure 7A,B). It is interesting to note that as the concentration of the drug decreases from 1 to 0.5 and 0.4 μM, the percentage of viable cells increases significantly, with viable cells percentage >80% at 0.4 μM for all compounds tested. This demonstrates that even at concentrations that are toxic to the MCF-7 cancer cells, the MCF-10A are not killed by the drug. Therefore, the compounds selected demonstrate good antiproliferative activity and additionally show good selectivity and low cytotoxicity to normal cells. Compound **24** was evaluated in MCF-10A cells at three different concentrations: 10, 1, and 0.1 μM over 72 h (Figure 7C). The percentage of viable cells at the three different concentrations was 61%, 71%, and 96%, respectively, with higher percentage of cells alive at the lower concentration. Compound **24** demonstrates good selectivity for cancer cells and low cytotoxicity even if the percentage of viable cells at 1 μM was slightly lower than the value observed previously for compounds **19e** and **21l** (>80%) at 72 h. These results are also supported by the low toxicity of the compounds determined from the NCI evaluation. Tubulin-targeting drugs such as taxanes and vinca alkaloids are among the most effective anti-cancer therapeutics in the treatment of castration-resistant prostate cancer and triple-negative breast cancer. However, their use is limited by toxicities including neutropenia and neurotoxicity; additionally, tumour cells can develop resistance to these drugs [109]. Our results demonstrate that azoles **19e**, **21l**, and **24** were less toxic to normal human breast cells than to breast cancer cells, providing a potential window of selectivity. 

### 3.5. Effects of Compounds **21l** and **24** on Cell Cycle Arrest and Apoptosis 

**Figure 8:**
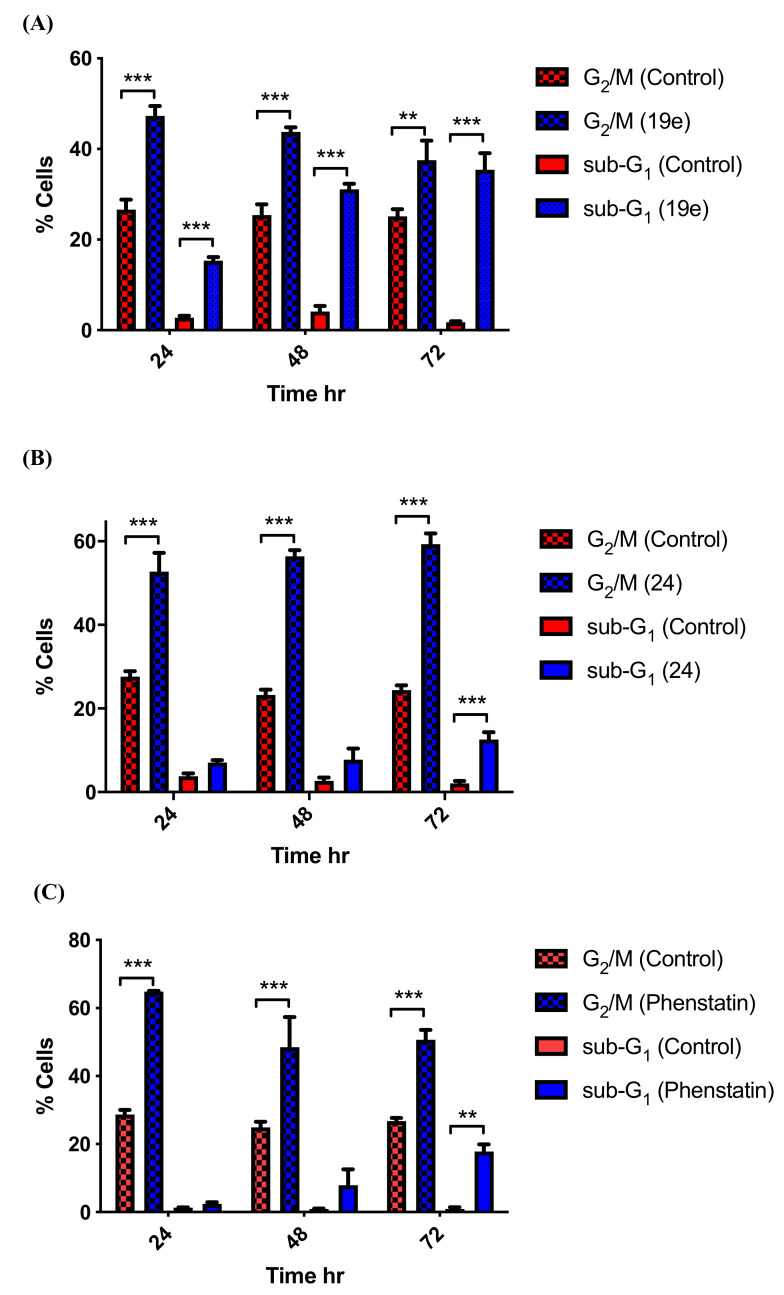
Compound (**A**) **19e**, (**B**) **24**, and (**C**) phenstatin (**7a**) in MCF-7 induced G2/M arrest followed by apoptosis in a time-dependent manner in MCF-7 cells. Cells were treated with either vehicle control (v) (0.1% ethanol (*v*/*v*)] or compound **19e**, **24**, or phenstatin (**7a**) (1 μM) for 24, 48, and 72 h). Then, cells were fixed, stained with PI, and analysed by flow cytometry. Cell cycle analysis was performed on histograms of gated counts per DNA area (FL2-A). The number of cells with <2 N (sub-G1), 2 N (G0G1), and 4 N (G2/M) DNA content was determined with CellQuest software. Values represent as the mean ± SEM for three separate experiments. Statistical analysis was performed using two-way ANOVA (**, *p* < 0.01, ***, *p* < 0.001).

To investigate further the mechanism of action of the novel azole compounds synthesised, the effect of selected potent compounds **21l** and **24** was investigated in MCF-7 cells by flow cytometry and propidium iodide (PI) staining, allowing the percentage of cells in each phase of the cell cycle to be quantified (Figure 8). For the imidazole compound **21l**, three time points were analysed (24, 48, and 72 h), and the values obtained for apoptosis and the G_2_/M phase of the cell cycle were quantified (concentration 1 μM), as shown in Figure 8A. It was observed that the percentage of cells undergoing apoptosis (sub-G_1_) increases significantly at all three time points to 15%, 31%, and 37% respectively compared to the background level of apoptosis with the vehicle ethanol (2%, 4%, and 2%) at the corresponding time points. It is also interesting to notice how the percentage of cells in the G_2_/M phase for the treated sample (47%, 43%, and 40%) is statistically higher than the cells in the same phase for the control sample treated with the vehicle (26%, 25%, 25%) at the corresponding time points. G_2_/M cell cycle arrest is strongly associated with an inhibition of tubulin polymerisation. CA-4 and related tubulin targeting compounds cause G2/M arrest. Hence, the higher percentage of cells observed in cells treated with **21l** may suggest that the mechanism of action is indeed the inhibition of tubulin polymerisation.

The 1,2,3-triazole compound **24**, which was the most potent compound evaluated in the viability assay, demonstrated the same effects on the relative percentages of cells in apoptosis and the G_2_/M phase, as shown in Figure 8B. Apoptosis increased with time, with a statistically significant difference compared to vehicle control at 72 h. A high percentage of cells were arrested in the G_2_/M phase (52%, 56%, and 59%) at time points 24, 48, and 72 h respectively, following treatment with compound **24** with a much lower percentage of cells in the G_2_/M phase for the sample treated with the vehicle (28%, 23%, and 24%) at the same time points.

Phenstatin **7a** was used as a positive control through all the biological experiments. Cell cycle analysis of MCF-7 cells treated with phenstatin at time points 24, 48, and 72 h and a concentration of 1 μM showed a very low percentage of cells undergoing apoptosis at 24 and 48 h, as shown in Figure 8C. Apoptosis increased to 18% between 48 and 72 h, while the percentage of cells in the G_2_/M phase was correspondingly high (65%, 49%, and 51% at 24, 48, and 72 h, respectively). This pattern was also observed in the compounds **21l** and **24** tested, but the percentage of cells in apoptosis was always higher than for phenstatin, possibly suggesting differences in the effects of these compounds on tubulin arising from the presence of the azole in the modified structures.

**Figure 9:**
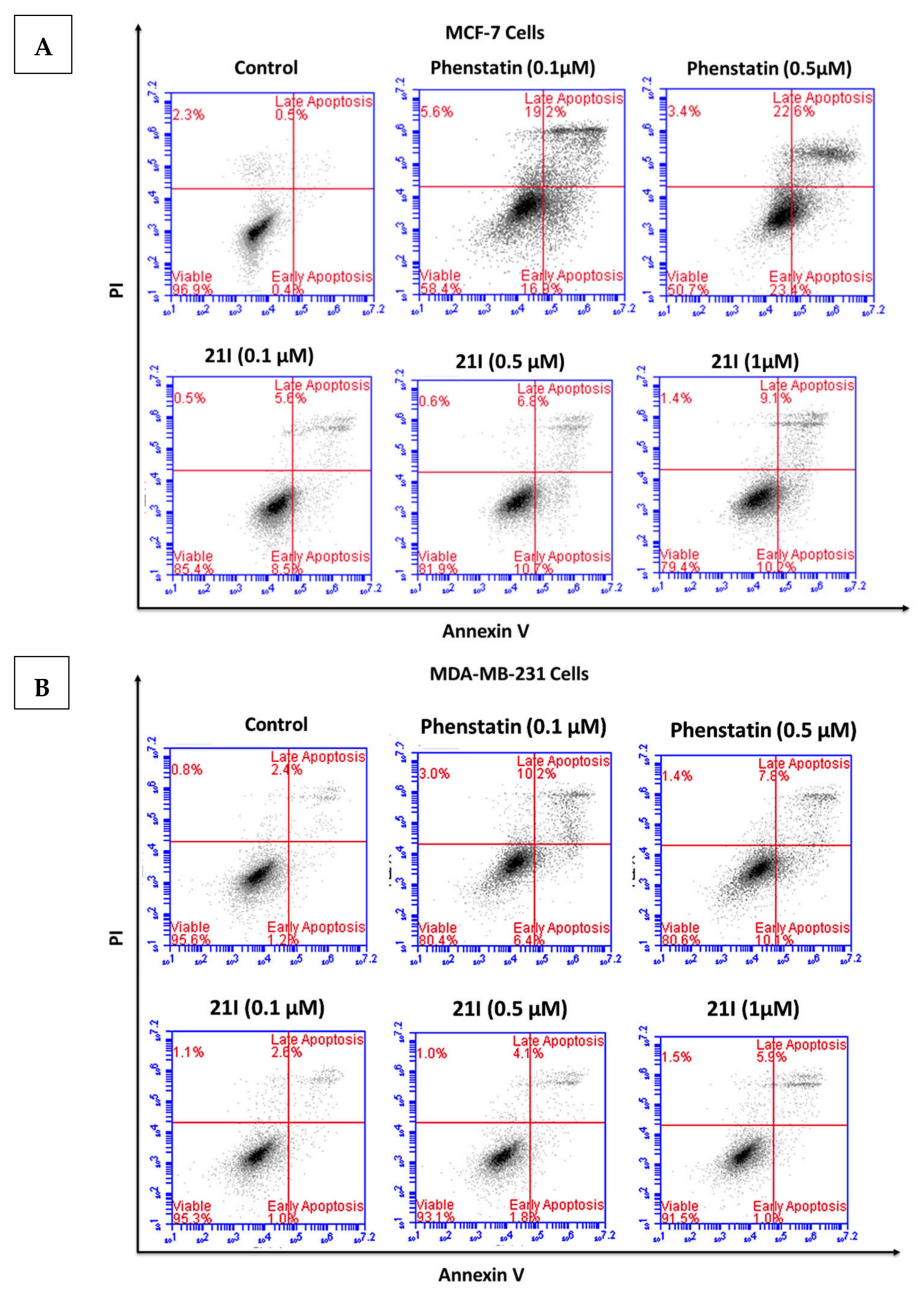
Compound **21l** induced cell apoptosis in (**A**) MCF-7 breast cancer cells and (**B**) MDA-MB-231 breast cancer cells. MCF-7 breast cancer cells and MDA-MB-231 breast cancer cells were treated with **21l** or phenstatin (**7a**) (1 µM) or control vehicle (0.1% ethanol (*v*/*v*)), and the percentage of apoptotic cells was determined by staining with Annexin V-FITC and PI. In each panel, the lower left quadrant shows cells that are negative for both PI and Annexin V-FITC, the upper left shows only PI cells that are necrotic. The lower right quadrant shows Annexin-positive cells that are in the early apoptotic stage and the upper right shows both Annexin/PI positive, which are in late apoptosis/necrosis. Control cells and cells treated with phenstatin **7a** and **21l** at 48 h are shown, respectively. Values represent the mean of three independent experiments.

The role of apoptosis in the inhibition of MCF-7 and MDA-MB-231 cell growth was further examined. MCF-7 and MDA-MB-231cells were treated with compound **21l** for 48 h and stained with Annexin V-fluorescein isothiocyante (FITC)/propidium iodide (PI) and then analysed by flow cytometry. Dual staining with Annexin-V and PI facilitates discrimination between live cells (annexin-V^−^/PI^−^), early apoptotic cells (annexin- V^+^/PI^−^), late apoptotic cells (annexin-V^+^/PI^+^), and necrotic cells (annexin-V^−^/PI^+^). Compound **21l** induced both early and late apoptosis in MCF-7 cells in a concentration-dependent manner when compared to the untreated control cells (Figure 9A). When MCF- 7 cells were treated with **21l** (0.1, 0.5, and 1 μM), the average proportion of Annexin V-stained positive cells (total apoptotic cells) increased from 0.9% in control cells to 14.1%, 17.5%, and 19.3%, respectively. These results suggested that compound **21l** induces the apoptosis of MCF-7 cells in a dose-dependent manner. In MDA-MB-231 cells, the percentage of cells observed in apoptosis following treatment with **21l** was significantly lower with 3.6%, 5.9%, and 6.9% at 0.1, 0.5, and 1.0 μM respectively, as shown in Figure 9B. In contrast, for phenstatin, the Annexin V-stained positive cells (total apoptotic) cells were determined as 36.1% and 46% in MCF-7 cells at 0.1 μM and 0.5 μM, respectively, as shown in Figure 9A. The total apoptotic MDA-MB-231 cells were determined as 16.6% and 17.9% following treatment with phenstatin (0.1 and 0.5 μM), respectively, as shown in Figure 9B. 

### 3.6. Tubulin Polymerisation 

**Figure 10:**
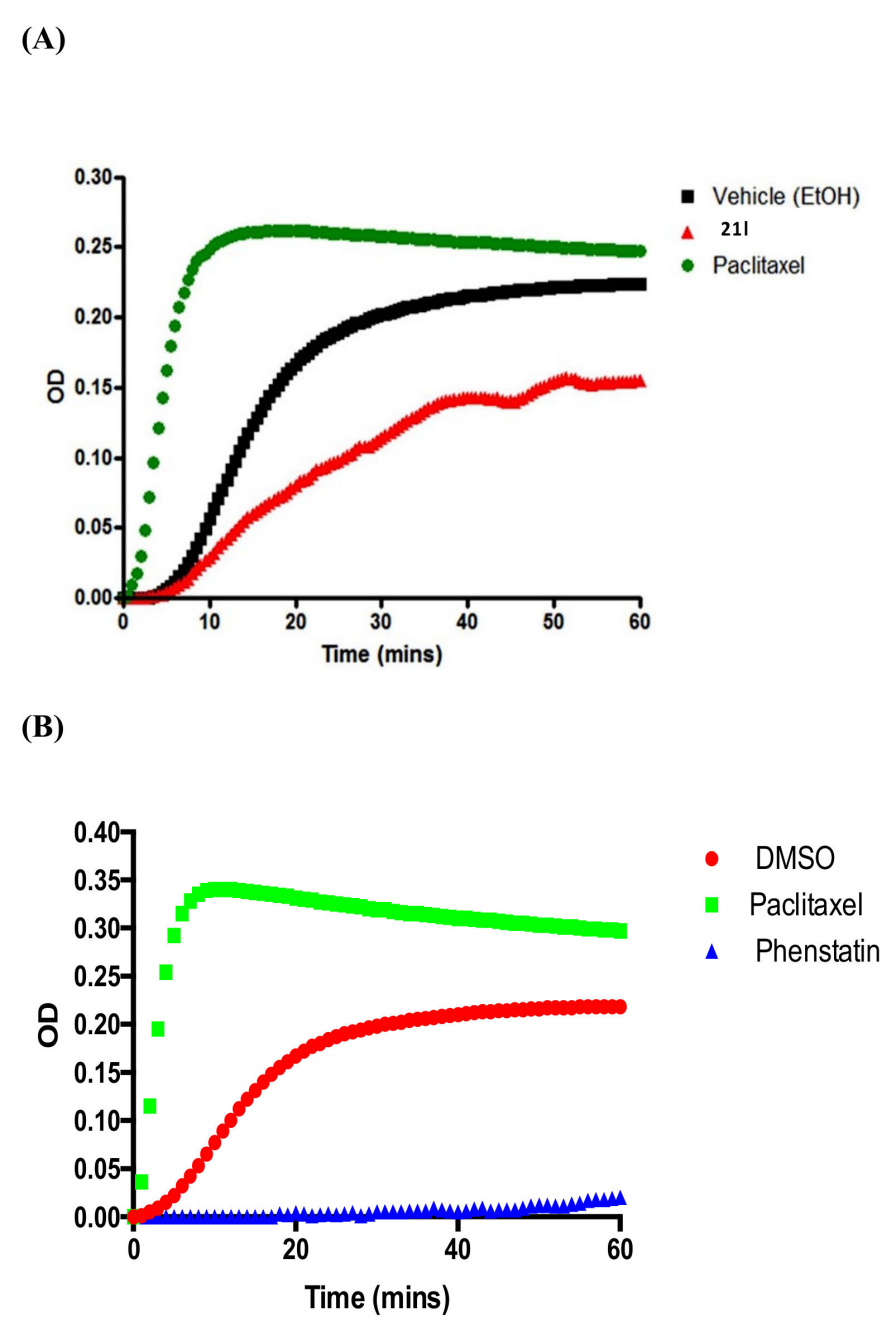
Effect of compound **21l** on tubulin polymerisation in vitro. (**A**) Tubulin polymerisation assay for compound **21l** at 10 μM. (**B**) Paclitaxel (10 μM) and phenstatin (**7a**) (10 μM) were used as references while ethanol (1% *v*/*v*) and DMSO (1% *v*/*v*) were used as vehicle controls. Purified bovine tubulin and guanosine-5’-triphosphate (GTP) were mixed in a 96-well plate. The polymerisation reaction was initiated by warming the solution from 4 to 37 °C. The effect on tubulin assembly was monitored in a Spectramax 340PC spectrophotometer at 340 nm at 30 s intervals for 60 min at 37 °C. DMSO. Fold inhibition of tubulin polymerisation was calculated using the V_max_ value for each reaction. The results represent the mean for three separate experiments.

Compound **21l** was selected for further analysis using a tubulin polymerisation assay. Its promising antiproliferative activity (IC_50_ = 0.237 μM in MCF-7 cells) combined with structural features related to phenstatin **7a** and CA-4 indicate that the mechanism of action of this compound could be the inhibition of tubulin polymerisation. The assay is based on the capacity of microtubules to scatter light proportionally to their concentration. The imidazole compound **21l** (red) showed good inhibition of tubulin polymerisation after 60 min (V_max_ value 2.84 ± 0.10 mOD/min at 10 μM), corresponding to a 1.34-fold reduction of the polymer mass compared to the vehicle, as shown in Figure 10A. Paclitaxel (in green) was used as a positive control as it stabilises polymerised tubulin, as shown in Figure 10A. Phenstatin **7a** is a potent inhibitor of tubulin polymerisation comparable to CA-4 [66], as shown in Figure 10B. Incubation with either imidazole **21l** or phenstatin resulted in a significant inhibition of tubulin polymerisation and assembly. 

**Figure 11:**
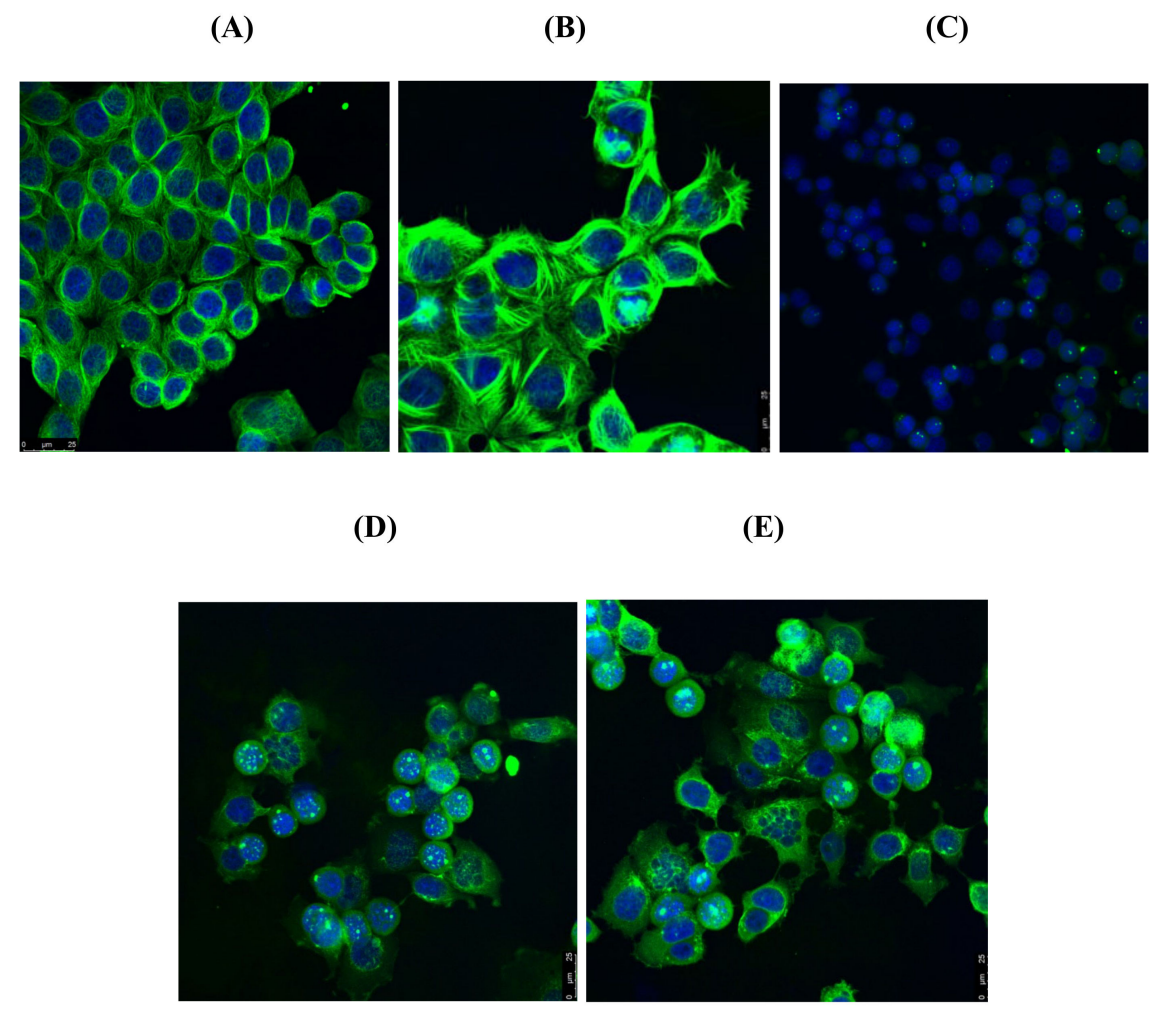
Compounds **19e** and **21l** depolymerise the microtubule network of MCF-7 breast cancer cells. Cells were treated with (**A**) vehicle control (0.1% ethanol (*v*/*v*)), (**B**) paclitaxel (1 μM), (**C**) phenstatin (**7a**) (1 μM), (**D**) compound **19e** (10 μM), or (**E**) compound **21l** (10 μM) for 16 h. Cells were fixed in ice-cold methanol and stained with mouse monoclonal anti-α-tubulin–fluorescein isothiocyante (FITC) antibody (clone DM1A) (green), Alexa Fluor 488 dye, and counterstained with DAPI 4’-6’-diamidino-2-phenylindole (blue). Images obtained with Leica SP8 confocal microscopy, Leica application suite X software. Representative confocal micrographs of three separate experiments are shown. Scale bar indicates 25 μm.

Following the experiment above, the in vitro effects of compounds **19e** and **21**l were examined on the microtubule structure of MCF-7 breast cancer cells with confocal microscopy using anti-tubulin antibodies. Paclitaxel and phenstatin, a known polymeriser and depolymeriser of tubulin respectively, were used as controls. In Figure 11A, a well-organised microtubule network (stained green) is clearly seen for the vehicle control, together with the MCF-7 cell nuclei (stained blue). Hyperpolymerisation of tubulin was demonstrated in the paclitaxel-treated sample (Figure 11B), whereas the phenstatin-treated sample Figure 11C shows an extensive depolymerisation of tubulin. Cells treated with the azoles **19e** (Figure 11D) and **21l** (Figure 11E) displayed disorganised microtubule networks with similar effects to phenstatin, together with multinucleation (formation of multiple micronuclei), which is a recognised sign of mitotic catastrophe [110] previously observed by us and others upon treatment with tubulin-targeting agents such as CA-4 and related compounds in non-small cell lung cancer cells and breast cancer MCF-7 cells [111,112]. 

### 3.7. Effects of Compounds **21l** and **24** on Expression Levels of Apoptosis-Associated Proteins 

Some of the novel compounds synthesised during the project were selected for further investigation of their mechanism of action as pro-apoptotic agents based on their effect on the expression of proteins that can regulate apoptosis or proteins involved in the regulation of DNA repair. The effects of compounds **21l** and **24** on apoptosis were evaluated by Western blotting. Apoptosis regulating proteins Bcl-2 and Mcl-1 were investigated along with PARP. PARP (poly ADP-ribose polymerase) is involved in the repair of DNA single-strand breaks in response to environmental stress [113]; and PARP cleavage is considered a hallmark of apoptosis. Bcl-2 is an anti-apoptotic protein that prevents apoptosis by sequestering caspases (apoptosis promoters) or by preventing the release of pro-apoptotic cytochrome c and AIF (apoptosis inducing factor) from the mitochondria into the cytoplasm [114]. The Mcl-1 protein belongs to the Bcl-2 family; it is also an anti-apoptotic protein localised in the mitochondrial outer membrane that acts at a very early stage in the cascade, leading to the release of the cytochrome c [115]. Pro- and anti-apoptotic members of the Bcl-2 family can heterodimerise and titrate each other’s functions. If the expression levels of Mcl-1 and Bcl-2 are reduced (by drug treatment), apoptosis may be triggered. 

**Figure 12:**
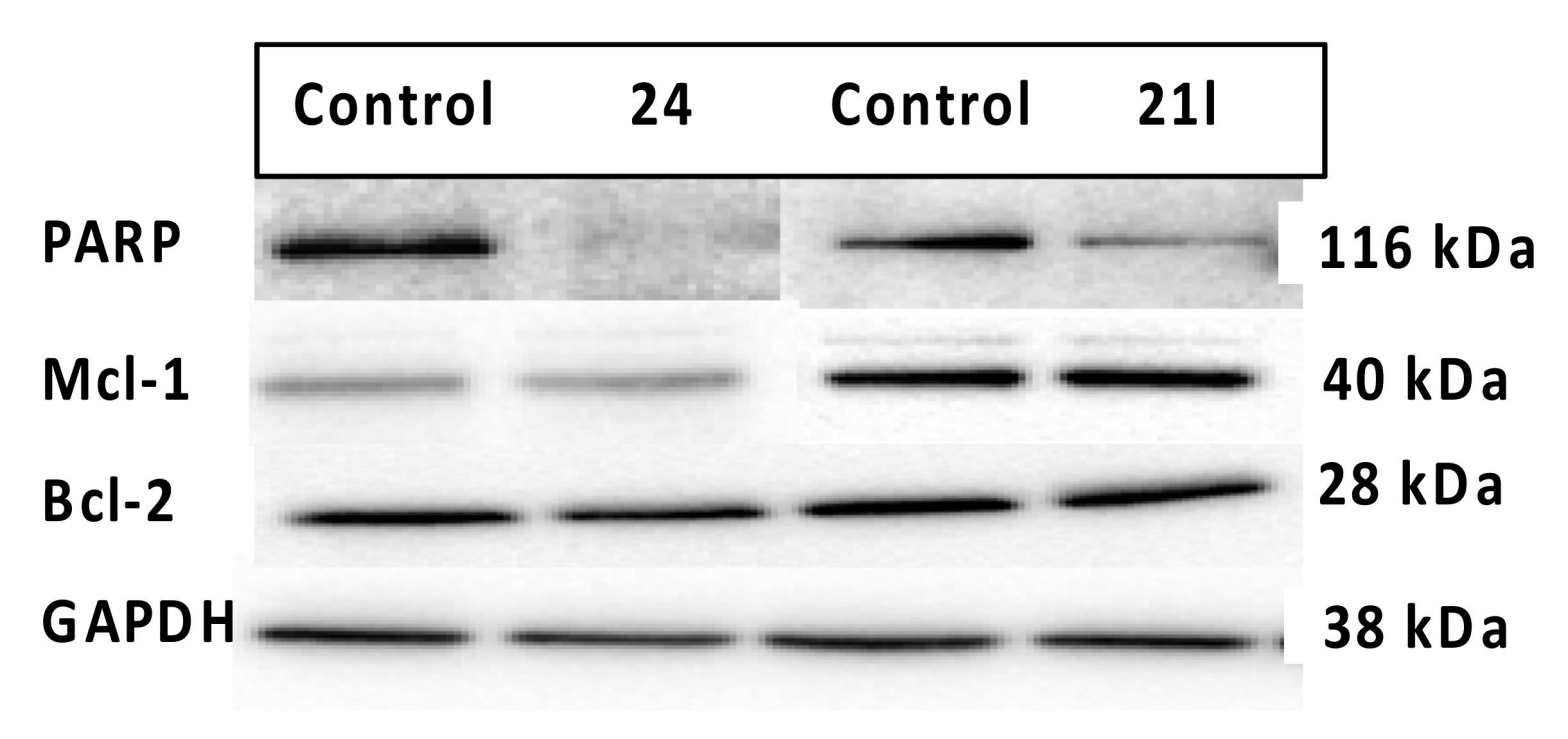
Effects of **21l** and **24** on expression of PARP (poly ADP-ribose polymerase) and anti-apoptotic proteins Bcl-2 and Mcl-1. MCF-7 cells were treated with either vehicle control (ethanol, 0.1% *v*/*v*) or with compounds **21l** and **24** (1 μM) for 24 h. After the required time, cells were harvested and separated by SDS-PAGE to detect the level of the apoptosis-related proteins. The membrane was probed with anti-PARP or anti-cleaved PARP antibodies. Results are representative of three separate experiments. To confirm equal protein loading, each membrane was stripped and re-probed with glyceraldehyde 3-phosphate dehydrogenase (GAPDH) antibody.

From the results obtained, no change in the expression levels of two anti-apoptotic proteins was observed, indicating that Bcl-2 and Mcl-1 may not play a critical role in the pro-apoptotic mechanism of action of the compounds (Figure 12). A significant reduction in the expression of full-length PARP (116 kDa) between the vehicle and the treated MCF-7 cells was observed (Figure 12), suggesting that **21l** and **24** cause PARP cleavage. PARP enzymes play a crucial role in the DNA repair, and PARP cleavage is affected by caspase 3 activity. PARP enzymes are found in the cell nucleus and are activated by damage of the DNA single strand; therefore, the inhibition of DNA repair in cancer cells represents an attractive strategy in cancer therapy [116]. In conclusion, the proposed mechanism of action of these compounds as pro-apoptotic drugs is supported by the observed increase in the percentage of cell in subG1 in the cell cycle profile, the flow cytometric analysis of Annexin V/PI-stained cells, and also by PARP cleavage. 

### 3.8. Aromatase Inhibition

An objective of this research was the design of dual-acting tubulin/aromatase inhibitors. The evaluation of the aromatase inhibitory activity of the most potent compounds prepared was next investigated. Three compounds of the phenstatin hybrid panel **21l**, **24** and **19e** were selected for evaluation against two cytochrome members of the P450 family: CYP19 and CYP1A1. CYP19 is the aromatase cytochrome directly responsible for the synthesis of estradiol by the aromatisation of its steroid precursors testosterone and androstenedione, while CYP1A1 is involved in the metabolism of estrogen. The specificity of aromatase inhibition was evaluated by an assay carried out with xenobiotic-metabolising cytochrome P450 enzymes CYP1A1. The methodology applied in this study requires the detection of the hydrolysed dibenzylfluorescein (DBF) by the aromatase enzyme [117]. Aromatase and CYP1A1 inhibition were quantified by measuring the fluorescent intensity of fluorescein, the hydrolysis product of dibenzylfluorescein (DBF), by aromatase as previously described [118,119]. Naringenin was used as a positive control, yielding IC_50_ values of 4.9 µM. The test was initially conducted at one concentration (20 µg/mL). Further experiments to determine the IC_50_ value were performed if the compound caused greater than 90% inhibition at 20 µg/mL. The results are presented in Table 5. Of these, 1,2,3-triazole **24** was inactive, as it did not show any inhibition of the enzyme at 20 µg/mL, 0.05 μM (0.01% for CYP19 and 12.81% for CYP1A1), whereas imidazole **21l** (0.05 μM) and 1,2,4-triazole **19e** (0.05 μM) were active in the first screen against CYP19 (Table 5). The inhibition for imidazole **21l,** although potent, was not concentration-dependent, and the IC_50_ could not be determined. 1,2,4-Triazole **19e** inhibited aromatase in a concentration-dependent manner, and its IC_50_ was determined as 29 µM. Of all the tested compounds (**21l**, **24**, and **19e**), none showed significant inhibition of CYP1A1, yielding IC_50_ values above 53 µM, which is regarded as inactive [119,120]. From the results obtained, we can suggest that the 1,2,4-triazole heterocycle is required for aromatase inhibition in the phenstatin related compound **19e**. Therefore, the 1,2,4-triazole compound **19e** could be identified as a potential dual-acting drug for the treatment of breast cancer targeting both aromatase inhibition and tubulin polymerisation.

### 3.9. Molecular Docking of Phenstatin Hybrids **19e**, **21l**, and **24**

**Figure 13:**
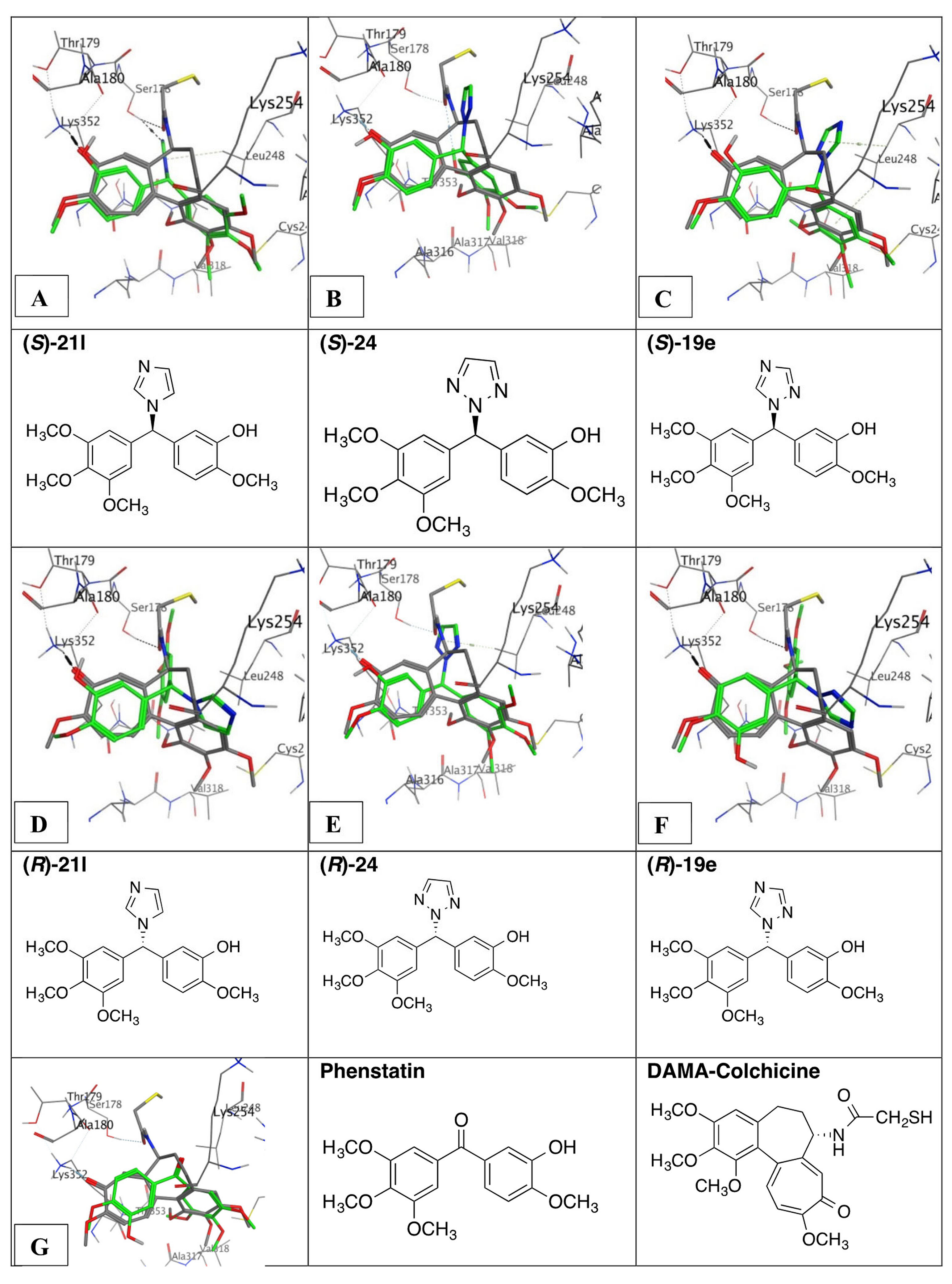
Docking of compounds **19e**, **21l**, and **24** in the colchicine binding site of tubulin. Overlay of the X-ray structure of tubulin co-crystallised with DAMA-colchicine (PDB entry 1SA0) on the best-ranked docked poses of the three *S* enantiomers (**A**) **21l,** (**B**) **24**, and (**C**) **19e**. Overlay of the X-ray structure of tubulin co-crystallised with DAMA-colchicine (PDB entry 1SA0) on the best ranked docked poses of the three *R* enantiomers (**D**) **21l**, (**E**) **24**, (**F**) **19e**, and (**G**) Phenstatin (**7a**). Ligands are rendered as tube and amino acids as a line. Tubulin amino acids and DAMA-colchicine are coloured by atom type; the three heterocycles are coloured green. The atoms are coloured by element type, carbon = grey, hydrogen = white, oxygen = red, nitrogen = blue, sulphur = yellow. Key amino acid residues are labelled, and multiple residues are hidden to enable a clearer view.

Compounds **19e**, **21l**, and **24** were next examined in tubulin molecular docking experiments to rationalise the observed biochemical activities. These three molecules contain a 3-hydroxy-4-methoxy substituted aromatic ring and a 3,4,5-trimethoxyphenyl ring and differ in the nitrogen heterocycle that is substituted on the benzyhdryl linkage. The compounds phenstatin **7a** and N-deacetyl-N-(2-mercaptoacetyl)colchicine (DAMA-colchicine) were used as reference compounds in the docking experiments. Since the compounds **19e**, **21l**, and **24** were synthesised as racemates, both R and S enantiomers of each compound were docked in the crystallised tubulin structure 1SA0 [121] and ranked based on the substituent and enantiomer giving the best binding results as illustrated in Figure 13. The co-crystallised tubulin DAMA–colchicine structure 1SA0 [121] was used for this study, as it has been demonstrated that both CA-4 **4a** and phenstatin **7a** interact at the colchicine-binding site of tubulin. Figure 13A–C shows the binding of the *S* enantiomers, the ranking for the binding of the three different compounds in order: ***S*-21l**, ***S*-24**, and ***S*-19e**. All three compounds demonstrate a strong interaction with the same amino acid residue Lys352. Compound ***S*-21l** forms a hydrogen bond acceptor interaction between an imidazole nitrogen and Ser178. The imidazole also forms a π–CH interaction with Leu248. Compounds ***S*-24** and ***S*-19e** show very similar behaviour; they do not bind Ser178 but still have the same interaction with Leu248. In the *R*-enantiomer series, the heterocycle is directed differently, and very different binding poses and less favourable binding interactions between the ligands and the tubulin binding site are predicted for these compounds (Figure 13D–F). In order to maintain the A and C-ring overlays, the heterocycle would clash with binding site amino acids, so for the three *R*-enantiomers, the heterocycle overlays with either the A or C-ring and the 3,4,5-trimethoxyphenyl mapping is no longer possible or not as ideal.

Compound ***S*-21l** was the highest ranked compound in the series; therefore, it would be of interest to obtain in vitro results for the enantiomerically pure compound. Phenstatin **7a** also maps well to the colchicine binding pose with the 3,4-5-trimethoxyaryl residues overlaying effectively and the B-ring 4-methoxy group positioned to form a hydrogen bond with Lys352 (Figure 12G). The results provide rationalisation of the observed biochemical experiments in which cell cycle and tubulin binding was confirmed, indicating that these compounds are apoptotic and tubulin depolymerising agents.

## 4. Materials and Methods 

### 4.1. Chemistry

All reagents were commercially available and were used without further purification unless otherwise indicated. Anhydrous solvents were purchased from Sigma. Uncorrected melting points were measured on a Gallenkamp apparatus. Infrared (IR) spectra were recorded on a Perkin Elmer FT-IR Paragon 1000 spectrometer. ^1^H and ^13^C nuclear magnetic resonance spectra (NMR) were recorded at 27 °C on a Brucker DPX 400 spectrometer (400.13 MHz, ^1^H; 100.61 MHz, ^13^C) in CDCl_3_ (internal standard tetramethylsilane (TMS)). For CDCl_3_, ^1^H NMR spectra were assigned relative to the TMS peak at 0.00 ppm, and ^13^C NMR spectra were assigned relative to the middle CDCl_3_ peak at 77.0 ppm. Electrospray ionisation mass spectrometry (ESI-MS) was performed in the positive ion mode on a liquid chromatography time-of-flight mass spectrometer (Micromass LCT, Waters Ltd., Manchester, UK). The samples were introduced to the ion source by an LC system (Waters Alliance 2795, Waters Corporation, Milford, MA, USA) in acetonitrile/water (60:40% *v*/*v*) at 200 µL/min. The capillary voltage of the mass spectrometer was at 3 kV. The sample cone (de-clustering) voltage was set at 40 V. For exact mass determination, the instrument was externally calibrated for the mass range m/z 100 to 1000. A lock (reference) mass (m/z 556.2771) was used. Mass measurement accuracies of < ±5 ppm were obtained. Thin-layer chromatography (TLC) was performed using Merck Silica gel 60 TLC aluminium sheets with fluorescent indicator visualising with UV light at 254 nm. Flash chromatography was carried out using standard silica gel 60 (230–400 mesh) obtained from Merck. All products isolated were homogenous on TLC. The purity of the tested compounds was determined by HPLC. Analytical high-performance liquid chromatography (HPLC) was performed using a Waters 2487 Dual Wavelength Absorbance detector, a Waters 1525 binary HPLC pump, and a Waters 717 plus Autosampler. The column used was a Varian Pursuit XRs C18 reverse phase 150 × 4.6 mm chromatography column. Samples were detected using a wavelength of 254 nm. All samples were analysed using acetonitrile (60%)/water (40%) over 10 min and a flow rate of 1 mL/min. Microwave experiments were carried using a Biotage Discover CEM microwave synthesiser on a standard power setting (maximum power supplied is 300 watts) unless otherwise stated. Details of the synthesis and characterisation of intermediate compounds and target azole products are available in the Supporting Information. 

#### 4.1.1. (3-Hydroxy-4-Methoxyphenyl)(3,4,5-Trimethoxyphenyl)Methanone, Phenstatin (**7a**)

2-Methoxy-5-(3,4,5-trimethoxybenzoyl)phenyl 2-chloroacetate (**23c**) (1 eq, 1.28 mmol, 0.51 g) was reacted with sodium acetate (4.5 eq, 5.76 mmol, 0.47 g) in methanol (10 mL) at reflux for 2 h. After cooling, the mixture was concentrated under reduced pressure. Distilled water was added to the residue, and the resulting precipitate was filtered and recrystallised from ethanol. Yield: 89% (0.361 g) white solid Mp: 152–156 °C [66,67]. ^1^H NMR (400 MHz, CDCl_3_) δ 7.42 (d, *J* = 2.1 Hz, 1H, Ar-H), 7.37 (dd, *J* = 8.3, 2.1 Hz, 1H, Ar-H), 7.01 (s, 2H, Ar-H), 6.90 (d, *J* = 8.4 Hz, 1H, Ar-H), 3.96 (s, 3H, OCH_3_), 3.91 (s, 3H, OCH_3_), 3.86 (s, 6H, OCH_3_). ^13^C NMR (101 MHz, CDCl_3_) δ 194.63 (C=O), 152.75 (2xC-O), 150.17 (C-O), 145.29 (C-OH), 141.61 (C-O), 133.13 (C), 131.03 (C), 123.61 (CH), 116.19 (CH), 109.66 (CH), 107.49 (2xCH), 60.93 (OCH_3_), 56.28 (2xOCH_3_), 56.07 (OCH_3_). LRMS (EI): found 319.37 (M+H)^+^; C_17_H_19_O_6_ requires 319.12. IR: ν_max_ (ATR) cm^−1^: 3249, 3003, 2840, 1632, 1578, 1505, 1443, 1414, 1331, 1236, 1222, 1118, 1002, 931, 892, 870, 758, 736, 670, 639, 576.

#### 4.1.2. General Method A: Preparation of Alcohols 

To a solution of the benzophenone in methanol (25 mL), NaBH_4_ (1 eq) was added in small portions. The solution was stirred at 0 °C until the reaction was complete from TLC. Dilute HCl (10%) was added, and the solvent was removed with the rotary evaporator. Then, the product was dissolved in ethyl acetate (30 mL) and washed with water (20 mL) and brine (10 mL), dried over sodium sulphate, filtered, and concentrated. Purification via flash column chromatography (eluent: *n*-hexane/ethyl acetate 1:1) afforded the product.

##### *N*-(4-(Hydroxy(Phenyl)Methyl)Phenyl)Acetamide (**12i**)

As per general method A, a solution of compound **11i** (1 eq, 0.5 mmol, 0.12 g) in methanol (25 mL) was treated with sodium borohydride (1 eq, 0.5 mmol, 0.02 g). The product was isolated without further purification, as an oil (0.1 g, 83%). IR: ν_max_ (ATR) cm^−1^: 3058, 1653, 1601, 1512, 1408, 1370, 1250, 1174, 1092, 1018. ^1^H NMR (400 MHz, CDCl_3_) δ 7.43 (s, 2H, Ar-H), 7.32 (s, 2H, Ar-H), 7.31 (d, *J* = 1.4 Hz, 2H, Ar-H), 7.29 (d, *J* = 2.4 Hz, 2H, Ar-H), 7.27 (s, 1H, Ar-H), 5.20 (s, 1H, CH-OH), 2.14 (s, 3H, CH_3_). ^13^C NMR (101 MHz, CDCl_3_) δ 168.28 (NH-C=O), 143.72 (C), 138.02 (C), 137.10 (C-NH), 128.36 (2xCH), 127.59 (2xCH), 127.44 (CH), 126.82 (2xCH), 119.80 (2xCH), 84.92 (CH-OH), 30.91 (CH_3_). LRMS (EI): C_15_H_16_NO_2_, 242.27 (M+H)^+^. 

##### 4-(Hydroxy(Phenyl)Methyl)Phenyl 2,2,2-Trifluoroacetate (**12j**)

As per general method A, a solution of compound **11j** (1 eq, 2.12 mmol, 0.622 g) was treated with sodium borohydride (1 eq, 2.12 mmol, 0.08 g). Following purification via flash column chromatography (eluent: *n*-hexane/ethyl acetate 1:1), the product was isolated as a white solid, 89% (0.562 g) Mp. 145–147 °C. IR: ν_max_ (ATR) cm^−1^: 3299, 1701, 1602, 1542, 1494, 1448, 1420, 1246, 1178, 1152, 1016. ^1^H NMR (400 MHz, CDCl_3_) δ 7.73–7.66 (m, 1H, Ar-H), 7.52 (d, *J* = 8.6 Hz, 2H, Ar-H), 7.41 (d, *J* = 8.6 Hz, 2H, Ar-H), 7.35–7.32 (m, 4H, Ar-H), 5.84 (s, 1H, CH-OH). ^13^C NMR (101 MHz, CDCl_3_) δ 163.43 (C=O), 143.40 (C-O), 141.94 (C), 132.90 (C), 128.62 (2xCH), 127.84 (CH), 127.47 (2xCH), 126.49 (2xCH), 120.45 (2xCH), 113.60 (CF_3_), 75.65 (CH-OH). HRMS (EI): found 294.0745 (M-2H)^+^; C_15_H_9_F_3_O_3_ requires 294.0504.

#### 4.1.3. General Method B: Preparation of 1-(Diarylmethyl)-1*H*-1,2,4-Triazoles

To a solution of the secondary alcohol (1 eq) in toluene (60 mL), in a round-bottom flask connected to a Dean-Stark trap was added 1,2,4-triazole (3 eq) and *p*-toluenesulfonic acid (200 mg, 0.61 eq). The source of heating used for the reaction was a Biotage open vessel microwave reactor (90–250 W). The reaction mixture was heated at reflux for 4 h, the toluene was evaporated, and the crude product was dissolved in ethyl acetate (30 mL) and washed with water (20 mL) and brine (10 mL). The solution was dried over sodium sulphate, filtered, and concentrated under reduced pressure. The crude product was purified via flash chromatography (*n*-hexane/ethyl acetate, 1:1) over silica gel to afford the desired product.

##### 1-((4-Fluorophenyl)(Phenyl)Methyl)-1*H*-1,2,4-Triazole (**13c**)

As per general method B, compound **12d** (1 eq, 2.47 mmol, 0.5 g) was reacted with 1,2,4-triazole and *p*-TSA in toluene. The crude product was purified via flash chromatography (eluent: *n*-hexane/ethyl acetate 5:3), white crystals, 26% (0.16 g), Mp. 96–99 °C, (HPLC 97%). IR: ν_max_ (ATR) cm^−1^: 3120, 3051, 3010, 1605, 1509, 1496, 1276, 1228, 1207, 1160, 1137, 1015. ^1^H NMR (400 MHz, CDCl_3_) δ 6.72 (s, 1 H, CH-N-R), 7.02–7.08 (m, 2 H, Ar-H), 7.08–7.13 (m, 4 H, Ar-H), 7.35–7.39 (m, 3 H, Ar-H), 7.91 (s, 1 H, CH-N), 8.01 (s, 1 H, CH-N). ^13^C NMR (101 MHz, CDCl_3_) δ 67.13 (CH-N-R), 115.83 (CH), 116.05 (CH), 128.00 (2 × CH), 128.76 (CH), 129.04 (2 × CH), 129.90 (CH), 129.98 (CH), 133.85 (C), 137.74 (C), 143.47 (CH-N), 152.41 (CH-N), 163.87 (C-F). HRMS (EI): found 252.0941 (M – H)^+^; C_15_H_11_FN_3_ requires 252.0937. 

##### 2,2,2-Trifluoro-*N*-(4-(Phenyl(1H-1,2,4-Triazol-1-yl)Methyl)Phenyl)Acetamide (**13i**)

As per general method B, compound **12j** (1 eq, 1.67 mmol, 0.49 g) was reacted with 1,2,4-triazole and *p*-TSA in toluene. The crude product was purified via flash chromatography (eluent: *n*-hexane/ethyl acetate 1:1) to afford a pale yellow solid, 26%, 0.154 g, Mp. 143–145 °C. IR: ν_max_ (ATR) cm^−1^: 3112, 3039, 1725, 1612, 1560, 1503, 1252, 1209, 1186, 1159, 1135. ^1^H NMR (400 MHz, CDCl_3_) δ 6.74 (s, 1 H, CH-N-R), 7.16 (d, *J* = 8.54 Hz, 4 H, Ar-H), 7.36 (d, *J* = 1.83 Hz, 3 H, Ar-H), 7.58 (d, *J* = 8.54 Hz, 2 H, Ar-H), 7.92 (s, 1 H, CH-N), 8.02 (s, 1 H, CH-N). ^13^C NMR (101 MHz, CDCl_3_) δ 154.64 (C=O), 152.33 (CH-N), 143.54 (CH-N), 137.34 (C), 135.97 (C-NH), 135.40 (C), 129.08 (2 × CH), 128.88 (CH), 128.11 (2 × CH), 120.80 (2 × CH), 114.14 (CF_3_), 67.23 (CH-N-R). HRMS (EI): found 347.1116 (M + H)^+^; C_17_H_14_F_3_N_4_O requires 347.1119. 

##### 1-((4-(Benzyloxy)Phenyl)(Phenyl)Methyl)-1*H*-1,2,4-Triazole (**13j**)

As per general method B, compound **12k** (1 eq, 2.41 mmol, 0.7 g) was reacted with 1,2,4-triazole and *p*-TSA in toluene. The crude product was purified via flash chromatography (eluent: *n*-hexane/ethyl acetate 1:2) to afford a white solid, 54%, 0.44 g, Mp. 132–134 °C. IR: ν_max_ (ATR) cm^−1^: 3089, 3033, 3050, 2888, 2853, 1576, 1452, 1379, 1289, 1276, 1245, 1171, 1041. ^1^H NMR (400 MHz, CDCl_3_) δ 5.04 (s, 2 H, CH_2_), 6.69 (s, 1 H, CH-N-R), 6.95 (d, *J* = 8.54 Hz, 2 H, Ar-H), 7.05–7.09 (m, 4 H, Ar-H), 7.31–7.42 (m, 8 H, Ar- H), 7.89 (s, 1 H, CH-N), 8.00 (s, 1 H, CH-N). ^13^C NMR (101 MHz, CDCl_3_) δ 158.93 (C-O), 152.24 (CH-N), 143.43 (CH-N), 138.32 (C), 136.57 (C), 130.10 (C), 129.63 (2 × CH), 128.86 (2 × CH), 128.60 (2 × CH), 128.40 (2 × CH), 128.07 (CH), 127.75 (2 × CH), 127.42 (CH), 115.19 (2 × CH), 70.07 (CH-N-R), 67.35 (CH_2_). LRMS (EI): found 341.90 (M^+^); C_22_H_19_N_3_O requires 341.15. 

##### 1-((4-Ethoxyphenyl)(Phenyl)Methyl)-1*H*-1,2,4-Triazole (**13l**)

As per general method B, compound **12m** (1 eq, 1.98 mmol, 0.45 g) was reacted with 1,2,4-triazole and *p*-TSA in toluene. The crude product was purified via flash chromatography (eluent: *n*-hexane/ethyl acetate 1:1) to afford white crystals, 98%, 0.54 g, Mp. 98–100 °C, (HPLC 97%). IR: ν_max_ (ATR) cm^−1^: 3091, 2926, 1611, 1579, 1468, 1458, 1430, 1390, 1375, 1248, 1138, 1015. ^1^H NMR (400 MHz, CDCl_3_) δ 1.41 (t, *J* = 7.02 Hz, 3 H, CH_3_), 4.03 (q, *J* = 6.71 Hz, 2 H, CH_2_), 6.71 (s, 1 H, CH-N-R), 6.86–6.90 (m, 2 H, Ar-H), 7.05–7.11 (m, 4 H, Ar-H), 7.32–7.40 (m, 3 H, Ar-H), 7.90 (s, 1 H, CH-N), 8.02 (s, 1 H, CH-N). ^13^C NMR (101 MHz, CDCl_3_) δ 14.76 (CH_3_), 63.55 (CH_2_), 67.42 (CH-N-R), 114.82 (2 × CH), 127.74 (CH), 128.38 (2 × CH), 128.86 (2 × CH), 129.63 (2 × CH, C), 138.43 (C), 143.45 (CH-N), 152.22 (CH-N), 159.14 (C-O). HRMS (EI): found 280.1447 (M + H)^+^; C_17_H_18_N_3_O requires 280.1450. 

##### 1-((4-(Benzyloxy)phenyl)(3,4,5-trimethoxyphenyl)methyl)-1*H*-1,2,4-triazole (**16b**)

As per general method B, compound **15b** (1 eq, 1.06 mmol, 0.405 g) was reacted with 1,2,4-triazole (3 eq, 3.19 mmol, 0.22 g) and *p*-TSA (0.61 eq, 200 mg) in toluene (60 mL). The crude product was purified via flash chromatography (eluent: *n*-hexane/ethyl acetate 1:1), pale yellow oil, 72%, 0.33 g. IR: ν_max_ (ATR) cm^−1^: 1589, 1504, 1453, 1330, 1230, 1175, 1120, 1005. ^1^H NMR (400 MHz, CDCl_3_) δ 3.73 (s, 6 H, OCH_3_), 3.82 (s, 3 H, OCH_3_), 5.06 (s, 2 H, CH_2_), 6.28 (s, 2 H, Ar-H), 6.62 (s, 1 H, CH-N-R), 6.96 (d, *J* = 8.54 Hz, 2 H, Ar-H), 7.08 (d, *J* = 8.54 Hz, 2 H, Ar-H), 7.31–7.42 (m, 5 H, Ar-H), 7.91 (s, 1 H, CH-N), 8.01 (s, 1 H, CHN). ^13^C NMR (101 MHz, CDCl_3_) δ 158.98 (C), 153.54 (2 × C), 152.25 (CH), 143.47 (CH), 137.97 (C), 136.53 (C), 133.76 (C), 129.84 (C), 129.55 (2 × CH), 128.62 (2 × CH), 128.11 (CH), 127.44 (2 × CH), 115.24 (2 × CH), 104.98 (2 × CH), 70.09 (CH_2_), 67.43 (CH), 60.84 (OCH_3_), 56.12 (2 × OCH_3_). HRMS (EI): found 454.1748 (M+Na)^+^; C_25_H_25_N_3_NaO_4_ requires 454.1743. 

##### 1-(Phenyl(3,4,5-Trimethoxyphenyl)Methyl)-1*H*-1,2,4-Triazole (**16e**)

As per general method B, compound **15e** (1 eq, 3.2 mmol, 0.87 g) was reacted with 1,2,4-triazole (3 eq, 9.6 mmol, 0.66 g) and *p*-TSA (0.6 eq, 200 mg) in toluene (60 mL). The crude product was purified via flash chromatography (eluent: *n*-hexane/ethyl acetate 1:1), white crystals, 70%, 0.735 g, Mp. 139–140 °C, (HPLC 100%). IR: ν_max_ (ATR) cm^−1^: 3119, 2944, 1592, 1502, 1454, 1434, 1420, 1332, 1270, 1219, 1120. ^1^H NMR (400 MHz, CDCl_3_) δ 3.74 (s, 6 H, OCH_3_), 3.83 (s, 3 H, OCH_3_), 6.32 (s, 2 H, Ar-H), 6.67 (s, 1 H, CH-N-R), 7.13 (d, *J* = 7.32 Hz, 2 H, Ar-H), 7.35–7.40 (m, 3 H, Ar-H), 7.93 (s, 1 H, CH-N), 8.02 (s, 1 H, CH-N). ^13^C NMR (101 MHz, CDCl_3_) δ 56.10 (2 × OCH_3_), 60.83 (OCH_3_), 67.89 (CH-N-R), 105.32 (2 × CH), 128.01 (2 × CH), 128.70 (CH), 128.94 (2 × CH), 133.32 (C), 137.69 (C-O), 138.10 (C), 143.55 (CH-N), 152.32 (CH-N), 153.56 (2 × C-O). HRMS (EI): found 348.1314 (M+Na)^+^; C_18_H_19_N_3_NaO_3_ requires 348.1324. 

##### 1-((3-(Benzyloxy)-4-Methoxyphenyl)(3,4,5-Trimethoxyphenyl)Methyl)-1*H*-1,2,4-Triazole (**19a**)

As per general method B, compound **18a** (1 eq, 2.43 mmol, 1.0 g) was reacted with 1,2,4-triazole (3 eq, 7.3 mmol, 0.5 g) and *p*-TSA (200 mg) in toluene (60 mL). The toluene was evaporated, and the crude product was dissolved in ethyl acetate (30 mL), washed with water (20 mL), brine (10 mL), dried over sodium sulphate and concentrated under reduced pressure, white solid, 64%, 0.71 g, Mp. 98–100 °C. IR: ν_max_ (ATR) cm^−1^: 3440, 2938, 1591, 1517, 1464, 1416, 1253, 1127, 1006. ^1^H NMR (CDCl_3_, 400 MHz) δ 8.02 (s, 1H, CH-N), 7.83 (s, 1H, CH-N), 7.29–7.35 (m, 5H, Ar-H), 6.89 (d, *J* = 8.53 Hz, 1H, Ar-H), 6.69–6.74 (m, 1H, Ar-H), 6.66 (d, *J* = 2.01 Hz, 1H, Ar-H), 6.59 (s, 1H, CH-N-R), 6.23 (s, 2H, Ar-H), 5.08 (s, 2H, CH_2_), 3.91 (s, 3H, OCH_3_), 3.86 (s, 3H, OCH_3_), 3.72 (s, 6H, OCH_3_). ^13^C NMR (CDCl_3_, 100 MHz) δ 153.0 (COBn), 151.8 (CH-N), 149.5 (C), 147.6 (C), 143.0 (CH-N), 137.4 (C), 136.0 (C), 133.2 (C), 129.3 (C), 128.1 (2 × CH), 127.6 (2 × CH), 126.8 (CH), 120.9 (CH), 113.7 (CH), 111.2 (CH), 104.3 (2 × CH), 77.0 (CH_2_), 70.5 (CH-N-R), 67.0 (OCH_3_), 60.4 (OCH_3_), 55.6 (OCH_3_), 55.5 (OCH_3_). HRMS (EI): 484.1833 (M + Na)^+^; C_26_H_27_N_3_NaO_5_ requires 484.1848. 

##### 4-((3-(Benzyloxy)-4-Methoxyphenyl)(1*H*-1,2,4-Triazol-1-yl)Methyl)Benzonitrile (**19f**)

As per the general method B, **18e** (1 eq., 600 mg, 1.73 mmol), 1,2,4-triazole (3 eq., 360 mg, 5.21 mmol) and *p*-TSA (0.5 eq, 0.87 mmol, 150 mg) were reacted in a microwave reactor. The fixed microwave was set to 120 W for the duration of the reaction. The product was purified via flash chromatography on silica gel (DCM: EtOAc, gradient 25:1 to 10:1) to afford a yellow solid, 507 mg, 74%, Mp. 91–93 °C. IR: ν_max_ (KBr) cm^−1^: 3032, 2934, 2228, 1607, 1513, 1441, 1264, 1139, 1015. ^1^H NMR (CDCl_3_, 400 MHz) δ 8.04 (s, 1H, CH), 7.88 (s, 1H, CH), 7.61 (d, *J* = 8.53 Hz, 2H, Ar-H), 7.30–7.37 (m, 5H, Ar-H), 7.06 (d, *J* = 8.03 Hz, 2H, Ar-H), 6.91 (d, *J* = 8.03 Hz, 1H, Ar-H), 6.76 (dd, *J* = 1.51, 8.03 Hz, 1H, Ar-H), 6.64 (d, *J* = 13.55 Hz, 2H, Ar-H), 5.10 (s, 2H, CH_2_), 3.92 (s, 3H, OCH_3_). ^13^C NMR (CDCl_3_, 100 MHz) δ 151.9 (NCHN), 150.0 (C_q_), 147.8 (C_q_), 143.1 (NCHN), 135.9 (C_q_), 132.1, 128.2, 127.7, 127.7, 127.6, 126.7, 121.5, 117.8 (C_q_), 114.2, 111.8, 111.3 (C_q_), 70.6 (CH_2_), 66.5 (CH), 55.6 (OCH_3_). HRMS (EI): 395.1516 (M – H)^-^; C_24_H_19_N_4_O_2_ requires 395.1508. 

##### 4-((4-(Benzyloxy)Phenyl)(1*H*-1,2,4-Triazol-1-yl)Methyl)Benzonitrile (**19g**)

As per general method B, **18f** (1 eq., 3 g, 9.51 mmol), 1,2,4-triazole (3 eq., 1.69 g, 24.5 mmol) and *p*-TSA (1.0 eq, 1.74 mmol, 300 mg) were reacted for 4 h. The fixed microwave was set to 95 W for the duration of the reaction. The material was purified via flash chromatography on silica gel (DCM: EtOAC, gradient 25:1 to 10:1) to afford a pale yellow solid, 2.33 g, 67%, Mp. 79–81 °C. IR: ν_max_ (KBr) cm^−1^: 3443, 3118, 2920, 2227, 1608, 1561, 1511, 1418, 1394, 1217, 1169, 1154, 1039. ^1^H NMR (CDCl_3_, 400 MHz) δ 8.07 (s, 1H, CH), 7.99 (s, 1H, CH), 7.68 (d, *J* = 8.53 Hz, 2H, Ar-H), 7.33–7.48 (m, 5H, Ar-H), 7.19 (d, *J* = 8.53 Hz, 2H, Ar-H), 7.11–7.16 (m, 2H, Ar-H), 6.98–7.05 (m, 2H, Ar-H), 6.75 (s, 1H, CH), 5.10 (s, 2H, CH_2_). ^13^C NMR (CDCl_3_, 100 MHz) δ 159.0 (COBn), 152.2 (NCHN), 143.4 (NCHN), 135.9 (C_q_), 132.2, 129.7, 128.3, 128.0, 127.8 (C_q_), 127.0, 117.8 (C_q_), 115.1, 111.9 (C_q_), 69.7 (CH_2_), 66.4 (CH). HRMS (EI): 365.1408 (M – H)^-^; C_23_H_17_N_4_O requires 365.1402. 

##### 5-((2*H*-1,2,3-Triazol-1-yl)(3,4,5-Trimethoxyphenyl)Methyl)-2-Methoxyphenol (**24**)

As per general method B, compound **15i** (1 eq, 0.2 mmol, 0.5 g) was reacted with 1,2,3-triazole (3 eq, 1.5 mmol, 0.33 g) of and *p*-TSA (0.61 eq, 200 mg) in toluene (60 mL). The crude product was purified via flash column chromatography (eluent: *n*-hexane/ethyl acetate 1:1), yellow oil, 77%, 0.143 g, (HPLC 98%). IR: ν_max_ (ATR) cm^−1^: 3392, 2939, 2838, 1590, 1507, 1458, 1419, 1330, 1275, 1236, 1120, 1001, 1025. ^1^H NMR (400 MHz, DMSO-*d_6_*) δ 9.01 (s, 1H, OH), 7.83 (s, 2H, Ar-H), 6.99 (s, 1H, CH), 6.83 (d, *J* = 8.4 Hz, 1H, Ar-H), 6.65 (d, *J* = 2.2 Hz, 1H, Ar-H), 6.60 (s, 2H, Ar-H), 6.56 (dd, *J* = 8.4, 2.1 Hz, 1H, Ar-H), 3.70 (s, 3H, OCH_3_), 3.65 (s, 6H, OCH_3_), 3.62 (s, 3H, OCH_3_). ^13^C NMR (101 MHz, DMSO-*d_6_*) δ 153.12 (2 × C-O), 147.78 (C-O), 146.67 (C-O), 137.49 (C), 135.21 (C), 134.90 (CH), 131.93 (CH, C), 119.34 (CH), 115.79 (CH), 112.28 (CH), 105.96 (2 × CH), 70.96 (CH-N-R), 60.42 (OCH_3_), 56.29 (2 × OCH_3_), 56.01 (OCH_3_). LRMS (EI): found 394.26 (M + Na)^+^; C_19_H_21_N_3_NaO_5_ requires 394.14. 

#### 4.1.4. 4-(Phenyl(1*H*-1,2,4-Triazol-1-yl)Methyl)Aniline (**13m**)

To a solution of compound **13i** (1 eq, 0.44 mmol, 0.154 g) in MeOH and water, K_2_CO_3_ (4 eq, 1.78 mmol, 0.25g) was added. The mixture was stirred for 72 h; then, it was acidified with HCl 10% (50 mL) and extracted with DCM (3 × 25 mL). The acid phase was made alkaline with aqueous NaOH (15%) and extracted with DCM (3 × 25 mL). The organic extracts were combined, dried over Na_2_SO_4_, filtered and concentrated under reduced pressure, orange solid, 16%, 0.043 g, Mp. 149–151 °C. IR: ν_max_ (ATR) cm^−1^: 3357, 3194, 3124, 1611, 1517, 1504, 1273, 1209, 1184, 1134, 1022. ^1^H NMR (400 MHz, DMSO-*d_6_*) δ 5.15 (s, 2 H, NH), 6.49 (m, 2 H, Ar-H), 6.77 (s, 1 H, CH-N-R), 6.88 (d, *J* = 8.54 Hz, 2 H, Ar-H), 7.09 (d, *J* = 7.32 Hz, 2 H, Ar-H), 7.22–7.35 (m, 3 H, Ar-H), 7.98 (s, 1 H, CH-N), 8.40 (s, 1 H, CH-N). ^13^C NMR (101 MHz, DMSO-*d*_6_) δ 65.76 (CH-N-R), 113.57 (2 × CH), 125.32 (2 × CH), 127.44 (2 × CH), 127.51 (C), 128.38 (CH), 129.15 (2 × CH), 140.02 (C), 143.92 (CH-N), 148.58 (C-NH_2_), 151.60 (CH-N). LRMS (EI): found 251.18 (M^+^); C_15_H_16_N_4_ requires 251.13. 

#### 4.1.5. 4-(Phenyl(1*H*-1,2,4-Triazol-1-yl)Methyl)Phenol (**13n**)

Compound **13j** (1 eq, 1.28 mmol, 0.44 g) was stirred in ethyl acetate (20 mL) and palladium hydroxide (0.05 g) under hydrogen atmosphere for 3 h. Then, the crude product was purified via flash chromatography (eluent: *n*-hexane/ethyl acetate 1:2), off-white solid, 67%, 0.217 g, Mp. 189–191 °C, (HPLC 93%). IR: ν_max_ (ATR) cm^−1^: 3104, 2599, 1612, 1590, 1512, 1493, 1449, 1372, 1283, 1238, 1217, 1025. ^1^H NMR (400 MHz, CDCl_3_) δ 6.68 (s, 1 H, CH-N-R), 6.81 (d, *J* = 7.93 Hz, 2 H, Ar-H), 7.01 (s, 2 H, Ar-H), 7.07 (d, *J* = 6.10 Hz, 2 H, Ar-H), 7.34 (s, 3 H, Ar-H), 7.89 (s, 1 H, CH-N), 8.00 (s, 1 H, CH-N). ^13^C NMR (101 MHz, CDCl_3_) δ 156.13 (C-OH), 151.53 (CH-N), 142.78 (CH-N), 138.00 (C), 129.82 (2 × CH), 129.53 (C), 128.92 (2 × CH), 128.52 (CH), 127.70 (2 × CH), 115.89 (2 × CH), 67.62 (CH-N-R). HRMS (EI): found 250.0981 (M - H)^+^; C_15_H_12_N_3_O requires 250.0981. 

#### 4.1.6. General Method C: Preparation of Secondary Alcohols **15b**, **15c**, **15d**, **15g**, **15h**

A solution of the aryl bromide in dry THF was cooled to −78° under nitrogen. *n*-BuLi was added dropwise, and the mixture was allowed to stir for 1 h under nitrogen. After 1 h, a solution of the aryl aldehyde in dry THF was added, and the mixture was stirred for a further 1.5 h at −78 °C. The mixture was stirred at room temperature for 2 h; then, it was concentrated under reduced pressure to remove the THF. The residue was dissolved in DCM (30 mL) and washed with water (20 mL) and brine (10 mL), dried over sodium sulfate, filtered, and concentrated. Then, the crude product was purified via flash chromatography (eluent: *n*-hexane/ethyl acetate).

##### (4-(Benzyloxy)Phenyl)(3,4,5-Trimethoxyphenyl)Methanol (**15b**)

As per general method C, compound **14b** (1 eq, 5.2 mmol, 1.37 g) in dry THF (50 mL) was treated with *n*-BuLi (2.4 mL) followed by the addition 3,4,5-trimethoxybenzaldehyde (1 eq, 5.2 mmol, 1 g). The crude product was purified via flash chromatography (eluent: *n*-hexane/ethyl acetate 1:1), white solid, 21%, 0.405 g, Mp. 98–102 °C. IR: ν_max_ (ATR) cm^−1^: 3477, 2935, 2833, 1590, 1503, 1451, 1418, 1329, 1301, 1287, 1231, 1178, 1121, 1002. ^1^H NMR (400 MHz, CDCl_3_) δ 3.81 (s, 9 H, CH_3_), 5.04 (s, 2 H, CH_2_), 5.72 (s, 1 H, CH-OH), 6.58 (s, 2 H, Ar-H), 6.93 (d, *J* = 8.54 Hz, 2 H, Ar-H), 7.24–7.31 (m, 3 H, Ar-H), 7.31–7.37 (m, 2 H, Ar-H), 7.40 (t, *J* = 7.02 Hz, 2 H, Ar-H). ^13^C NMR (101 MHz, CDCl_3_) δ 56.08 (2 × OCH_3_), 60.81 (OCH_3_), 70.03 (CH_2_), 75.85 (CH-OH), 103.38 (2 × CH), 114.83 (2 × CH), 127.44 (2 × CH), 127.90 (CH), 127.98 (C), 128.42 (2 × CH), 136.16 (C-O), 136.88 (2 × C), 139.58 (2 × CH), 153.23 (2 × C-O), 158.34 (C-OBn). LRMS (EI)**:** Found 403.21 (M + Na)^+^; C_23_H_24_NaO_5_ requires 403.15. 

##### (4-Methoxyphenyl)(3,4,5-Trimethoxyphenyl)Methanol (**15c**)

*Method (i)* As per general method C, 1-bromo-4-methoxybenzene **14c** (1 eq, 7.2 mmol, 1.34 g) in dry THF (50 mL) was treated with *n*-BuLi (3.3 mL) followed by the addition after 1 h of 3,4,5-trimethoxybenzaldehyde (1 eq, 7.2 mmol, 1.41 g). The crude product was purified via flash chromatography (eluent: *n*-hexane/ethyl acetate gradient 7:3 to 1:1), pink solid, 22%, 0.5 g, Mp. 107–109 °C [103]. IR: ν_max_ (ATR) cm^−1^: 3358, 2936, 2837, 1611, 1590, 1508, 1459, 1423, 1325, 1234, 1125, 1055, 1034, 1000. ^1^H NMR (400 MHz, CDCl_3_) δ 3.79 (s, 3 H, CH_3_), 3.81 (s, 9 H, CH_3_), 5.73 (d, *J* = 2.44 Hz, 1 H, CH-OH), 6.59 (s, 2 H, Ar-H), 6.86 (d, *J* = 8.54 Hz, 2 H, Ar-H), 7.28 (d, *J* = 8.54 Hz, 2 H, Ar-H). ^13^C NMR (101 MHz, CDCl_3_) δ 55.26 (OCH_3_), 56.07 (2 × OCH_3_), 60.80 (OCH_3_), 75.85 (CH-OH), 103.36 (2 × CH), 113.87 (2 × CH), 127.86 (C), 135.89 (C), 137.13 (C-O), 139.63 (2 × CH), 153.21 (2 × C-O), 159.11 (C-O). HRMS (EI): found 327.1221 (M+Na)^+^ C_17_H_20_NaO_5_ requires 327.1209. *Method (ii)* As per general method A, compound **22c** (1 eq, 4.16 mmol, 1.26 g) was treated with sodium borohydride (3 eq, 12.48 mmol, 0.47 g). The product was obtained as a pink solid, 50%, 0.6 g, Mp. 107–109 °C, which was identical (^1^H-NMR, ^13^C-NMR, HRMS, IR) to the sample obtained through general method C.

##### (3,4-Dimethoxyphenyl)(3,4,5-Trimethoxyphenyl)Methanol (**15d**)

*Method (i)* As per general method C, compound **14d** (1 eq, 7.2 mmol, 1.56 g) in dry THF (50 mL) was treated with *n*-BuLi (3.3 mL) followed by the addition of 3,4,5-trimethoxybenzaldehyde (1 eq, 7.2 mmol, 1.41 g). The crude product was purified via flash chromatography (eluent: *n*-hexane/ethyl acetate 5:3) as a dark oil, 30%, 0.725 g [67]. IR: ν_max_ (ATR) cm^−1^: 3503, 2936, 2835, 1587, 1504, 1452, 1413, 1328, 1259, 1229, 1120, 1024, 1003. ^1^H NMR (400 MHz, CDCl_3_) δ 3.82–3.83 (m, 9 H, CH_3_), 3.86 (s, 3 H, CH_3_), 3.86 (s, 3 H, CH_3_), 5.71 (d, *J* = 2.90 Hz, 1 H, CH-OH), 6.60 (s, 2 H, Ar-H), 6.83 (d, *J* = 8.29 Hz, 1 H, Ar-H), 6.88 (dd, *J* = 8.29, 1.66 Hz, 1 H, Ar-H), 6.93 (d, *J* = 2.07 Hz, 1 H, Ar-H). ^13^C NMR (101 MHz, CDCl_3_) δ 55.82 (2 × OCH_3_), 56.02 (2 × OCH_3_), 60.74 (OCH_3_), 75.90 (CH-OH), 103.44 (2 × CH), 109.74 (CH), 110.86 (CH), 118.93 (CH), 136.25 (C), 137.10 (C), 139.50 (C-O), 148.45 (C-O), 148.94 (C-O), 153.11 (2 × C-O). HRMS (EI): found 357.1305 (M+Na)^+^; C_18_H_22_NaO_6_ requires 357.1314. *Method (ii)* As per general method A, compound **20a** (1 eq, 3.9 mmol, 1.3 g) was treated with sodium borohydride (3 eq, 11.73 mmol, 0.44 g). The product was afforded as a dark oil, 89%, 1.16 g which was identical (^1^H-NMR, ^13^C-NMR, HRMS, IR) to the sample obtained by general method C.

#### 4.1.7. 4-((1*H*-1,2,4-Triazol-1-yl)(3,4,5-Trimethoxyphenyl)Methyl)Phenol (**16i**)

Compound **16b** (1 eq, 0.76 mmol, 0.330 g) was dissolved in ethyl acetate (20 mL) and stirred with palladium hydroxide (0.05 g) under a hydrogen atmosphere. The reaction mixture was filtered through Celite, and the solvent was concentrated. The crude product was purified via flash chromatography (eluent: *n*-hexane/ethyl acetate 1:2), yellow oil, 49%, 0.16 g, (HPLC 96%). IR: ν_max_ (ATR) cm^−1^: 3113, 2999, 2939, 2837, 1592, 1505, 1459, 1420, 1332, 1274, 1236, 1123. ^1^H NMR (400 MHz, CDCl_3_) δ 3.76 (s, 6 H, OCH_3_), 3.85 (s, 3 H, OCH_3_), 6.31 (s, 2 H, Ar-H), 6.63 (s, 1 H, CH-N-R), 6.85 (d, *J* = 8.54 Hz, 2 H, Ar-H), 7.06 (d, *J* = 8.54 Hz, 2 H, Ar-H), 7.94 (s, 1 H, CH-N), 8.04 (s, 1 H, CH-N). ^13^C NMR (101 MHz, CDCl_3_) δ 56.13 (2 × OCH_3_), 60.85 (OCH_3_), 67.58 (CH-N-R), 105.01 (2 × CH), 115.94 (2 × CH), 129.67 (2 × CH), 133.64 (2 × C), 137.41 (C-O), 143.43 (CH-N), 151.97 (CH-N), 153.55 (2 × C-O), 156.58 (C-OH). HRMS (EI): found 340.1302 (M – H)^+^; C_18_H_18_N_3_O_4_ requires 340.1297. 

##### 4.1.8. 5-((1*H*-1,2,4-Triazol-1-yl)(3,4,5-Trimethoxyphenyl)methyl)-2-Methoxyphenol (**19e**)

Compound **19a** (1 eq, 0.43 mmol, 0.2 g) was dissolved in ethyl acetate (20 mL) and stirred with palladium hydroxide (0.05 g) under hydrogen atmosphere. The reaction mixture was filtered through Celite, and the solvent was concentrated. The crude product was purified via flash chromatography (eluent: *n*-hexane/ethyl acetate 1:2), white solid, 76%, 0.12 g, Mp. 61–63 °C. IR: ν_max_ (ATR) cm^−1^: 3389, 2936, 1591, 1508, 1461, 1276, 1127, 1010. ^1^H NMR (CDCl_3_, 400 MHz) δ 8.04 (s, 1H, CH-N), 7.97 (s, 1H, CH-N), 6.86 (d, *J* = 8.53 Hz, 1H, Ar-H), 6.76 (d, *J* = 2.01 Hz, 1H, Ar-H), 6.67 (dd, *J* = 2.01, 8.03 Hz, 1H, Ar-H), 6.61 (s, 1H, CH-N-R), 6.33 (s, 2H, Ar-H), 3.90 (s, 3H, OCH_3_), 3.85 (s, 3H, OCH_3_), 3.77 (s, 6H, OCH_3_). ^13^C NMR (CDCl_3_, 100 MHz) δ 153.1 (2 × C-O), 151.6 (CH-N), 146.5 (C-OH), 145.6 (C-O), 143.0 (CH-N), 137.4 (C-O), 133.2 (C), 130.0 (C), 119.6 (CH), 114.2 (CH), 110.4 (CH), 104.5 (2 × CH), 67.1 (CH-N-R), 60.4 (OCH3), 55.7 (2 × OCH_3_), 55.5 (OCH_3_). HRMS (EI): 394.1363 (M+Na)^+^; C_19_H_21_N_3_NaO_5_ requires 394.1379. 

#### 4.1.9. 4-((3-Hydroxy-4-Methoxyphenyl)(1*H*-1,2,4-Triazol-1-yl)Methyl)Benzonitrile (**19h**)

Compound **19f** (190 mg, 0.479 mmol) and Pd(OH)_2_ (20%, 20 mg) were reacted in ethyl acetate (25 mL) for 60 min under a hydrogen atmosphere. The material was purified via flash chromatography over silica gel (DCM: EtOAc, gradient 10:1 to 5:1) to afford the product as a white solid, 120 mg, 82%, Mp. 168–170 °C. IR: ν_max_ (KBr) cm^−1^: 3123, 2937, 2842, 2230, 1609, 1592, 1505, 1442, 1278, 1132, and 1022. ^1^H NMR (CDCl_3_, 400 MHz) δ 8.07 (s, 1H, CH), 8.05 (s, 1H, CH), 7.68 (d, *J* = 8.53 Hz, 2H, Ar-H), 7.19 (d, *J* = 8.03 Hz, 2H, Ar-H), 6.88 (d, *J* = 8.03 Hz, 1H, Ar-H), 6.77 (s, 1H, CH), 6.66–6.74 (m, 2H, Ar-H), 3.93 (s, 3H, OCH_3_), ^13^C NMR (CDCl_3_, 100 MHz), δ 151.8 (NCHN), 146.9 (C_q_), 145.9 (C_q_), 143.2 (NCHN), 132.2, 128.6 (C_q_), 127.8, 120.2, 117.8 (C_q_), 114.5, 111.9 (C_q_), 110.5, 66.5 (CH), 55.6 (OCH_3_), HRMS (EI): 305.1039 (M – H)^-^, C_17_H_13_N_4_O_2_ requires 305.1039. 

#### 4.1.10. 4-((4-Hydroxyphenyl)(1*H*-1,2,4-Triazol-1-yl)Methyl)Benzonitrile (**19i**)

Compound **19g** (400 mg, 1.09 mmol) and Pd(OH)_2_ (20%, 20 mg) were reacted in ethyl acetate (25 mL) for 60 min under a hydrogen atmosphere. The material was purified via flash chromatography over silica gel (DCM: EtOAc, gradient 10:1 to 5:1) to afford the product as a brown solid, 200 mg, 66%, Mp. 78–81 °C [89]. IR: ν_max_ (KBr) cm^−1^: 3409 (br.), 2333 (CN), 1641, 1607, 1284, 1169. ^1^H NMR (DMSO-d_6_, 400 MHz) δ 10.60 (br. s., 1H, OH), 8.01 (d, *J* = 8.03 Hz, 2H, Ar-H), 7.80 (d, *J* = 8.03 Hz, 2H, Ar-H), 7.67 (d, *J* = 8.53 Hz, 2H, Ar-H), 6.91 (d, *J* = 8.53 Hz, 2H, Ar-H) ^13^C NMR (DMSO-d_6_, 100 MHz) δ 193.1 (C=O), 162.7 (COH), 142.2 (C_q_), 132.8, 132.5, 129.5, 126.9 (C_q_), 118.3 (C_q_), 115.5, 113.8 (C_q_) HRMS (EI): 222.0557 (M – H)^-^, C_14_H_8_NO_2_ requires 222.0555. 

#### 4.1.11. General Method D: Preparation of 1-(Diarylmethyl)-1*H*-Imidazoles

To a solution of the secondary alcohol (1 eq) in dry acetonitrile (60 mL), CDI was added (1.3 eq). The mixture was refluxed for 3 h under nitrogen, the acetonitrile was evaporated, and the crude product was dissolved in DCM (30 mL) and washed with water (20 mL) and brine (10 mL). The product was dried over sodium sulphate and concentrated under reduced pressure. The crude product was purified via flash chromatography over silica gel to afford the desired product (eluent: *n*-hexane/ethyl acetate 1:1).

##### 1-((4-Bromophenyl)(Phenyl)methyl)-1*H*-Imidazole (**20c**)

As per general method D, compound (**12c**) was reacted with CDI in ACN at reflux for 3 h. Then, the crude product was purified via flash chromatography (eluent: *n*-hexane/ethyl acetate 1:2), white solid, 10%, 0.06 g, Mp. 126–128 °C. IR: ν_max_ (ATR) cm^−1^: 3130, 1758, 1487, 1475, 1387, 1289, 1253, 1061. ^1^H NMR (400 MHz, CDCl_3_) δ 6.98 (s, 1 H, CH-N-R), 7.09 (s, 1 H, CH-N), 7.26 (s, 1H, CH-N), 7.34–7.42 (m, 6 H, Ar-H), 7.47 (s, 1 H, Ar-H), 7.52 (d, *J* = 8.54 Hz, 2 H, Ar-H), 8.20 (s, 1 H, CH-N). ^13^C NMR (101 MHz, CDCl_3_) δ 80.51 (CH-N-H), 117.12 (CH-N), 122.91 (C), 127.06 (2 × CH), 128.83 (CH), 128.93 (CH-N), 128.95 (2 × CH), 130.91 (2 × CH), 132.00 (2 × CH), 137.07 (C), 137.36 (C), and 137.74 (CH-N). HRMS (EI): found 313.0337 (M + H)^+^; C_16_H_14_^79^BrN_2_ requires 313.0340. 

##### 1-((3-(Benzyloxy)-4-Methoxyphenyl)(3,4,5-Trimethoxyphenyl)Methyl)-1*H*-Imidazole (**21h**)

As per general method D, compound **18a** (1 eq, 1.71 mmol, 0.70 g) was reacted with CDI in ACN (50 mL) at reflux for 1 h. The crude product was then purified via flash chromatography (eluent: *n*-hexane/ethyl acetate/methanol 10:2:1), colourless oil, 49%, 0.380 g. IR: ν_max_ (ATR) cm^−1^: 2937, 2836, 1590, 1506, 1455, 1419, 1330, 1229, 1122, 1076, 1004. ^1^H NMR (400 MHz, CDCl_3_), δ 3.68 (s, 6 H, OCH_3_), 3.84 (s, 3 H, OCH_3_), 3.88 (s, 3 H, OCH_3_), 5.04 (s, 2 H. CH_2_), 6.16 (s, 2 H, Ar-H), 6.31 (s, 1 H, CH-N-R), 6.58 (d, *J* = 2.07 Hz, 1 H), 6.66 (dd, *J* = 8.29, 2.07 Hz, 1 H, Ar-H), 6.73 (s, 1 H, Ar-H), 6.84 (d, *J* = 8.71 Hz, 1 H, CH-N), 7.05 (s, 1 H, CH-N), 7.26–7.31 (m, 5 H, Ar-H), 7.33 (s, 1 H, CH-N). ^13^C NMR (101 MHz, CDCl_3_), δ 56.00 (OCH_3_), 56.07 (2 × OCH_3_), 60.85 (OCH_3_), 64.60 (CH_2_), 70.98 (CH-N-R), 104.79 (2 × CH), 111.60 (CH), 114.16 (CH), 119.21 (CH), 121.16 (CH), 127.29 (3xCH), 127.94 (CH), 128.52 (2 × CH), 129.29 (C), 131.14 (C), 136.56 (2 × C), 137.71 (CH), 147.98 (C-O), 149.75 (C-OBn), 153.41 (2 × C-O). HRMS (EI): found 461.2056 (M + H)^+^; C_27_H_29_N_2_O_5_ requires 461.2076. 

##### 1-(Bis(3,4,5-Trimethoxyphenyl)Methyl)-1*H*-Imidazole (**21i**)

As per general method D, compound **18b** (1 eq, 1.38 mmol, 0.51 g) was reacted with CDI in ACN (50 mL) at reflux for 3 h. Then, the crude product was purified via flash chromatography (eluent: *n*-hexane/ethyl acetate 1:1) to afford an orange solid, 52%, 0.3 g, Mp. 154–156 °C, (HPLC 100%). IR: ν_max_ (ATR) cm^−1^: 2934, 2830, 1589, 1504, 1455, 1329, 1226, 1121, 1106, 1003. ^1^H NMR (400 MHz, CDCl_3_) δ 3.77 (s, 12 H, OCH_3_), 3.87 (s, 6 H, OCH_3_), 6.32 (s, 4 H, Ar-H), 6.38 (s, 1 H, CH-N-R), 6.88 (s, 1 H, CH-N), 7.12 (s, 1 H, CH-N), 7.44 (s, 1 H, CH-N). ^13^C NMR (101 MHz, CDCl_3_) δ 56.22 (4xOCH_3_), 60.91 (2 × CH_3_), 65.15 (CH-N-R), 105.24 (4xCH), 119.35 (CH-N), 129.44 (CH-N), 134.47 (2 × C), 137.44 (2 × C-O), 138.02 (CH-N), 153.53 (4xC-O). HRMS (EI): found 413.1718 (M – H)^+^; C_22_H_25_N_2_O_6_ requires 413.1713.

#### 4.1.12. 4-((1*H*-Imidazol-1-yl)(Phenyl)Methyl)Aniline (**20l**)

To a solution of compound **20i** (1 eq, 0.20 mmol, 0.07 g) in MeOH and water K_2_CO_3_ (4 eq, 0.81 mmol, 0.11 g) was added. The mixture was stirred for 72 h and then acidified with HCl 10% (50 mL) and extracted with DCM (3 × 25 mL). The acid phase was made basic with NaOH (15%) and extracted with DCM (3 × 25 mL). Organic phases combined, dried over Na_2_SO_4_, filtered, and concentrated. The crude product was purified via flash chromatography (eluent: *n*-hexane/ethyl acetate 1:1), orange oil, [122] 50%, 0.01 g. IR: ν_max_ (ATR) cm^−1^: 3130, 1696, 1613, 1560, 1515, 1491, 1283, 1259, 1215, 1151, 1103, 1068. ^1^H NMR (400 MHz, CDCl_3_) δ 3.72 (br. s., 2 H, NH_2_), 6.39 (s, 1 H, CH-N-R), 6.62 (d, *J* = 8.54 Hz, 2 H, Ar-H), 6.82 (s, 1 H, CH-N), 6.89 (d, *J* = 7.93 Hz, 2 H, Ar-H), 7.02–7.07 (m, 3 H, Ar-H), 7.27–7.35 (m, 3 H, Ar-H), 7.38 (s, 1 H, CH-N). ^13^C NMR (101 MHz, CDCl3), δ 64.62 (CH-N-R), 115.01 (2 × CH), 118.07 (CH), 127.60 (3xCH), 127.99 (C), 128.67 (3xCH), 129.42 (2 × CH), 139.90 (C, CH), 146.52 (C-NH_2_), HRMS (EI): found 250.1338 (M + H)^+^; C_16_H_16_N_3_ requires 250.1344. 

#### 4.1.13. 5-((1H-Imidazol-1-yl)(3,4,5-Trimethoxyphenyl)Methyl)-2-Methoxyphenol (**21l**)

1-((3-(Benzyloxy)-4-methoxyphenyl)(3,4,5-trimethoxyphenyl)methyl)-1*H*-imidazole (**21h**) (1 eq, 0.83 mmol, 0.38 g) was stirred in ethyl acetate (20 mL) and palladium hydroxide (0.05 g) under hydrogen atmosphere for 1 h. The product was filtered through Celite, and the solvent was removed under reduced pressure, off-white solid, 93%, 0.28 g, Mp. 154–157 °C, (HPLC 97%). IR: ν_max_ (ATR) cm^−1^: 3136, 2932 2836, 1589, 1532, 1452, 1437, 1331, 1294, 1223, 1121, and 1084. ^1^H NMR (400 MHz, DMSO-*d_6_*) δ 9.05 (s, 1H, OH), 7.58 (s, 1H, Ar-H), 7.07 (s, 1H, Ar-H), 6.91 (s, 1H, Ar-H), 6.87 (d, *J* = 8.4 Hz, 1H, Ar-H), 6.57 (s, 1H, CH-N-R), 6.54 (d, *J* = 2.2 Hz, 1H, Ar-H), 6.47 (dd, *J* = 8.3, 2.1 Hz, 1H, Ar-H), 6.43 (s, 2H, Ar-H), 3.71 (s, 3H, OCH_3_), 3.65 (s, 6H, OCH_3_), 3.62 (s, 3H, OCH_3_). ^13^C NMR (101 MHz, DMSO-*d_6_*) δ 153.34 (2 × C-O), 147.72 (C-OH), 146.90 (C-O), 137.45 (CH-N), 136.30 (C-O), 132.66 (2 × C), 128.84 (CH-N), 119.69 (CH-N), 118.98 (CH), 115.48 (CH), 112.53 (CH), 105.71 (2 × CH), 63.56 (CH), 60.44 (OCH_3_), 56.32 (2 × OCH_3_), 56.01 (OCH_3_). HRMS (EI): found 369.1453 (M – H)^+^; C_20_H_21_N_2_O_5_ requires 369.1451. 

#### 4.1.14. (4-Methoxyphenyl)(3,4,5-trimethoxyphenyl)methanone (**23a**)

Anisole (1 eq, 7.0 mmol, 0.75 g 0.75 mL) was reacted with 3,4,5-trimethoxybenzoic acid (1.5 eq, 10.5 mmol, 2.23 g) in Eaton’s reagent (0.99 g P_2_O_5_/6.69 mL CH_3_SO_3_H). The mixture was stirred at 60 °C for 3 h under N_2_. The product was diluted in DCM (60 mL) and poured in a separatory funnel containing NaHCO_3_ 50% (40 mL) and extracted. The crude product was purified via flash chromatography (eluent: *n*-hexane/ethyl acetate 5:4) to afford a pink solid, 60%, 1.26 g, Mp. 76–81 °C [103]. IR: ν_max_ (ATR) cm^−1^: 3401, 2951, 2837, 1641, 1600, 1510, 1494, 1445, 1332, 1305, 1233, 1250, 1111, 1018, 1025, 922, 840, 696, 761, 738, 696, 611. ^1^H NMR (400 MHz, CDCl_3_) δ 3.88 (s, 6 H, OCH_3_), 3.90 (s, 3 H, OCH_3_), 3.94 (s, 3 H, OCH_3_), 6.98 (d, *J* = 8.53 Hz, 2 H, Ar-H), 7.02 (s, 2 H, Ar-H), 7.83 (d, *J* = 9.03 Hz, 2 H, Ar-H). ^13^C NMR (101 MHz, CDCl_3_), δ 55.46 (OCH_3_), 56.25 (2 × OCH_3_), 60.92 (OCH_3_), 107.40 (2 × CH), 113.50 (2 × CH), 130.23 (C), 132.34 (2 × CH), 133.30 (C), 141.54 (C-O), 152.78 (2 × C-O), 163.08 (C-O), 194.61 (C=O). HRMS (EI): Found 325.1056 (M+Na)^+^; C_17_H_18_NaO_5_ requires 325.1052. (Eaton’s reagent was prepared from phosphorus pentoxide and methanesulfonic acid in a weight ratio P_2_O_5_:CH_3_SO_3_H of 1:10, mixed in a round-bottomed flask and heated at 40 °C under nitrogen atmosphere until homogenous).

#### 4.1.15. (3,4-Dimethoxyphenyl)(3,4,5-Trimethoxyphenyl)Methanone (**23b**)

1,2-Dimethoxybenzene (1 eq, 7.24 mmol, 1 g) was reacted with 3,4,5-trimethoxybenzoic acid (1.5 eq, 10.86 mmol, 2.30 g) in Eaton’s reagent (1.02 g P_2_O_5_/7.24 mL CH_3_SO_3_H). The mixture was stirred at 60 °C for 3 h under N_2_. The product was diluted in DCM (60 mL) and poured in a separatory funnel containing NaHCO_3_ 50% (40 mL) and extracted. The crude product was purified via flash chromatography (eluent: *n*-hexane/ethyl acetate 5:4) to afford an orange solid, 57%, 1.37 g, Mp. 128–131 °C [67]. IR: ν_max_ (ATR) cm^−1^: 2942, 1640, 1599, 1576, 1411, 1330, 1256, 1232, 1118, 1026. ^1^H NMR (400 MHz, CDCl_3_) δ 3.89 (s, 6 H, OCH_3_), 3.94 (s, 3 H, OCH_3_), 3.95 (s, 3 H, OCH_3_), 3.98 (s, 3 H, OCH_3_), 6.92 (d, *J* = 8.29 Hz, 1 H, CH), 7.04 (s, 2 H, Ar-H), 7.40 (d, *J* = 2.07 Hz, 1 H, Ar-H), 7.47 (d, *J* = 2.07 Hz, 1 H, Ar-H). ^13^C NMR (101 MHz, CDCl_3_) δ 56.07 (2 × OCH_3_), 56.30 (2 × OCH_3_), 60.96 (OCH_3_), 107.44 (2 × CH), 109.76 (CH), 112.25 (CH), 125.04 (CH), 130.30 (2 × C), 148.95 (2 × C-O), 152.80 (3xC-O), 194.62 (C=O). HRMS (EI): Found 333.1330 (M + H)^+^; C_18_H_21_O_6_ requires 333.1338.

#### 4.1.16. General Method E for the Preparation of Diarylmethylpyrrolidines, Diarylmethylpiperidines and Diarylmethylpiperazines

The benzhydryl alcohol (1 eq) was reacted with thionyl chloride (5 eq) in dry DCM (30 mL) for 12 h. The reaction mixture was concentrated under reduced pressure, and the crude product was used in the next step without any further purification. The chlorinated benzhydryl alcohol was reacted with pyrrolidine or piperidine (5 eq) in dry ACN (30 mL) and refluxed for 12 h. The solvent was removed, and the residue was dissolved in DCM (50 mL) and washed with 1 M NaOH (30 mL). The organic phase was dried over sodium sulphate, filtered, and concentrated. Then, the crude product was purified via flash chromatography (eluent: *n*-hexane/ethyl acetate).

##### 1-((4-Bromophenyl)(Phenyl)methyl)Pyrrolidine (**25b**)

As per general method E, compound **12c** (1 eq, 2.9 mmol, 0.82 g) was treated with thionyl chloride followed by reaction with pyrrolidine (5 eq, 14.5 mmol, 1.03 g, 1.20 mL) in acetonitrile (ACN) (50 mL) at reflux for 12 h. The crude product was purified via flash chromatography (eluent: *n*-hexane/ethyl acetate 9:1), white solid, 37%, 0.345, Mp. 70–72 °C, (HPLC 95%). IR: ν_max_ (ATR) cm^−1^: 2964, 2784, 1648, 1585, 1484, 1450, 1280, 1194, 1070, 1009. ^1^H NMR (400 MHz, CDCl_3_) δ 1.71–1.80 (m, 4 H, CH_2_), 2.35–2.43 (m, 4 H, CH_2_), 4.11 (s, 1 H, CH-N.R), 7.14–7.19 (m, 1 H, Ar-H), 7.23 (s, 1 H, Ar-H), 7.27 (s, 1 H, Ar-H), 7.31–7.42 (m, 6 H, Ar-H). ^13^C NMR (101 MHz, CDCl_3_) δ 23.53 (2 × CH_2_), 53.55 (2 × CH_2_), 75.72 (CH-N-R), 120.44 (C-Br), 127.00 (CH), 127.36 (2 × CH), 128.45 (2 × CH), 129.15 (2 × CH), 131.46 (2 × CH), 143.44 (C), 143.75 (C). HRMS (EI): found 316.0711 (M + H)^+^; C_17_H_19_^79^BrN requires 316.0701. 

##### 1-((4-Methoxyphenyl)(3,4,5-Trimethoxyphenyl)Methyl)Piperidine (**26b**)

As per general method E, compound 15c (1 eq, 1.83 mmol, 0.59 g) was treated with thionyl chloride (5 eq) in dry DCM (30 mL) for 12 h, then reacted with piperidine (5 eq, 9.15 mmol, 0.78 g, 0.90 mL) in dry ACN (50 mL) and refluxed for 12 h. The product did not require any further purification, brown oil, 54%, 0.37 g, (HPLC 95%). IR: ν_max_ (ATR) cm^−1^: 2956, 2813, 1598, 1507, 1450, 1419, 1331, 1230, 1174, 1145, 1125, 1031. ^1^H NMR (400 MHz, CDCl_3_) δ 1.42 (d, *J* = 4.98 Hz, 2 H, CH_2_), 1.52–1.57 (m, 4 H, CH_2_), 2.28 (d, *J* = 4.15 Hz, 4 H, CH_2_), 3.76 (s, 3 H, OCH_3_), 3.78 (s, 3 H, OCH_3_), 3.82 (s, 6 H, OCH_3_), 4.06 (s, 1 H, CH-N-R), 6.63 (s, 2 H, Ar-H), 6.80 (d, *J* = 8.71 Hz, 2 H, Ar-H), 7.27 (d, *J* = 8.71 Hz, 2 H, Ar-H). ^13^C NMR (101 MHz, CDCl_3_) δ 158.38 (C-O), 153.00 (2 × C-O), 139.44 (C), 136.40 (C-O), 134.97 (C), 128.97 (2 × CH), 113.60 (2 × CH), 104.46 (2 × CH), 76.03 (CH-N-R), 60.74 (OCH_3_), 56.03 (2 × OCH_3_), 55.14 (OCH_3_), 53.04 (2 × CH_2_), 26.23 (2 × CH_2_), 24.68 (2 × CH_2_). LRMS (EI): found 372.17 (M + H)^+^; C_22_H_30_NO_4_ requires 372.21. 

##### 1-((4-Methoxyphenyl)(3,4,5-Trimethoxyphenyl)Methyl)-4-Phenylpiperazine (**27c**)

As per general method E, compound **15c** (1 eq, 0.805 mmol, 0.26 g) was reacted with 1-phenyl-piperazine (5 eq, 4.02 mmol, 0.81 g, 0.79 mL) in dry ACN (40 mL) and refluxed for 12 h under nitrogen atmosphere. The crude product was purified via flash chromatography (eluent: *n*- hexane/ethyl acetate 1:1) to afford a white solid, 74%, 0.26 g, Mp. 125 ^o^C, (HPLC 98%). IR: ν_max_ (ATR) cm^−1^: 2950, 2823, 1590, 1501, 1451, 1418, 1386, 1301, 1227, 1122, 1176, 1032, 1004. ^1^H NMR (400 MHz, CDCl_3_) δ 2.53 (dt, *J* = 10.47, 4.92 Hz, 4 H, CH_2_), 3.18 (t, *J* = 4.77 Hz, 4 H, CH_2_), 3.77 (s, 3 H, OCH_3_), 3.78 (s, 3 H, OCH_3_), 3.83 (s, 6 H, OCH_3_), 4.11 (s, 1 H, CH-N-R), 6.67 (s, 2 H, Ar-H), 6.83 (d, *J* = 8.71 Hz, 2 H, Ar-H), 6.89 (d, *J* = 8.29 Hz, 2 H, Ar-H), 7.21–7.26 (m, 3 H, Ar-H), 7.33 (d, *J* = 8.71 Hz, 2 H, Ar-H). ^13^C NMR (101 MHz, CDCl_3_) δ 158.65 (C-O), 153.20 (2 × C-O), 151.26 (C), 138.76 (C), 136.65 (C-O), 134.42 (C), 129.04 (2 × CH), 128.88 (2 × CH), 119.48 (CH), 115.74 (2 × CH), 113.87 (2 × CH), 104.37 (2 × CH), 75.56 (CH-N-R), 60.76 (OCH_3_), 56.07 (2 × OCH_3_), 55.18 (OCH_3_), 51.86 (CH_2_), 49.18 (CH_2_). LRMS (EI): found 449.19 (M + H)^+^; C_27_H_33_N_2_O_4_ requires 449.24. 

##### Tert-Butyl 4-((4-Methoxyphenyl)(3,4,5-Trimethoxyphenyl)Methyl)Piperazine-1-Carboxylate (**27e**)

As per general method E, compound **15c** (1 eq, 0.86 mmol, 0.28 g) was treated with excess thionyl chloride and then reacted with BOC-piperazine (5 eq, 4.3 mmol, 0.8 g) in dry ACN (40 mL) and refluxed for 12 h under nitrogen atmosphere. The product was obtained as a yellow oil, 80%, 0.32 g. IR: ν_max_ (ATR) cm^−1^: 3475, 2938, 2835, 1682, 1591, 1503, 1451, 1418, 1240, 1120, 1175, 1120, 1002, 1078. ^1^H NMR (400 MHz, CDCl_3_) δ 2.07 (s, 9 H, CH_3_), 2.78 (s, 4 H, CH_2_), 3.37 (s, 4 H, CH_2_), 3.74 (s, 3 H, OCH_3_), 3.75 (s, 3H, OCH_3_), 3.80 (s, 6H, OCH_3_), 4.04 (s, 1 H, CH-N-R), 6.61 (s, 2 H, Ar-H), 6.80 (d, *J* = 8.8 Hz, 2 H, Ar-H), 7.27 (d, *J* = 8.7 Hz, 2 H, Ar-H). ^13^C NMR (101 MHz, CDCl_3_) δ 158.64 (C-O), 154.77 (C=O), 153.17 (2 × C-O), 138.50 (C), 136.67 (C-O), 134.12 (C), 128.85 (2 × CH), 113.85 (2 × CH), 104.37 (2 × CH), 79.60 (CH-N-R), 75.42 (C-(CH_3_)_3_), 60.73 (OCH_3_), 56.04 (2 × OCH_3_), 55.15 (OCH_3_), 51.62 (2 × CH_2_), 45.73 (2 × CH_2_), 28.38 (3xCH_3_). LRMS (EI): found 473.26 (M + H)^+^; C_26_H_37_N_2_O_6_ requires 473.26. 

##### 1-((3-(Benzyloxy)-4-Methoxyphenyl)(3,4,5-Trimethoxyphenyl)Methyl)-4-Phenylpiperazine (**27g**)

As per general method E, compound **18a** (1 eq, 0.58 mmol, 0.25 g) was first reacted with thionyl chloride; then, it was treated with phenylpiperazine (5 eq, 2.91 mmol, 0.47 g, 0.44 mL) in dry ACN (50 mL) at reflux for 12 h. The crude product was purified via flash column chromatography (eluent: *n*-hexane/ethyl acetate 1:1) to afford the product as off-white powder, 67%, 0.21 g, Mp. 124–126 ^°C^, (HPLC 99%). IR: ν_max_ (ATR) cm^−1^: 2957, 2831, 1599, 1507, 1450, 1419, 1232, 1174, 1119, 1031, 925, 823, 726, 698. ^1^H NMR (400 MHz, CDCl_3_) δ 2.41–2.54 (m, 4 H, CH_2_), 3.11–3.13 (m, 4 H, CH_2_), 3.80 (s, 9 H, OCH_3_), 3.86 (s, 3 H, OCH_3_), 4.05 (s, 1 H, CH-N-R), 5.16 (s, 2 H. CH_2_), 6.60 (s, 2 H, Ar-H), 6.81–6.85 (m, 1 H, Ar-H), 6.90 (d, *J* = 8.29 Hz, 2 H,Ar-H), 6.92–6.95 (m, 1 H, Ar-H), 6.99–7.01 (s, 1 H, Ar-H), 7.23–7.29 (m, 4 H, Ar-H), 7.32–7.34 (m, 2 H, Ar-H), 7.38–7.43 (m, 2 H, Ar-H). ^13^C NMR (101 MHz, CDCl_3_) δ 49.15 (2 × CH_2_), 51.73 (2 × CH_2_), 55.95 (2 × OCH_3_), 56.08 (OCH_3_), 60.79 (OCH_3_), 71.06 (CH_2_), 75.51 (CH-N-R), 104.37 (2 × CH), 111.56 (C), 113.85 (CH), 115.72 (2 × CH), 119.48 (CH), 120.84 (CH), 127.25 (CH), 127.79 (2 × CH), 128.48 (2 × CH), 129.07 (2 × CH), 134.78 (C-O), 137.16 (2 × C), 138.48 (C), 147.93 (C-O), 148.84 (C), 151.26 (C-OBn), 153.17 (2 × C-O). LRMS (EI): found 555.16 (M + H)^+^; C_34_H_39_N_2_O_5_ requires 555.29.

##### 1,4-bis((4-Methoxyphenyl)(3,4,5-Trimethoxyphenyl)Methyl)Piperazine (**28**)

As per general method E, compound **15c** (1 eq, 0.99 mmol, 0.32 g) was treated with excess thionyl chloride, followed by a reaction with piperazine (5 eq, 4.95 mmol, 0.42 g) in dry ACN (40 mL), and the reaction mixture was refluxed for 12 h under nitrogen atmosphere. The crude product was purified via flash chromatography (eluent: *n*- hexane/ethyl acetate 1:1). The product was obtained as a brown solid, 12%, 0.08 g, Mp. 220 ^o^C. IR: ν_max_ (ATR) cm^−1^: 3059, 3025, 2957, 2819, 1599, 1450, 1419, 1331, 1300, 1287, 1232, 1174, 1108, 1031. ^1^H NMR (400 MHz, CDCl_3_) δ 2.40 (br. s, 8 H, CH_2_), 3.73 (s, 6 H, OCH_3_), 3.75 (s, 6 H, OCH_3_), 3.80 (s, 12 H, OCH_3_), 4.08 (s, 2 H, CH-N-R), 6.62 (s, 4 H, Ar-H), 6.78 (d, *J* = 7.88 Hz, 4 H, Ar-H), 7.28 (d, *J* = 7.46 Hz, 4 H, Ar-H). **^1^**^3^C NMR (101 MHz, CDCl_3_), δ 158.57 (2 × C-O), 153.10 (4xC-O), 138.59 (2 × C), 136.62 (2 × C-O), 134.27 (2 × C), 128.91 (4xCH), 113.74 (4xCH), 104.49 (4xCH), 75.70 (2 × CH), 60.73 (2 × OCH_3_), 56.06 (4xOCH_3_), 55.16 (2 × OCH_3_), 52.00 (4xCH_2_). LRMS (EI): found 658.77 (M)^+^; C_38_H_46_N_2_O_8_ requires 658.33. 

#### 4.1.17. 1-((4-Methoxyphenyl)(3,4,5-Trimethoxyphenyl)Methyl)Piperazine (**27h**)

A solution of **27e** (0.21 mmol, 0.1 g) in DCM (15 mL) was treated with trifluoroacetic acid (TFA) (1 mL) for 30 min at 20 ^o^C. The reaction mixture was quenched with NaHCO_3_, washed with water and brine, dried over sodium sulphate, filtered, and concentrated to afford the product as a yellow oil, 42%, 0.03 g. IR: ν_max_ (ATR) cm^−1^: 3477, 2937, 2835, 1689, 1591, 1504, 1451, 1418, 1326, 1234, 1119, 1078, 1034. ^1^H NMR (400 MHz, CDCl_3_) δ 3.41 (br. s., 4 H, CH_2_), 3.46 (br. s., 4 H, CH_2_), 3.77 (s, 3 H, OCH_3_), 3.79 (s, 3 H, OCH_3_), 3.83 (s, 6 H, OCH_3_), 4.07 (s, 1 H, CH-N-R), 6.64 (s, 2 H, Ar-H), 6.83 (d, *J* = 8.71 Hz, 2 H, Ar-H), 7.30 (d, *J* = 8.29 Hz, 2 H, Ar-H). ^13^C NMR (101 MHz, CDCl_3_) δ 45.22 (2 × CH_2_), 51.65 (2 × CH_2_), 55.19 (OCH_3_), 56.07 (2 × OCH_3_), 60.76 (OCH_3_), 75.44 (CH-N-R), 103.71 (2 × CH), 104.40 (2 × CH), 113.82 (2 × CH), 128.89 (C), 129.25 (C), 134.16 (C-O), 138.53 (C), 153.20 (2 × C-O), 158.68 (C-O). LRMS (EI): found 372.09 (M)^+^ C_21_H_28_N_2_O_4_ requires 372.20. 

#### 4.1.18. 2-Methoxy-5-((4-Phenylpiperazin-1-yl)(3,4,5-Trimethoxyphenyl)Methyl)Phenol (**27i**)

Compound (**27g**) (1 eq, 0.21 mmol, 0.1 g) was stirred in ethyl acetate (25 mL) and palladium hydroxide (0.05 g) under a hydrogen atmosphere. The reaction mixture was filtered through Celite and the solvent evaporated to afford the product as a light brown oil, 45%, 0.04 g, (HPLC 97%). IR: ν_max_ (ATR) cm^−1^: 3475, 2937, 2834, 1591, 1503, 1451, 1418, 1327, 1231, 1119, 1077. ^1^H NMR (400 MHz, CDCl_3_) δ 2.47–2.62 (m, 4 H, CH_2_), 3.18–3.20 (m, 4 H, CH_2_), 3.80 (s, 3 H, OCH_3_), 3.83–3.88 (d, 9H, OCH_3_), 4.06 (s, 1 H, CH-N-R), 5.58 (s, 1 H, OH), 6.69 (s, 2 H, Ar-H), 6.78 (d, *J* = 8.29 Hz, 1 H, Ar-H), 6.84 (t, 1 H, Ar-H), 6.91 (d, *J* = 7.88 Hz, 3 H, Ar-H), 7.05 (s, 1 H, Ar-H), 7.25–7.27 (m, 2 H, Ar-H). ^13^C NMR (101 MHz, CDCl_3_), δ 49.21 (2 × CH_2_), 51.88 (2 × CH_2_), 55.89 (OCH_3_), 56.09 (2 × OCH_3_), 60.77 (OCH_3_), 75.74 (CH), 104.34 (2 × CH), 110.44 (CH), 113.82 (2 × CH), 115.75 (CH), 119.40 (2 × CH), 128.20 (2 × CH), 135.78 (C-O), 138.70 (C), 145.63 (C), 151.30 (2 × C-O), 153.22 (C), 156.37 (2 × C-O). LRMS (EI): found 464.95 (M + H)^+^ C_27_H_33_N_2_O_5_ requires 464.23. 

### 4.2. Stability Study of Compounds **21l** and **24**

Stability studies for compounds **21l** and **24** were performed by analytical HPLC using a Symmetry^®^ column (C18, 5 mm, 4.6 × 150 mm), a Waters 2487 Dual Wavelength Absorbance detector, a Waters 1525 binary HPLC pump, and a Waters 717 plus Autosampler (Waters Corporation, Milford, MA, USA). Samples were detected at λ 254 nm using acetonitrile (70%)/water (30%) as the mobile phase over 15 min and a flow rate of 1 mL/min. Stock solutions of the compounds are prepared using 10 mg of compounds **21l** and **24** in 10 mL of mobile phase (1 mg/mL). Phosphate buffers at the desired pH values (4, 7.4, and 9) were prepared following the British Pharmacopoeia monograph 2020. Then, 30 μL of stock solution was diluted with 1 mL of appropriate buffer, shaken, and injected immediately. Samples were withdrawn and analysed at time intervals of t = 0 min, 5 min, 30 min, 60 min, and hourly for 24 h. 

### 4.3. X-ray Crystallography

Data for samples **16e**, **16f**, **19c**, **21e**, and **26a** were collected on a Bruker APEX DUO using Mo Kα and Cu Kα radiation (λ = 0.71073 and 1.54178 Å). Each sample was mounted on a MiTeGen cryoloop and data were collected at 100(2) K using an Oxford Cobra cryosystem. Bruker APEX [123] software was used to collect and reduce data, determine the space group, solve, and refine the structures. Absorption corrections were applied using SADABS 2014 [124]. Structures were solved with the XT structure solution program [125] using Intrinsic Phasing and refined with the XL refinement package [126] using Least Squares minimisation. All non-hydrogen atoms were refined anisotropically. Hydrogen atoms were assigned to calculated positions using a riding model with appropriately fixed isotropic thermal parameters. Molecular graphics were generated using OLEX2 [127]. All structures are racemates. In **26a**, the disordered fluorine was modelled in two positions with occupancies of 84% and 16%. Geometric restraints (SADI) were used to model the C-F bond lengths. Crystallographic data for the structures in this paper have been deposited with the Cambridge Crystallographic Data Centre as supplementary publication nos. 201543, 2015432, 2015433, 2015434, and 2015435. Copies of the data can be obtained, free of charge, on application to CCDC, 12 Union Road, Cambridge CB2 1EZ, UK, (fax: +44-(0)1223-336033 or e-mail:deposit@ccdc.cam.ac.uk).

### 4.4. Biochemical Evaluation of Activity

All biochemical assays were performed in triplicate and on at least three independent occasions for the determination of mean values reported.

#### 4.4.1. Cell Culture

The human breast carcinoma cell line MCF-7 was purchased from the European Collection of Animal Cell Cultures (ECACC) and cultured in Eagles minimum essential medium with 10% foetal bovine serum, 2 mM l-glutamine, and 100 μg/mL penicillin/streptomycin. The medium was supplemented with 1% non-essential amino acids. The human breast carcinoma cell line MDA-MB-231 was purchased from the ECACC. MDA-MB-231 cells were maintained in Dulbecco’s modified Eagle’s medium (DMEM) supplemented with 10% (*v*/*v*) foetal bovine serum, 2 mM l-glutamine, and 100 μg/mL penicillin/streptomycin (complete medium). HL-60 cells were derived from a patient with acute myeloid leukaemia and were obtained from the ECACC (Salisbury, UK). Cells were cultured in RPMI-1640 Glutamax medium supplemented with 10% FCS media and 100 μg/mL penicillin/streptomycin. MCF-10A cells were obtained as a kind gift from Dr Susan McDonnell, School of Chemical and Bioprocess Engineering, University College Dublin and were cultured in Dulbecco’s Modified Eagle Medium: Nutrient Mixture F-12 (DMEM/F12; Gibco) supplemented with 5% horse serum (Invitrogen), 20 ng mL^−1^ epidermal growth factor (Merck Millipore), 0.5 μg mL^−1^ hydrocortisone (Sigma,-Aldrich, Arklow, Co. Wicklow, Ireland), 100 ng mL^−1^ cholera toxin (Sigma, Aldrich, Arklow, Co. Wicklow, Ireland), 10 μg mL^−1^ insulin (Sigma, Aldrich, Arklow, Co. Wicklow, Ireland), and penicillin/streptomycin 5000 U mL^−1^ (1%)(Gibco, Biosciences, 3 Charlemont Terrace, Crofton Road, Dun Laoghaire, Co Dublin, A96 K7H7, Ireland). Cells were maintained at 37 °C in 5% CO_2_ in a humidified incubator. All cells were sub-cultured 3 times/week by trypsinisation using TrypLE Express (1×).

#### 4.4.2. Cell Viability Assay

Cells were seeded at a density of 2.5 × 10^4^ cells/well (MCF-7, MDA-MB-231, MCF-10A cells) and 1 × 10^4^ cells/well (HL-60) in 96-well plates (200 μL per well). After 24 h, cells were then treated with either medium alone, vehicle control (1% ethanol (*v*/*v*)) or with serial dilutions of CA-4 (**4a**), phenstatin (**7a**) or selected compounds (0.001–100 μM) in triplicate. Cell proliferation for MCF-7, MDA-MB-231, and MCF-10A cells was analysed using the alamarBlue assay (Invitrogen Corp.) according to the manufacturer’s instructions. After 67–69 h, alamarBlue (10% (*v*/*v*)) was added to each well, and plates were incubated for 3–5 h at 37 °C in the dark. Fluorescence was read using a 96-well fluorimeter with excitation at 530 nm and emission at 590 nm. Results were expressed as percentage viability relative to vehicle control (100%). Dose–response curves were plotted, and IC_50_ values (concentration of drug resulting in 50% reduction in cell survival) were obtained using the commercial software package Prism (GraphPad Software, Inc., La Jolla, CA, USA). 

#### 4.4.3. Cell Cycle Analysis

Cells were seeded at a density of 1 × 10^5^ cells/well in 6-well plates (3 mL) and treated with indicated compound **19e**, **24**, and phenstatin (**7a**), (1 µM) for 24, 48, or 72 h. The cells were collected by trypsinisation and centrifuged at 800× *g* for 15 min. Cells were washed twice with ice-cold phosphate-buffered saline (PBS) and fixed in ice-cold 70% ethanol overnight at −20 °C. Fixed cells were centrifuged at 800× *g* for 15 min and stained with 50 μg/mL of PI, containing 50 μg/mL of DNase-free RNase A, at 37 °C for 30 min. The DNA content of cells (10,000 cells/experimental group) was analysed by flow cytometer at 488 nm using a FACSCalibur flow cytometer (BD Biosciences, San Jose, CA, USA), and all data were recorded and analysed using the CellQuest Software (Becton-Dickinson)

#### 4.4.4. Annexin V/PI Apoptotic Assay

Apoptotic cell death was detected by flow cytometry using Annexin V and propidium iodide (PI). MCF-7 and MDA-MB-231 cells were seeded in 6-well plates at a density of 1 × 10^5^ cells/mL (3 mL) and treated with either vehicle (0.1% (*v*/*v*) EtOH), Phenstatin (**7a**), or **21l** at different concentrations for the selected time. Then, cells were harvested and prepared for flow cytometric analysis. Cells were washed in 1X binding buffer (20× binding buffer: 0.1M 4-(2-hydroxyethyl)-1-piperazineethanesulfonic acid (HEPES), pH 7.4; 1.4 M NaCl; 25 mM CaCl_2_ diluted in dH_2_O) and incubated in the dark for 30 min on ice in Annexin V-containing binding buffer (1:100). Then, cells were washed once in binding buffer and then re-suspended in PI-containing binding buffer (1:1000). Samples were analysed immediately using the BD Accuri flow cytometer (BD Biosciences, 2350 Qume Dr, San Jose, CA, USA) and prism software for analysis of the data (GraphPad Software, Inc., 2365 Northside Dr., Suite 560, San Diego, CA, USA). Four populations are produced during the assay: Annexin V and PI negative (Q4, healthy cells), Annexin V positive and PI negative (Q3, early apoptosis), Annexin V and PI positive (Q2, late apoptosis), and Annexin V negative and PI positive (Q1, necrosis). 

#### 4.4.5. Immunofluorescence Microscopy

Confocal microscopy was used to study the effects of drug treatment on MCF-7 cytoskeleton. For immunofluorescence, MCF-7 cells were seeded at 1 × 10^5^ cells/mL on eight chamber glass slides (BD Biosciences). Cells were treated with vehicle (1% ethanol (*v*/*v*)), CA-4 (0.01 µM), paclitaxel (1 μM), phenstatin (1 μM), compound **19e** (10 μM), or compound **21l** (10 μM) for 16 h. Following treatment, cells were gently washed in PBS, fixed for 20 min with 4% paraformaldehyde in PBS, and permeabilised in 0.5% Triton X-100. Following washes in PBS containing 0.1% Tween (PBST), cells were blocked in 5% bovine serum albumin diluted in PBST. Then, cells were incubated with mouse monoclonal anti-α-tubulin−FITC antibody (clone DM1A) (Sigma) (1:100) for 2 h at room temperature (rt). Following washes in Phosphate Buffered Saline with Tween® 20 (PBST), cells were incubated with Alexa Fluor 488 dye (1:500) for 1 h at rt. Following washes in PBST, the cells were mounted in Ultra Cruz Mounting Media (Santa Cruz Biotechnology, Santa Cruz, CA) containing 4,6-diamino-2-phenolindol dihydrochloride (DAPI). Images were captured by Leica SP8 confocal microscopy with Leica application suite X software. All images in each experiment were collected on the same day using identical parameters. Experiments were performed on three independent occasions.

#### 4.4.6. Evaluation of Expression Levels of Anti-Apoptotic Proteins Mcl-1, Bcl-2 and PARP Cleavage

MCF-7 cells were seeded at a density of 1 × 10^5^ cells/flask (10 mL) in T25 flasks. After 48 h, whole cell lysates were prepared from untreated cells or cells treated with vehicle control (EtOH, 0.1% *v*/*v*) or selected compound **21l** or **24** (1 μM). MCF-7 cells were harvested in Radioimmunoprecipitation assay buffer (RIPA) buffer supplemented with protease inhibitors (Roche Diagnostics), phosphatase inhibitor cocktail 2 (Sigma-Aldrich), and phosphatase inhibitor cocktail 3 (Sigma-Aldrich). Equal quantities of protein (as determined by a bicinchoninic acid assay (BCA assay) were resolved by SDS-PAGE (12%) followed by transfer to polyvinylidene fluoride PVDF membranes. Membranes were blocked in 5% bovine serum albumin/Tris-buffered saline with 0.1% Tween®20 Detergent (TBST) for 1 h. Membranes were incubated in the relevant primary antibodies at 4 °C overnight, washed with TBST, and incubated in horseradish peroxidase-conjugated secondary antibody for 1 h at rt and washed again. Western blot analysis was performed as described above using antibodies directed against Mcl-1 (1:1000) (Millipore), Bcl-2 [1:500] (Millipore), and PARP followed by incubation with a horseradish peroxidase-conjugated anti-mouse antibody (1:2000) (Promega, Madison, WI, USA). All blots were probed with anti-glyceraldehyde 3-phosphate dehydrogenase (GAPDH) antibody (1:5000) (Millipore) to confirm equal loading. Proteins were detected using enhanced chemiluminescent Western blot detection (Clarity Western ECL substrate) (Bio Rad) on the ChemiDoc MP System (Bio Rad). Experiments were performed on three independent occasions.

#### 4.4.7. Tubulin Polymerisation Assay

The assembly of purified bovine tubulin was monitored using a kit, BK006, purchased from Cytoskeleton Inc. (Denver, CO, USA). The assay was carried out in accordance with the manufacturer’s instructions using the standard assay conditions [128]. Briefly, purified (>99%) bovine brain tubulin (3 mg/mL) in a buffer consisting of 80 mM piperazine-N,N’-bis(2-ethanesulfonic acid) (PIPES) (pH 6.9), 0.5 mM ethylene glycol tetraacetic acid (EGTA), 2 mM MgCl_2_, 1 mM guanosine-5’-triphosphate (GTP)GTP and 10% glycerol was incubated at 37 °C in the presence of either vehicle (2% (*v*/*v*) ddH_2_O) paclitaxel, phenstatin (**7a**), or **21l** (all at 10 µM). Light is scattered proportionally to the concentration of polymerised microtubules in the assay. Therefore, tubulin assembly was monitored turbidimetrically at 340 nm in a Spectramax 340 PC spectrophotometer (Molecular Devices, Sunnyvale, CA, USA). The absorbance was measured at 30 s intervals for 60 min.

#### 4.4.8. Cytochrome P450 Assays (CYP19 (Aromatase) and CYP1A1)

The substrate DBF (dibenzylfluorescein) was obtained from Gentest Corporation (Woburn, MA). All human recombinant cytochrome P450 enzymes were purchased from BD Biosciences, San Jose, CA. Aromatase and CYP1A1 inhibition were quantified by measuring the fluorescent intensity of fluorescein, the hydrolysis product of dibenzylfluorescein (DBF), by aromatase, as previously described [118,119]. In brief, the test substance (10 μL) was pre-incubated with a NADPH regenerating system (90 μL of 2.6 mM NADP^+^, 7.6 mM glucose 6-phosphate, 0.8 U/mL glucose 6-phosphate dehydrogenase, 13.9 mM MgCl_2_, and 1 mg/mL albumin in 50 mM potassium phosphate, pH 7.4), for 10 min, at 37 °C, before 100 μL of the enzyme and substrate (E/S) mixture were added (4.0 pmol/well of CYP19/0.4 μM DBF; 5.0 pmol/well of CYP2C8/2.0 μM DBF; 5.0 pmol/well of CYP3A4/2.0 μM DBF and 0.5 pmol/well of CYP1A1/2.0 μM DBF). The reaction mixtures were incubated for 30 min (excepting CYP1A1, 25 min) at 37 °C to allow the generation of product, quenched with 75 μL of 2 N NaOH, shaken for 5 min, and incubated for 2 h at 37 °C to enhance the noise/background ratio. Finally, fluorescence was measured at 485 nm (excitation) and 530 nm (emission). Three independent experiments were performed, each one in triplicate, and the average values were used to construct dose–response curves. At least four concentrations of the test substance were used, and the IC_50_ value was calculated (*Tablecurve^TM^2D,* AISN Software, EUA, 1996). Naringenin was used as positive controls, yielding an IC_50_ value of 4.9 µM. Compounds **19e**, **21l**, and **24** were dissolved in dimethyl sulfoxide (DMSO) and diluted to final concentrations. An equivalent volume of DMSO was added to control wells, and this had no measurable effect on cultured cells or enzymes. Compounds are considered for further experiments when showing inhibition great than 90%.

### 4.5. Molecular Modelling and Docking Study

The X-ray structure of bovine tubulin co-crystallised with N-deacetyl-N-(2-mercaptoacetyl)-colchicine (DAMA-colchicine) 1SA0 [121] was downloaded from the PDB website. A UniProt Align analysis confirmed a 100% sequence identity between human and bovine β tubulin. The crystal structure was prepared using QuickPrep (minimised to a gradient of 0.001 kcal/mol/Å), Protonate 3D, Residue pKa and Partial Charges protocols in MOE 2015 with the MMFF94x force field [129]. Both enantiomers of selected compounds **19e**, **24**, and **21l** were drawn in ChemBioDraw 13.0, saved as mol files, and opened in MOE. For both enantiomers of each compound, MMFF94x partial charges were calculated, and each was minimised to a gradient of 0.001 kcal/mol/Å. Default parameters were used for docking, except that 300 poses were sampled for each enantiomer, and the top 50 docked poses were retained for subsequent analysis.

## 5. Conclusions

In this work, a novel series of heterocyclic phenstatin-based compounds have been designed and synthesised as tubulin-targeting agents. The structural modifications introduced on the phenstatin moiety included the nitrogen heterocycles 1,2,4-triazole, 1,2,3-triazole, and imidazole to afford a hybrid structure of the vascular targeting agent phenstatin and the aromatase inhibitor letrozole, which contains a 1,2,4-triazole heterocycle. The introduction of aliphatic amines such as pyrrolidine, piperazine, and various piperidine derivatives was also achieved. The resulting compounds were investigated for potential dual activity as tubulin and aromatase inhibitors. All novel compounds were initially evaluated in the MCF-7 breast cancer cell line and of particular interest were compounds **19e**, **21l**, and **24**, which displayed antiproliferative activity in the nanomolar range e.g., **19e** (IC_50_ = 424 nM, **21l** (IC_50_ = 132 nM), and **24** (IC_50_ = 52 nM). They were selected for further studies to provide a better understanding of their mechanism of action in breast cancer cells.

The most potent compounds **21l** and **24** were evaluated in MCF-10A cells (normal breast epithelial cells) for cytotoxicity. Minimal cell death was observed when treated at a concentration similar to the IC_50_ value of the compounds in MCF-7 cells, indicating that the compounds were selective towards cancer cells. Compounds showed impressive antiproliferative activity at nanomolar levels against a range of susceptible human cancer cell lines when tested in the 60 cancer cell line panel of the NCI. Cell cycle analysis of compounds **21l** and **24** resulted in an increase in G_2_/M arrest and apoptotic cell death in MCF-7 cells. Flow cytometric analysis of Annexin V/PI-stained cells indicated that compound **21l** induces the apoptosis of MCF-7 cells in a dose-dependent manner. Compounds **21l** and **24** were also shown to promote PARP cleavage and an inhibition of tubulin polymerisation. The tubulin effects were confirmed when MCF-7 cells treated with the azoles **19e** and **21l** displayed disorganised microtubule networks with similar effects to phenstatin, together with multinucleation.

The molecular docking of selected compounds indicated possible binding to the colchicine-binding site of tubulin and a preference for the *S* enantiomer. The results showed an efficient introduction of the azoles 1,2,4-triazole, 1,2,3-triazole, and imidazole on the phenstatin scaffold structure to retain antiproliferative effects. The selective inhibition of aromatase is an important tool to select compounds that act as chemopreventative agents for hormone-dependent cancer [130]. The aromatase inhibition of the most potent antiproliferative compounds **19e**, **21l**, and **24** was evaluated, and compound **19e** was identified as the most potent with over 85% inhibition of CYP19 at 20 μM and an IC_50_ of 29 μM. We can conclude that the 1,2,4-triazole heterocycle is essential for aromatase inhibition in these compounds, and its activity was optimised when included in a phenstatin-related scaffold such as **19e**. On the basis of the structural modifications of phenstatin described in this work, e.g., introduction of the azoles 1,2,4-triazole, 1,2,3-triazole, and imidazole on the phenstatin scaffold, we have developed lead compounds that exhibit promising anti-cancer properties with potential for further development. The investigation of the stereoselective effects of the compounds together with the optimisation of the dual aromatase–antiproliferative action of compound **19e** is in progress. 

## Figures and Tables

**Table 1:** Crystal data and structure refinement details for compounds **16e**, **16f**, **19c**, **21i**, and **26a**.

Compound	16e	16f	19c	21i	26a
CCDC no.	2015431	2015432	2015433	2015434	2015435
Empirical formula	C_18_H_19_N_3_O_3_	C_19_H_21_N_3_O_3_	C_20_H_23_N_3_O_4_	C_22_H_26_N_2_O_6_	C_18_H_20_FN
M (g/mol)	325.36	339.39	369.41	414.45	269.35
T (K)	100(2)	100(2)	100(2)	100(2)	100(2)
Crystal System	monoclinic	monoclinic	monoclinic	monoclinic	monoclinic
SG	P2_1_/n	P2_1_/c	P2_1_/c	P2_1_/c	P2_1_/c
*a* (Å)	8.1796(5)	9.2487(4)	12.8748(6)	14.1937(6)	5.9228(2)
*b* (Å)	14.1725(10)	9.6620(4)	9.8550(5)	13.1553(5)	14.0705(5)
*c* (Å)	14.4742(10)	20.0006(9)	14.8091(7)	12.4613(5)	17.7920(6)
*α* (°)	90	90	90	90	90
*β* (°)	98.7882(11)	97.8110(10)	93.1792(8)	113.2250(10)	98.275(2)
*γ* (°)	90	90	90	90	90
*V* (Å^3^)	1658.23(19)	1770.69(13)	1876.10(16)	2138.25(15)	1467.29(9)
*Z*	4	4	4	4	4
*Dcalc* (g/cm^3^)	1.303	1.273	1.308	1.287	1.219
μ (mm^−1^)	0.09	0.088	0.092	0.094	0.628
F(000)	688	720	784	880	576
Radiation	MoKα (λ = 0.71073)	MoKα (λ = 0.71073)	MoKα (λ = 0.71073)	MoKα (λ = 0.71073)	CuKα (λ = 1.54178)
Reflections collected	61043	115661	95764	85391	20214
Independent reflections	4298 [R_int_ = 0.0532, R_sigma_ = 0.0237]	5438 [R_int_ = 0.0264, R_sigma_ = 0.0097]	5504 [R_int_ = 0.0364, R_sigma_ = 0.0143]	6261 [R_int_ = 0.0385, R_sigma_ = 0.0188]	2684 [R_int_ = 0.0634, R_sigma_ = 0.0356]
Data/restraints/parameters	4298/0/217	5438/0/230	5504/0/248	6261/0/277	2684/1/191
Goodness-of-fit on F^2^ (*S*)	1.018	1.043	1.046	1.005	1.063
Final R indexes [I ≥ 2σ (I)] *	R_1_ = 0.0407, wR_2_ = 0.0904	R_1_ = 0.0374, wR_2_ = 0.1018	R_1_ = 0.0381, wR_2_ = 0.0938	R_1_ = 0.0405, wR_2_ = 0.0967	R1 = 0.0479, wR2 = 0.1325
Final R indexes [all data]	R_1_ = 0.0613, wR_2_ = 0.1005	R_1_ = 0.0445, wR_2_ = 0.1083	R_1_ = 0.0506, wR_2_ = 0.1025	R_1_ = 0.0580, wR_2_ = 0.1079	R1 = 0.0547, wR2 = 0.1369
Largest diff. peak/hole/e Å-3	0.31/-0.21	0.42/-0.39	0.42/-0.21	0.38/-0.24	0.19/-0.21

* *R*_1_ = Σ||*F_o_*| − |*F_c_*||/ Σ|*F_o_*|, w*R*_2_ = [Σ *w*(F_o_^2^ − F_c_^2^)^2^/Σw(F_o_^2^)^2^]^1/2^.

**Table 2:** Antiproliferative effects of selected azole compounds **19e**, **21l**, and **24** in MCF-7, MDA-MB-231 human breast cancer cells, and HL-60 leukaemia cell line.

Compound	Antiproliferative Activity ^a^ MCF-7 Cells IC_50_ (μM)	Antiproliferative Activity ^a^ MDA-MB-231 Cells IC_50_ (μM)	Antiproliferative Activity ^a^ HL-60 Cells IC_50_ (μM)
**19e**	0.042 ± 0.070	0.978 ± 0.130	0.261^b^
**21l**	0.132 ± 0.007	0.237 ± 0.040	0.156 ± 0.052
**24**	0.052 ± 0.040	0.074 ± 0.030	0.173 ± 0.055
**CA-4**	0.0039 ± 0.00032^c^	0.0430^c^	0.0019 ± 0.0005^c^

^a^ IC_50_ values are half maximal inhibitory concentrations required to block the growth stimulation of MCF-7, MDA-MB-231, and HL-60 cells. Values represent the mean (SEM (error values 10^−6^) for three experiments performed in triplicate. ^b^ Antiproliferative activity for compound **19e** against HL-60 from NCI. ^c^ The IC_50_ value obtained for CA-4 in this assay are in agreement with the reported values for CA-4 in MCF-7 and MDA-MB-231 human breast cancer and leukaemia cell lines (see Refs. [62,102,103]).

**Table 3:** Antiproliferative evaluation of compounds **21l** and **19e** in the NCI 60 cell line in vitro screen ^a^.

Cell Line	Compound 21l ^c^	Compound 19e ^d^	Cell Line	Compound 21l ^c^	Compound 19e ^d^
	GI_50_ (μM) ^b^	GI_50_ (μM) ^b^		GI_50_ (μM) ^b^	GI_50_ (μM) ^b^
***Leukemia***			***Melanoma***		
CCRF-CEM	0.289	0.402	LOX IMV1	0.523	0.523
HL-60 (TB)	0.229	0.261	MALME-3M	Nd ^e^	Nd ^e^
K-562	0.225	0.427	M14	0.180	0.374
MOLT-4	0.565	0.510	MDA-MB-435	0.119	0.181
RPMI-8226	0.385	0.452	SK-MEL-2	0.324	0.361
SR	0.182	0.376	UACC-62	0.668	0.550
***Non-Small Cell Lung Cancer***			SK-MEL-28	Nd ^e^	Nd ^e^
A549/ATCC	0.836	0.969	SK-MEL-5	0.367	0.440
HOP-62	0.516	0.514	UACC-257	1.44	>100
HOP-92	28.6	9.69	***Ovarian Cancer***		
EKVX	0.702	2.24	IGROV1	0.862	0.959
NCI-H226	0.640	1.43	OVCAR-3	0.264	0.323
NCI-H23	0.882	0.977	OVCAR-4	3.44	3.02
NCI-H332M	0.789	0.696	OVCAR-5	0.648	1.99
NCI-H460	0.377	0.414	OVCAR-8	0.450	1.14
NCI-H552	0.247	0.395	NCI/ADR-RES	1.92	1.18
***Colon Cancer***			SK-OV-3	0.465	0.402
COLO 205	0.361	0.332	***Renal Cancer***		
HCT-2998	1.72	1.35	786-0	0.369	0.475
HCT-116	0.249	0.448	A498	0.304	0.324
HCT-15	0.264	0.438	ACHN	0.693	0.653
HT29	0.312	0.330	CAKI-1	0.681	0.567
KM12	0.389	0.418	RXF 393	Nd^e^	0.321
SW-620	0.387	0.431	SN12C	0.910	0.853
***CNS Cancer***			TK-10	Nd^e^	>100
SF-268	0.731	0.469	UO-31	0.883	0.903
SF295	0.307	0.325	***Breast Cancer***		
SF539	0.217	0.300	MCF-7	0.339	0.347
SNB-19	0.532	0.578	MDA-MB-231/ATCC	0.664	1.50
SNB-75	0.192	Nd^e^	HS 578T	0.414	0.548
AC	0.391	0.446	BT-549	0.306	0.618
***Prostate cancer***			T-47D	Nd^e^	Nd^e^
PC-3	0.384	0.431	MDA-MB-468	0.316	0.371
DU-145	0.407	0.530			
**MG-MID (µM) ^f^**	0.234	0.243			

^a^ Data obtained from NCI in vitro human tumour cell screen 5 dose assay. ^b^ GI_50_ is the molar concentration of the compound causing 50% inhibition of growth of the tumour cells; ^c^ NSC 78806; ^d^ NSC 788805; **^e^** Nd: Not determined; ^f^ MG-MID: the mean of GI_50_ values over all cell lines for the tested compound.

**Table 4:** Standard COMPARE analysis of compounds **19e** and **21l** *^a^*.

Rank	19e	*r*	21l	*r*
	**Based on GI_50_ mean graph**		**Based on GI_50_ mean graph**	
1	Vinblastine sulphate, hiConc:-7.6	0.586	Paclitaxel (Taxol) hiConc:-6.0	0.587
2	S-Trityl-L-cysteine	0.575	S-Trityl-L-cysteine	0.58
3	Maytansine	0.547	Paclitaxel (Taxol) hiConc:-4.0	0.544
4	Rhizoxin	0.544	Maytansine	0.53
5	Vinblastine sulphate, hiConc:-5.6	0.509	Paclitaxel (Taxol) hiConc:-4.6	0.518
	**Based on TGI mean graph**		**Based on TGI mean graph**	
1	Vinblastine sulfate	0.737	Maytansine hiConc: -9.0	0.775
2	Rhizoxin	0.726	Vinblastine sulfate	0.765
3	Paclitaxel (Taxol)	0.704	Rhizoxin	0.748
4	Maytansine hiConc:-9.0	0.696	Paclitaxel (Taxol)	0.703
5	Maytansine hiConc:-4.0	0.689	Maytansine hiConc:-9.0	0.700

*^a^* The target set was the standard agent database and the target set endpoints were selected to be equal to the seed endpoints. Standard COMPARE analysis was performed. Correlation values (r) are Pearson correlation coefficients. Vinblastine sulfate and maytansine appear at different concentrations, as it has been tested by the NCI at multiple concentration ranges (see reference 107).

**Table 5:** Inhibitory effect of compounds **19e**, **21l**, and **24** on aromatase and CYP1A1 activity.

Compound	% Inhibition at 20 µg/mL^*a*^		IC_50_ (µM) *^a^*^,*b*^	
	**CYP19**	CYP1A1	CYP19	CYP1A1
19e	85.34	18.08	29.62	>53.85
21l	74.73	18.44	>53.99	>53.99
24	0.01	12.81	>53.85	>53.85

*^a^* The values are mean values of at least three experiments; *^b^* Concentration required to decrease the aromatase and CYP1A1 inhibition activity by 50%.

## Data Availability

Data sharing not applicable.

## Supplementary Materials

The following are available online at https://www.mdpi.com/1424-8247/14/2/169/s1. Tier-1 profiling and Lipinski properties of selected compounds; details of experimental procedures and spectroscopic data; full NCI60 cell line data for compounds **19e**, **21l**, **25g**, **26b** and **27d**.

## Abbreviations

The following abbreviations are used in this manuscript:

AI	Aromatase inhibitor
ADC	Antibody–drug conjugate
ATR	Attenuated total reflection
CDI	1,1’-Carbonyldiimidazole
DEPT	Distortionless Enhancement by Polarization Transfer
DMEM	Dulbecco’s Modified Eagle Medium
DMSO	Dimethyl sulfoxide
ECACC	European Collection of Animal Cell Cultures
EGFR	Epidermal growth factor receptor
ER	Estrogen receptor
FACS	Fluorescence activated cell sorting
FBS	Foetal bovine serum
GI50	50% Growth inhibitory concentration
HER2	Human epidermal growth factor receptor 2
HER/neu	Receptor tyrosine-protein kinase erbB-2, CD340
HDBC	Hormone-dependent breast cancer
HR	Hormone receptor
LC50	Median lethal concentration
MBC	Metastatic breast cancer
MDR	Multidrug resistance
MEM	Minimum essential media
NCI	National Cancer Institute
NMR	Nuclear magnetic resonance
PARP	Poly (ADP-ribose) polymerase
PBS	Phosphate buffered saline
PI	Propidium iodide
PIK3CA	Phosphatidylinositol-4,5-Bisphosphate 3-Kinase Catalytic Subunit Alpha
PR	Progesterone receptor
RIPA	Radioimmunoprecipitation assay
SERM	Selective estrogen receptor modulator
STS	Steroid sulfatase
TGI	Total growth inhibitory concentration
TLC	Thin layer chromatography
TNBC	Triple negative breast cancer
