# Supplementary material for: Synthesis and Biological Evaluation of 1-(Diarylmethyl)-1H-1,2,4-triazoles and 1-(Diarylmethyl)-1H-imidazoles as a Novel Class of Anti-Mitotic Agent for Activity in Breast Cancer
Source: Pharmaceuticals (Basel). 2021 Feb 22;14(2):169. doi: 10.3390/ph14020169 (PMC7926793; doi:10.3390/ph14020169)
Supplement: Supplementary file 1 [file pharmaceuticals-14-00169-s001.pdf]

Supplementary Information

Synthesis and Biological Evaluation of 1-(Diarylmethyl)-1*H*-1,2,4-triazoles and 1-(Diarylmethyl)-1*H*-imidazoles as a Novel Class of Antimitotic Agents

Gloria Ana[1], Patrick M. Kelly[1], Azizah M. Malebari[2], Sara Noorani[1], Seema M Nathwani[3], Brendan Twamley[4], Darren Fayne[3], Niamh M. O'Boyle[1], Daniela M Zisterer[3], Elisangela Flavia Pimentel[5], Denise Coutinho Endringer[5], Mary J. Meegan[1*]

[1] School of Pharmacy and Pharmaceutical Sciences, Trinity College Dublin, Trinity Biomedical Sciences Institute, 152-160 Pearse Street, 2 DO2R590 Dublin, Ireland.

[2] Department of Pharmaceutical Chemistry, College of Pharmacy, King Abdulaziz University, Jeddah 21589, Saudi Arabia

[3] School of Biochemistry and Immunology, Trinity College Dublin, Trinity Biomedical Sciences Institute, 152-160 Pearse Street, Dublin 2, DO2R590, Ireland

[4] School of Chemistry, Trinity College Dublin, Dublin 2, DO2R590, Ireland

[5]Department of Pharmaceutical Sciences, University Vila Velha, Av. Comissário José Dantas de Melo, n°21, Boa Vista Vila Velha - Espírito Santo. CEP 29102-920. Brazil.

* Correspondence: mmeegan@tcd.ie Tel.:+353-1-896-2798;Fax:+353-1-8962793

Table S1. Tier-1 Profiling Screen of Selected 1-(Diarylmethyl)-1*H*-1,2,4-triazoles and 1-(Diarylmethyl)-1*H*-imidazoles...3

Table S2. Lipinski-Properties for Selected 1-(Diarylmethyl)-1*H*-1,2,4-triazoles and 1-(Diarylmethyl)-1*H*-imidazoles...5

Table S3: Comparative Antitumour Evaluations of compounds **19e, 21l, 25g, 26b** and **27d** in the NCI-60 cell line *in vitro* single dose primary screen……………………………..7

Figures S1-S19. NMR data for selected compounds…………………………...……9

Experimental chemistry…………………………………………………………… 20

References ………………………………………………………………… 41

Table S1: Tier-1 Profiling Screen of Selected 1-(Diarylmethyl)-1*H*-1,2,4-triazoles and 1-(Diarylmethyl)-1*H*-imidazoles [a]

Molecule	ID	ADMET Solubility[b]	ADMET Solubility Level[c]	ADMET BBB[d]	ADMET BBB Level[e]	ADMET EXT CYP2D6 Prediction	ADMET EXT Hepatotoxic Prediction
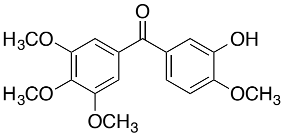	**2a**	-3.6030	3	-0.41800	2	false	true
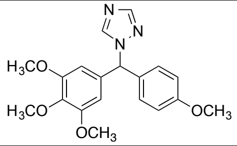	**16c**	-3.9820	3	-0.25600	2	false	true
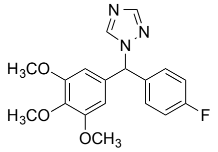	**16g**	-4.2720	2	-0.046000	2	false	true
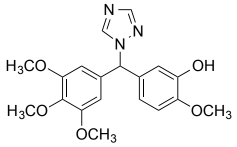	**19e**	-3.6410	3	-0.66000	3	false	true
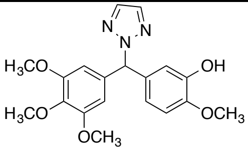	**24**	-3.8300	3	-0.59300	3	false	true
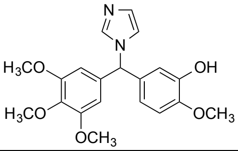	**21l**	-3.6660	3	-0.44300	2	true	true
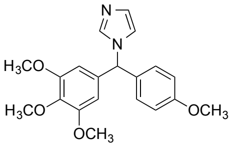	**21e**	-4.0930	2	-0.038000	2	true	true
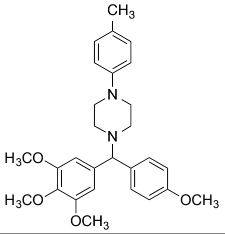	**27f**	-6.2520	1	0.89500	0	false	false

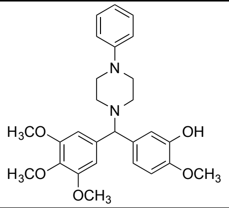	**27i**	-5.3710	2	0.34000	1	false	true
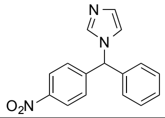	**20b**	-4.2290	2	-0.16300	2	false	true
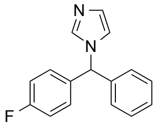	**20d**	-4.3940	2	0.61100	1	true	true
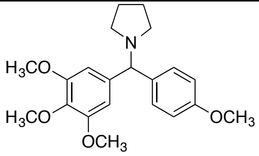	**26g**	-4.8420	2	0.43600	1	true	false
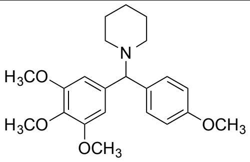	**25b**	-5.2490	2	0.57700	1	true	false

[a]Calculated using Pipeline Pilot Professional (v8.5.0.200) BIOVIA, Dassault Systèmes
[b]ADMET Solubility: Log of the water solubility at 25 °C (LogSw)(mol/L)
[c]ADMET Solubility Level: Ranking of the solubility values into the following classes: 0: Extremely Low; 1: Very Low; 2: Low; 3: Good; 4: Optimal; 5: Very Soluble
[d]ADMET BBB: Predicts the blood brain barrier penetration of a molecule, defined as the ratio of the concentrations of solute (compound) on the both sides of the membrane after oral administration.
[e]ADMET Blood Brain Barrier Absorption (BBB) Level: Ranking of LogBBB values into one of the following levels: 0: Very High; 1: High; 2: Medium; 3: Low; 4: Undefined (molecule is outside the confidence area of the regression model used to calculate LogBB)

Table S2: ADMET and Lipinski Properties for Selected 1-(Diarylmethyl)-1*H*-1,2,4-triazoles and 1-(Diarylmethyl)-1*H*-imidazoles [a]

Molecule	ID	ADMET Absorption Level[b]	ADMET EXT PPB Prediction[c]	LogP[d]	MW	Num HBA	Num HBD	Rot Bonds	Molecular Volume	Molecular Polar Surface Area
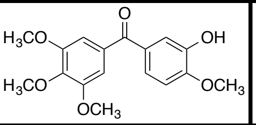	**2a**	0	true	2.35	318.32	6	1	6	210.94	74.220
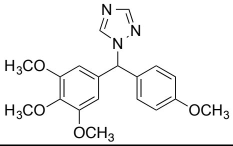	**16c**	0	false	2.93	355.39	6	0	7	235.64	67.630
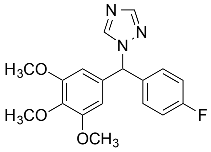	**16g**	0	true	3.09	343.35	5	0	6	221.92	58.400
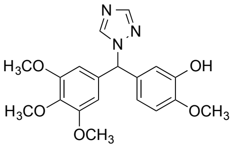	**19e**	0	false	2.41	371.39	7	1	7	241.12	87.860
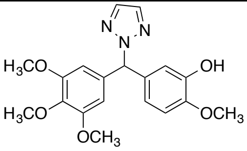	**24**	0	false	3.50	371.39	7	1	7	244.90	87.860
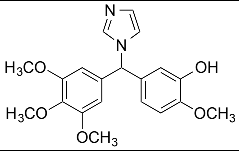	**21l**	0	true	2.91	370.40	6	1	7	247.64	74.970
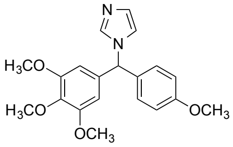	**21e**	0	true	3.30	354.40	5	0	7	242.84	54.740
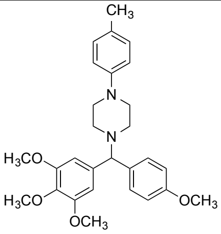	**27f**	0	true	5.50	462.58	6	0	8	328.93	43.400

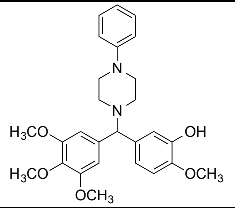	**27i**	0	true	3.92	464.55	7	1	8	326.53	63.630
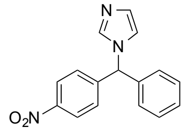	**20b**	0	true	3.01	279.29	3	0	4	178.01	63.640
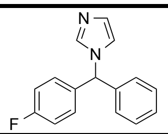	**20d**	0	true	3.96	252.29	1	0	3	164.29	17.820
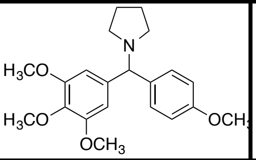	**26g**	0	true	3.50	357.44	5	0	7	257.59	40.160
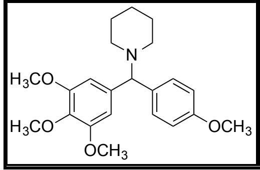	**25b**	0	true	3.92	371.47	5	0	7	269.25	40.160

[a]Calculated using Pipeline Pilot Professional (v8.5.0.200) BIOVIA, Dassault Systèmes
[b]ADMET Absorption Level: Ranking of the molecule into one of the following levels: 0: Good; 1: Moderate; 2: Poor; 3: Very Poor
[c]ADMET Plasma Protein Binding (PPB) Prediction: If true, the compound is predicted to be a binder (>=90%). Otherwise, it is predicted to be a weak or nonbinder(<90%).
[d] ChemBioDraw Ultra 13.0.2.3020

Table S3: Comparative Antitumour Evaluations of compounds **19e, 21l, 25g, 26b** and **27d** in the NCI-60 cell line *in vitro* single dose primary screen[a]

Cell Line	Compound 19e	Compound 21l	Compound 25g	Compound 26b	Compound 27d
	Growth percentage[a, e]	Growth percentage[a, f]	Growth percentage[a, g]	Growth percentage[a, h]	Growth percentage[a, i]
Leukemia					
CCRF-CEM	9.32	6.97	71.21	29.91	68.76
HL-60 (TB)	-32.53 [c]	-35.94 [c]	35.51	4.36	22.98
K-562	8.34	8.00	25.23	18.04	26.38
MOLT-4	16.7	14.01	78.09	31.54	53.48
RPMI-8226	20.71	18.86	97.42	41.20	84.15
SR	7.03	8.48	23.87	18.46	16.10
Non-Small Cell Lung Cancer					
A549/ATCC	18.6	12.80	77.65	49.14	78.38
EKVX	39.79	30.23	87.78	59.13	82.69
HOP-62	32.17	29.46	*79.46*	29.82	69.72
HOP-92	43.92	25.77	*85.48*	44.15	70.59
NCI-H226	73.39	68.70	87.61	55.46	85.40
NCI-H23	39.85	38.92	85.27	59.28	88.53
NCI-H332M	51.45	48.23	87.51	47.90	65.07
NCI-H460	9.82	6.11	84.31	19.38	74.38
NCI-H552	12.78	12.12	58.47	19.85	52.38
Colon Cancer					
COLO 205	-1.65 [c]	-9.99 [c]	71.23	16.14	61.78
HCT-2998	25.25	24.97	89.14	79.59	91.92
HCT-116	7.25	6.88	62.27	29.08	56.66
HCT-15	10.04	7.70	52.67	20.63	47.63
HT29	5.30	5.94	40.59	7.93	28.31
KM12	21.79	17.05	43.91	30.23	40.62
SW-620	29.08	28.61	47.76	14.47	43.57
CNS Cancer					
SF-268	48.75	39.64	84.26	47.22	86.44
SF295	2.56	-3.02 [c]	69.71	18.75	68.71
SF539	9.34	9.52	95.69	27.71	81.24
SNB-19	33.31	30.51	90.50	30.43	69.95
SNB-75	48.64	56.72	84.06	26.61	92.44
U251	15.32	15.52	77.78	26.12	62.75
Prostate cancer					
PC-3	21.20	20.69	63.89	25.08	59.28
DU-145	19.64	12.91	103.12	64.18	102.00
Melanoma					
LOX IMV1	43.48	25.65	72.96	36.34	67.02
MALME-3M	70.24	66.57	85.32	55.25	74.83
M14	-1.61 [c]	0.91	60.08	23.27	52.83
MDA-MB-435	-46.16	-29.47 [c]	-21.80 [c]	-21.79	-32.20 [c]
SK-MEL-2	55.94	43.54	55.67	22.02	27.38
SK-MEL-28	57.04	62.95	80.08	47.22	78.33
SK-MEL-5	14.60	-12.88 [c]	65.20	29.49	52.63
UACC-257	44.45	37.75	74.57	54.88	84.23
UACC-62	32.04	36.48	57.35	18.17	49.44

Ovarian Cancer					
IGROV1	49.22	36.42 [c]	82.87	47.13	62.96
OVCAR-3	1.74	-9.52	66.63	8.29	68.96
OVCAR-4	72.08	73.56	101.25	69.25	97.02
OVCAR-5	72.98	71.11	92.64	69.57	84.69
OVCAR-8	13.43	10.28	95.58	50.11	92.58
NCI/ADR-RES	1.80	12.06	69.39	23.95	61.59
SK-OV-3	20.25	21.78	77.46	16.68	66.24
Renal Cancer					
786-0	28.10	19.99	93.40	38.84	82.57
A498	9.66	8.04	31.23	7.32	51.90
ACHN	52.36	42.41	103.18	50.89	92.77
CAKI-1	40.43	41.26	77.46	29.22	66.73
RXF 393	-13.85 [c]	-5.38 [c]	103.13	25.99	72.30
SN12C	39.32	37.55	108.47	54.32	88.36
TK-10	61.31	52.86	88.29	57.56	84.76
UO-31	57.96	61.96	75.13	48.76	68.80
Breast Cancer					
MCF-7	17.87	18.38	64.09	23.57	47.67
MDA-MB-231/ATCC	43.15	34.75	80.82	53.09	71.05
HS 578T	31.34	29.10	80.45	16.61	59.07
BT-549	11.30	-0.07 [c]	81.58	49.73	92.41
T-47D	34.98	42.62	89.77	33.59	75.11
MDA-MB-468	7.88	15.64	54.75	9.32	52.99
Mean Growth percentage	26.24	23.38	73.14	34.17	65.45

[a]Data obtained from NCI in vitro 60 cell line human tumour cell screen at 10 μM concentration. [b]Mean growth percentage; [c] negative values indicate cytotoxicity/cell death [d] Nd: Not determined; [e] NSC 788805; [f] NSC 788806; [g] NSC 803351; [h] NSC 803350; [i] NSC 803353.

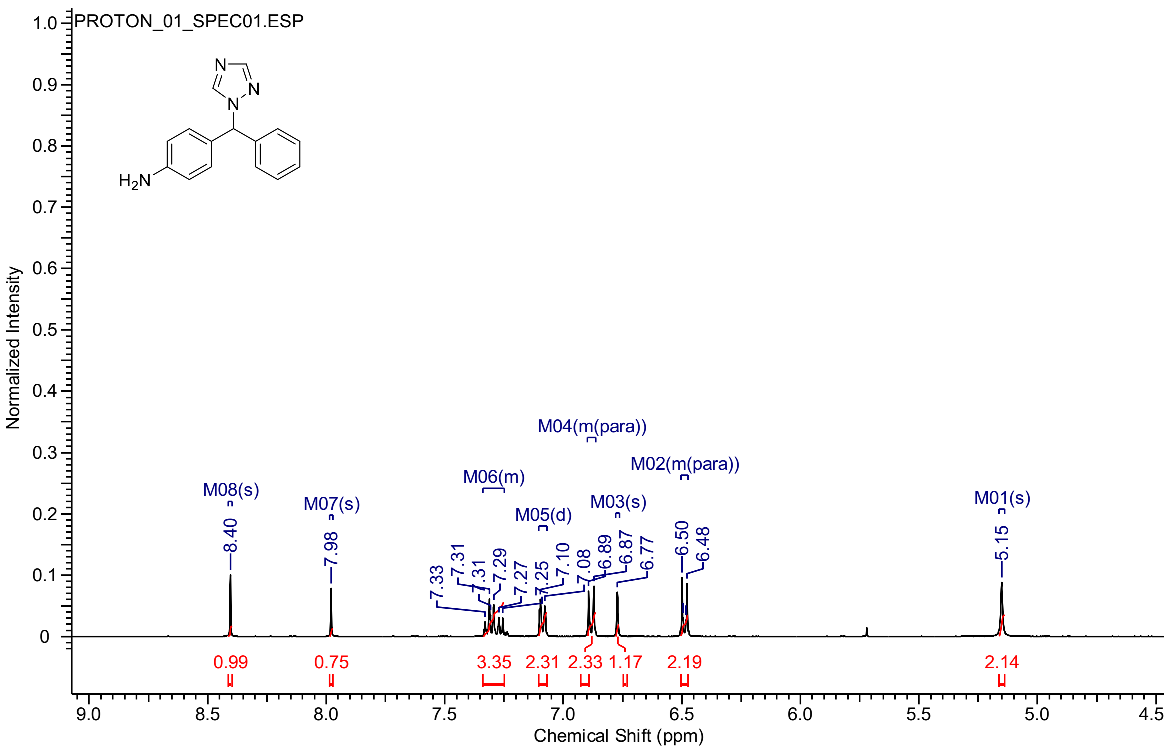

Figure S1: ^{1}H-NMR spectrum of compound **13m** (DMSO-d$_6$)

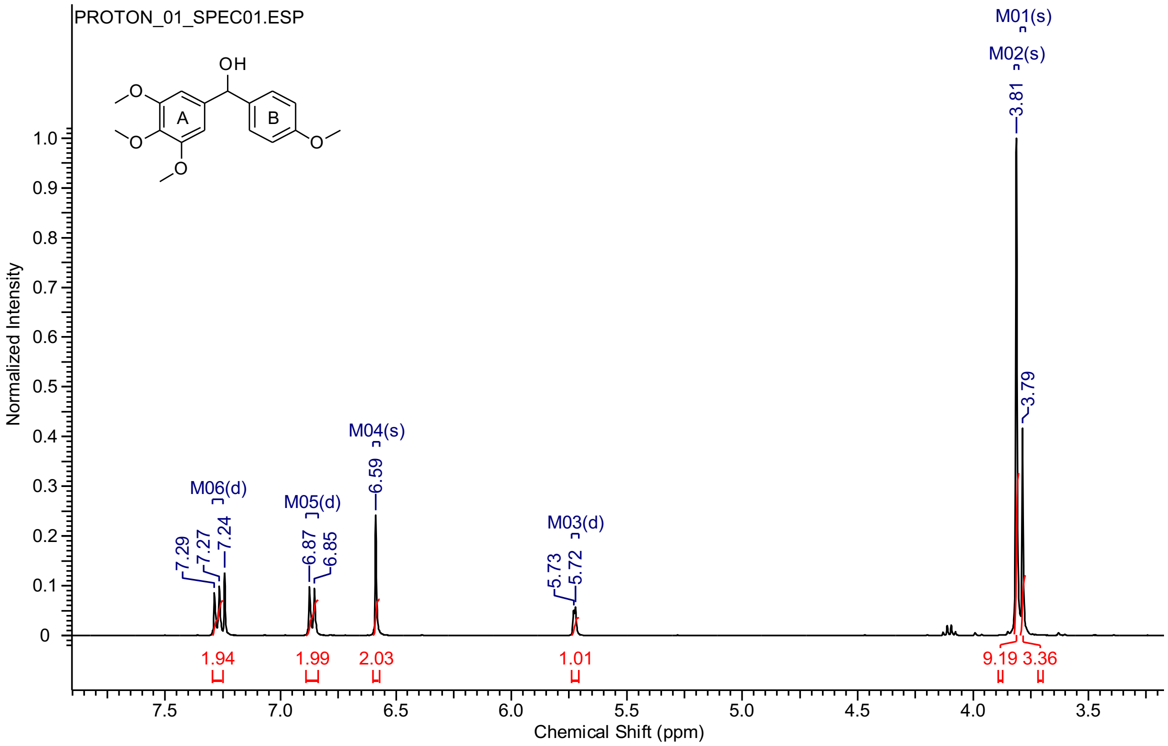

Figure S2: ^{1}H-NMR spectrum of compound **15c** ($CDCl_3$)

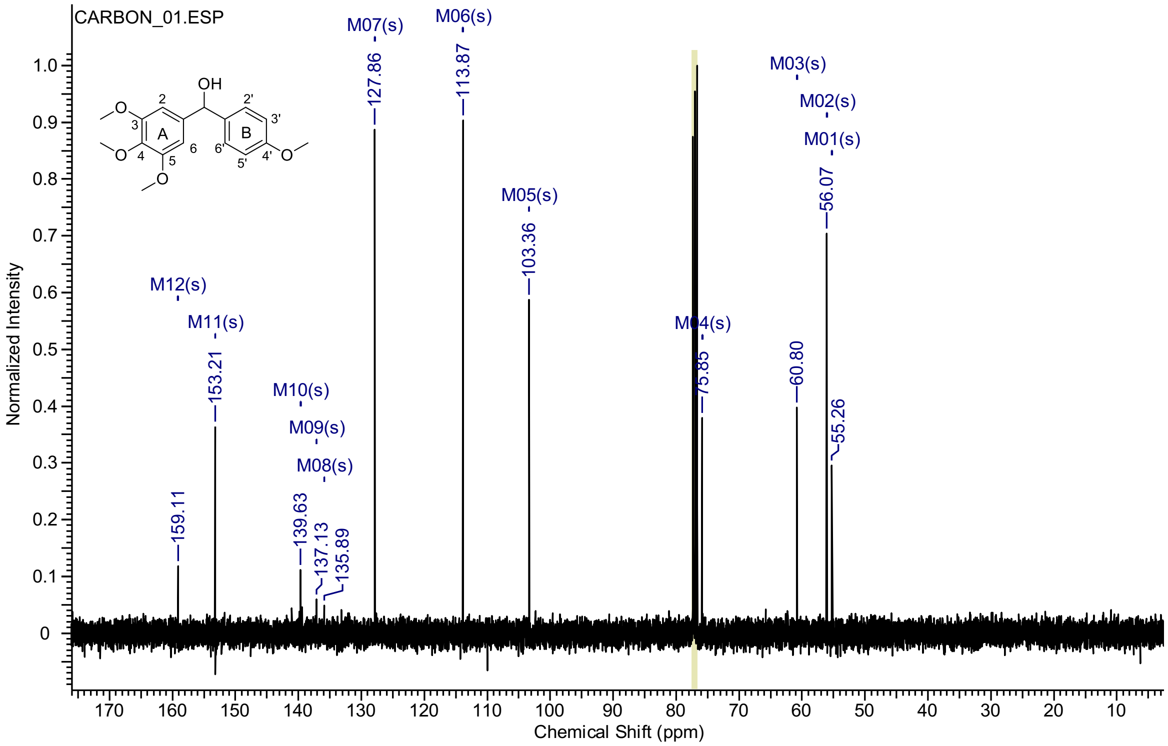

Figure S3: ^{13}C-NMR spectrum of compound **15c** ($CDCl_3$)

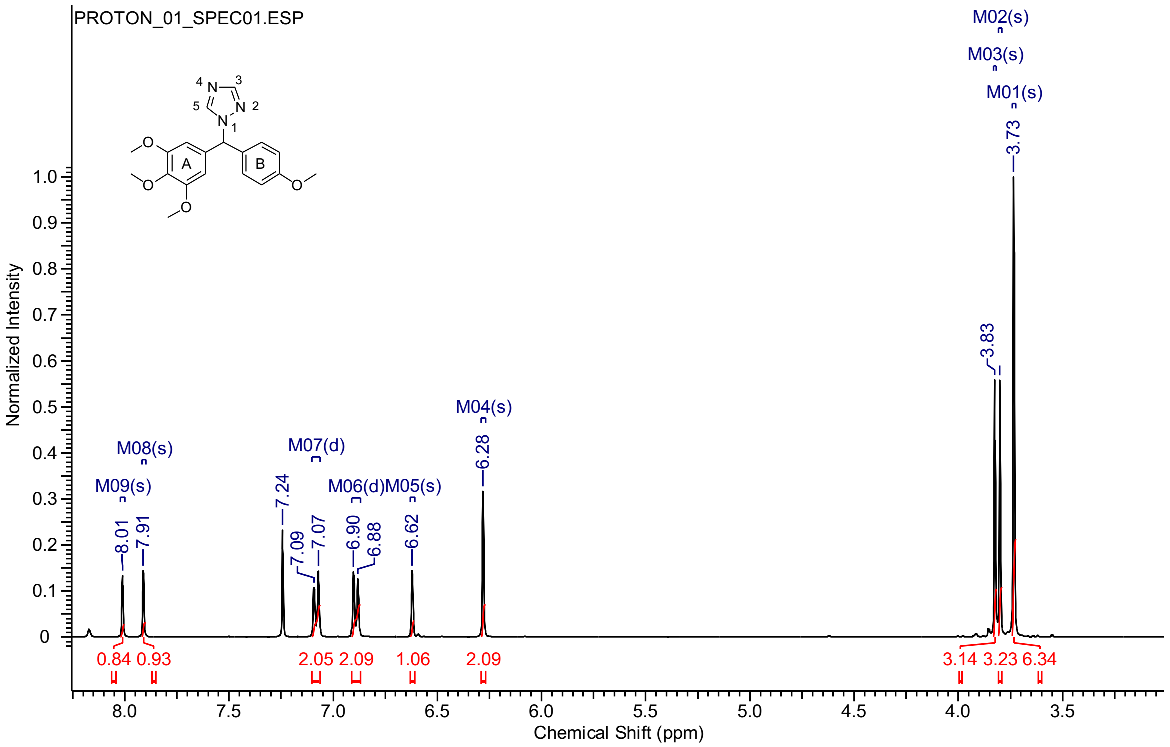

Figure S4: ^{1}H-NMR spectrum of compound **16c** ($CDCl_3$)

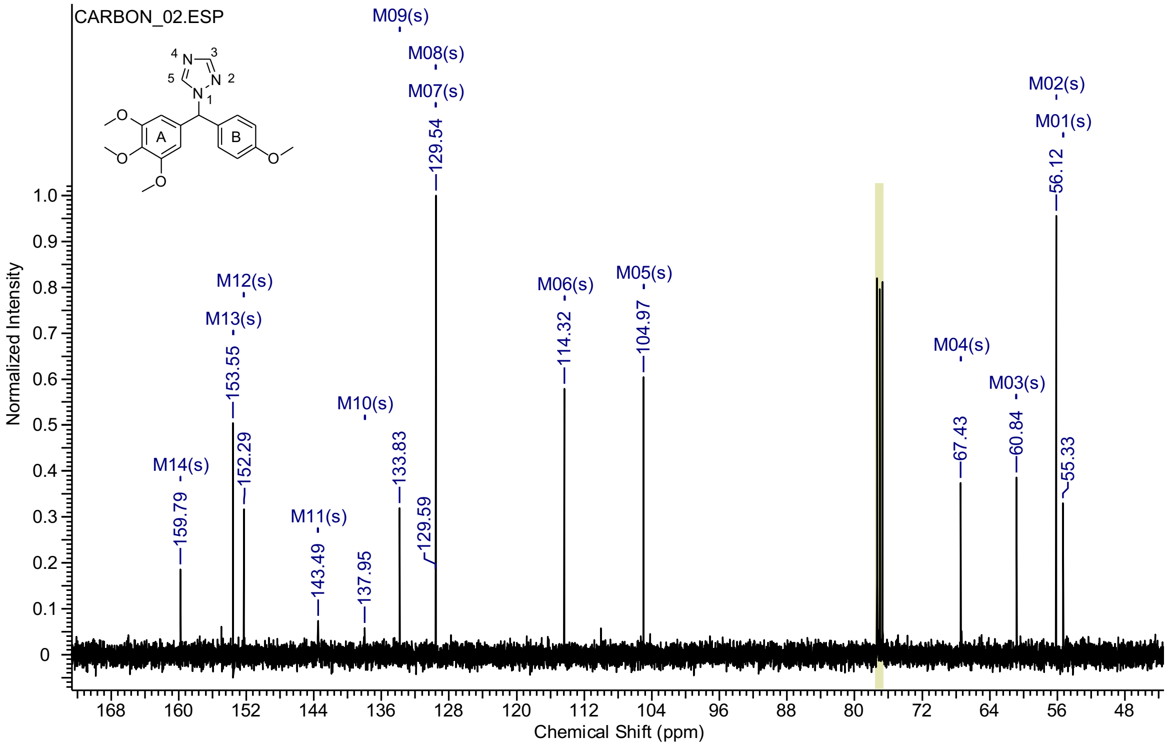

Figure S5: ^{13}C-NMR spectrum of compound **16c** ($CDCl_3$)

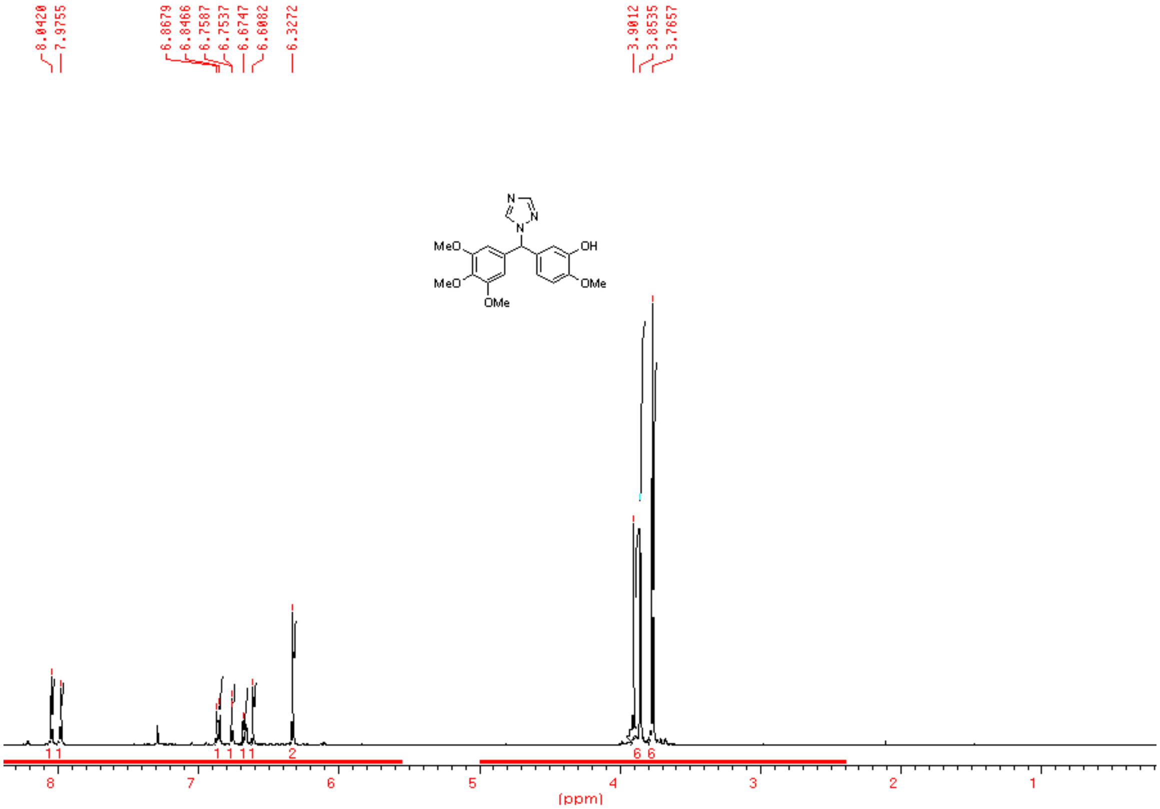

Figure S6: ^{1}H-NMR spectrum of compound **19e** ($CDCl_3$)

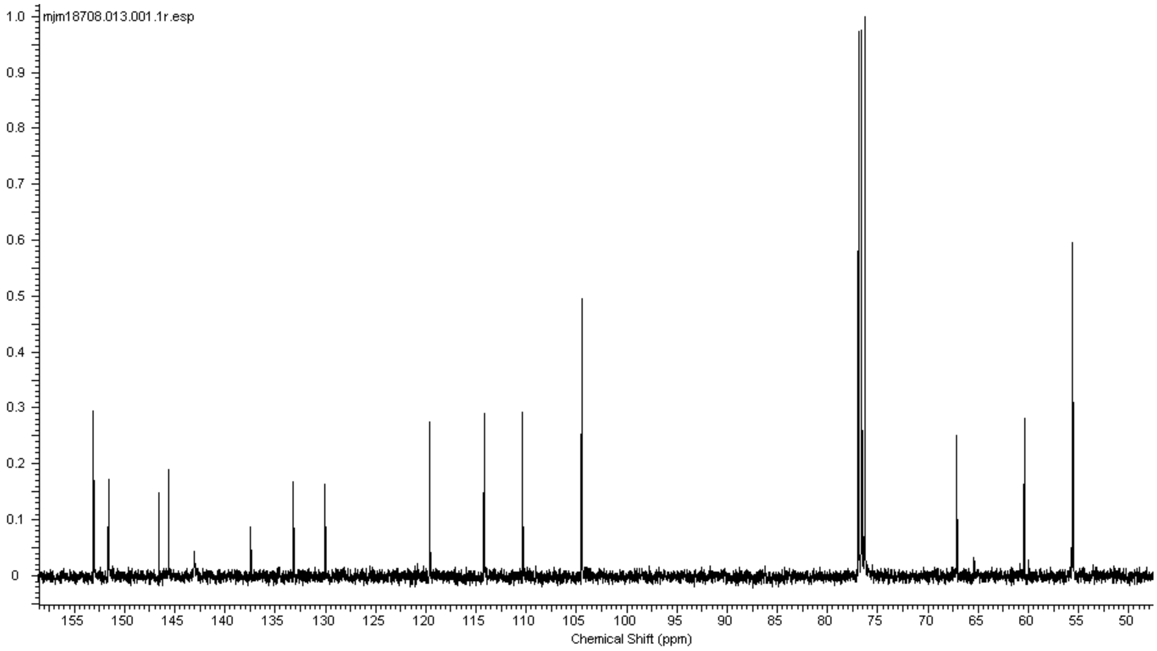

Figure S7: ^{13}C-NMR spectrum of compound **19e** ($CDCl_3$)

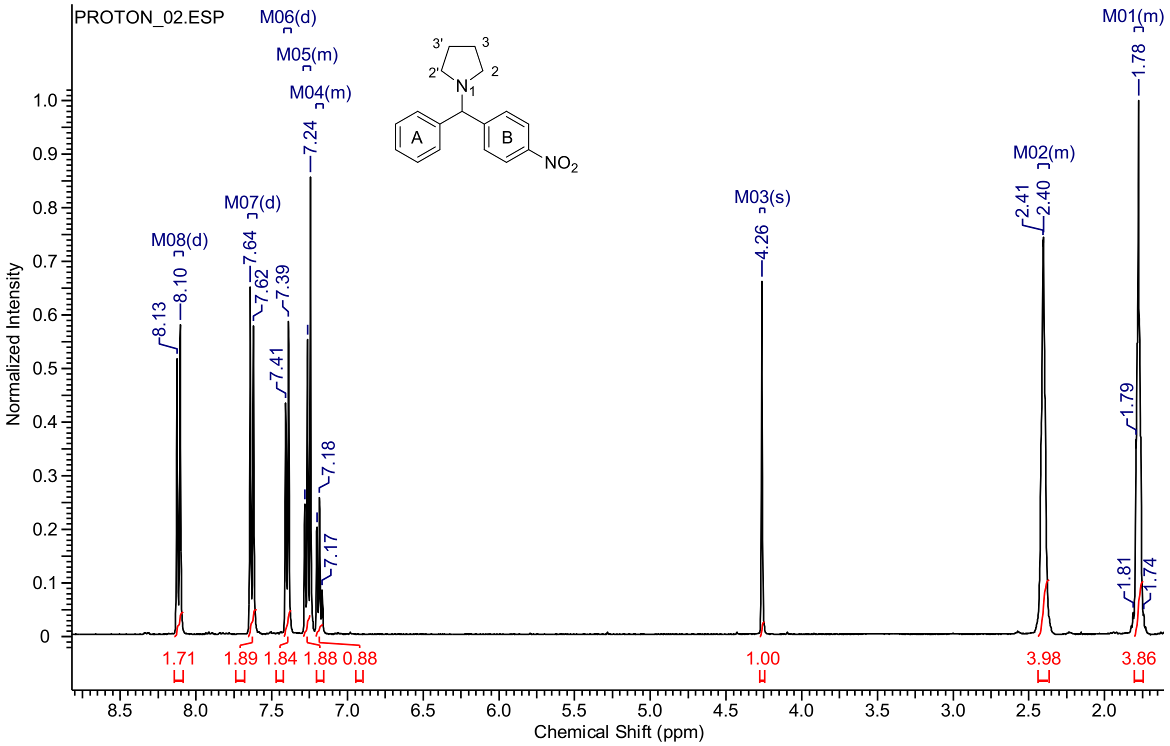

Figure S8: ^{1}H-NMR spectrum of compound **25a** ($CDCl_3$)

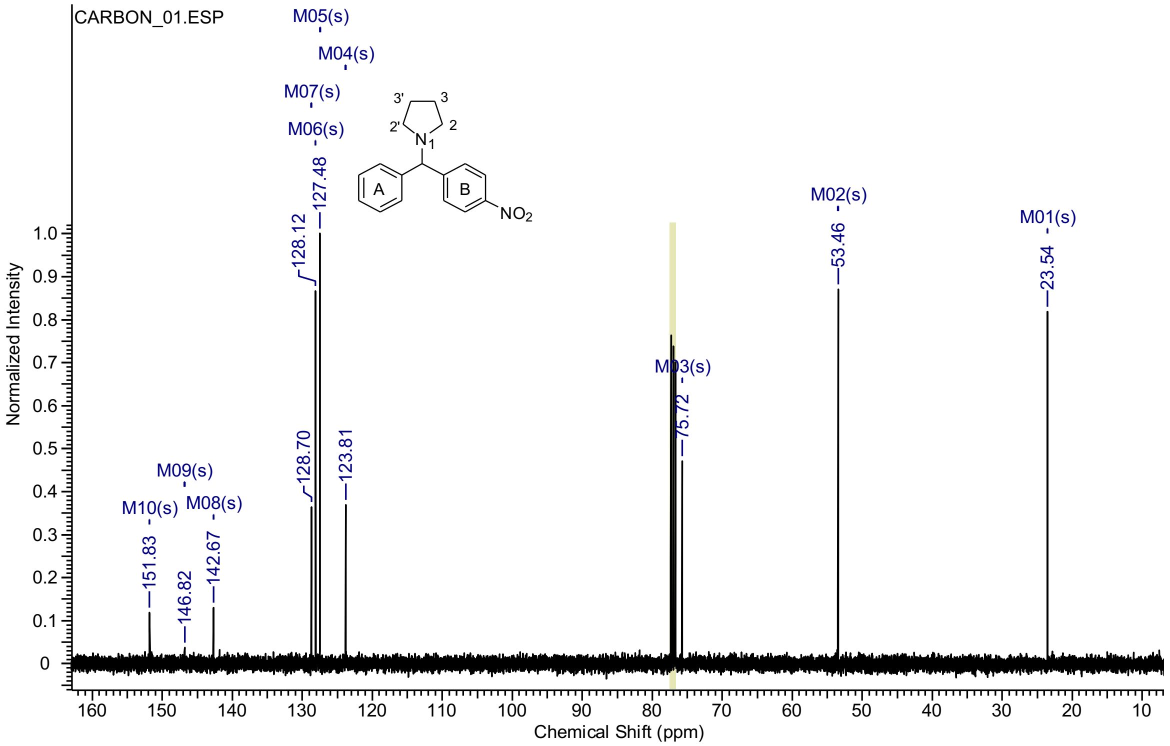

Figure S9: ^{13}C-NMR spectrum of compound **25a** ($CDCl_3$)

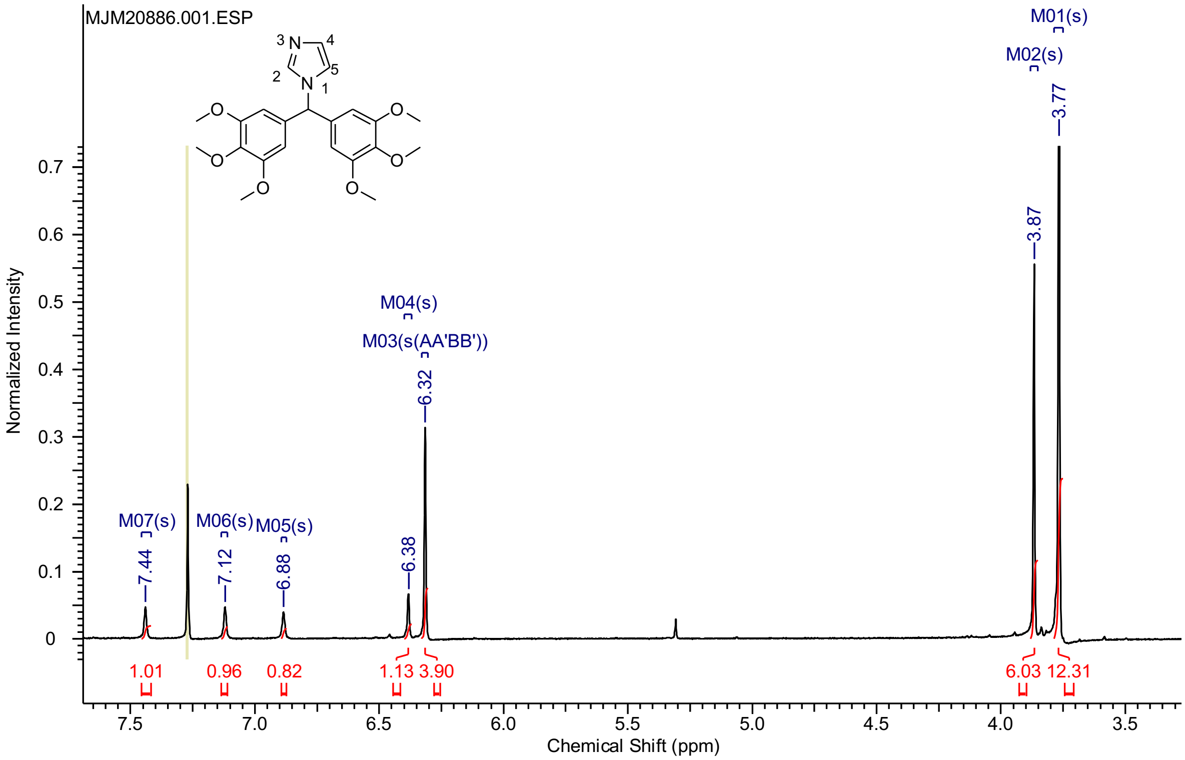

Figure S10: ^{1}H-NMR spectrum of compound **21i** ($CDCl_3$)

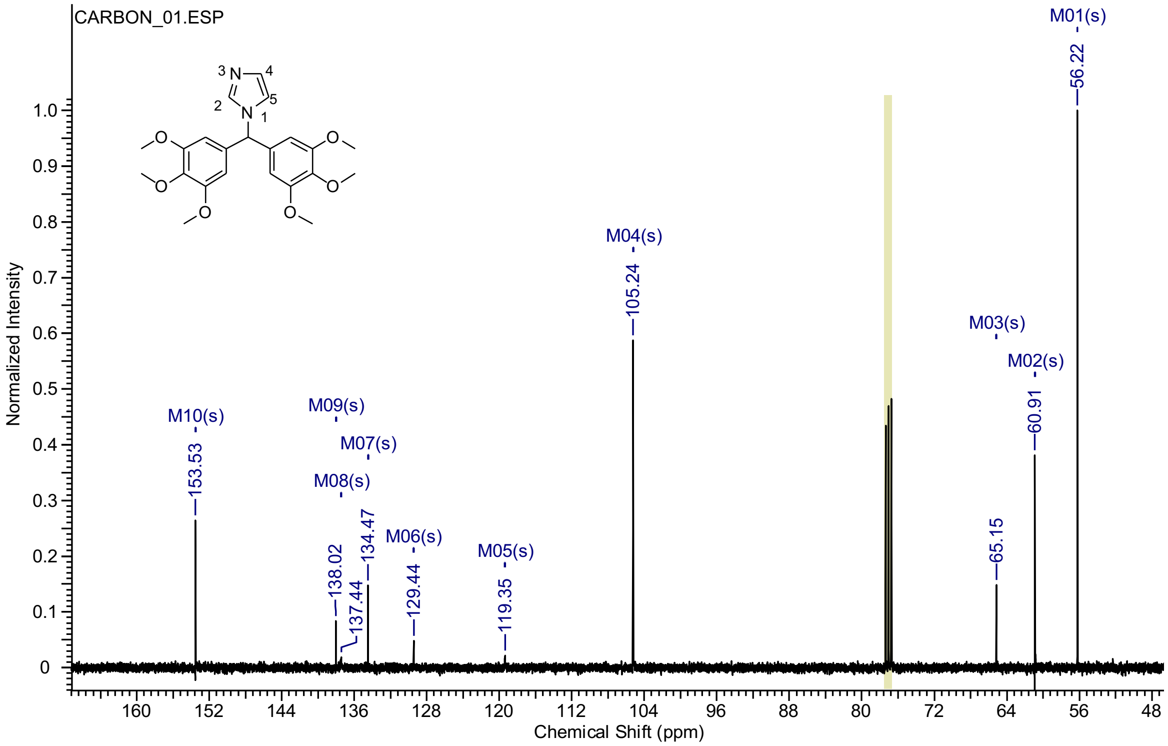

Figure S11: ^{13}C-NMR spectrum of compound **21i** ($CDCl_3$)

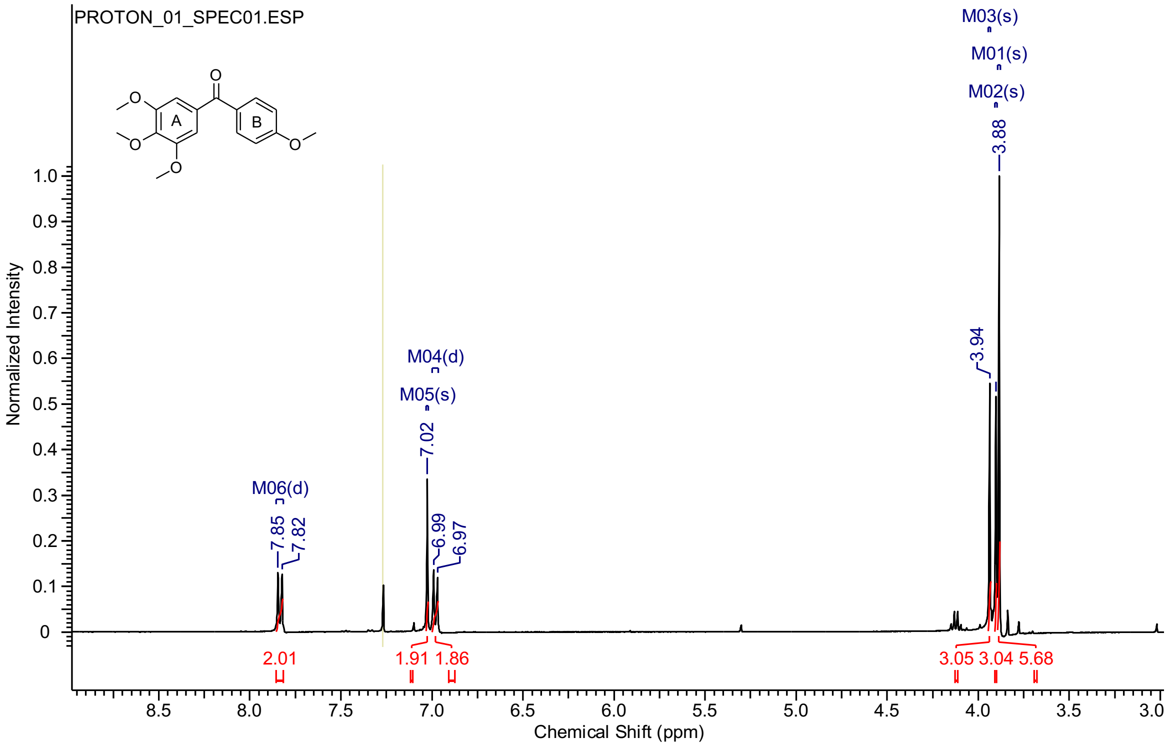

Figure S12: ^{1}H-NMR spectrum of compound **23a** ($CDCl_3$)

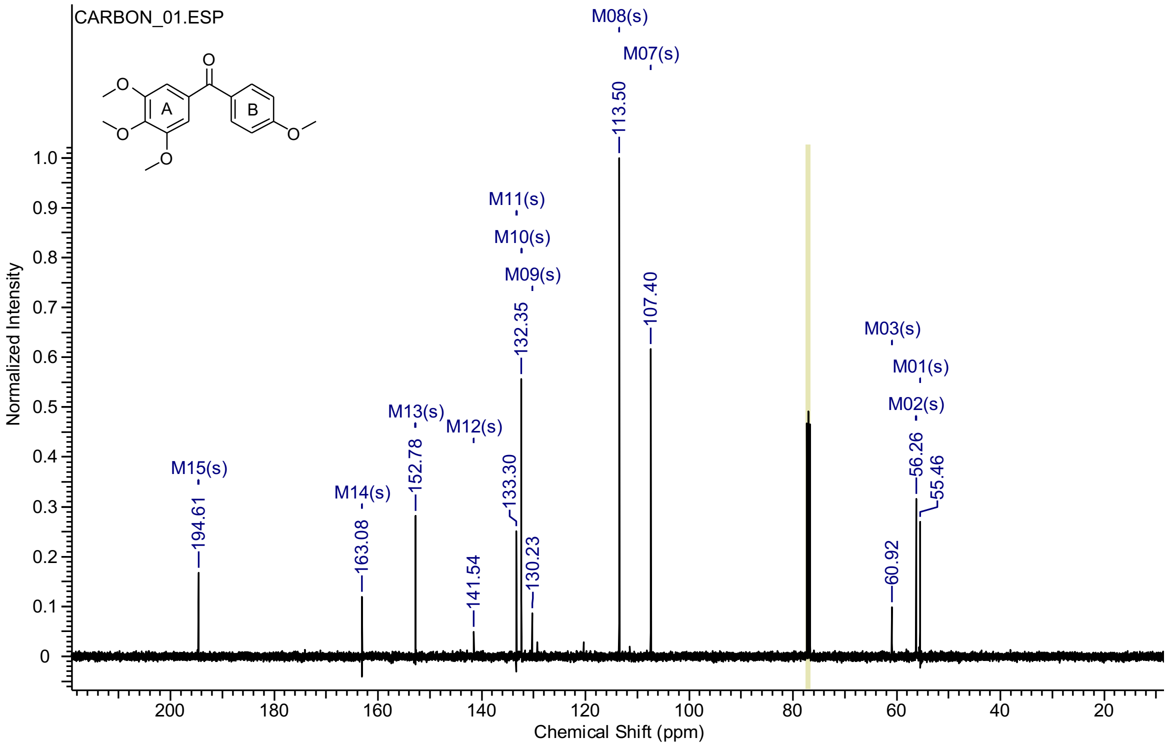

Figure S13: ^{13}C-NMR spectrum of compound **23a** ($CDCl_3$)

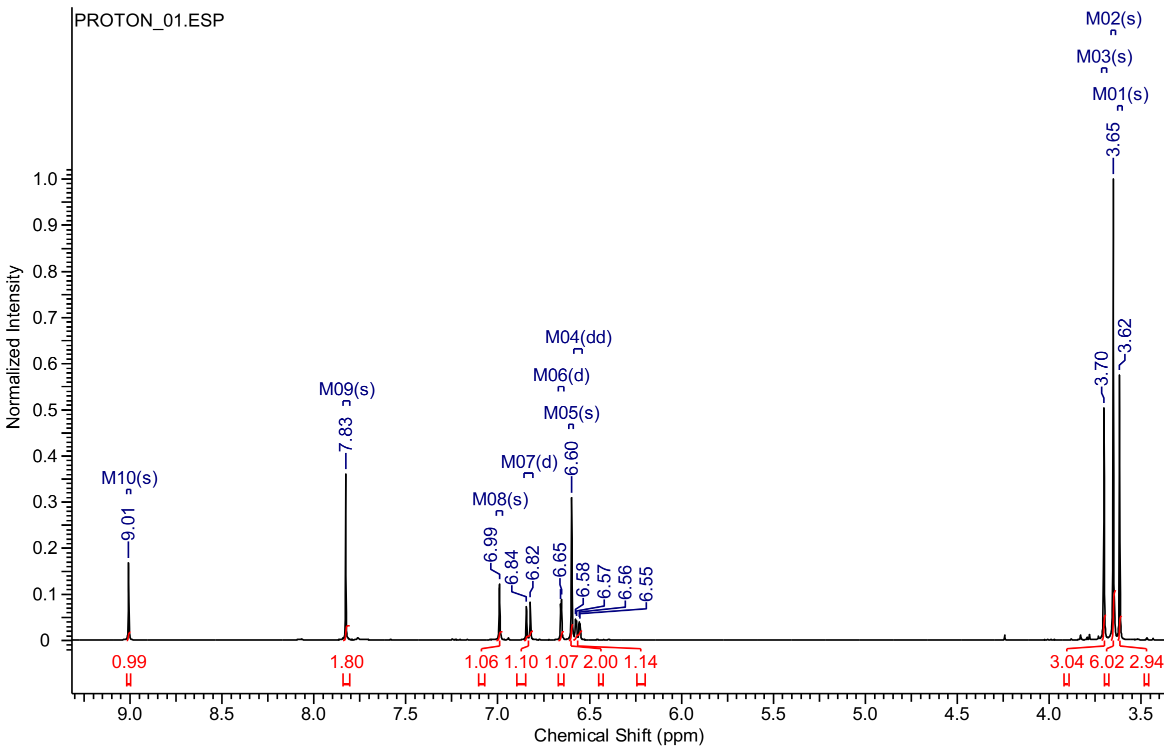

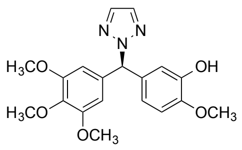

Figure S14: ^{1}H-NMR spectrum of compound **24** (DMSO-d_6)

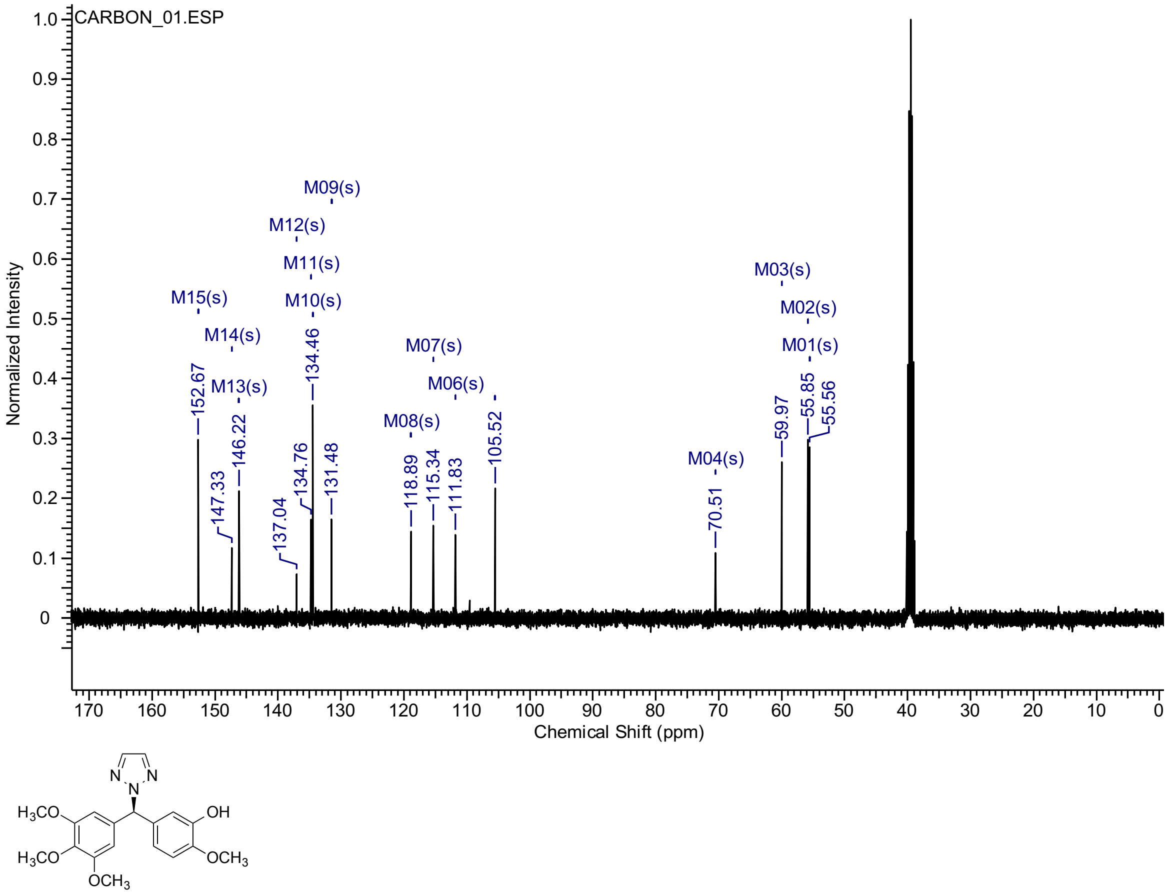

Figure S15: ^{13}C-NMR spectrum of compound **24** (DMSO-d$_6$)

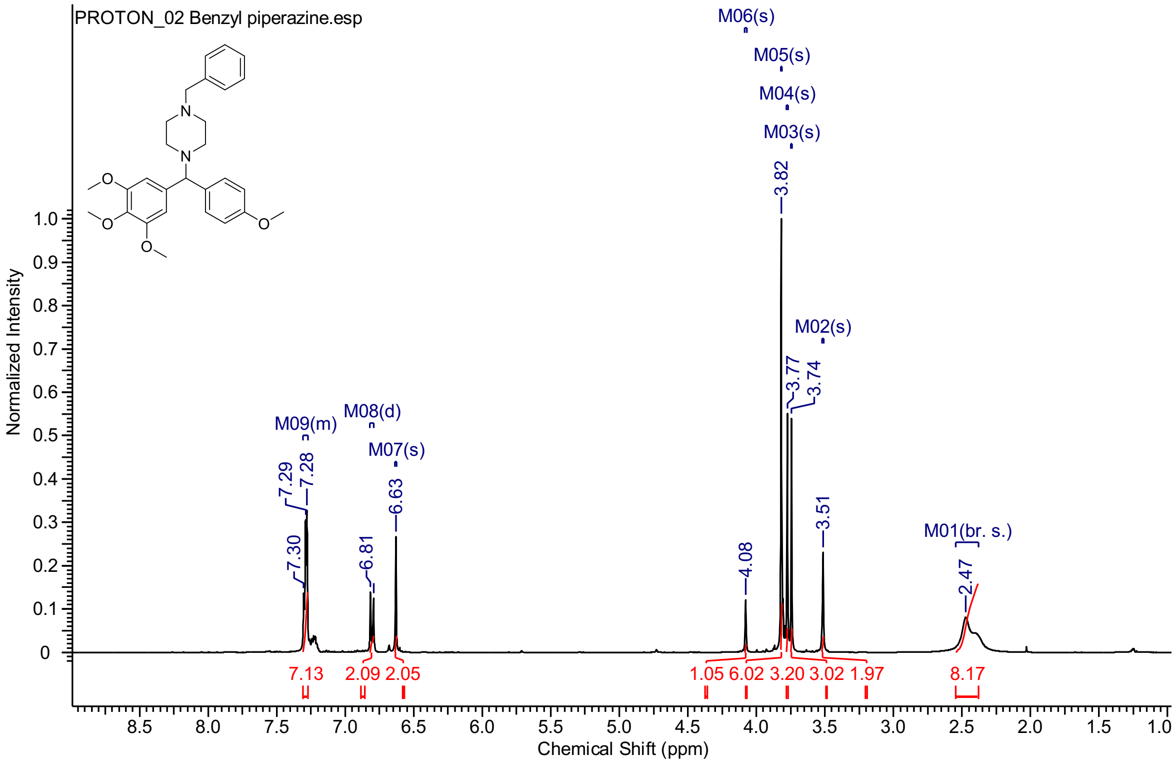

Figure S16: ^{1}H-NMR spectrum of compound **27d** ($CDCl_3$)

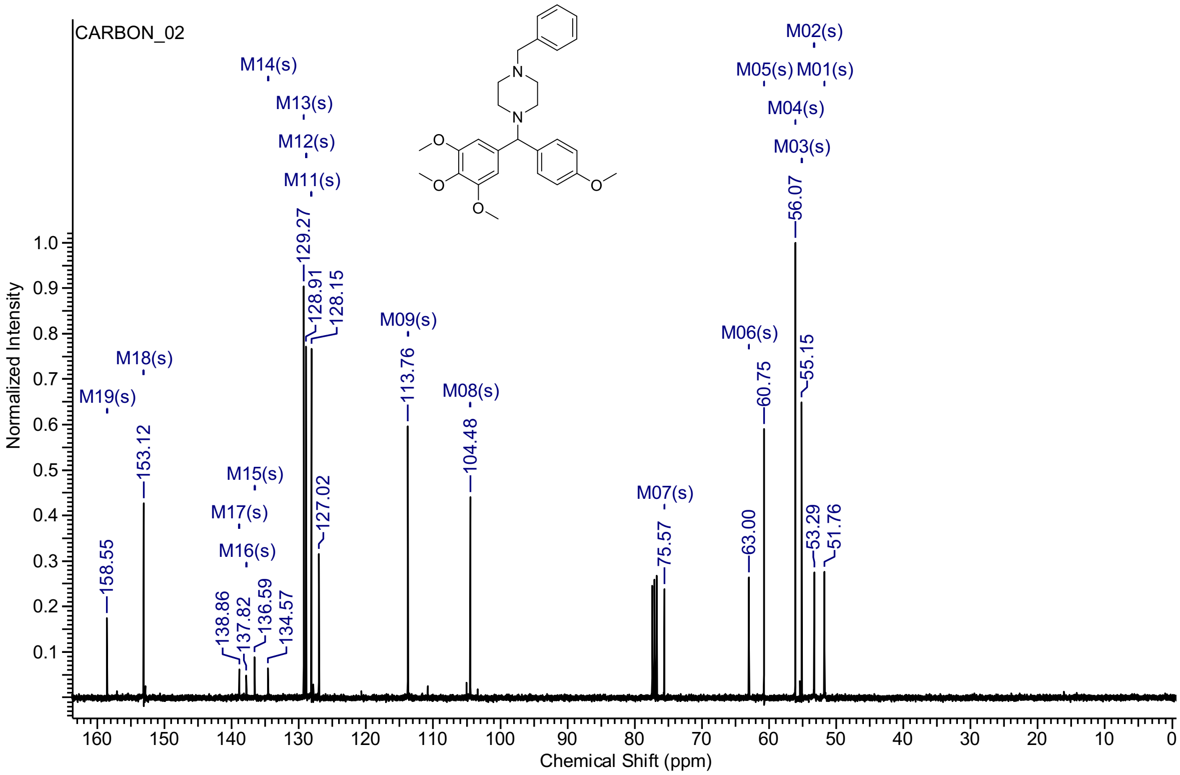

Figure S17: ^{13}C-NMR spectrum of compound **27d** ($CDCl_3$)

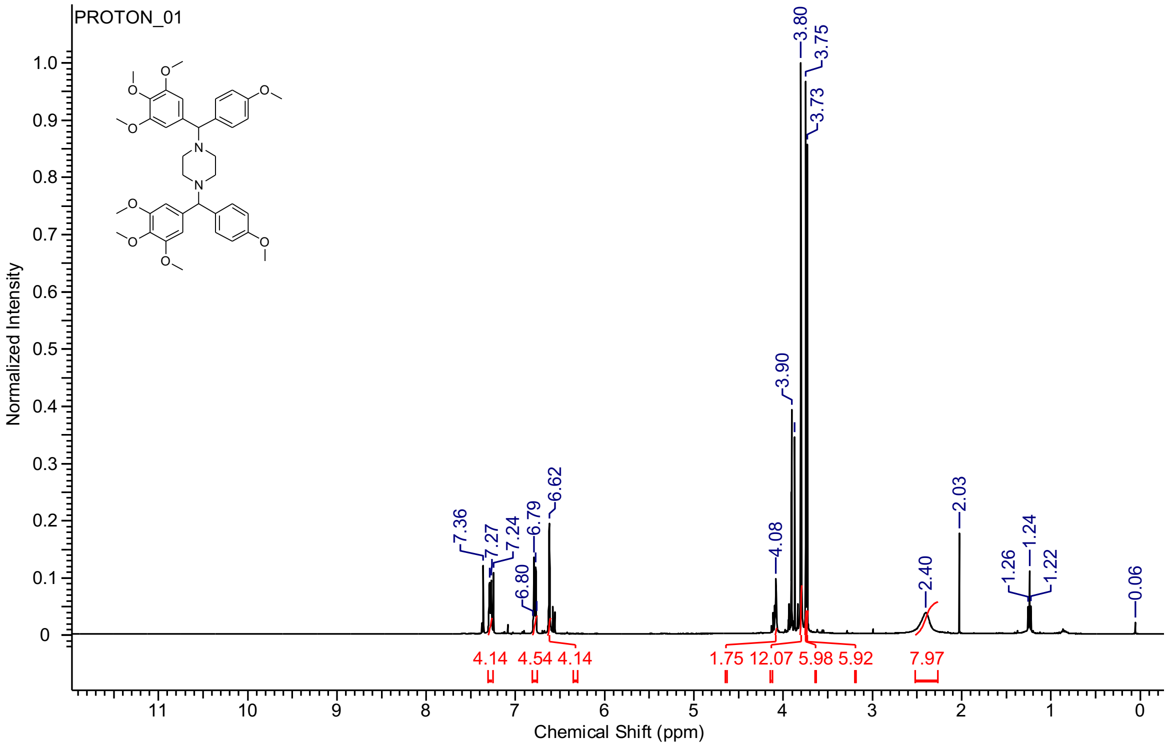

Figure S18: ^{1}H-NMR spectrum of compound **28** ($CDCl_3$)

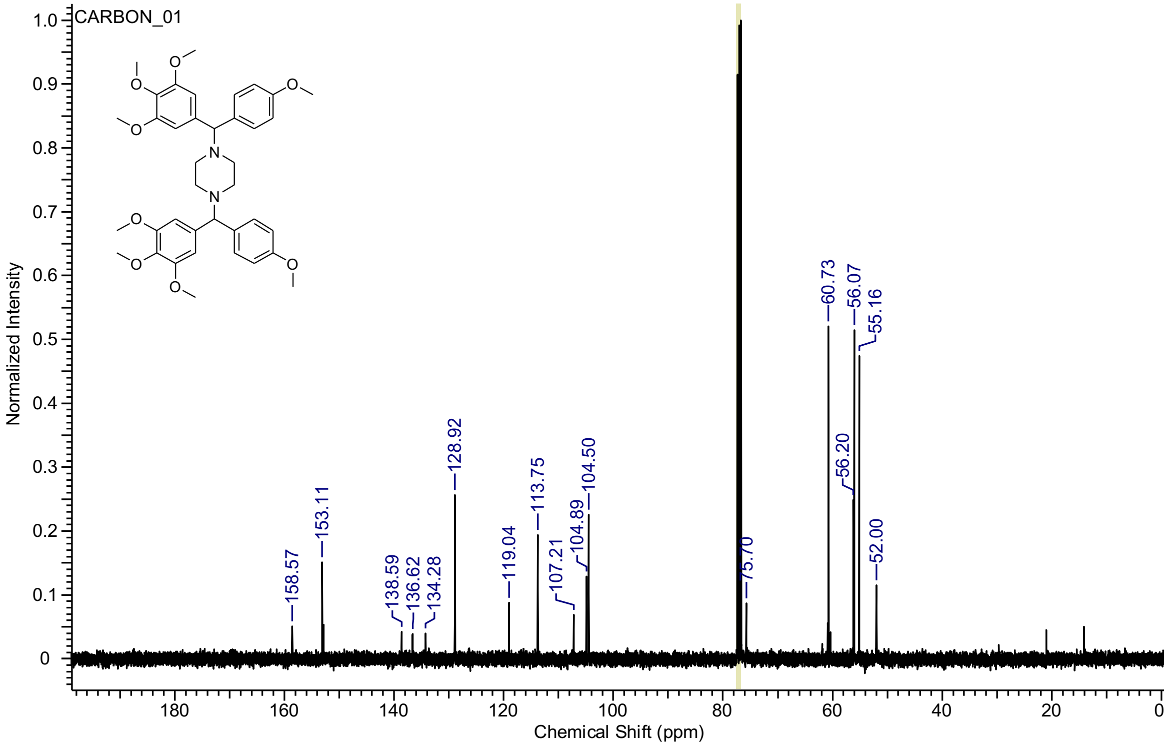

Figure S19: ^{13}C-NMR spectrum of compound **28** ($CDCl_3$)

Experimental chemistry

All reagents were commercially available and were used without further purification unless otherwise indicated. Anhydrous solvents were purchased from Sigma. Uncorrected melting points were measured on a Gallenkamp apparatus. Infra-red (IR) spectra were recorded on a Perkin Elmer FT-IR Paragon 1000 spectrometer. ^{1}H and ^{13}C nuclear magnetic resonance spectra (NMR) were recorded at 27 °C on a Brucker DPX 400 spectrometer (400.13 MHz, ^{1}H; 100.61 MHz, ^{13}C) in $CDCl_3$ (internal standard tetramethylsilane (TMS)). For $CDCl_3$, ^{1}H NMR spectra were assigned relative to the TMS peak at 0.00 ppm and ^{13}C NMR spectra were assigned relative to the middle $CDCl_3$ peak at 77.0 ppm. Electrospray ionisation mass spectrometry (ESI-MS) was performed in the positive ion mode on a liquid chromatography time-of-flight mass spectrometer (Micromass LCT, Waters Ltd., Manchester, UK). The samples were introduced to the ion source by an LC system (Waters Alliance 2795, Waters Corporation, USA) in acetonitrile: water (60:40 % v/v) at 200 µL/min. The capillary voltage of the mass spectrometer was at 3 kV. The sample cone (de-clustering) voltage was set at 40 V. For exact mass determination, the instrument was externally calibrated for the mass range m/z 100 to m/z 1000. A lock (reference) mass (m/z 556.2771) was used. Mass measurement accuracies of $< \pm 5$ ppm were obtained. TLC was performed using Merck Silica gel 60 TLC aluminium sheets with fluorescent indicator visualizing with UV light at 254 nm. Flash chromatography was carried out using standard silica gel 60 (230-400 mesh) obtained from Merck. All products isolated were homogenous on TLC. The purity of the tested compounds was determined by HPLC. Analytical high-performance liquid chromatography (HPLC) was performed using a Waters 2487 Dual Wavelength Absorbance detector, a Waters 1525 binary HPLC pump and a Waters 717 plus Autosampler. The column used was a Varian Pursuit XRs C18 reverse phase 150 × 4.6 mm chromatography column. Samples were detected using a wavelength of 254 nm. All samples were analyzed using acetonitrile (60%): water (40%) over 10 min and a flow rate of 1 mL/min. Microwave experiments were carried using a Biotage Discover CEM microwave synthesiser on standard power setting (maximum power supplied is 300 watts) unless otherwise stated.

4-Benzoylphenyl acetate (11h): To a solution of 4-hydroxybenzophenone (1 eq, 5.04 mmol, 1 g) in dry DCM (50 mL), was added trimethylamine (1 eq, 5.04 mmol, 0.70 mL) followed by addition of acetyl chloride (3 eq, 15.13 mmol, 1 mL). The reaction was allowed to stir for 3 h under a nitrogen atmosphere at room temperature. The organic phase was then washed with water, (30 mL) dried over sodium sulphate filtered and concentrated. Yield: 93% (1.13 g) white solid Mp. 81-83 °C [1]. ^{1}H NMR (400 MHz, $CDCl_3$) δ 2.32 (s, 3 H, CH_3), 7.20 (d, *J*=8.5 Hz, 2 H, Ar-H), 7.45-7.49 (m, 2 H, Ar-H), 7.56-7.59 (m, 1 H, Ar-H), 7.78 (d, *J*=7.32 Hz, 2 H, Ar-H), 7.84 (d, *J*=8.5 Hz, 2 H, Ar-H). ^{13}C NMR (101 MHz, $CDCl_3$) δ 21.15 (CH_3), 121.49 (2xCH), 128.30 (2xCH), 129.92 (4xCH), 131.64 (CH), 132.44 (C), 137.46 (C), 153.85 (C-O), 168.87 (C=O), 195.50 (C=O). LRMS (APCI): found 241.20 $(M+H)^+$; $C_{15}H_{13}O_3$ requires 241.10. IR: ν_{max} (ATR) cm^{-1}: 3285, 2990, 2959, 2929, 1749, 1647, 1595, 1578, 1410, 1365, 1281, 1212, 1195, 1161, 1146, 1014, 941, 918, 854, 789, 704, 655, 593.

***N*-(4-Benzoylphenyl)acetamide (11i):** 4-aminobenzophenone (1 eq, 2.5 mmol, 0.5 g) was dissolved in dry DCM (20 mL), trimethylamine (1 eq, 2.5 mmol, 0.25 g, 0.35 mL) was added followed by acetyl chloride (3 eq, 7.6 mmol, 0.6 g, 0.54 mL). The reaction was stirred at room temperature under nitrogen for 1 h. After 1 h the solution was washed with water (10 mL), brine (10 mL) and dried over sodium sulphate. The crude product was purified *via* flash column chromatography (eluent: *n*-hexane/ethyl acetate 1:1). Yield: 87% (0.52 g) white solid Mp. 151-152 °C [2]. ^{1}H NMR (400 MHz, $CDCl_3$) δ 7.79 (d, *J* = 8.5 Hz, 2H, Ar-H), 7.75 (d, *J* = 7.6 Hz, 2H, Ar-H), 7.62 (d, *J* = 8.3 Hz, 2H, Ar-H), 7.55 (d, *J* = 7.4 Hz, 1H, Ar-H), 7.44 - 7.48 (m, 2H, Ar-H), 2.20 (s, 3H, CH_3). ^{13}C NMR (101 MHz, $CDCl_3$) δ 195.70 (C=O), 168.61 (NH-C=O), 141.87 (C), 137.76 (C), 132.94 (C), 132.23 (CH), 131.58 (2xCH), 129.83 (2xCH), 128.25 (2xCH), 118.72 (2xCH), 24.74 (CH_3). LRMS (EI): found 240.19 $(M+H)^+$; $C_{15}H_{14}NO_2$ requires 240.10. IR: ν_{max} (ATR) cm^{-1}: 3341, 1702, 1600, 1586, 1523, 1462, 1371, 1343, 1284, 1253, 1172, 1040, 927, 842, 744, 736, 690, 652.

***N*-(4-Benzoylphenyl)-2,2,2-trifluoroacetamide (11j):** To a solution of **8** (1 eq, 2.5 mmol, 0.5 g) in dry DCM (50 mL) under nitrogen atmosphere, triethylamine (1 eq, 2.5 mmol, 0.35 mL) was added, followed by trifluoroacetic anhydride (2 eq, 5 mmol, 0.7

mL). The reaction mixture was allowed to stir for 15 min over ice and for another 2 h at room temperature. The solution was then washed with HCl 10% (3x20 mL), $NaHCO_3$ (2x20 mL), water (20 mL) and brine (20 mL). The organic phase was dried over sodium sulphate, filtered and concentrated. No further purification was required. Yield: 94% (0.69 g) pale yellow solid, Mp. 135-137 °C. IR: ν_{max} (ATR) cm^{-1}: 3538, 3280, 3201, 1738, 1640, 1598, 1541, 1413, 1281, 1248, 1208, 1175, 1140. ^{1}H NMR (400 MHz, DMSO-*d*$_6$) δ 7.51 - 7.56 (m, 2 H, Ar-H), 7.62 - 7.67 (m, 1 H, Ar-H), 7.68 - 7.72 (m, 2 H, Ar-H), 7.76 - 7.80 (m, 2 H, Ar-H), 7.83 - 7.87 (m, 2 H, Ar-H), 11.56 (s, 1 H, NH). ^{13}C NMR (101 MHz, DMSO-*d*$_6$) δ 117.03 (CF_3), 120.43 (2xCH), 128.55 (2xCH), 129.49 (2xCH), 130.92 (2xCH), 132.57 (CH), 133.67 (C), 137.07 (C), 140.25 (C), 154.59 (C=OCF_3), 194.63 (C=O). HRMS (EI): found 292.0581 $(M-H)^+$ $C_{15}H_9F_3NO_2$ requires 292.0586.

(4-(Benzyloxy)phenyl)(phenyl)methanone (11k): To a solution of 4-hydroxybenzophenone (1 eq, 5.78 mmol, 1 g) in ACN (50 mL), K_2CO_3 (1.3 eq, 7.5 mmol, 1.03 g) was added while stirring followed by the dropwise addition of benzyl bromide (1 eq, 5.78 mmol, 0.68 mL). The mixture was stirred for 6 h under nitrogen atmosphere at reflux. After 6 h the mixture was filtered and concentrated giving the desired final product. Yield: 90% (1.89 g) white crystals, Mp. 85-87 °C [3]. ^{1}H NMR (400 MHz, $CDCl_3$) δ 5.14 (s, 2 H, CH_2), 7.02 (d, *J*=8.54 Hz, 2 H, Ar-H), 7.32 - 7.48 (m, 7 H, Ar-H), 7.52 - 7.58 (m, 1 H, Ar-H), 7.74 (d, *J*=7.32 Hz, 2 H, Ar-H), 7.81 (d, *J*=9.16 Hz, 2 H, Ar-H). ^{13}C NMR (101 MHz, $CDCl_3$) δ 70.16 (CH_2), 114.39 (2xCH), 127.47 (2xCH), 128.16 (2xCH), 128.23 (CH), 128.69 (2xCH), 129.71 (2xCH), 130.36 (C), 131.87 (2xCH), 132.54 (CH), 136.20 (C), 138.23 (C), 162.34 (C-O), 195.49 (C=O). HRMS (EI): found 311.1028 $(M+Na)^+$ $C_{20}H_{16}NaO_2$ requires 311.1048. IR: ν_{max} (ATR) cm^{-1}: 3099, 3039, 2863, 1638, 1599, 1575, 1416, 1317, 1305, 1242, 1149, 1000, 938, 921, 842, 795, 742, 705, 690, 639.

Bis(4-((*tert*-butyldimethylsilyl)oxy)phenyl)methanone (11l): A solution of 4,4’-dihydroxy benzophenone (1 eq, 4.67 mmol, 1 g) in dry DCM (50 mL) was reacted with tert-butyldimethylsilyl chloride (2 eq, 9.34 mmol, 1.40 g) and DBU (2.5 eq, 11.675 mmol, 1.77 g 1.74 mL) and stirred at RT for 4 h. After 4 h the solution was washed with

HCl (0.1 M, 30 mL), $NaHCO_3$ sat. (30 mL) and water (20 mL). The organic phase was then dried over sodium sulphate The crude product was purified via flash chromatography (eluent: *n*-hexane/ethyl acetate 8:2). Yield: 78% (1.60 g) white solid, Mp. 214-216 °C [4]. ^{1}H NMR (400 MHz, $CDCl_3$) δ 0.24 - 0.27 (m, 12 H, CH_3), 1.00 - 1.02 (m, 18 H, CH_3), 6.88 - 6.93 (m, 4 H, Ar-H), 7.71 - 7.75 (m, 4 H, Ar-H). ^{13}C NMR (101 MHz, $CDCl_3$) δ -4.35 (4xCH_3), 18.26 (2xC), 25.61 (6xCH_3), 119.61 (4xCH), 131.28 (2xC), 132.13 (4xCH), 159.49 (2xC-O), 194.69 (C=O). LRMS (EI): found 443.40 $(M+H)^+$ $C_{25}H_{39}O_3Si_2$ requires 443.24. IR: ν_{max} (ATR) cm^{-1}: 3323, 3120, 1627, 1584, 1570, 1445, 1316, 1267, 1239, 1162, 1151, 1106, 971, 930, 851, 814, 769, 683, 632, 580, 557.

(4-Ethoxyphenyl)(phenyl)methanone (11m): To a solution of 4-hydroxybenzophenone (1 eq, 5.0 mmol, 1 g) in DMF (50 mL) K_2CO_3 (2.5 eq, 12.6 mmol, 1.74 g) was added followed by the addition of iodoethane (2 eq, 10.0 mmol, 1.55 g, 0.80 mL) and stirred overnight. The reaction was quenched with water (25 mL) and extracted with ethyl acetate (2x50 mL). The organic phase was then washed with brine (30 mL), dried over sodium sulfate, filtered and concentrated under reduced pressure. Yield: 100% (1.13 g) pale yellow oil [5]. ^{1}H NMR (400 MHz, $CDCl_3$) δ 1.46 (t, *J*=7.05 Hz, 3 H, CH_3), 4.12 (q, *J*=6.91 Hz, 2 H, CH_2), 6.93 - 6.98 (m, 2H, Ar-H), 7.45 - 7.51 (m, 2 H, Ar-H), 7.54 - 7.60 (m, 1 H, Ar-H), 7.75 - 7.78 (m, 2 H, Ar-H), 7.81 - 7.85 (m, 2 H, Ar-H). ^{13}C NMR (101 MHz, $CDCl_3$) δ 14.67 (CH_3), 63.74 (CH_2), 113.95 (2xCH), 128.13 (2xCH), 129.67 (2xCH), 129.91 (C), 131.79 (2xCH), 132.53 (CH), 138.31 (C), 162.63 (C-O), 195.51 (C=O). HRMS (EI)**:** Found 249.0894 $(M+Na)^+$; $C_{15}H_{14}NaO_2$ requires 249.0886. **IR:** ν_{max} (ATR) cm^{-1}: 2981, 2884, 1750, 1650, 1598, 1576, 1506, 1304, 1280, 1248, 1171, 1147, 1041, 918, 791, 740, 697, 621, 606, 566.

General method A: Reduction of benzophenones

To a solution of the benzophenone in methanol (25 mL), $NaBH_4$ (2 eq) was added in small portions. The solution was stirred at 0 °C until the reaction was complete from TLC. Dilute HCl (10%) was added and the solvent was removed with the rotary evaporator. The product was then re-dissolved in ethyl acetate and washed with water and brine, dried over sodium sulphate, filtered and concentrated. No further purification is required.

Diphenylmethanol (12a): As per general general method A, benzophenone **(11a)** (1 eq, 12.34 mmol, 2.25 g) was dissolved in methanol and cooled to 0°C. Sodium borohydride (2 eq, 24.69 mmol, 0.93 g) was added in small portions and the mixture was stirred until the reaction was complete from TLC. HCl (10%) was added and the solvent was evaporated under reduced pressure. The product was re-dissolved in ethyl acetate (30 mL) and washed with water (20 mL) and brine (10 mL), dried over sodium sulphate, filtered and concentrated under reduced pressure to afford the desired product with no need of further purification. Yield: 55% (1.2 g) white solid, Mp. 67-69 °C [6]. ^{1}H NMR (400 MHz, $CDCl_3$) δ 5.84 (s, 1 H CH-OH), 7.26 (d, *J*=7.32 Hz, 2 H Ar-H), 7.30 - 7.35 (m, 4 H Ar-H), 7.35 - 7.39 (m, 4 H Ar-H). ^{13}C NMR (101 MHz, $CDCl_3$) δ 76.28 (CH-OH), 126.53 (2xCH), 127.57 (4xCH), 128.50 (4xCH), 143.78 (2xC). LRMS (EI): found 185.17 $(M+H)^+$ $C_{13}H_{13}O$ requires 185.10. IR: ν_{max} (ATR) cm^{-1}: 3359, 2973, 2898, 2936, 1611, 1590, 1508, 1459, 1423, 1325, 1234, 1124, 1054, 1036, 999, 971, 827, 816, 716, 658, 623.

(4-Nitrophenyl)(phenyl)methanol (12b): As per general method A, 4-nitrobenzophenone **(11b)** (1 eq, 6.6 mmol, 1.5 g) and sodium borohydride (2 eq, 13.20 mmol, 0.5 g) were reacted. Yield 97% (1.47 g), yellow solid Mp. 65-68 °C [7]. ^{1}H NMR (400 MHz, $CDCl_3$) δ 5.91 (s, 1 H, CH-OH), 7.27 - 7.38 (m, 5 H, Ar-H), 7.57 (d, *J*=8.54 Hz, 2 H, Ar-H), 8.18 (d, *J*=9.16 Hz, 2 H, Ar-H). ^{13}C NMR (101 MHz, $CDCl_3$) δ 75.49 (CH-OH), 123.64 (2xCH), 126.68 (2xCH), 127.03 (x2CH), 128.37 (CH), 128.92 (2xCH), 142.67 (C), 147.13 (C-NO_2), 150.73 (C). HRMS (EI): found 264.0446 $(M+Cl)^+$ $C_{13}H_{11}{}^{35}ClNO_3$ requires 264.0427. IR: ν_{max} (ATR) cm^{-1}: 3445, 3106, 3078, 2990, 1595, 1504, 1339, 1188, 1181, 1047, 1024, 1012, 830, 811, 759, 754, 693, 616, 552.

(4-Bromophenyl)(phenyl)methanol (12c): As per general method A, 4-bromobenzophenone **(11c)** (1 eq, 9.5 mmol, 2.5 g) and sodium borohydride (2 eq, 19.5 mmol, 0.72 g) were reacted. Yield: 90% (2.27 g) brown solid Mp. 76-80 °C [8]. ^{1}H NMR (400 MHz, $CDCl_3$) δ 5.74 (s, 1 H, CH-OH), 7.22 (d, *J*=7.93 Hz, 2 H, Ar-H), 7.25 - 7.36 (m, 5 H, Ar-H), 7.44 (d, *J*=8.54 Hz, 2 H, Ar-H). ^{13}C NMR (101 MHz, $CDCl_3$) δ 75.60 (CH-OH), 121.38 (C-Br), 126.52 (CH), 127.84 (2xCH), 128.21 (2xCH), 128.63 (2xCH), 131.51 (2xCH), 142.72 (C), 143.34 (C). HRMS (EI): found 244.9969 $(M-OH)^+$

$C_{13}H_{10}{}^{79}Br$ requires 244.9966. IR: ν_{max} (ATR) cm^{-1}: 3295, 3085, 3029, 1702, 1559, 1482, 1452, 1243, 1191, 1154, 1068, 1034, 1007, 919, 902, 838, 846, 825, 711, 698, 618.

(4-Fluorophenyl)(phenyl)methanol (12d): As per general method A, 4-fluorobenzophenone **(11d)** (1 eq, 7.49 mmol, 1.5 g) and sodium borohydride (2 eq, 15 mmol, 0.56 g) were reacted. Yield: 93% (1.41 g) white solid Mp: 46-48°C [9]. ^{1}H NMR (400 MHz, $CDCl_3$) δ 5.81 (br. s., 1 H, CH-OH), 6.98 - 7.02 (m, 2 H, Ar-H), 7.26 - 7.35 (m, 7 H, Ar-H). ^{13}C NMR (101 MHz, $CDCl_3$) δ 75.63 (CH-OH), 115.19 (CH), 115.40 (CH), 126.46 (CH), 127.75 (2xCH), 128.18 (CH), 128.26 (CH), 128.59 (2xCH), 139.57 (C), 143.64 (C), 160.94 (C-F). HRMS (EI): found 201.0720 $(M-H)^+$ $C_{13}H_{10}FO$ requires 201.0716. IR: ν_{max} (ATR) cm^{-1}: 3307, 3064, 3031 1602, 1506, 1218, 1184, 1156, 1034, 1020, 1012, 919, 872, 849, 811, 792, 744, 697, 562.

(4-Methoxyphenyl)(phenyl)methanol (12e): As per general method A, 4-methoxybenzophenone **(11e)** (1 eq, 4.7 mmol, 1 g) and sodium borohydride (2 eq, 9.4 mmol, 0.35 g) were reacted. Yield: 85% (1.28 g) white solid Mp: 48-49 °C [10]. ^{1}H NMR (400 MHz, $CDCl_3$) δ 3.77 (s, 3 H, CH_3), 5.80 (d, *J*=3.05 Hz, 1 H, CH-OH), 6.85 (d, *J*=8.54 Hz, 2 H, Ar-H), 7.27 (d, *J*=8.54 Hz, 3 H, Ar-H), 7.29 - 7.34 (m, 2 H, Ar-H), 7.34 - 7.38 (m, 2 H, Ar-H). ^{13}C NMR (101 MHz, $CDCl_3$) δ 55.27 (CH_3), 75.81 (CH-OH), 113.86 (2xCH), 126.37 (2xCH), 127.42 (CH), 127.89 (C), 128.42 (2xCH), 136.15 (2xCH), 143.98 (C), 159.05 (C-O). LRMS (EI): found 213.12 $(M-H)^+$; $C_{14}H_{13}O_2$ requires 213.09. IR: ν_{max} (ATR) cm^{-1}: 3399, 3067, 2909, 2836, 1609, 1586, 1510, 1494, 1444, 1344, 1304, 1248, 1172, 1031, 1017, 1008, 861, 840, 808, 724, 695, 653, 576.

Bis(4-chlorophenyl)methanol (12f): As per general method A, 4,4'-dichlorobenzophenone **(11f)** (1 eq, 3.98 mmol, 1 g) and sodium borohydride (2 eq, 7.96 mmol, 0.30 g) were reacted. Yield: 99% (1 g) white solid Mp: 97-100 °C [11]. ^{1}H NMR (400 MHz, $CDCl_3$) δ 5.78 (d, *J*=3.66 Hz, 1 H, CH-OH), 7.25 - 7.31 (m, 8 H, Ar-H). ^{13}C NMR (101 MHz, $CDCl_3$) δ 74.96 (CH-OH), 127.84 (8xCH), 128.75 (2xC), 133.60 (2xC-Cl). HRMS (EI): found 251.0033 $(M-H)^+$; $C_{13}H_9{}^{35}Cl_2O$ requires 251.0031. IR: ν_{max} (ATR) cm^{-1}: 3222, 3009, 1638, 1486, 1408, 1088, 1037, 1011, 943, 827, 811, 793, 651, 553.

Phenyl(*p*-tolyl)methanol (12g): As per general method A, 4-methylbenzophenone **(11g)** (1 eq, 7.6 mmol, 1.5 g) and sodium borohydride (2 eq, 15.3 mmol, 0.58 g) were reacted.

Yield: 95% (1.42 g) white solid Mp: 62-65°C [11]. ^{1}H NMR (400 MHz, $CDCl_3$) δ 2.31 (s, 3 H, CH_3), 5.80 (br. s., 1 H, CH-OH), 7.13 (d, *J*=7.32 Hz, 2 H, Ar-H), 7.24 - 7.26 (m, 3 H, Ar-H), 7.29 - 7.33 (m, 2 H, Ar-H), 7.35 - 7.38 (m, 2 H, Ar-H). ^{13}C NMR (101 MHz, $CDCl_3$) δ 21.10 (CH_3), 76.10 (CH-OH), 126.43 (2xCH), 126.50 (2xCH), 127.44 (CH), 128.44 (CH), 129.17 (2xCH), 137.28 (C), 140.95 (C), 143.94 (C). LRMS (EI): Found 197.22 $(M-H)^+$; $C_{14}H_{13}O$ requires 197.10. IR: ν_{max} (ATR) cm^{-1}: 3347, 2890, 2859, 1509, 1494, 1455, 1171, 1024, 1019, 860, 795, 775, 696.

4-(Hydroxyl(phenyl)methyl)phenyl acetate (12h): As per general method A, 4-benzoylphenyl acetate **(11h)** (1 eq, 5 mmol, 1.2 g) and sodium borohydride (2 eq, 10 mmol, 0.37 g) were reacted. Yield: 91% (0.527 g) colourless oil [12]. ^{1}H NMR (400 MHz, $CDCl_3$) δ 2.27 (s, 3 H, CH_3), 5.83 (s, 1 H, CH-OH), 7.04 (d, *J*=7.93 Hz, 2 H, Ar-H), 7.26 (d, *J*=6.71 Hz, 1 H, Ar-H), 7.30 - 7.39 (m, 6 H, Ar-H). ^{13}C NMR (101 MHz, $CDCl_3$) δ 21.11 (CH_3), 75.72 (CH-OH), 121.52 (2xCH), 126.53 (2xCH), 127.65 (2xCH), 127.69 (CH), 128.54 (2xCH), 132.83 (C), 141.33 (C), 143.45 (C-O), 169.53 (C=O). HRMS (EI): found 265.0831 $(M+Na)^+$; $C_{15}H_{14}NaO_3$ requires 265.0841. IR: ν_{max} (ATR) cm^{-1}: 3451, 1748, 1602, 1503, 1368, 1318, 1279, 1191, 1164, 1013, 912, 851, 742, 697, 606.

((4-Benzyloxy)phenyl)(phenyl)methanol (12k): As per general method A, (4-(benzyloxy)phenyl)(phenyl)methanone **(11k)** (1 eq, 6.5 mmol, 1.89 g) and sodium borohydride (2 eq, 13.10 mmol, 0.49 g) of were reacted. Yield: 91% (1.73 g) white solid Mp: 61-64°C [13]. ^{1}H NMR (400 MHz, DMSO-d_6) δ 5.02 (s, 2 H, CH_2), 5.72 (d, *J*=3.66 Hz, 1 H, CH-OH), 6.89 (d, *J*=8.54 Hz, 2 H, Ar-H), 7.20 - 7.27 (m, 5 H, Ar-H), 7.28 - 7.35 (m, 5 H, Ar-H), 7.35 - 7.38 (m, 2 H, Ar-H). ^{13}C NMR (101 MHz, $CDCl_3$) δ 70.00 (CH_2), 75.78 (CH-OH), 114.78 (2xCH), 126.37 (CH), 127.41 (2xCH), 127.90 (CH), 128.16 (C), 128.41 (2xCH), 128.55 (2xCH), 129.71 (2xCH), 136.40 (C), 136.92 (2xCH), 143.95 (C), 158.24 (C-O). LRMS (APCI): found 313.27 $(M+Na)^+$; $C_{20}H_{18}NaO_2$ requires 313.12. IR: ν_{max} (ATR) cm^{-1}: 3099, 3038, 2863, 1639, 1599, 1575, 1505, 1288, 1242, 1172, 1149, 938, 860, 795, 741, 691.

Bis(4-((*tert*-butyldimethylsilyl)oxy)phenyl)methanol (12l): As per general method A, bis(4-((*tert*-butyldimethylsilyl)oxy)phenyl)methanone **(11l)** (1 eq, 3.61 mmol, 1.60 g) and sodium borohydride (2 eq, 7.22 mmol, 0.27 g) were reacted. to afford the desired

compound after purification *via* flash chromatography (eluent: *n*-hexane/ethyl acetate 8:2) . Yield: 38% (0.61 g) colourless oil [14]. ^{1}H NMR (400 MHz, $CDCl_3$) δ 0.21 (d, *J*=2.01 Hz, 12 H, CH_3), 0.99 - 1.01 (m, 18 H, CH_3), 5.77 (br. s., 1 H, CH-OH), 6.82 (dd, *J*=8.53, 2.01 Hz, 4 H, Ar-H), 7.21 - 7.25 (m, 4 H, Ar-H). ^{13}C NMR (101 MHz, $CDCl_3$) δ -4.42 (4x(Si)CH_3), 18.16 (2xC), 25.66 (6xCH_3), 84.66 (CH-OH), 119.80 (4xCH), 127.75 (2xC), 128.14 (4xCH), 154.89 (2xC-O). LRMS (EI): found 443.41 $(M-H)^+$; $C_{25}H_{39}O_3Si_2$ requires 443.24. IR: ν_{max} (ATR) cm^{-1}: 3060, 2995, 2886, 1606, 1506, 1250, 1164, 1091, 1012, 908, 836, 798, 778, 663, 555.

(4-Ethoxyphenyl)(phenyl)methanol (12m): As per general method A, (4-ethoxyphenyl)(phenyl)methanone **(11m)** (1 eq, 5.0 mmol, 1.13 g) and sodium borohydride (2 eq, 10.0 mmol, 0.38 g) were reacted. Purification *via* flash chromatography (eluent: *n*-hexane/ethyl acetate 8:2) afforded a colourless oil, Yield: 80% (0.91 g) [15]. ^{1}H NMR (400 MHz, $CDCl_3$) δ 1.39 (t, *J*=7.05 Hz, 3 H, CH_3), 4.00 (q, *J*=7.05 Hz, 2 H, CH_2), 5.78 (s, 1 H, CH-OH), 6.81 - 6.87 (m, 2 H, Ar-H), 7.23 - 7.27 (m, 3 H, Ar-H), 7.29 - 7.39 (m, 4 H, Ar-H). ^{13}C NMR (101 MHz, $CDCl_3$) δ 14.80 (CH_3), 63.40 (CH_2), 75.77 (CH-OH), 114.37 (2xCH), 126.36 (CH), 127.35 (C), 127.86 (2xCH), 128.37 (2xCH), 135.96 (2xCH), 143.98 (C), 158.36 (C-O). HRMS (EI): found 211.1127 $(M-OH)^+$; $C_{15}H_{15}O$ requires 211.1117. IR: ν_{max} (ATR) cm^{-1}: 3483, 2979, 2875, 1611, 1507, 1445, 1421, 1241, 1149, 1170, 1044, 920, 899, 788, 697, 621, 592, 560.

General method B: **Reaction of triazole with secondary alcohols**

To a solution of the specific secondary alcohol (1 eq,) in toluene (60 mL) 1,2,4-triazole was added (3 eq,) and *p*-TSA (200 mg) in a round bottom flask connected to a Dean-Stark trap. The source of heating used for the reaction is an open vessel microwave reactor (90-250 W). The mixture was refluxed for 4 h, the toluene was evaporated and the crude product was re-dissolved in ethyl acetate (30 mL) and washed with water (20 mL) and brine (10 mL). The product was dried over sodium sulphate, filtered and concentrated under reduced pressure. The crude product was purified *via* flash chromatography (*n*-hexane/ethyl acetate 1:1) over silica gel to afford the desired product.

1-Benzhydryl-1*H*-1,2,4-triazole (13a): As per general method B, diphenylmethanol **(17)** (1 eq, 2.7 mmol, 0.5 g) was reacted with 1,2,4-triazole (3 eq, 8.1 mmol, 0.56 g) and *p*-TSA (200 mg) in toluene (60 mL). The crude product was purified *via* flash

chromatography (eluent: *n*-hexane/ethyl acetate from 2:1 to 1:1). Yield: 71% (0.45 g) white solid Mp 106-109 °C [16]. ^{1}H NMR (400 MHz, $CDCl_3$) δ 6.76 (s, 1 H, CH-N-R), 7.10 - 7.14 (m, 4 H, Ar-H), 7.32 - 7.40 (m, 6 H, Ar-H), 7.97 (br. s., 1 H, CH-N), 8.05 (s, 1 H, CH-N). ^{13}C NMR (101 MHz, $CDCl_3$) δ 67.86 (CH-N-R), 128.12 (4xCH), 128.60 (2xCH), 128.94 (4xCH), 137.92 (2xC), 143.54 (CH-N), 152.30 (CH-N). HRMS (EI): found 258.1003 $(M+Na)^+$; $C_{15}H_{13}N_3Na$ requires 258.1007. IR**:** ν_{max} (ATR) cm^{-1}: 3120, 3027, 2940, 1492, 1458, 1450, 1342, 1192, 1135, 1091, 961, 922, 844, 680, 663.

1-((4-Bromophenyl)(phenyl)methyl)-1*H*-1,2,4-triazole (13b): As per general method B, compound **12c** (1 eq, 1.91mmol, 0.5 g) was reacted with 1,2,4-triazole and *p*-TSA in toluene. The product was purified *via* flash chromatography (eluent: *n*-hexane/ethyl acetate 1:1) and obtained as an orange solid**,** 3% (0.024 g), Mp. 94-97 °C, (HPLC 94%). IR: ν_{max} (ATR) cm^{-1}: 3124, 3036, 2927, 1499, 1490, 1454, 1204, 1207, 1338, 1016. ^{1}H NMR (400 MHz, $CDCl_3$) δ 6.69 (s, 1 H, CH-N-R), 6.98 (d, *J*=7.32 Hz, 2 H, Ar-H), 7.12 (br. s., 2 H, Ar-H), 7.36 (br. s., 3 H, Ar-H), 7.49 (d, *J*=7.32 Hz, 2 H, Ar-H), 7.94 (br. s., 1 H, CH-N), 8.02 (br. s., 1 H, CH-N). ^{13}C NMR (101 MHz, $CDCl_3$) δ 67.20 (CH-N-R), 122.75 (C-Br), 128.16 (2xCH), 128.89 (CH), 129.09 (2xCH), 129.67 (2xCH), 132.08 (2xCH), 137.07 (C), 137.27 (C), 152.46 (CH-N). HRMS (EI): found 314.0292 $(M+H)^+$; $C_{15}H_{13}{}^{79}BrN_3$ requires 314.0293.

1-((4-Methoxyphenyl)(phenyl)methyl)-1*H*-1,2,4-triazole (13d): As per general method B, compound **12e** (1 eq, 2.33 mmol, 0.5 g) was reacted with 1,2,4-triazole and *p*-TSA in toluene. The product was purified *via* flash chromatography (eluent: *n*-hexane/ethyl acetate 1:1), white solid, 82% (0.415 g), Mp. 85-89 °C, (HPLC 98%). IR: ν_{max} (ATR) cm^{-1}: 3154, 3123, 3052, 1608, 1511, 1497, 1276, 1229, 1139, 1016. ^{1}H NMR (400 MHz, $CDCl_3$) δ 3.82 (s, 3 H, CH_3), 6.73 (s, 1 H, CH-N-R), 6.88 - 6.93 (m, 2 H, Ar-H), 7.08 - 7.13 (m, 4 H, Ar-H), 7.34 - 7.41 (m, 3 H, Ar-H), 7.92 (s, 1 H, CH-N), 8.03 (s, 1 H, CH-N). ^{13}C NMR (101 MHz, $CDCl_3$) δ 55.32 (CH_3), 67.37 (CH-N-R), 114.31 (2xCH), 127.75 (2xCH), 128.41 (CH), 128.87 (4xCH), 129.63 (C), 138.39 (C), 143.45 (CH-N), 152.26 (CH-N), 158.54 (C-O). LRMS (EI): found 265.97 $(M+H)^+$; $C_{16}H_{16}N_3O$ requires 265.12.

1-(Phenyl(*p*-tolyl)methyl)-1*H*-1,2,4-triazole (13f): As per general method B, compound **12g** (1 eq, 3.28 mmol, 0.65 g) was reacted with 1,2,4-triazole and *p*-TSA in toluene. The product was purified *via* flash chromatography (eluent: *n*-hexane/ethyl acetate 1:1), white solid, 30% (0.25 g), Mp. 96-98 °C [33], (HPLC 96%). IR: ν_{max} (ATR) cm^{-1}: 3106, 3030, 1513, 1498, 1456, 1430, 1273, 1215, 1134, 1013, 953, 891, 773, 697. ^1H NMR (400 MHz, $CDCl_3$) δ 2.34 (s, 3 H, CH_3), 6.71 (s, 1 H, CH-N-R), 7.02 (d, *J*=7.93 Hz, 2 H, Ar-H), 7.10 (dd, *J*=7.32, 1.83 Hz, 2 H, Ar-H), 7.16 (d, *J*=7.93 Hz, 2 H, Ar-H), 7.32 - 7.38 (m, 3 H, Ar-H), 7.89 (s, 1 H, CH-N), 8.00 (s, 1 H, CH-N). ^{13}C NMR (101 MHz, $CDCl_3$) δ 21.12 (CH_3), 67.68 (CH-N-R), 127.94 (2xCH) 128.16 (CH), 128.47 (2xCH), 128.88 (2xCH), 129.63 (2xCH), 134.89 (C-CH_3), 138.20 (C), 138.57 (C), 143.49 (CH-N), 152.24 (CH-N). HRMS (EI): found 250.1352 $(M+H)^+$; $C_{16}H_{16}N_3$ requires 250.1344.

4-(Phenyl(1*H*-1,2,4-triazol-1-yl)methyl)phenyl acetate (13g): As per general method B, compound **12h** (1 eq, 1.94 mmol, 0.47 g) was reacted with 1,2,4-triazole and *p*-TSA in toluene. The crude product was purified *via* flash chromatography (eluent: *n*-hexane/ethyl acetate 1:1) to afford a white solid, 30%, 0.17 g, Mp. 158-161 °C. IR: ν_{max} (ATR) cm^{-1}: 3105, 2948, 1749, 1639, 1601, 1506, 1373, 1279, 1211, 1168, 1025. ^1H NMR (400 MHz, $CDCl_3$) δ 2.30 (s, 3 H, CH_3), 6.76 (s, 1 H, CH-N-R), 7.11 - 7.16 (m, 5 H, Ar-H), 7.35 - 7.39 (m, 4 H, Ar-H), 7.95 (s, 1 H, CH-N), 8.03 (s, 1 H, CH-N). ^{13}C NMR (101 MHz, $CDCl_3$) δ 21.12 (CH_3), 67.31 (CH-N-R), 122.10 (2xCH), 127.72 (CH), 128.10 (2xCH), 129.02 (2xCH), 129.25 (2xCH), 135.39 (C), 137.60 (C), 144.36 (CH-N), 150.71 (C-O), 152.26 (CH-N), 169.19 (C=O). HRMS (EI): found 294.1236 $(M+H)^+$; $C_{17}H_{16}N_3O_2$ requires 294.1243.

***N*-(4-(Phenyl(1*H*-1,2,4-triazol-1-yl)methyl)phenyl)acetamide (13h):** As per general method B, compound **12i** (1 eq, 2.15 mmol, 0.52 g) was reacted with 1,2,4-triazole and *p*-TSA in toluene. The crude product was purified *via* flash chromatography (eluent: DCM/methanol 9:1) to afford a white solid, 23%, 0.15 g, Mp. 217-219 °C, (HPLC 93%). IR: ν_{max} (ATR) cm^{-1}: 3257, 3191, 3125, 3053, 1669, 1607, 1551, 1512, 1498, 1412, 1325, 1276, 1208, 1169, 1138, 1083, 1017. ^1H NMR (400 MHz, DMSO-d_6) δ 1.99 (s, 3 H, CH_3), 6.97 (s, 1 H, CH-N-R), 7.12 - 7.17 (m, 4 H, Ar-H), 7.28 - 7.37 (m, 3 H, Ar-H), 7.52 (d, *J*=8.54 Hz, 2 H, Ar-H), 8.01 (s, 1 H, CH-N), 8.52 (s, 1 H, CH-N), 9.96 (s, 1 H, NH).

^{13}C NMR (101 MHz, DMSO- d_6) δ 168.80 (C=O), 152.28 (CH-N), 144.72 (CH-N), 139.71 (C), 139.49 (C), 133.70 (C-NH), 129.00 (2xCH), 128.33 (CH), 128.24 (2xCH), 119.41 (4xCH), 65.70 (CH-N-R), 24.40 (CH_3). HRMS (EI): found 291.1241 $(M-H)^+$; $C_{17}H_{15}N_4O$ requires 291.1246.

1-(Bis(4-((*tert*-butyldimethylsilyl)oxy)phenyl)methyl)-1*H*-1,2,4-triazole (13k): As per general method B, compound **12l** (1 eq, 1.37 mmol, 0.61 g) was reacted with 1,2,4-triazole and *p*-TSA in toluene. The crude product was purified *via* flash chromatography (eluent: *n*-hexane/ethyl acetate 1:1) to afford a colourless oil, 78%, 0.53 g. IR: ν_{max} (ATR) cm^{-1}: 2930, 2858, 1605, 1508, 1471, 1254, 1169, 1136. ^1H NMR (400 MHz, $CDCl_3$) δ 0.18 (d, *J*=3.66 Hz, 12 H, Si-CH_3), 0.96 (d, *J*=3.66 Hz, 18 H, CH_3), 6.63 (br. s., 1 H, CH-N-R), 6.80 (dd, *J*=8.54, 3.05 Hz, 4 H, Ar-H), 6.91 - 6.98 (m, 4 H, Ar-H), 7.81 - 7.86 (m, 1 H, CH-N), 7.97 - 8.01 (m, 1 H, CH-N). ^{13}C NMR (101 MHz, $CDCl_3$) δ -4.42 (4xCH_3), 25.62 (6xCH_3), 32.45 (2xC), 67.01 (CH-N-R), 120.32 (4xCH), 129.22 (4xCH), 130.87 (2xC), 143.37 (CH-N), 152.17 (CH-N), 155.81 (2xC-O). LRMS (EI): found 518.25 $(M+Na)^+$; $C_{27}H_{41}N_3NaO_2Si_2$ requires 518.26.

1-((4-Propoxyphenyl)(3,4,5-trimethoxyphenyl)methyl)-1*H*-1,2,4-triazole (16a): As per general method B, compound **15a** (1 eq, 1.5 mmol, 0.5 g) was reacted with 1,2,4-triazole (3 eq, 4.5 mmol, 0.31 g) and *p*-TSA (0.61 eq, 200 mg) in toluene (60 mL). The crude product was purified *via* flash chromatography (eluent: *n*-hexane/ethyl acetate 1:1), white solid, 93%, 0.53 g, Mp. 98-99°C, (HPLC 99%). IR: ν_{max} (ATR) cm^{-1}: 3569, 3103, 2971, 2939, 2882, 1612, 1591, 1508, 1461, 1418, 1305, 1251, 1176, 1221, 1002. ^1H NMR (400 MHz, $CDCl_3$) δ 1.01 (t, *J*=7.63 Hz, 3 H, CH_3), 1.76 - 1.82 (m, 2 H, CH_2), 3.73 (s, 6 H, OCH_3), 3.82 (s, 3 H, OCH_3), 3.90 (t, *J*=6.41 Hz, 2 H, CH_2), 6.27 (s, 2 H, Ar-H), 6.61 (s, 1 H, CH-N-R), 6.88 (d, *J*=8.54 Hz, 2 H, Ar-H), 7.06 (d, *J*=8.54 Hz, 2 H, Ar-H), 7.90 (s, 1 H, CH-N), 8.01 (s, 1 H, CH-N). ^{13}C NMR (101 MHz, $CDCl_3$) δ 10.49 (CH_3), 22.50 (CH_2), 56.12 (OCH_3), 60.84 (2xOCH_3), 67.47 (CH-N-R), 69.59 (CH_2), 104.95 (2xCH), 114.85 (2xCH), 129.31 (C), 129.52 (2xCH), 133.90 (C), 137.93 (C-O), 143.48 (CH-N), 152.28 (2xC-O), 153.53 (CH-N), 159.39 (C-Pr). HRMS (EI): found 406.1729 $(M+Na)^+$; $C_{21}H_{25}N_3NaO_4$ requires 406.1743.

1-(Bis(4-chlorophenyl)methyl)-1*H*-1,2,4-triazole (13e): As per general method B, bis(4-chlorophenyl)methanol **(12f)** (1 eq, 1.97 mmol, 0.5 g) was reacted with 1,2,4-triazole (3 eq, 5.92 mmol, 0.41 g) and *p*-TSA (200 mg) in toluene (60 mL). The crude product was purified *via* flash chromatography (eluent: *n*-hexane/ethyl acetate 5:3). Yield: 6% (0.035 g) dark brown solid Mp: 118-120 °C [17]. ^{1}H NMR (400 MHz, $CDCl_3$) δ ppm 6.67 (s, 1 H, CH-N-R), 7.03 - 7.08 (m, 4 H, Ar-H), 7.33 - 7.36 (m, 4 H, Ar-H), 7.94 (s, 1 H, CH-N), 8.01 (s, 1 H, CH-N). ^{13}C NMR (101 MHz, $CDCl_3$) δ ppm 66.42 (CH-N-R), 129.26 (4xCH), 129.39 (4xCH), 134.91 (2xC-Cl), 136.02 (2xC), 152.58 (CH-N). HRMS (EI): found 302.0266 $(M-H)^+$; $C_{15}H_{10}{}^{35}Cl_2N_3$ requires 302.0252. IR: ν_{max} (ATR) cm^{-1}: 3112, 3056, 2920, 2850, 1491, 1405, 1348, 1315, 1278, 1204, 1136, 1091, 1014, 957, 875, 857, 840, 802, 788, 678.

4,4'-((1*H*-1,2,4-Triazol-1-yl)methylene)diphenol (13o): A solution of 1-(bis(4-((*tert*-butyldimethylsilyl)oxy)phenyl)methyl)-1*H*-1,2,4-triazole **(13k)** (1 eq, 0.25 mmol, 0.517 g) in dry THF was reacted with TBAF (2.5 eq, 0.625 mmol, 0.16 g, 0.18 mL) at 0° C. After completion the solution was diluted with ethyl acetate (50 mL) washed with HCl 0.1 M (20 mL) and water (20 mL). The crude product was then purified *via* flash chromatography (eluent: *n*-hexane/ethyl acetate 1:1). Yield: 90% (0.125 g) pink solid Mp: 229-230 °C [18]; HPLC: 97% ^{1}H NMR (400 MHz, DMSO-d_6) δ 6.69 (d, *J*=8.54 Hz, 4 H, Ar-H), 6.75 (s, 1 H, CH-N-R), 6.96 (d, *J*=8.54 Hz, 4 H, Ar-H), 7.97 (s, 1 H, CH-N), 8.41 (s, 1 H, CH-N), 9.50 (br. s., 2 H, OH). ^{13}C NMR (101 MHz, DMSO-d_6) δ 65.12 (CH-N-R), 115.16 (4xCH), 129.11 (4xCH), 129.69 (2xC), 143.93 (CH-N), 151.52 (CH-N), 156.98 (2xC-OH). HRMS (EI): found 302.0699 $(M+Cl)^+$; $C_{15}H_{13}{}^{35}ClN_3O_2$ requires 302.0697 IR: ν_{max} (ATR) cm^{-1}: 3132, 1604, 1508, 1473, 1443, 1228, 1207, 1135, 1110, 1011, 977, 836, 786, 678.

General method C: Reaction of aryl aldehyde with aryl bromide

In a 2 necked round bottom flask with a solution of the aryl bromide in dry THF was cooled to -78° under nitrogen. *n*-BuLi was added dropwise and the mixture was allowed to stir for 1 h under nitrogen. After 1 h a solution of the aryl aldehyde in dry THF was added and the mixture was allowed to stir for another 1.5 h at -78 °C. The mixture was then left stirring at room temperature for 2 h. After 2 h the mixture was concentrated under reduced pressure to remove the THF. The residue was re-dissolved in DCM (30

mL) and washed with water (20 mL) and brine (10 mL), dried over sodium sulfate, filtered and concentrate. The crude product was then purified *via* flash chromatography (eluent: *n*-hexane/ethyl acetate).

1-Bromo-4-propoxybenzene (14a): To a solution of 4-bromophenol (1eq, 11.56 mmol, 2 g) in DMF, K_2CO_3 (2.5 eq, 28.9 mmol, 3.99 g) was added followed by the addition of iodopropane (2 eq, 23.12 mmol, 3.93 g, 2.2 mL) and stirred overnight. The reaction was quenched with water (25 mL) and extracted with ethyl acetate (2x50 mL). The organic phase was then washed with brine (30 mL), dried over sodium sulfate, filtered and concentrated under reduced pressure. Yield: 100% (2.4 g) clear liquid [19]. ^{1}H NMR (400 MHz, $CDCl_3$) δ 0.97 (t, *J*=7.63 Hz, 3 H, CH_3), 1.70 - 1.77 (m, 2 H, CH_2), 3.83 (t, *J*=6.41 Hz, 2 H, CH_2), 6.72 (d, *J*=7.93 Hz, 2 H, Ar-H), 7.28 - 7.32 (m, 2 H, Ar-H). ^{13}C NMR (101 MHz, $CDCl_3$) δ 9.45 (CH_3), 21.47 (CH_2), 68.72 (CH_2), 111.52 (C-Br), 115.27 (2xCH), 131.15 (2xCH), 157.22 (C-O). IR: ν_{max} (ATR) cm^{-1}: 3501, 1663, 1545, 1488, 1387, 1286, 1256, 1090, 1065, 934, 898, 824, 766, 711, 658, 637, 598.

1-(Benzyloxy)-4-bromobenzene (14b): To a solution of 4-bromophenol (1 eq, 7.56 mmol, 1.5 g), in ACN (50 mL), K_2CO_3 (1.3 eq, 9.8 mmol, 1.35 g) of was added while stirring followed by the dropwise addition of benzyl bromide (1eq, 7.56 mmol, 0.90 mL). The mixture was stirred for 6 h under nitrogen atmosphere at reflux. After 6 h the mixture was filtered and concentrated giving the desired final product. Yield: 90% (1.37 g) white solid Mp: 66-68 °C [20]. ^{1}H NMR (400 MHz, $CDCl_3$) δ 5.02 (s, 2 H, CH_2), 6.83 (d, *J*=8.54 Hz, 2 H, Ar-H), 7.29 - 7.36 (m, 3 H, Ar-H), 7.37 (d, *J*=7.32 Hz, 4 H, Ar-H). ^{13}C NMR (101 MHz, $CDCl_3$) δ 70.21 (CH_2), 113.11 (C-Br), 116.68 (2xCH), 127.41 (2xCH), 128.10 (CH), 128.63 (2xCH), 132.27 (2xCH), 136.53 (C), 157.83 (C-O). IR: ν_{max} (ATR) cm^{-1}: 3089, 3062, 2888, 2823, 1587, 1486, 1452, 1378, 1246, 1170, 1041, 1026, 999, 823, 812, 731, 657, 618.

(4-Propoxyphenyl)(3,4,5-trimethoxyphenyl)methanol (15a): As per general method C, 1-bromo-4-propoxybenzene **(14a)** (1 eq, 5.3 mmol, 1.13 g) was dissolved in dry THF (50 mL). *n*-BuLi (2.5 mL) was added followed by the addition after 1 h of stirring of 3,4,5-trimethoxybenzaldehyde (1 eq, 5.3 mmol, 1.03 g). The product was concentrated, re-dissolved in DCM (50 mL) washed with water (30 mL) and brine (10 mL), dried over sodium sulphate, filtered and concentrated under reduced pressure. The crude product

was purified *via* flash chromatography (eluent: *n*-hexane/ethyl acetate 1:1). Yield: 53% (0.917 g) white solid Mp: 91-94 °C [15]. ^{1}H NMR (400 MHz, $CDCl_3$) δ 1.01 (t, *J*=7.32 Hz, 3 H, CH_3), 1.74 - 1.82 (m, 2 H, CH_2), 3.81 (s, 9 H, OCH_3), 3.89 (t, *J*=6.71 Hz, 2 H, CH_2), 5.72 (br. s., 1 H, CH-OH), 6.59 (s, 2 H, Ar-H), 6.85 (d, *J*=8.54 Hz, 2 H, Ar-H), 7.27 (s, 2 H, Ar-H). ^{13}C NMR (101 MHz, $CDCl_3$) δ 10.51 (CH_3), 22.57 (CH_2), 56.09 (2xCH_3), 60.81 (CH_3), 69.53 (CH_2), 75.90 (CH), 103.37 (2xCH), 114.47 (2xCH), 127.85 (C), 134.66 (C), 135.68 (C), 139.65 (2xCH), 153.22 (2xC), 158.73 (C). HRMS (EI): found 355.1523 $(M+Na)^+$; $C_{19}H_{24}NaO_5$ requires 355.1521. IR: ν_{max} (ATR) cm^{-1}: 3358, 2933, 2832, 1610, 1590, 1508, 1458, 1422, 1390, 1325, 1233, 1170, 1125, 1055, 971, 829, 794, 757, 662, 624.

(4-Methoxyphenyl)(3,4,5-trimethoxyphenyl)methanol (15c): As per general method C, 1-bromo-4-methoxybenzene **(14c)** (1 eq, 7.2 mmol, 1.34 g) was dissolved in dry THF (50 mL). *n*-BuLi (3.328 mL) was added followed by the addition after 1 h of stirring of 3,4,5-trimethoxybenzaldehyde (1 eq, 7.2 mmol, 1.41 g) . The product was concentrated, re-dissolved in DCM (50 mL) washed with water (30 mL) and brine (10 mL), dried over sodium sulphate, filtered and concentrated under reduced pressure. The crude product was purified *via* flash chromatography (eluent: *n*-hexane/ethyl acetate gradient 7:3 to1:1). Yield: 22% (0.5 g) pink solid Mp: 107-109 °C [21]. ^{1}H NMR (400 MHz, $CDCl_3$) δ 3.79 (s, 3 H, CH_3), 3.81 (s, 9 H, CH_3), 5.73 (d, *J*=2.44 Hz, 1 H, CH-OH), 6.59 (s, 2 H, Ar-H), 6.86 (d, *J*=8.54 Hz, 2 H, Ar-H), 7.28 (d, *J*=8.54 Hz, 2 H, Ar-H). ^{13}C NMR (101 MHz, $CDCl_3$) δ 55.26 (OCH_3), 56.07 (2xOCH_3), 60.80 (OCH_3), 75.85 (CH-OH), 103.36 (2xCH), 113.87 (2xCH), 127.86 (C), 135.89 (C), 137.13 (C-O), 139.63 (2xCH), 153.21 (2xC-O), 159.11 (C-O). HRMS (EI): found 327.1221 $(M+Na)^+$ $C_{17}H_{20}NaO_5$ requires 327.1209. IR: ν_{max} (ATR) cm^{-1}: 3358, 2936, 2837, 1611, 1590, 1508, 1459, 1423, 1325, 1234, 1125, 1055, 1034, 1000, 971, 866, 828, 816, 716, 659, 640.

(3,4-Dimethoxyphenyl)(3,4,5-trimethoxyphenyl)methanol (15d): As per general method C, 4-bromo-1,2-dimethoxybenzene **(14d)** (1 eq, 7.2 mmol, 1.56 g) was dissolved in dry THF (50 mL). *n*-BuLi (3.328mL) was added followed by the addition after 1 h of stirring of 3,4,5-trimethoxybenzaldehyde (1 eq, 7.2 mmol, 1.41 g). The product was concentrated, re-dissolved in DCM (50 mL) washed with water (30 mL) and brine (10 mL), dried over sodium sulphate, filtered and concentrated under reduced pressure. The

crude product was purified *via* flash chromatography (eluent: *n*-hexane/ethyl acetate 5:3). Yield: 30% (0.725 g) dark oil [22]. ^{1}H NMR (400 MHz, $CDCl_3$) δ 3.82 - 3.83 (m, 9 H, CH_3), 3.86 (s, 3 H, CH_3), 3.86 (s, 3 H, CH_3), 5.71 (d, *J*=2.90 Hz, 1 H, CH-OH), 6.60 (s, 2 H, Ar-H), 6.83 (d, *J*=8.29 Hz, 1 H, Ar-H), 6.88 (dd, *J*=8.29, 1.66 Hz, 1 H, Ar-H), 6.93 (d, *J*=2.07 Hz, 1 H, Ar-H). ^{13}C NMR (101 MHz, $CDCl_3$) δ 55.82 (2xOCH_3), 56.02 (2xOCH_3), 60.74 (OCH_3), 75.90 (CH-OH), 103.44 (2xCH), 109.74 (CH), 110.86 (CH), 118.93 (CH), 136.25 (C), 137.10 (C), 139.50 (C-O), 148.45 (C-O), 148.94 (C-O), 153.11 (2xC-O). HRMS (EI): found 357.1305 $(M+Na)^+$; $C_{18}H_{22}NaO_6$ requires 357.1314. IR: ν_{max} (ATR) cm^{-1}: 3503, 2936, 2835, 1587, 1504, 1452, 1413, 1328, 1259, 1229, 1120, 1024, 1003, 916, 858, 811, 731, 680, 616, 590.

Phenyl(3,4,5-trimethoxyphenyl)methanol (15e): As per general method C, bromobenzene **(14e)** (1 eq, 7.2 mmol, 1.13 g 0.75 mL) was dissolved in dry THF (50 mL). *n*-BuLi (3.328 mL) was added followed by the addition after 1 h of stirring of 3,4,5-trimethoxybenzaldehyde (1 eq, 7.2 mmol, 1.41 g). The product was concentrated, re-dissolved in DCM (50 mL) washed with water (30 mL) and brine (10 mL), dried over sodium sulphate, filtered and concentrated under reduced pressure. The crude product was purified *via* flash chromatography (eluent: *n*-hexane/ethyl acetate 2:1). Yield: 45% (0.878 g) white crystals Mp: 117-120 °C [23]. ^{1}H NMR (400 MHz, $CDCl_3$) δ 3.81 (s, 9 H, OCH_3), 5.76 (d, *J*=3.05 Hz, 1 H, CH-OH), 6.59 (s, 2 H, Ar-H), 7.24 - 7.29 (m, 1 H, Ar-H), 7.31 - 7.39 (m, 4 H, Ar-H). ^{13}C NMR (101 MHz, $CDCl_3$) δ 56.09 (2xOCH_3), 60.81 (OCH_3), 76.32 (CH-OH), 103.51 (2xCH), 126.49 (2xCH), 127.70 (2xCH), 128.52 (CH), 134.92 (C), 137.27 (C-O), 143.55 (C), 153.26 (2xC-O). HRMS (EI): found 275.1282 $(M+H)^+$; $C_{16}H_{19}O_4$ requires 275.1283. IR: ν_{max} (ATR) cm^{-1}: 3062, 3034, 3050, 2914, 2823, 1588, 1576, 1451, 1289, 1279, 1246, 1170, 1041, 905, 823, 813, 731, 657.

***p*-Tolyl(3,4,5-trimethoxyphenyl)methanol (15f):** As per general method C, 1-bromo-4-methylbenzene **(14f)** (1 eq, 7.2 mmol, 1.23 g 0.88 mL) was dissolved in dry THF (50 mL). *n*-BuLi (3.328 mL) was added followed by the addition after 1 h of stirring of 3,4,5-trimethoxybenzaldehyde (1 eq, 7.2 mmol, 1.41 g). The product was concentrated, re-dissolved in DCM (50 mL) washed with water (30 mL) and brine (10 mL), dried over sodium sulphate, filtered and concentrated under reduced pressure. The crude product was purified *via* flash chromatography (eluent: *n*-hexane/ethyl acetate 1:1). Yield: 41%

(0.843 g) white solid Mp: 94-98 °C [24]. ^{1}H NMR (400 MHz, $CDCl_3$) δ 2.32 (s, 3 H, CH_3), 3.80 (s, 3 H, OCH_3), 3.81 (s, 6 H, OCH_3), 5.73 (d, *J*=3.66 Hz, 1 H, CH-OH), 6.59 (s, 2 H, Ar-H), 7.14 (d, *J*=7.93 Hz, 2 H, Ar-H), 7.26 (s, 2 H, Ar-H). ^{13}C NMR (101 MHz, $CDCl_3$) δ 21.12 (CH_3), 56.08 (2xOCH_3), 60.80 (OCH_3), 76.16 (CH-OH), 103.39 (2xCH), 126.45 (2xCH), 129.20 (2xCH), 133.35 (C), 134.92 (C), 139.57 (C-O), 140.72 (C), 153.23 (2xC-O). HRMS (EI): found 311.1263 $(M+Na)^+$; $C_{17}H_{20}NaO_4$ requires 311.1263. IR: $ν_{max}$ (ATR) cm^{-1}: 3351, 2991, 2929, 1725, 1509, 1459, 1421, 1325, 1236, 1126, 1058, 1003, 969, 918, 825, 762, 709, 660, 620.

1-((4-Methoxyphenyl)(3,4,5-trimethoxyphenyl)methyl)-1*H*-1,2,4-triazole (16c): As per general method B, compound **15c** (1 eq, 1.61 mmol, 0.49 g) was reacted with 1,2,4-triazole (3 eq, 4.83 mmol, 0.33 g) and *p*-TSA (0.61 eq, 200 mg) in toluene (60 mL). The product was obtained as a yellow solid, 78%, 0.445 g, Mp. 119-122°C, (HPLC 100%). IR: $ν_{max}$ (ATR) cm^{-1}: 3570, 2937, 2833, 2279, 1594, 1508, 1462, 1418, 1338, 1303, 1276, 1238, 1124, 1034. ^{1}H NMR (400 MHz, $CDCl_3$) δ 3.73 (s, 6 H, OCH_3), 3.80 (s, 3 H, OCH_3), 3.83 (s, 3 H, OCH_3), 6.28 (s, 2 H, Ar-H), 6.62 (s, 1 H, CH-N-R), 6.87 - 6.91 (m, 2 H, Ar-H), 7.08 (d, *J*=8.55 Hz, 2 H, Ar-H), 7.91 (s, 1 H, CH-N), 8.01 (s, 1 H, CH-N). ^{13}C NMR (101 MHz, $CDCl_3$) δ 55.33 (OCH_3), 56.12 (2xOCH_3), 60.84 (OCH_3), 67.43 (CH-N-R), 104.97 (2xCH), 114.32 (2xCH), 129.54 (C), 129.59 (2xCH), 133.83 (C), 137.95 (C-O), 143.49 (CH-N), 152.29 (CH-N), 153.55 (2xC-O), 159.79 (C-O). HRMS (EI): found 378.1418 $(M+Na)^+$; $C_{19}H_{21}N_3NaO_4$ requires 378.1430.

1-((3,4-Dimethoxyphenyl)(3,4,5-trimethoxyphenyl)methyl)-1*H*-1,2,4-triazole (16d): As per general method B, compound **15d** (1 eq, 2.17 mmol, 0.725 g) was reacted with 1,2,4-triazole (3 eq, 6.5 mmol, 0.45 g) and *p*-TSA (0.61 eq, 200 mg) in toluene (60 mL). The crude product was purified *via* flash chromatography (eluent: *n*-hexane/ethyl acetate 1:2), off white solid, 52%, 0.438 g, Mp. 114-120°C. IR: $ν_{max}$ (ATR) cm^{-1}: 3131, 3070, 2937, 1592, 1518, 1508, 1495, 1421, 1414, 1272, 1257, 1234, 1124, 1039, 1020. ^{1}H NMR (400 MHz, $CDCl_3$) δ 3.76 (s, 6 H, OCH_3), 3.81 (s, 3 H, OCH_3), 3.85 (s, 3 H, OCH_3), 3.89 (s, 3 H, OCH_3), 6.32 (s, 2 H, Ar-H), 6.64 (s, 1 H, CH-N-R), 6.66 - 6.73 (m, 2 H, Ar-H), 6.87 (d, *J*=8.29 Hz, 1 H, Ar-H), 7.96 (s, 1 H, CH-N), 8.05 (s, 1 H, CH-N). ^{13}C NMR (101 MHz, $CDCl_3$) δ 55.91 (OCH_3), 55.94 (OCH_3), 56.13 (2xCH_3), 60.84

(OCH_3), 67.64 (CH-N-R), 105.06 (2xCH), 111.14 (CH), 111.28 (CH), 120.71 (CH), 129.90 (C), 133.58 (C), 138.01 (C-O), 143.51 (CH-N), 149.29 (C-O), 149.31 (C-O), 152.26 (CH-N), 153.53 (2xC-O). HRMS (EI): found 408.1538 $(M+Na)^+$; $C_{20}H_{23}N_3NaO_5$ requires 408.1535.

1-(*p*-Tolyl(3,4,5-trimethoxyphenyl)methyl)-1*H*-1,2,4-triazole (16f): As per general method B, compound **15f** (1 eq, 2.4 mmol, 0.692 g) was reacted with 1,2,4-triazole (3 eq, 7.2 mmol, 0.49 g) and *p*-TSA (0.61 eq, 200 mg) in toluene (60 mL). The crude product was purified *via* flash chromatography (eluent: *n*-hexane/ethyl acetate 1:1), white crystals, 67%, 0.524 g, Mp. 112-113 °C, (HPLC 100%). IR: ν_{max} (ATR) cm^{-1}: 3698, 3660, 2937, 2840, 1594, 1499, 1457, 1423, 1333, 1238, 1182, 1124, 873, 793, 753, 678. ^1H NMR (400 MHz, $CDCl_3$) δ 2.15 (s, 3 H, CH_3), 3.73 (s, 6 H, OCH_3), 3.82 (s, 3 H, OCH_3), 6.30 (s, 2 H, Ar-H), 6.63 (s, 1 H, CH-N-R), 7.03 (d, *J*=7.93 Hz, 2 H, Ar-H), 7.17 (d, *J*=7.93 Hz, 2 H, Ar-H), 7.92 (s, 1 H, CH-N), 8.01 (s, 1 H, CH-N). ^{13}C NMR (101 MHz, $CDCl_3$) δ 21.12 (CH_3), 56.10 (2xOCH_3), 60.82 (OCH_3), 67.72 (CH-N-R), 105.13 (2xCH), 128.06 (2xCH), 129.63 (2xCH), 133.61 (C), 134.63 (C), 137.99 (C-CH_3), 138.67 (C-O), 143.50 (CH-N), 152.26 (CH-N), 153.53 (2xC). HRMS (EI): found 340.1655 $(M+H)^+$; $C_{19}H_{22}N_3O_3$ requires 340.1661.

1-((4-Fluorophenyl)(3,4,5-trimethoxyphenyl)methyl)-1*H*-1,2,4-triazole (16g): As per general method B, compound **15g** (1 eq, 2.04 mmol, 0.597 g) was reacted with 1,2,4-triazole (3 eq, 6.12 mmol, 0.42 g) and *p*-TSA (0.6 eq, 200 mg) in toluene (60 mL). The crude product was purified *via* flash chromatography (eluent: *n*-hexane/ethyl acetate 1:1 to 1:2), white solid, 34%, 0.235 g, Mp. 115-118°C. (HPLC 100%). IR: ν_{max} (ATR) cm^{-1}: 3403, 3107, 2930, 2867, 1593, 1497, 1464, 1425, 1273, 1225, 1183, 1165. ^1H NMR (400 MHz, $CDCl_3$) δ 3.74 (s, 6 H, OCH_3), 3.83 (s, 3 H, OCH_3), 6.30 (s, 2 H, Ar-H), 6.65 (s, 1 H, CH-N-R), 7.05 - 7.12 (m, 4 H, Ar-H), 7.94 (s, 1 H, CH-N), 8.02 (s, 1 H, CH-N). ^{13}C NMR (101 MHz, $CDCl_3$) δ 56.15 (2xOCH_3), 60.86 (OCH_3), 67.17 (CH-N-R), 105.23 (2xCH), 115.85 (CH), 116.06 (CH), 129.81 (CH), 129.89 (CH), 133.14 (C), 133.64 (C), 138.23 (C-O), 143.53 (CH-N), 152.46 (CH-N), 153.65 (2xC-O), 163.89 (C-F). HRMS (EI): found 344.1415 $(M+H)^+$; $C_{18}H_{19}FN_3O_3$ requires 344.1411.

4-((1*H*-1,2,4-Triazol-1-yl)(3,4,5-trimethoxyphenyl)methyl)benzonitrile (16h): As per general method B, compound **15h** (1 eq, 1,15 mmol, 0,4 g) was reacted with 1,2,4-triazole (3 eq, 3.42 mmol, 0.24g) and *p*-TSA (0.6 eq, 200 mg) in toluene (60 mL). The crude product was purified *via* flash chromatography (eluent: *n*-hexane/ethyl acetate 1:2), white solid, 60%, 0.5 g, Mp. 144-146°C, (HPLC 97%). IR: ν_{max} (ATR) cm^{-1}: 3132, 3061, 2943, 2228, 1590, 1501, 1460, 1425, 1328, 1270, 1240, 1123, 1012, 1000. ^{1}H NMR (400 MHz, $CDCl_3$) δ 3.78 (s, 6 H, OCH_3), 3.87 (s, 3 H, OCH_3), 6.39 (s, 2 H, Ar-H), 6.70 (s, 1 H, CH-N-R), 7.22 (d, *J*=8.29 Hz, 2 H, Ar-H), 7.69 (d, *J*=8.29 Hz, 2 H, Ar-H), 8.03 (s, 1 H, CH-N), 8.07 (s, 1 H, CH-N). ^{13}C NMR (101 MHz, $CDCl_3$) δ 56.22 (2xCH_3), 60.89 (CH_3), 67.29 (CH), 105.75 (2xCH), 112.61 (C), 118.13 (CN), 128.45 (2xCH), 131.72 (C), 132.63 (2xCH), 138.72 (C), 143.26 (C), 143.67 (CH-N), 152.73 (CH-N), 153.83 (2xC). HRMS (EI): found 349.1296 $(M-H)^+$; $C_{19}H_{17}N_4O_3$ requires 349.1301.

Bis(3,4,5-trimethoxyphenyl)methanol (18b): As per general method C, 5-bromo-1,2,3-trimethoxybenzene (1 eq, 7.2 mmol, 1.77 g) was dissolved in dry THF (50 mL). *n*-BuLi (3.328 mL) was added followed by the addition after 1 h of stirring of 3,4,5-trimethoxybenzaldehyde (**17b**) (1 eq, 7.2 mmol, 1.41 g). The product was concentrated, re-dissolved in DCM (50 mL) washed with water (30 mL) and brine (10 mL), dried over sodium sulphate, filtered and concentrated under reduced pressure. The crude product was purified *via* flash chromatography (eluent: *n*-hexane/ethyl acetate 1:1). Yield: 16% (0.42 g) yellow solid Mp: 103-105 °C [25]. ^{1}H NMR (400 MHz, $CDCl_3$) δ 3.83 (s, 18 H, OCH_3), 5.69 (s, 1 H, CH-OH), 6.59 (s, 4 H, Ar-H). ^{13}C NMR (101 MHz, $CDCl_3$) δ 56.16 (4xOCH_3), 60.83 (2xOCH_3), 76.34 (CH-OH), 103.65 (4xCH), 137.41 (2xC), 139.12 (2xC-O), 153.27 (4xC-O). HRMS (EI): found 387.1414 $(M+Na)^+$; $C_{19}H_{24}NaO_7$ requires 387,1420. IR: ν_{max} (ATR) cm^{-1}: 3443, 2992, 2938, 2900, 1590, 1501, 1451, 1418, 1330, 1229, 1181, 1120, 1010, 992, 837, 788, 753, 729, 687, 646, 619, 575, 555.

Benzo[d][1,3]dioxol-5-yl(3,4,5-trimethoxyphenyl)methanol (18d): As per general method C, 5-bromo-1,2,3-trimethoxybenzene (1 eq, 4.37 mmol, 1.08 g) was dissolved in dry THF (50 mL). *n*-BuLi (2.05 mL) was added followed by the addition after 1 h of stirring of benzo[d][1,3]dioxole-5-carbaldehyde **(17d)** (1 eq, 4.37 mmol, 0.65 g). The product was concentrated, re-dissolved in DCM (50 mL) washed with water (30 mL) and

brine (10 mL), dried over sodium sulphate, filtered and concentrated under reduced pressure. The crude product was purified *via* flash chromatography (eluent: *n*-hexane/ethyl acetate 1:1). Yield: 30% (0.42 g) pale yellow solid Mp: 106-109°C [25]. ^{1}H NMR (400 MHz, $CDCl_3$) δ 3.80 - 3.83 (m, 9 H, OCH_3), 5.67 (s, 1 H, CH-OH), 5.93 (s, 2 H, CH_2), 6.58 (s, 2 H, Ar-H), 6.74 - 6.77 (m, 1 H, Ar-H), 6.81 - 6.86 (m, 2 H, Ar-H). ^{13}C NMR (101 MHz, $CDCl_3$) δ 56.10 (2xOCH_3), 60.82 (OCH_3), 76.04 (CH-OH), 101.07 (CH_2), 103.28 (2xCH_2), 107.12 (CH), 108.08 (CH), 120.02 (CH), 135.71 (C), 137.23 (C), 137.78 (C-O), 147.08 (C-O), 147.82 (C-O), 153.26 (2xC-O). LRMS (EI): Found 341.16 $(M+Na)^+$; $C_{17}H_{18}NaO_6$ requires 341.10. IR: ν_{max} (ATR) cm^{-1}: 3317, 2936, 2837, 1591, 1501, 1486, 1434, 1327, 1234, 1185, 1150, 1121, 1091, 1057, 975, 927, 845, 812, 771, 674, 627.

1-(Bis(3,4,5-trimethoxyphenyl)methyl)-1*H*-1,2,4-triazole (19b): As per general method B, compound **18b** (1 eq, 1.3 mmol, 0.47 g) was reacted with 1,2,4-triazole (3 eq, 3.8 mmol, 0.25 g) and *p*-TSA (0.61 eq, 200 mg) in toluene (60 mL). The crude product was purified *via* flash chromatography (eluent: *n*-hexane/ethyl acetate 1:2), off-white solid, 77%, 0.42 g, Mp. 132-135 °C, (HPLC 100%). IR: ν_{max} (ATR) cm^{-1}: 3570, 3127, 1590, 1459, 1417, 1337, 1242, 1230, 1119, 1017. ^{1}H NMR (400 MHz, $CDCl_3$) □ δ 3.75 (s, 12 H, OCH_3), 3.84 (s, 6 H, OCH_3), 6.33 (s, 4 H, Ar-H), 6.59 (s, 1 H, CH-N-R), 7.96 (s, 1 H, CH-N), 8.03 (s, 1 H, CH-N). ^{13}C NMR (101 MHz, $CDCl_3$) δ 56.18 (4x$OCH_{3,}$), 60.85 (2xOCH_3), 67.95 (CH-N-R), 105.30 (4xCH), 133.07 (2xC), 138.20 (2xC-O), 143.57 (CH-N), 152.35 (CH-N), 153.58 (4xC-O). HRMS (EI): found 438.1614 $(M+Na)^+$; $C_{21}H_{25}N_3NaO_6$ requires 438.1641.

1-((4-Ethoxyphenyl)(3,4,5-trimethoxyphenyl)methyl)-1*H*-1,2,4-triazole (19c): As per general method B, compound **18c** (1 eq, 1.66 mmol, 0.528 g) was reacted with 1,2,4-triazole (3 eq, 4.97 mmol, 0.34 g) and *p*-TSA (0.61 eq, 200 mg) in toluene (60 mL). The crude product was purified *via* flash chromatography (eluent: *n*-hexane/ethyl acetate 1:1), orange solid, 74%, 0.43 g, Mp. 140-145 °C, (HPLC 100%). IR: ν_{max} (ATR) cm^{-1}: 3570, 2972, 2939, 1612, 1592, 1508, 1496, 1461, 1416, 1276, 1247, 1236, 1000. ^{1}H NMR (400 MHz, $CDCl_3$) δ 1.40 (t, *J*=7.02 Hz, 3 H, CH_3), 3.73 (s, 6 H, OCH_3), 3.82 (s, 3 H, OCH_3), 4.02 (q, *J*=6.71 Hz, 2 H, CH_2), 6.28 (s, 2 H, Ar-H), 6.61 (s, 1 H, CH-N-R), 6.88 (d,

J=9.16 Hz, 2 H, Ar-H), 7.07 (d, J=8.54 Hz, 2 H, Ar-H), 7.90 (s, 1 H, CH-N), 8.01 (s, 1 H, CH-N). ^{13}C NMR (101 MHz, $CDCl_3$) δ 14.75 (CH_3), 56.11 (2xOCH_3), 60.84 (OCH_3), 63.55 (CH_2), 67.46 (CH-N-R), 104.96 (2xCH), 114.82 (2xCH), 129.38 (C), 129.52 (2xCH), 133.88 (C), 137.94 (C), 152.29 (CH-N), 153.54 (2xC-O), 159.19 (C-OEt). HRMS (EI): found 392.1594 $(M+Na)^+$; $C_{20}H_{23}N_3NaO_4$ requires 392.1586.

1-(Benzo[d][1,3]dioxol-5-yl(3,4,5-trimethoxyphenyl)methyl)-1*H*-1,2,4-triazole (19d): As per general method B, compound **18d** (1 eq, 1.32 mmol, 0.42 g) was reacted with 1,2,4-triazole (3 eq, 3.9 mmol, 0.27 g) and *p*-TSA (0.61 eq, 200 mg) in toluene (60 mL). The crude product was purified *via* flash chromatography (eluent: *n*-hexane/ethyl acetate 1:2), pale yellow solid, 95%, 0.456 g, Mp. 87-91 °C. IR: ν_{max} (ATR) cm^{1}: 3012, 2938, 2839, 1593, 1505, 1492, 1465, 1444, 1333, 1256, 1235, 1188, 1125, 1107, 1033. ^{1}H NMR (400 MHz, $CDCl_3$) δ 3.74 (s, 6 H, OCH_3), 3.83 (s, 3 H, OCH_3), 5.28 (s, 1 H, CH-N-R), 5.97 (s, 2 H, CH_2), 6.29 (s, 2 H, Ar-H), 6.57 (s, 1 H, Ar-H), 6.63 (s, 1 H, Ar-H), 6.79 (d, J=8.54 Hz, 1 H, Ar-H), 7.94 (s, 1 H, CH-N), 8.01 (s, 1 H, CH-N). ^{13}C NMR (101 MHz, $CDCl_3$) δ 56.14 (2xOCH_3), 60.84 (OCH_3), 67.61 (CH-N-R), 101.49 (CH_2), 104.98 (2xCH), 108.49 (CH), 108.60 (CH), 121.99 (CH), 131.38 (C), 133.51 (C), 138.07 (C-O), 139.42 (CH-N), 147.94 (C-O), 148.24 (C-O), 152.35 (CH-N), 153.58 (2xC-O). HRMS (EI): found 392.1228 $(M+Na)^+$; $C_{19}H_{19}N_3NaO_5$ requires 392.1222.

(4-Fluorophenyl)(3,4,5-trimethoxyphenyl)methanol (15g): As per general method C, compound **14g** (1 eq, 7.2 mmol, 1.26 g 0.79 mL) was dissolved in dry THF (50 mL). *n*-BuLi (3.328 mL) was added followed by the addition after 1 h of stirring of 3,4,5-trimethoxybenzaldehyde (1 eq, 7.2 mmol, 1.41 g). The product was concentrated, dissolved in DCM (50 mL) washed with water (30 mL) and brine (10 mL), dried over sodium sulphate and concentrated under reduced pressure. The crude product was purified *via* flash chromatography (eluent: *n*-hexane/ethyl acetate 1:1), yellow oil, 33%, 0.692 g. IR: ν_{max} (ATR) cm^{-1}: 3437, 2939, 2837, 1591, 1504, 1453, 1418, 1327, 1219, 1183, 1122, 1053, 1002. ^{1}H NMR (400 MHz, $CDCl_3$) δ 3.81 (s, 9 H, OCH_3), 5.74 (d, J=3.05 Hz, 1 H, CH-OH), 6.56 (s, 2 H, Ar-H), 6.99 - 7.04 (m, 2 H, Ar-H), 7.33 (dd, J=8.54, 5.49 Hz, 2 H, Ar-H). ^{13}C NMR (101 MHz, $CDCl_3$) δ 56.08 (2xOCH_3), 60.80 (OCH_3), 75.62 (CH-OH), 103.44 (2xCH), 115.19 (CH), 115.40 (CH), 128.15 (CH),

128.23 (CH), 132.23 (C), 137.35 (C-O), 139.24 (C), 153.30 (2xC-O), 160.96 (C-F). HRMS (EI): found 315.1014 $(M+Na)^+$; $C_{16}H_{17}FNaO_4$ requires 315.1009.

4-(Hydroxy(3,4,5-trimethoxyphenyl)methyl)benzonitrile (15h): As per general method C, compound **14h** (1 eq, 7.2 mmol, 1.31 g) was dissolved in dry THF (50 mL). *n*-BuLi (3.328 mL) was added followed by the addition after 1 h of stirring of 3,4,5-trimethoxybenzaldehyde (1 eq, 7.2 mmol, 1.41 g). The product was concentrated, dissolved in DCM (50 mL) washed with water (30 mL) and brine (10 mL), dried over sodium sulphate and concentrated under reduced pressure. The crude product was purified *via* flash chromatography (eluent: *n*-hexane/ethyl acetate 1:1), white solid, 21%, 0.452 g, Mp: 115-118°C. IR: ν_{max} (ATR) cm^{-1}: 3422, 2948, 2846, 2228, 1595, 1501, 1458, 1421, 1331, 1228, 1119, 1052. ^1H NMR (400 MHz, $CDCl_3$) δ 3.83 (s, 9 H, OCH_3), 5.79 (d, *J*=2.90 Hz, 1 H, CH-OH), 6.54 (s, 2 H, Ar-H), 7.52 (d, *J*=8.29 Hz, 2 H, Ar-H), 7.64 (d, *J*=8.29 Hz, 2 H, Ar-H). ^{13}C NMR (101 MHz, $CDCl_3$) δ 56.13 (2xOCH_3), 60.82 (OCH_3), 75.69 (CH-OH), 103.63 (2xCH), 111.28 (C-CN), 118.75 (CN), 126.95 (2xCH), 132.25 (2xCH), 137.78 (C-O), 138.36 (C), 148.53 (C), 153.51 (2xC-O). HRMS (EI): found 334.0850 $(M+Cl)^+$ $C_{17}H_{17}{}^{35}ClNO_4$ requires 334.0846.

(3-(Benzyloxy)-4-methoxyphenyl)(3,4,5-trimethoxyphenyl)methanol (18a): As per general method C, 5-bromo-1,2,3-trimethoxybenzene (1 eq, 7.2 mmol, 1.74 g) was dissolved in dry THF (50 mL). *n*-BuLi (3.328 mL) was added followed by the addition after 1 h of stirring of compound **17a** (1 eq, 7.2 mmol, 1.78 g). The product was concentrated, dissolved in DCM (50 mL) washed with water (30 mL) and brine (10 mL), dried over sodium sulphate and concentrated under reduced pressure. The crude product was purified *via* flash chromatography (eluent: *n*-hexane/ethyl acetate 5:1), off-white solid, 48%, 1.4 g, Mp. 97-101°C. IR: ν_{max} (ATR) cm^{-1}: 3499, 2937, 1591, 1505, 1455, 1418, 1330, 1260, 1232, 1128, 1024. ^1H NMR (400 MHz, $CDCl_3$) δ 3.76 (s, 6 H, CH_3), 3.81 (s, 3 H, CH_3), 3.85 (s, 3 H, CH_3), 5.10 (s, 2 H, CH_2), 5.64 (s, 1 H, CH-N-R), 6.50 (s, 2 H, Ar-H), 6.82 - 6.85 (m, 1 H, Ar-H), 6.88 - 6.90 (m, 2 H, Ar-H), 7.24 - 7.27 (m, 1 H, Ar-H), 7.28 - 7.33 (m, 2 H, Ar-H), 7.35 - 7.38 (m, 2 H, Ar-H). ^{13}C NMR (101 MHz, $CDCl_3$) δ 56.02 (CH_3), 56.04 (2xCH_3), 60.79 (CH_3), 70.94 (CH_2), 75.87 (CH-N-R), 103.33 (2xCH), 111.55 (CH), 112.64 (CH), 119.57 (CH), 127.29 (2xCH), 127.80 (CH),

128.45 (2xCH), 136.20 (C), 136.96 (C-O), 139.38 (2xC), 148.04 (C-O), 149.23 (C-OBn), 153.16 (2xC-O). HRMS (EI): found 433.1621 $(M+Na)^+$; $C_{24}H_{26}NaO_6$ requires 433.1627.

(4-Ethoxyphenyl)(3,4,5-trimethoxyphenyl)methanol (18c): As per general method C, 5-bromo-1,2,3-trimethoxybenzene (1 eq, 7.2 mmol, 1.77 g) was dissolved in dry THF (50 mL). *n*-BuLi (3.328 mL) was added followed by the addition after 1 h of stirring of compound **17c**(1 eq, 7.2 mmol, 1.08 g 1 mL). The product was concentrated, dissolved in DCM (50 mL) washed with water (30 mL) and brine (10 mL), dried over sodium sulphate and concentrated under reduced pressure. The crude product was purified *via* flash chromatography (eluent: *n*-hexane/ethyl acetate 1:1), white solid, 23%, 0.528 g, Mp. 106-109 °C. IR: ν_{max} (ATR) cm^{-1}: 3363, 3077, 2897, 2834, 1590, 1508, 1459, 1423, 1325, 1234, 1170, 1126, 1054, 1037. ^1H NMR (400 MHz, $CDCl_3$) δ 1.43 (t, *J*=7.03 Hz, 3 H, CH_3), 3.86 (s, 9 H, OCH_3), 4.05 (q, *J*=7.03 Hz, 2 H, CH_2), 5.77 (d, *J*=3.51 Hz, 1 H, CH-OH), 6.63 (s, 2 H, Ar-H), 6.90 (d, *J*=8.03 Hz, 2 H, Ar-H), 7.32 (s, 2 H, Ar-H). ^{13}C NMR (101 MHz, $CDCl_3$) δ 14.83 (CH_3), 56.09 (2xOCH_3), 60.82 (OCH_3), 63.46 (CH_2), 75.88 (CH-OH), 103.39 (2xCH), 114.44 (2xCH), 127.88 (C), 135.73 (C), 137.13 (C-O), 139.64 (2xCH), 153.22 (2xC-O), 158.52 (C-OEt). HRMS (EI): found 341.1368 $(M+Na)^+$; $C_{18}H_{22}NaO_5$ requires 341.1365.

4-((3-(Benzyloxy)-4-methoxyphenyl)(hydroxy)methyl)benzonitrile (18e) As per general method C, 4-bromobenzonitrile (1 eq., 1.31 g, 7.2 mmol), *n*-BuLi (3.328 mL, 2.5 M) and 3-benzyloxy-4-methoxybenzaldehyde (1 eq., 1.74 g, 7.2 mmol) were reacted. The material was purified *via* flash chromatography on silica gel (*n*-hexane : EtOAc, gradient 5:1 to 1:1) to afford the product as a cream solid, 2.01 g, 81%, Mp. 105-107 °C. IR : ν_{max} (KBr) cm^{-1}: 3514, 2227, 1607, 1511, 1253, 1223, 1137, 1017. ^1H NMR ($CDCl_3$, 400 MHz) δ 7.56 (d, *J* = 8.03 Hz, 2H, Ar-H), 7.30 - 7.41 (m, 7H, Ar-H), 6.84 - 6.90 (m, 2H, Ar-H), 6.80 (s, 1H, Ar-H), 5.72 (s, 1H, CH), 5.07 - 5.16 (m, 2H, CH_2), 3.88 (s, 3H, CH_3), 2.63 (br. s., 1H, OH). ^{13}C NMR ($CDCl_3$, 100 MHz) δ 149.1 (COBn), 148.5 (C_q), 147.7 (C_q), 136.3 (C_q), 134.9 (C_q), 131.7, 128.1, 127.4, 126.8, 126.4, 119.3, 118.5 (C_q), 112.2, 111.1, 110.4 (C_q), 74.7 (CH), 70.4 (CH_2), 55.6 (OCH_3). HRMS (EI): 368.1266 $(M+Na)^+$; $C_{22}H_{19}NNaO_3$ requires 368.1263.

4-((4-(Benzyloxy)phenyl)(hydroxy)methyl)benzonitrile (18f) As per general method C, 4-bromobenzonitrile (1 eq., 1.31 g, 7.2 mmol), n-BuLi (3.328 mL, 2.5 M) and 4-benzyloxybenzaldehyde (1 eq., 1.52 g, 7.2 mmol) were reacted. The material was purified *via* flash chromatography on silica gel (*n*-hexane:EtOAc, gradient 5:1 to 1:1) over silica gel to afford the product as a yellow solid, 1.99 g, 88%, Mp. 89-92 °C. IR: ν_{max} (KBr) cm^{-1}: 3469, 2234, 1605, 1506, 1230, 1007. ^{1}H NMR ($CDCl_3$, 400 MHz) δ 7.64 (d, *J* = 8.53 Hz, 2H, Ar-H), 7.52 (d, *J* = 8.03 Hz, 2H, Ar-H), 7.33 - 7.47 (m, 5H, Ar-H), 7.25 (d, *J* = 8.53 Hz, 2H, Ar-H), 6.97 (d, *J* = 9.03 Hz, 2H, Ar-H), 5.84 (br s, 1H), 5.07 (s, 2H, CH_2). ^{13}C NMR ($CDCl_3$, 100 MHz) δ 158.3 (COBn), 148.6 (C_q), 136.2 (C_q), 134.9 (C_q), 131.8, 128.2, 127.7, 127.6, 127.0, 126.5, 118.5 (C_q), 114.7, 110.5 (C_q), 74.7 (CH), 69.6 (CH_2) HRMS (EI): 338.1143 $(M+Na)^+$, $C_{21}H_{17}NNaO_2$ requires 338.1157.

General method D: Preparation of 1-(Diarylmethyl)-1*H*-imidazoles

To a solution of the secondary alcohol (1 eq) in dry acetonitrile (60 mL), CDI was added (1.3 eq,). The mixture was refluxed for 3 h under nitrogen, the acetonitrile was evaporated and the crude product was dissolved in DCM (30 mL) and washed with water (20 mL) and brine (10 mL). The product was dried over sodium sulphate and concentrated under reduced pressure. The crude product was purified *via* flash chromatography over silica gel to afford the desired product, (eluent: *n*-hexane/ethyl acetate 1:1).

1-((4-Fluorophenyl)(phenyl)methyl)-1*H*-imidazole (20d): As per general method D, compound **12d** (1 eq, 1.73 mmol, 0.35 g) was reacted with CDI in ACN (50 mL) at reflux for 3 h. The crude product was then purified *via* flash chromatography (eluent: *n*-hexane/ethyl acetate 1:1), yellow solid, 16%, 0.07 g, Mp. 96-99 °C, (HPLC 94%). IR: ν_{max} (ATR) cm^{-1}: 3124, 3030, 2913, 2694, 1736, 1603, 1508, 1495, 1298, 1260, 1223, 1187, 1157.^{1}H NMR (400 MHz, $CDCl_3$) δ 6.63 (s, 1 H, CH-N-R), 6.96 - 7.01 (m, 3 H, Ar-H), 7.10 (s, 2 H, Ar-H), 7.28 - 7.31 (m, 6 H, Ar-H), 7.68 (s, 1 H, CH-N). ^{13}C NMR (101 MHz, $CDCl_3$) δ 80.47 (CH-N-R) 115.58 (2xCH) 121.96 (CH) 126.75 (CH) 128.23 (CH) 128.60 (4xCH) 128.89 (CH) 128.94 (CH) 131.17 (C) 135.34 (C) 139.20 (CH). HRMS (EI): found 253.1142 $(M+H)^+$; $C_{16}H_{14}FN_2$ requires 253.1141.

1-((4-Methoxyphenyl)(phenyl)methyl)-1*H*-imidazole (20e): As per general method D, compound **12e** (1 eq, 1.86 mmol, 0.40 g) was reacted with CDI in ACN (50 mL) at reflux for 3h. The crude product was then purified *via* flash chromatography (eluent: *n*-hexane/ethyl acetate 1:2), yellow oil [34], 54%, 0.26 g. IR: ν_{max} (ATR) cm^{-1}: 3031, 2934, 1585, 1511, 1494, 1249, 1222, 1175, 1072, 1028. ^{1}H NMR (400 MHz, $CDCl_3$) δ 3.81 (s, 3 H, OCH_3), 6.47 (s, 1 H, CH-N-R), 6.84 (s, 1 H, Ar-H), 6.88 (d, *J*=7.32 Hz, 2 H, Ar-H), 7.03 - 7.10 (m, 5 H, Ar-H), 7.31 - 7.37 (m, 3 H, Ar-H), 7.40 (s, 1 H, CH-N). ^{13}C NMR (101 MHz, $CDCl_3$) δ 55.28 (OCH_3), 64.48 (CH-N-R), 114.15 (2xCH), 119.27 (CH-N), 127.70 (2xCH), 128.18 (CH-N), 128.77 (2xCH), 129.20 (CH), 129.42 (2xCH), 131.02 (C), 137.28 (CH-N), 139.53 (C), 159.50 (C-O). HRMS (EI): found 265.1342 $(M+H)^+$; $C_{17}H_{17}N_2O$ requires 265.1341.

1-(Phenyl(*p*-tolyl)methyl)-1*H*-imidazole (20g): As per general method D, compound **12g** (1 eq, 2.52 mmol, 0.50 g) was reacted with CDI (1.3 eq, 3.27 mmol, 0.53 g) in ACN (50 mL) at reflux for 3 h. The crude product was then purified *via* flash chromatography (eluent: *n*-hexane/ethyl acetate 1:1), orange oil, 54%, 0.334 g. IR: ν_{max} (ATR) cm^{-1}: 3028, 2920, 1654, 1603, 1513, 1493, 1451, 1413, 1278, 1222, 1073, 1028. ^{1}H NMR (400 MHz, $CDCl_3$) δ 2.35 (s, 3 H, CH_3), 6.48 (s, 1 H, CH-N-R), 6.84 (s, 1 H, Ar-H), 7.00 (d, *J*=8.29 Hz, 2 H, Ar-H), 7.07 - 7.10 (m, 3 H, Ar-H), 7.16 (d, *J*=7.88 Hz, 2 H, Ar-H), 7.31 - 7.37 (m, 3 H, Ar-H), 7.40 (s, 1 H, CH-N). ^{13}C NMR (101 MHz, $CDCl_3$) δ 21.06 (CH_3), 64.82 (CH-N-R), 119.35 (CH-N), 127.86 (CH), 128.00 (2xCH), 128.23 (CH-N), 128.78 (2xCH), 129.04 (2xCH), 129.49 (2xCH), 135.99 (C-CH_3), 137.27 (C), 138.24 (C), 139.28 (CH-N). HRMS (EI): found 249.1386 $(M+H)^+$; $C_{17}H_{17}N_2$ requires 249.1392.

4-((1*H*-Imidazol-1-yl)(phenyl)methyl)phenyl acetate (20h): As per general method D, compound **12h** (1 eq, 0.62 mmol, 0.15 g) was reacted with CDI in ACN (50 mL) at reflux for 3 h. The crude product was then purified *via* flash chromatography (eluent: *n*-hexane/ethyl acetate 1:1), white solid, 64%, 0.116 g, Mp. 164-167 °C. IR: ν_{max} (ATR) cm^{-1}: 3114, 2945, 1611, 1593, 1510, 1498, 1451, 1353, 1257, 1222, 1172, 1082. ^{1}H NMR (400 MHz, $CDCl_3$) δ 7.37 (s, 1H, CH-N), 7.32 (dd, *J* = 5.4, 3.4 Hz, 3H, Ar-H), 7.10 (s, 1H, CH-N), 7.05 (dd, *J* = 7.5, 1.8 Hz, 2H, Ar-H), 6.93 – 6.89 (m, 2H, Ar-H), 6.87 (s, 1H, CH-N), 6.84 – 6.79 (m, 2H, Ar-H), 6.42 (s, 1H, CH-N-R), 1.24 (s, 3H, CH_3). ^{13}C NMR

(101 MHz, $CDCl_3$) δ 157.81 (C=O), 139.34 (CH, C), 136.97 (C), 129.50 (2xCH), 129.05 (CH), 128.80 (2xCH), 128.22 (CH), 127.63 (2xCH), 119.82 (CH), 116.14 (2xCH), 64.99 (CH-N-R), 29.67 (CH_3). HRMS (EI): found 293.1277 $(M+H)^+$; $C_{18}H_{17}N_2O_2$ requires 293.1290.

4-((1*H*-Imidazol-1-yl)(phenyl)methyl)phenyl 2,2,2-trifluoroacetate (20i): As per general method D, compound **12j** (1 eq, 0.42 mmol, 0.125 g) was reacted with CDI in ACN (20 mL) at reflux for 3 h. The crude product was then purified *via* flash chromatography (eluent: *n*-hexane/ethyl acetate 1:1), off white solid, 50%, 0.07 g, Mp. 210-212°C. IR: ν_{max} (ATR) cm^{-1}: 3121, 3072, 3039, 2564, 1702, 1610, 1554, 1415, 1251, 1212, 1150, 1080. ^{1}H NMR (400 MHz, $CDCl_3$) δ 7.59 (d, *J* = 8.6 Hz, 2H, Ar-H), 7.35 (dd, *J* = 5.1, 1.8 Hz, 4H, Ar-H), 7.10 (s, 1H, CH-N), 7.09 – 7.04 (m, 4H, Ar-H), 6.86 (s, 1H, CH-N), 6.50 (s, 1H, CH-N-R). ^{13}C NMR (101 MHz, $CDCl_3$) δ 155.21 (C), 138.35 (C, CH-N), 136.30 (CH-N), 136.18 (C), 129.02 (2xCH), 128.76 (2xCH), 128.67 (CH), 127.90 (2xCH), 121.35 (2xCH), 119.74 (CH-N), 114.39 (CF_3), 64.71 (CH-N-R). LRMS (EI): found 346.16 $(M)^+$; $C_{18}H_{13}F_3N_2O_2$ requires 346.09.

1-((4-(Benzyloxy)phenyl)(phenyl)methyl)-1*H*-imidazole (20j): As per general method D, compound **12k** (1 eq, 1.85 mmol, 0.54 g) was reacted with CDI in ACN (50 mL) at reflux for 3 h. The crude product was then purified *via* flash chromatography (eluent: *n*-hexane/ethyl acetate 1:1 MeOH), yellow oil, 45%, 0.63 g. IR: ν_{max} (ATR) cm^{-1}: 3071, 2566, 1701, 1510, 1281, 1248, 1214, 1150, 1078, 1026. ^{1}H NMR (400 MHz, $CDCl_3$) δ 5.07 (s, 2 H, CH_2), 6.48 (s, 1 H, CH-N-R), 6.85 (s, 1 H, CH-N), 6.96 (d, *J*=8.54 Hz, 2 H, Ar-H), 7.02 - 7.12 (m, 5, Ar-H), 7.31 - 7.46 (m, 9 H, Ar-H). ^{13}C NMR (101 MHz, $CDCl_3$) δ 158.77 (C-OBn), 139.35 (C), 137.20 (CH-N), 136.59 (C), 131.15 (C), 129.47 (4xCH), 128.82 (2xCH), 128.61 (CH), 128.26 (CH), 128.07 (CH-N), 127.72 (2xCH), 127.44 (2xCH), 119.37 (CH-N), 115.10 (2xCH), 70.08 (CH_2), 64.61 (CH-N-R). HRMS (EI): found 341.1650 $(M+H)^+$; $C_{23}H_{21}N_2O$ requires 341.1654.

1-((4-Ethoxyphenyl)(phenyl)methyl)-1*H*-imidazole (20k) : As per general method D, compound **12m** (1 eq, 2.05 mmol, 0.47 g) was reacted with CDI in ACN (50 mL) at reflux for 1.5 h. The crude product was then purified *via* flash chromatography (eluent: *n*-

hexane/ethyl acetate 1:1), pale yellow oil, 54%, 0.308 g. IR: ν_{max} (ATR) cm^{-1}: 3031, 2928, 1610, 1583, 1510, 1494, 1477, 1281, 1246, 1175, 1044, 921, 873, 786, 734, 698, 662. ^{1}H NMR (400 MHz, $CDCl_3$) δ 1.42 (t, *J*=7.02 Hz, 3 H, CH_3), 4.03 (q, *J*=7.12 Hz, 2 H, CH_2), 6.47 (s, 1 H, CH-N-R), 6.85 (d, *J*=6.71 Hz, 2 H, Ar-H), 6.88 (s, 1 H, CH-N), 7.03 (d, *J*=8.54 Hz, 2 H, Ar-H), 7.07 - 7.10 (m, 3 H, Ar-H), 7.32 - 7.37 (m, 3 H, Ar-H), 7.40 (s, 1 H, CH-N). ^{13}C NMR (101 MHz, $CDCl_3$) δ 14.77 (CH_3), 63.52 (CH_2), 64.51 (CH-N-R), 114.67 (2xCH), 119.29 (CH-N), 127.72 (CH), 128.17 (2xCH), 128.77 (2xCH), 129.26 (CH-N), 129.44 (2xCH), 130.87 (C), 137.35 (C), 139.62 (CH-N), 158.91 (COEt). HRMS (EI): found 279.1497 $(M+H)^+$; $C_{18}H_{19}N_2O$ requires 279.1497.

1-((4-Propoxyphenyl)(3,4,5-trimethoxyphenyl)methyl)-1*H*-imidazole (21a): As per general method D, compound **15a** (1 eq, 1.35 mmol, 0.45 g) was reacted with CDI in ACN (50 mL) at reflux for 3 h. The crude product was then purified *via* flash chromatography (*n*-hexane : ethyl acetate), yellow oil**,** 56%, 0.288 g., (HPLC 99%). IR: ν_{max} (ATR) cm^{-1}: 2964, 2937, 2878, 1590, 1506, 1459, 1330, 1176. ^{1}H NMR (400 MHz, $CDCl_3$) δ 1.04 (t, *J*=7.53 Hz, 3 H, CH_3), 1.78 - 1.85 (m, 2 H, CH_2), 3.74 (s, 6 H, OCH_3), 3.85 (s, 3 H, OCH_3), 3.92 (t, *J*=6.53 Hz, 2 H, CH_2), 6.27 (s, 2 H, Ar-H), 6.40 (s, 1 H, CH-N-R), 6.84 - 6.91 (m, 3 H, Ar-H), 7.04 (d, *J*=8.53 Hz, 2 H, Ar-H), 7.09 (s, 1 H, CH-N), 7.40 (s, 1 H, CH-N). ^{13}C NMR (101 MHz, $CDCl_3$) δ 10.43 (CH_3), 22.46 (CH_2), 56.04 (2xOCH_3), 60.79 (OCH_3), 64.53 (CH-N-R), 69.51 (CH_2), 104.84 (2xCH), 114.64 (2xCH), 119.27 (CH-N), 129.14 (CH-N), 129.29 (2xCH and C), 130.57 (C), 135.12 (C-O), 137.66 (CH-N), 153.39 (2xC-O), 159.11 (C-OPr). HRMS (EI): found 381.1819 $(M-H)^+$; $C_{22}H_{25}N_2O_4$ requires 381.1814.

1-(*p*-Tolyl(3,4,5-trimethoxyphenyl)methyl)-1*H*-imidazole (21b): As per general method D, compound **15c** (1 eq, 2.71 mmol, 0.78 g) was reacted with CDI in ACN (50 mL) at reflux for 3 h. The crude product was then purified *via* flash chromatography (eluent: *n*-hexane/ethyl acetate 1:1), yellow oil**,** 39%, 0.354 g, (HPLC 98%). IR: ν_{max} (ATR) cm^{-1}: 3111, 2934, 1590, 1506, 1459, 1421, 1329, 1127, 1184, 1124, 1076, 1004. ^{1}H NMR (400 MHz, $CDCl_3$) δ 2.35 (s, 3 H, CH_3), 3.72 (s, 6 H, OCH_3), 3.83 (s, 3 OCH_3), 6.26 (s, 2 H, Ar-H), 6.43 (s, 1 H, CH-N-R), 6.84 (s, 1H, CH-N), 7.07 - 7.12 (m, 2 H, Ar-H), 7.32 - 7.37 (m, 3 H, Ar-H), 7.40 (s, 1 H, CH-N). ^{13}C NMR (101 MHz, $CDCl_3$) δ

22.31 (CH_3), 56.18 (2xOCH_3), 60.84 (OCH_3), 61.89 (CH-N-R), 103.60 (2xCH), 127.97 (2xCH), 129.53 (2xCH), 129.74 (CH-N), 131.00 (C), 134.91 (C), 136.19 (C-CH_3), 137.01 (C-O), 137.76 (CH-N), 153.48 (2xC-O), HRMS (EI): 339.1712 $(M+H)^+$; $C_{20}H_{23}N_2O_3$ requires 339.1709.

1-((3,4-Dimethoxyphenyl)(3,4,5-trimethoxyphenyl)methyl)-1*H*-imidazole (21c): As per general method D, compound **15d** (1 eq, 3.29 mmol, 1.1 g) was reacted with CDI in ACN (50 mL) at reflux for 3 h. The crude product was then purified via flash chromatography (eluent: *n*-hexane/ethyl acetate: methanol 1:9:1), pale yellow solid, 67%, 0.85 g, Mp. 134-137 °C, (HPLC 96%). IR: ν_{max} (ATR) cm^{-1}: 2928, 2838, 1516, 1491, 1477, 1331, 1276, 1257, 1215, 1235, 1120, 1024, 1004. ^1H NMR (400 MHz, $CDCl_3$) δ 3.74 (s, 6 H, OCH_3), 3.79 (s, 3 H, OCH_3), 3.85 (s, 3 H, OCH_3), 3.88 (s, 3 H, OCH_3), 6.28 (s, 2 H, Ar-H), 6.39 (s, 1 H, CH-N-R), 6.61 - 6.66 (m, 2 H, Ar-H), 6.83 (s, 1 H, CH-N), 6.85 (d, *J*=3.73 Hz, 1 H, Ar-H), 7.09 (s, 1 H, CH-N), 7.41 (s, 1 H, CH-N). ^{13}C NMR (101 MHz, $CDCl_3$) δ 55.88 (OCH_3), 55.91 (2xOCH_3), 56.09 (OCH_3), 60.84 (OCH_3), 64.74 (CH-N-R), 104.97 (2xCH), 111.02 (CH), 111.15 (CH), 119.29 (CH-N), 120.51 (CH), 129.28 (CH-N), 131.24 (C), 134.88 (C), 137.37 (C-O), 137.79 (CH-N), 149.05 (C-O), 149.17 (C-O), 153.43 (2xC-O). HRMS (EI): Found 419.1387 $(M+Cl)^+$; $C_{21}H_{24}{}^{35}ClN_2O_5$ requires 419.1374.

1-(Phenyl(3,4,5-trimethoxyphenyl)methyl)-1*H*-imidazole (21d): As per general method D, compound **15e** (1 eq, 1.20 mmol, 0.33 g) was reacted with CDI in ACN (50 mL) at reflux for 3 h. The crude product was then purified *via* flash chromatography (eluent: *n*-hexane/ethyl acetate 1:1), off-white solid, 52%, 0.205 g, Mp. 83-85°C, (HPLC 97%). IR: ν_{max} (ATR) cm^{-1}: 2964, 2937, 2838, 1590, 1459, 1330, 1234, 1004. ^1H NMR (400 MHz, $CDCl_3$) δ 3.75 (s, 6 H, OCH_3), 3.86 (s, 3 H, OCH_3), 6.30 (s, 2 H, Ar-H), 6.45 (s, 1 H, CH-N-R), 6.87 (s, 1 H, CH-N), 7.10 - 7.15 (m, 3 H, Ar-H), 7.35 - 7.39 (m, 3 H, Ar-H), 7.42 (s, 1 H, CH-N). ^{13}C NMR (101 MHz, $CDCl_3$) δ 56.08 (2xOCH_3), 60.84 (OCH_3), 65.05 (CH-N-R), 105.20 (2xCH), 119.35 (CH-N), 127.94 (CH), 128.45 (CH-N), 128.84 (2xCH), 129.26 (2xCH), 134.57 (C), 137.37 (C), 137.85 (C), 138.89 (CH-N), 153.45 (2xC). HRMS (EI): found 323.1400 $(M-H)^+$; $C_{19}H_{19}N_2O_3$ requires 323.1396.

1-((4-Methoxyphenyl)(3,4,5-trimethoxyphenyl)methyl)-1*H*-imidazole (21e): As per general method D, compound **15f** (1 eq, 1.84 mmol, 0.56 g) was reacted with CDI in ACN (50 mL) at reflux for 2 h. The crude product was then purified *via* flash chromatography (eluent: *n*-hexane/ethyl acetate: methanol 1:9:1), orange oil, 100%, 0.65 g, (HPLC 92%). IR: ν_{max} (ATR) cm^{-1}: 3358, 2964, 2937, 1590, 1459, 1330, 1176, 1123, 1072. ^1H NMR (400 MHz, $CDCl_3$) δ 3.74 (s, 6 H, OCH_3), 3.82 (s, 3 H, OCH_3), 3.85 (s, 3 H, OCH_3), 6.27 (s, 2 H, Ar-H), 6.40 (s, 1 H, CH-N-R), 6.85 (s, 1 H, CH-N), 6.88 - 6.91 (m, 2 H, Ar-H), 7.04 - 7.07 (m, 2 H, Ar-H), 7.09 (s, 1 H, CH-N), 7.40 (s, 1 H, CH-N). ^{13}C NMR (101 MHz, $CDCl_3$) δ 55.25 (OCH_3), 56.04 (2xOCH_3), 60.80 (OCH_3), 64.50 (CH-N-R), 104.85 (2xCH), 114.13 (2xCH), 119.27 (CH-N), 127.82 (CH-N), 129.17 (C), 129.32 (2xCH), 130.83 (C), 135.05 (C-O), 137.68 (CH-N), 153.40 (2xC-O), 159.52 (C-O). HRMS (EI): Found 355.1664 $(M+H)^+$; $C_{20}H_{23}N_2O_4$ requires 355.1658.

1-((4-Fluorophenyl)(3,4,5-trimethoxyphenyl)methyl)-1*H*-imidazole (21f): As per general method D, compound **15g** (1 eq, 1.48 mmol, 0.43 g) was reacted with CDI in ACN (50 mL) at reflux for 3 h. The crude product was then purified *via* flash chromatography (eluent *n*-hexane/ethyl acetate 1:1), yellow oil, 56%, 0.28 g, (HPLC 91%). IR: ν_{max} (ATR) cm^{-1}: 2939, 2838, 1590, 1505, 1458, 1420, 1223, 1122. ^1H NMR (400 MHz, $CDCl_3$) δ 3.75 (s, 6 H, OCH_3), 3.86 (s, 3 H, OCH_3), 6.27 (s, 2 H, Ar-H), 6.45 (s, 1 H, CH-N-R), 6.85 (s, 1 H, CH-N), 7.07 - 7.13 (m, 5 H, Ar-H), 7.42 (s, 1 H, CH-N). ^{13}C NMR (101 MHz, $CDCl_3$) δ 56.13 (2xOCH_3), 60.87 (OCH_3), 64.36 (CH-N-R), 105.06 (2xCH), 115.97 (2xCH), 119.21 (CH-N), 129.48 (CH-N), 129.72 (CH), 129.80 (CH), 134.39 (C), 134.83 (C), 137.28 (C-O), 137.99 (CH-N), 153.56 (2xC-O), 163.77 (C-F). HRMS (EI): Found 343.1445 $(M+H)^+$; $C_{19}H_{20}FN_2O_3$ requires 343.1458.

4-((1*H*-Imidazol-1-yl)(3,4,5-trimethoxyphenyl)methyl)benzonitrile (21g): As per general method D, compound **15h** (1 eq, 1.50 mmol, 0.45 g) was reacted with in ACN (50 mL) at reflux for 3 h. The crude product was then purified *via* flash chromatography (eluent: *n*-hexane/ethyl acetate 1:1), orange oil**,** 35%, 0.182 g, (HPLC 94%). IR: ν_{max} (ATR) cm^{-1}: 3120, 2938, 2839, 2227, 1589, 1461, 1327, 1230, 1185, 1124. ^1H NMR (400 MHz, $CDCl_3$) δ 3.76 (s, 6 H, OCH_3), 3.87 (s, 3 H, OCH_3), 6.28 (s, 2 H, Ar-H), 6.50 (s, 1 H, CH-N-R), 6.85 (s, 1 H, CH-N), 7.15 (s, 1 H, CH-N), 7.21 (d, *J*=8.03 Hz, 2 H, Ar-H),

7.44 (s, 1 H, CH-N), 7.69 (d, *J*=8.53 Hz, 2 H, Ar-H). ^{13}C NMR (101 MHz, $CDCl_3$) δ 56.23 (2xOCH_3), 60.92 (OCH_3), 64.63 (CH-N-R), 105.53 (2xCH), 112.57 (C-CN), 118.15 (CN), 119.11 (CH-N), 128.48 (2xCH), 129.96 (CH-N), 132.71 (2xCH), 132.94 (C), 137.27 (C-O), 138.48 (CH-N), 144.38 (C), 153.78 (2xC-O). HRMS (EI): Found 348.1354 $(M-H)^+$; $C_{20}H_{18}N_3O_3$ requires 348.1348.

1-((4-Ethoxyphenyl)(3,4,5-trimethoxyphenyl)methyl)-1*H*-imidazole (21j): As per general method D, compound (**18c**) (1 eq, 1.55 mmol, 0.81 g) was reacted with CDI in ACN (50 mL) at reflux for 3 h. The crude product was then purified *via* flash chromatography (eluent: *n*-hexane/ethyl acetate 1:1) to afford an orange oil, 99%, 0.564 g, (HPLC 91%). IR: ν_{max} (ATR) cm^{-1}: 2978, 2937, 1610, 1590, 1506, 1458, 1419, 1330, 1234, 1074, 1043. ^1H NMR (400 MHz, $CDCl_3$) δ 1.42 (t, *J*=6.85 Hz, 3 H, CH_3), 3.74 (s, 6 H, OCH_3), 3.85 (s, 3 H, OCH_3), 4.04 (q, *J*=6.85 Hz, 2 H, CH_2), 6.27 (s, 2 H, Ar-H), 6.40 (s, 1 H, CH-N-R), 6.85 - 6.90 (m, 3 H, Ar-H), 7.04 (d, *J*=8.80 Hz, 2 H, Ar-H), 7.09 (br. s., 1 H, CH-N), 7.40 (s, 1 H, CH-N). ^{13}C NMR (101 MHz, $CDCl_3$) δ 14.73 (CH_3), 56.08 (2xOCH_3), 60.84 (OCH_3), 63.51 (CH_2), 64.56 (CH-N-R), 104.88 (2xCH), 114.65 (2xCH), 129.22 (CH-N) 129.34 (2xCH, C), 130.67 (C), 135.13 (C), 137.70 (CH-N), 153.42 (2xC), 158.95 (C). HRMS (EI): Found 369.1800 $(M+H)^+$; $C_{21}H_{25}N_2O_4$ requires 369.1814.

1-(Benzo[d][1,3]dioxol-5-yl(3,4,5-trimethoxyphenyl)methyl)-1*H*-imidazole (21k): As per general method D, compound **18d** (1 eq, 0.5 mmol, 0.16 g) was treated with CDI in ACN (50 mL) at reflux for 3 h. The crude product was then purified via flash chromatography (eluent: *n*-hexane/ethyl acetate) to afford a yellow oil, 52%, 0.093 g, (HPLC 96%). IR: ν_{max} (ATR) cm^{-1}: 3114, 2938, 1590, 1502, 1488, 1459, 1330, 1232, 1121, 1075, 1034. ^1H NMR (400 MHz, $CDCl_3$) δ 3.73 (s, 6 H, OCH_3), 3.83 (s, 3 H, OCH_3), 5.97 (s, 2 H, CH_2), 6.25 (s, 2 H, Ar-H), 6.34 (s, 1 H, CH-N-R), 6.58 - 6.61 (m, 2 H, Ar-H), 6.77 (d, *J*=8.71 Hz, 1 H, Ar-H), 6.92 (br. s., 1 H, CH-N), 7.14 (br. s., 1 H, CH-N), 7.47 (br. s., 1 H, CH-N). ^{13}C NMR (101 MHz, $CDCl_3$) δ 153.52 (2xC-O), 148.20 (C-O), 147.77 (C-O), 137.91 (C-O, CH-N), 134.58 (2xC), 132.47 (CH), 121.81 (CH-N), 108.49 (CH), 108.41 (CH), 104.89 (2xCH), 101.44 (CH_2), 65.00 (CH-N), 60.86 (OCH_3), 56.14 (2xOCH_3). HRMS (EI): Found 369.1442 $(M+H)^+$; $C_{20}H_{21}N_2O_5$ requires 369.1450.

General method E for the preparation of diarylmethylpyrrolidines, diarylmethylpiperidines and diarylmethylpiperazines: The benzhydryl alcohol (1 eq) was reacted with thionyl chloride (5 eq) in dry DCM (30 mL) for 12 h. The reaction mixture was concentrated under reduced pressure and the crude product was used in the next step without any further purification. The chlorinated benzhydryl alcohol was reacted with pyrrolidine or piperidine (5 eq) in dry ACN (30 mL) and refluxed for 12 h. The solvent was removed and the residue dissolved in DCM (50 mL) and washed with 1 M NaOH (30 mL). The organic phase was dried over sodium sulphate, filtered and concentrated. The crude product was then purified *via* flash chromatography (eluent: *n*-hexane/ethyl acetate).

1-((4-Fluorophenyl)(phenyl)methyl)pyrrolidine (25c): As per general method E, compound **12d** (1 eq, 3.3 mmol, 0.72 g) was treated with thionyl chloride followed by reaction with pyrrolidine (5 eq, 16.54 mmol, 1.18 g 1.35 mL) in dry ACN (50 mL) at reflux for 12 h. The crude product was purified *via* flash chromatography (eluent: *n*-hexane/ethyl acetate 7:3), orange oil, 79%, 0.67 g. IR: ν_{max} (ATR) cm^{-1}: 2967, 2875, 1505, 1492, 1452, 1219, 1154, 1129, 1091, 1027. ^{1}H NMR (400 MHz, $CDCl_3$) δ 1.75 (dt, *J*=6.26, 3.28 Hz, 4 H, 2xCH_2), 2.38 (br. s., 4 H, 2xCH_2), 4.13 (s, 1 H, CH-N-R), 6.92 – 6.94 (m, 2 H, Ar-H), 7.13 - 7.18 (m, 1 H, Ar-H), 7.23 - 7.27 (m, 3 H, Ar-H), 7.40 (d, *J*=7.93 Hz, 3 H, Ar-H). ^{13}C NMR (101 MHz, $CDCl_3$) δ 23.52 (2xCH_2), 53.58 (2xCH_2), 75.61 (CH-N-R), 115.20 (2xCH), 126.87 (CH), 127.35 (2xCH), 128.41 (2xCH), 128.82 (2xCH), 136.65 (C), 143.24 (C), 162.87 (C-F). LRMS (EI): found 256.32 $(M+H)^+$; $C_{17}H_{19}FN$ requires 256.15.

1-((4-Methoxyphenyl)(3,4,5-trimethoxyphenyl)methyl)pyrrolidine (25g): As per general method E, compound **15c** was reacted with pyrrolidine (5 eq, 6.35 mmol, 0.38 g, 0.45 mL) in dry ACN (50 mL) at reflux for 12 h. The product did not require any further purification, brown oil, 91%, 0.41 g, (HPLC 92%). IR: ν_{max} (ATR) cm^{-1}: 2932, 2834, 2750, 1589, 1507, 1452, 1418, 1327, 1232, 1175, 1125, 1099, 1034, 1005. ^{1}H NMR (400 MHz, $CDCl_3$) δ 7.34 (d, *J* = 8.7 Hz, 2H, Ar-H), 6.80 (d, *J* = 8.7 Hz, 2H, Ar-H), 6.66 (s, 2H, Ar-H), 3.99 (s, 1H, CH-N-R), 3.83 (s, 6H, OCH_3), 3.76 (s, 3H, OCH_3), 3.75 (s, 3H, OCH_3), 2.43 (s, 2H, CH_2), 2.37 (s, 2H, CH_2), 1.76 (d, *J* = 2.9 Hz, 4H, CH_2). ^{13}C NMR

(101 MHz, $CDCl_3$) δ 158.45 (C-O), 153.05 (2xC-O), 136.51 (2xC), 136.05 (C-O), 128.37 (2xCH), 113.66 (2xCH), 104.04 (2xCH), 76.02 (CH-N-R), 60.73 (OCH_3), 56.08 (2xCH_2), 55.15 (OCH_3), 53.66 (2xOCH_3), 23.51 (2xCH_2). LRMS (EI): found 358.17 $(M+H)^+$; $C_{21}H_{28}NO_4$ requires 358.20.

1-((4-Methoxyphenyl)(phenyl)methyl)-4-phenylpiperazine (27a): As per general method E, compound 12e (1 eq, 2.14 mmol, 0.5 g) was initially treated with thionyl chloride (5 eq) in dry DCM (30 mL) for 12 h. followed by reaction with phenylpiperazine (5 eq, 10.74 mmol, 1.74 g 1.64 mL) in dry ACN (50 mL) and refluxed for 12 h. The crude product was purified *via* flash column chromatography (eluent: *n*-hexane/ethyl acetate 1:1), to afford a white solid, 54 %, 0.41 g, Mp. 130-145 °C [35] (HPLC 97%). IR: ν_{max} (ATR) cm^{-1}: 3056, 3026, 2933, 2806, 2766, 2038, 1784, 1640, 1608, 1583. ^1H NMR (400 MHz, $CDCl_3$) δ 2.51 - 2.57 (m, 4 H, CH_2), 3.15 - 3.21 (m, 4 H, CH_2), 3.75 (s, 3 H, OCH_3), 4.21 (s, 1 H, CH-N-R), 6.82 (d, *J*=8.29 Hz, 2 H, Ar-H), 6.89 (d, *J*=8.29 Hz, 2 H, Ar-H), 7.17 (s, 1 H, Ar-H), 7.22 - 7.29 (m, 5 H, Ar-H), 7.34 (d, *J*=8.71 Hz, 2 H, Ar-H), 7.43 (d, *J*=7.46 Hz, 2 H, Ar-H). ^{13}C NMR (101 MHz, $CDCl_3$) δ 49.20 (2xCH_2), 51.91 (2xCH_2), 55.19 (OCH_3), 75.48 (CH-N-R), 113.88 (2xCH), 115.77 (2xCH), 119.42 (CH), 126.85 (CH), 127.75 (2xCH), 128.49 (2xCH), 128.92 (2xCH), 129.03 (2xCH), 134.75 (C), 143.01 (C), 151.31 (C), 158.55 (C-O). LRMS (EI): found 359.14 $(M+H)^+$; $C_{24}H_{27}N_2O$ requires 359.21.

1-Benzyl-4-((4-methoxyphenyl)(phenyl)methyl)piperazine (27b): As per general method E, compound **12e** (1 eq, 1.63 mmol, 0.38 g) was reacted with benzylpiperazine (5 eq, 8.16 mmol, 1.43 g 1.41 mL) in dry ACN (50 mL) at reflux for 12 h. The crude product was purified *via* flash column chromatography (eluent: *n*-hexane/ethyl acetate 1:1) to afford a brown oil, 43%, 0.26 g, (HPLC 99%). IR: ν_{max} (ATR) cm^{-1}: 3060, 3026, 2933, 2806, 1608, 1508, 1451, 1244, 1173, 1134, 1031. ^1H NMR (400 MHz, $CDCl_3$) δ 2.39 - 2.56 (m, 8 H, CH_2), 3.55 (s, 2 H, CH_2), 3.77 (s, 3 H, OCH_3), 4.22 (s, 1 H, CH-N-R), 6.80 - 6.85 (m, 2 H, Ar-H), 7.15 - 7.21 (m, 1 H, Ar-H), 7.28 - 7.30 (m, 2 H, Ar-H), 7.31 - 7.35 (m, 6 H, Ar-H), 7.40 - 7.44 (m, 3 H, Ar-H). ^{13}C NMR (101 MHz, $CDCl_3$) δ 51.82 (2xCH_2), 53.33 (2xCH_2), 55.18 (OCH_3), 63.04 (CH_2), 75.48 (CH-N-R), 113.79 (2xCH), 126.74 (CH), 127.01 (CH), 127.82 (2xCH), 128.16 (2xCH), 128.40 (2xCH),

128.98 (2xCH), 129.30 (2xCH), 134.90 (C), 137.92 (C), 143.14 (C), 158.46 (C-O). LRMS (EI): found 373.15 $(M+H)^+$; $C_{25}H_{29}N_2O$ requires 373.23.

1-Benzyl-4-((4-methoxyphenyl)(3,4,5-trimethoxyphenyl)methyl)piperazine (27d): As per general method E, compound **15c** (1 eq, 0.86 mmol, 0.28 g) was treated with excess thionyl chloride, then reacted with 1-benzylpiperazine (5 eq, 3.71 mmol, 0.65 g, 0.64 mL) in dry ACN (40 mL) and refluxed for 12 h under nitrogen atmosphere. The crude product was purified *via* flash chromatography (eluent: *n*-hexane/ethyl acetate 1:1), to afford a red oil, 69%, 0.23 g, (HPLC 100%). IR: ν_{max} (ATR) cm^{-1}: 2954, 2812, 1598, 1505, 1450, 1419, 1300, 1230, 1031, 1004, 927, 839, 784, 756, 726, 697, 586, 571. ^1H NMR (400 MHz, $CDCl_3$) δ 7.28 - 7.30 (m, 7 H, Ar-H), 6.81 (d, *J* = 8.7 Hz, 2 H, Ar-H), 6.64 (s, 2 H, Ar-H), 4.09 (s, 1 H, CH), 3.82 (s, 6 H, OCH_3), 3.78 (s, 3 H, OCH_3), 3.73 (s, 3 H, OCH_3), 3.50 (s, 2 H, CH_2), 2.47 (s(br), 8 H, $4xCH_2$). ^{13}C NMR (101 MHz, $CDCl_3$) δ 158.54 (C-O), 153.11 (2xC-O), 138.86 (C), 137.81 (C), 136.58 (C-O), 134.56 (C), 129.27 (2xCH), 128.90 (2xCH), 128.14 (2xCH), 127.01 (CH), 113.75 (2xCH), 104.47 (2xCH), 75.57 (CH), 63.00 (CH_2), 60.74 (OCH_3), 56.07 ($2xOCH_3$), 55.15 (OCH_3), 53.29 ($2xCH_2$), 51.76 ($2xCH_2$). LRMS (EI): found 463.15 $(M+H)^+$; $C_{28}H_{35}N_2O_4$ requires 463.26.

1-((4-Methoxyphenyl)(3,4,5-trimethoxyphenyl)methyl)-4-(*p*-tolyl)piperazine (27f): As per general method E, compound **15c** (1 eq, 0.74 mmol, 0.24 g) was treated with excess thionyl chloride, then reacted with 1-(4-methylphenyl)piperazine (5 eq, 3.71 mmol, 0.92 g) in dry ACN (40 mL) and refluxed for 12 h under nitrogen. The crude product was purified *via* flash chromatography (eluent: *n*- hexane/ethyl acetate 1:1), to afford a yellow oil, 6%, 0.02 g, (HPLC 91%). IR: ν_{max} (ATR) cm^{-1}: 2936, 2832, 1589, 1508, 1452, 1417, 1330, 1229, 1123, 1004. ^1H NMR (400 MHz, $CDCl_3$) δ 2.25 (s, 3 H, CH_3), 2.50 - 2.59 (m, 4 H, CH_2), 3.11 - 3.14 (m, 4 H, CH_2), 3.76 (s, 3 H, OCH_3), 3.78 (s, 3 H, OCH_3), 3.83 (s, 6H, OCH_3), 4.12 (s, 1 H, CH-N-R), 6.67 (s, 2 H , Ar-H), 6.80 - 6.84 (m, 4 H, Ar-H), 7.05 (d, *J*=8.29 Hz, 2 H, Ar-H), 7.33 (d, *J*=8.71 Hz, 2 H, Ar-H). ^{13}C NMR (101 MHz, $CDCl_3$) δ 158.64 (C), 153.19 (2xC), 138.76 (C), 136.65 (C), 134.43 (C), 129.57 (2xCH), 128.89 (2xCH), 116.10 (2xCH), 113.85 (2xCH), 104.39 (2xCH), 75.56 (CH-N-R), 60.76 (OCH_3), 56.07 ($2xOCH_3$), 55.18 (OCH_3), 51.88 ($2xCH_2$), 49.76 ($2xCH_2$), 20.38 (CH_3). LRMS (EI): found 463.13 $(M+H)^+$; $C_{28}H_{35}N_2O_4$ requires 463.26.

2-Methoxyphenyl 2-chloroacetate (22c): To a chilled solution of 2-methoxyphenol (1 eq, 12.08 mmol, 1.5 g) and triethylamine (12.08 mmol, 1.22 g, 1.7 mL) in diethyl ether (50 mL) chloroacetyl chloride (1.1 eq, 13.28 mmol, 1.5 g 1.07 mL) was added and the mixture was stirred at RT for 1 h. After completion the mixture was diluted with more diethyl ether and washed with HCl 1M (20 mL) and $NaHCO_3$ (20 mL) dried over sodium sulphate, and concentrated. No further purification was required. Yield: 78% (1.88 g) brown solid Mp: 65-69 °C [26]. ^{1}H NMR (400 MHz, $CDCl_3$) δ 7.22 (dd, J = 1.6, 0.8 Hz, 1H, Ar-H), 7.05 (dd, J = 7.9, 1.7 Hz, 1H, Ar-H), 6.96 (td, J = 7.7, 1.3 Hz, 2H, Ar-H), 4.33 (s, 2H, CH_2), 3.82 (s, 3H, OCH_3). ^{13}C NMR (101 MHz, $CDCl_3$) δ 165.40 (C=O), 150.79 (C-O), 139.28 (C-O), 127.39 (CH), 122.37 (CH), 120.76 (CH), 112.50 (CH), 55.85 (OCH_3), 40.62 (CH_2). LRMS (EI): found 201.16 $(M+H)^+$; $C_9H_7{}^{35}ClO_3$ requires 201.03. IR: ν_{max} (ATR) cm^{-1}: 2948, 1769, 1639, 1605, 1584, 1501, 1443, 1409, 1314, 1281, 1258, 1229, 1173, 1013, 1002, 955, 925, 872, 833, 687, 633, 576.

2-Methoxy-5-(3,4,5-trimethoxybenzoyl)phenyl 2-chloroacetate (23c): 2-Methoxyphenyl 2-chloroacetate **(22c)** (1 eq, 4.6 mmol, 0.921 g) was reacted with 3,4,5-trimethoxybenzoic acid (1.5 eq, 6.8 mmol, 1.44 g) in Eaton's reagent (0.65 g P_2O_5 / 3.8 mL CH_3SO_3H). The mixture was stirred at 60 °C for 6 h under N_2. The product was diluted in DCM (60 mL) and poured into a separatory funnel containing $NaHCO_3$ 50% (40 mL) and extracted. The crude product was purified *via* flash chromatography (eluent: *n*-hexane/ethyl acetate 3:7). Yield: 17% (0.63 g) white crystals Mp: 158-161°C [26]. ^{1}H NMR (400 MHz, $CDCl_3$) δ 7.77 (dd, J = 8.6, 2.1 Hz, 1H, Ar-H), 7.60 (d, J = 2.1 Hz, 1H, Ar-H), 7.05 (d, J = 8.6 Hz, 1H, Ar-H), 7.01 (s, 2H Ar-H), 4.34 (s, 2H, CH_2), 3.92 (s, 3H, OCH_3), 3.92 (s, 3H, OCH_3), 3.87 (s, 6H, OCH_3). ^{13}C NMR (101 MHz, $CDCl_3$) δ 193.50 (C=O), 165.35 (C=O), 154.40 (C-O), 152.88 (C-O), 141.80 (2xC-O), 138.71 (C), 132.62 (C), 130.36 (C), 130.18 (CH), 124.79 (CH), 111.67 (CH), 107.39 (2xCH), 60.94 (OCH_3), 56.30 (2xOCH_3), 56.19 (OCH_3), 40.48 (CH_2). LRMS (EI): found 394.26 $(M+Na)^+$; $C_{19}H_{19}{}^{35}ClO_7$ requires 394.14. IR: ν_{max} (ATR) cm^{-1}: 3067, 2939, 1783, 1639, 1604, 1581, 1505, 1460, 1443, 1408, 1229, 1108, 1166, 1020, 953, 870, 832, 817, 760, 612.

(3-Hydroxy-4-methoxyphenyl)(3,4,5-trimethoxyphenyl)methanone, Phenstatin (7a): 2-Methoxy-5-(3,4,5-trimethoxybenzoyl)phenyl 2-chloroacetate **(23c)** (1 eq, 1.28 mmol, 0.51 g) was reacted with sodium acetate (4.5 eq, 5.76 mmol, 0.47 g) in methanol (10 mL)

at reflux for 2 h. After cooling the mixture was concentrated under reduced pressure. Distilled water was added to the residue and the resulting precipitate was filtered and recrystallized from ethanol. Yield: 89% (0.361 g) white solid Mp: 152-156 °C [26]. ^{1}H NMR (400 MHz, $CDCl_3$) δ 7.42 (d, *J* = 2.1 Hz, 1H, Ar-H), 7.37 (dd, *J* = 8.3, 2.1 Hz, 1H, Ar-H), 7.01 (s, 2H, Ar-H), 6.90 (d, *J* = 8.4 Hz, 1H, Ar-H), 3.96 (s, 3H, OCH_3), 3.91 (s, 3H, OCH_3), 3.86 (s, 6H, OCH_3). ^{13}C NMR (101 MHz, $CDCl_3$) δ 194.63 (C=O), 152.75 (2xC-O), 150.17 (C-O), 145.29 (C-OH), 141.61 (C-O), 133.13 (C), 131.03 (C), 123.61 (CH), 116.19 (CH), 109.66 (CH), 107.49 (2xCH), 60.93 (OCH_3), 56.28 (2xOCH_3), 56.07 (OCH_3). LRMS (EI): found 319.37 $(M+H)^+$; $C_{17}H_{19}O_6$ requires 319.12. IR: ν_{max} (ATR) cm^{-1}: 3249, 3003, 2840, 1632, 1578, 1505, 1443, 1414, 1331, 1236, 1222, 1118, 1002, 931, 892, 870, 758, 736, 670, 639, 576.

5-(Hydroxy(3,4,5-trimethoxyphenyl)methyl)-2-methoxyphenol (15i) : As per general method A, compound **(23c)** (1 eq, 1.10 mmol, 0.35 g) was dissolved in methanol and cooled to 0°C. Sodium borohydride (2 eq, 2.2 mmol, 0.08 g) was added in small portions and the mixture was stirred until the reaction was complete from TLC. HCl (10%) was added and the solvent was evaporated under reduced pressure. The product was re-dissolved in ethyl acetate (30 mL) and washed with water (20 mL) and brine (10 mL), dried over sodium sulphate, filtered and concentrated under reduced pressure. Yield: 96% (0.33 g) white solid Mp: 143-146 °C [22]. ^{1}H NMR (400 MHz, $CDCl_3$) δ 6.91 (d, *J* = 1.6 Hz, 1H, Ar-H), 6.83 (d, *J* = 1.8 Hz, 1H, Ar-H), 6.82 (s, 1H, Ar-H), 6.56 (s, 2H, Ar-H), 5.07 (s, 1H, CH-OH), 3.87 (s, 3H, OCH_3), 3.83 (s, 6H, OCH_3), 3.81 – 3.81 (m, 3H, OCH_3). ^{13}C NMR (101 MHz, $CDCl_3$) δ 153.18 (2xC-O), 145.98 (C-O), 145.53 (C-O), 137.83 (C-O), 137.07 (C), 135.22 (C), 118.59 (CH), 113.23 (CH), 110.38 (CH), 103.60 (2xCH), 85.05 (CH), 60.77 (OCH_3), 56.97 (2xOCH_3), 56.06 (OCH_3). LRMS (EI): Found 343.36 $(M+Na)^+$; $C_{17}H_{20}NaO_6$ requires 343.12. IR: ν_{max} (ATR) cm^{-1}:3431, 2937, 2837, 1590, 1507, 1459, 1438, 1420, 1327, 1264, 1225, 1119, 1086, 1001, 950, 910, 883, 824, 785, 761, 705, 627, 559.

General method D: 1,1-Diarylmethylpyrrolidines and piperidines

The reduced benzhydryl alcohols (1 eq) were reacted with thionyl chloride (5 eq) in dry DCM (30 mL) for 12 h. The reaction mixture was concentrated under reduced pressure and the crude product was used in the next step without any further purification. The

chlorinated benzhydryl alcohol was reacted with pyrrolidine or piperidine (5 eq) in dry ACN (30 mL) and refluxed for 12 h. The solvent was removed and the residue dissolved in DCM (50 mL) and washed with 1 M NaOH (30 mL). The organic phase was dried over sodium sulphate, filtered and concentrated. The crude product was then purified *via* flash chromatography in *n*-hexane/ethyl acetate.

1-((4-Fluorophenyl)(phenyl)methyl)piperidine (26a): As per general method D, chlorinated (4-fluorophenyl)(phenyl)methanol (**12d**) (1 eq, 3.12 mmol, 0.69 g) of was reacted with piperidine (5 eq, 15.63 mmol, 1.33 g, 1.54 mL) in dry ACN (50 mL) at reflux for 12 h. The product did not require any further purification. Yield: 85% (0.7 g) orange solid Mp: 81-85 °C [27]. ^{1}H NMR (400 MHz, $CDCl_3$) δ 1.37 - 1.46 (m, 2 H, CH_2), 1.52 - 1.58 (m, 4 H, CH_2), 2.29 (br. s., 4 H, CH_2), 4.21 (s, 1 H, CH)-N-R, 6.90 - 6.97 (m, 2 H, Ar-H), 7.14 - 7.19 (m, 1 H, Ar-H), 7.24 - 7.28 (m, 2 H, Ar-H), 7.31 - 7.39 (m, 4 H, Ar-H). ^{13}C NMR (101 MHz, $CDCl_3$) δ 24.65 (CH_2), 26.23 (2xCH_2), 53.05 (2xCH_2), 75.82 (CH-N-R), 114.96 (CH), 115.17 (CH), 126.77 (CH), 127.91 (2xCH), 128.35 (2xCH), 129.31 (CH), 129.39 (CH), 138.96 (C), 142.98 (C), 162.83 (C-F). HRMS (EI): found 270.1666 $(M+H)^+$; $C_{18}H_{21}FN$ requires 270.1658. IR: ν_{max} (ATR) cm^{-1}: 2962, 2932, 2789, 2752, 1604, 1506, 1491, 1450, 1388, 1303, 1223, 1162, 1109, 1092, 1066, 991, 873, 847, 796, 720, 695.

1-((4-Methoxyphenyl)(phenyl)methyl)piperidine (26c): As per general method D, chlorinated (4-methoxyphenyl)(phenyl)methanol **(12e) (**1 eq, 2.44 mmol, 0.57 g) was reacted with piperidine (5 eq, 12.24 mmol, 1.04 g 1.2 mL) in dry ACN (50 mL) at reflux for 12 h. The product did not require any further purification. Yield: 91 % (0.62 g) brown oil [28]. ^{1}H NMR (400 MHz, $CDCl_3$) δ 7.38 (dd, *J* = 8.2, 1.1 Hz, 2H, Ar-H), 7.29 (d, *J* = 8.7 Hz, 2H, Ar-H), 7.26 - 7.22 (m, 2H, Ar-H), 7.16 (d, *J* = 7.3 Hz, 1H, Ar-H), 6.80 (d, *J* = 8.8 Hz, 2H, Ar-H), 4.17 (s, 1H, CH-N-R), 3.74 (s, 3H, OCH_3), 2.30 (s, 4H, CH_2), 1.57 – 1.53 (m, 4H, CH_2), 1.44 – 1.40 (m, 2H, CH_2). ^{13}C NMR (101 MHz, $CDCl_3$) δ 24.70 (CH_2), 26.25 (2xCH_2), 53.09 (2xCH_2), 55.15 (OCH_3), 75.95 (CH-N-R), 113.63 (2xCH), 126.51 (CH), 127.85 (2xCH), 128.25 (2xCH), 128.99 (2xCH), 135.33 (C), 143.59 (C), 158.30 (C-O). LRMS (EI): found 282.15 $(M+H)^+$; $C_{19}H_{24}NO$ requires 281.18. IR: ν_{max} (ATR) cm^{-1}: 3026, 2930, 2852, 2750, 1609, 1508, 1493, 1451, 1440, 1300, 1243, 1212, 1174, 1034, 992, 872, 843, 817, 738, 698, 601, 570.

1-((4-Nitrophenyl)(phenyl)methyl)pyrrolidine (25a): As per general method D, chlorinated (4-nitrophenyl)(phenyl)methanol **(12b)** (1 eq, 4.53 mmol, 1.24 g) was reacted with pyrrolidine (5 eq, 22.69 mmol, 1.61 g 1.86 mL) in dry ACN (50 mL) at reflux for 12 h. The crude product was purified *via* flash chromatography (eluent: *n*-hexane/ethyl acetate 7:3). Yield: 23% (0.29 g) orange solid Mp: 70-72 ° C [29]. ^{1}H NMR (400 MHz, $CDCl_3$) δ 1.75 - 1.80 (m, 4 H, $2xCH_2$), 2.37 - 2.44 (m, 4 H, $2xCH_2$), 4.26 (s, 1 H, CH-N-R), 7.17 - 7.19 (m, 1 H, Ar-H), 7.27 (d, *J*=7.32 Hz, 2 H, Ar-H), 7.40 (d, *J*=7.32 Hz, 2 H, Ar-H), 7.63 (d, *J*=8.55 Hz, 2 H, Ar-H), 8.12 (d, *J*=9.16 Hz, 2 H, Ar-H). ^{13}C NMR (101 MHz, $CDCl_3$) δ 23.54 ($2xCH_2$), 53.46 ($2xCH_2$), 75.72 (CH), 123.81 (2xCH), 127.48 (2xCH), 128.12 (4xCH), 128.70 (CH), 146.81 (2xC), 151.83 ($C-NO_2$). HRMS (EI): found 283.1441 $(M+H)^+$; $C_{17}H_{19}N_2O_2$ requires 283,1446. IR: ν_{max} (ATR) cm^{-1}: 3057, 2963, 2768, 1606, 1592, 1518, 1488, 1450, 1341, 1194, 1128, 920, 756, 742, 691.

1-((4-Methoxyphenyl)(phenyl)methyl)pyrrolidine (**25d**): As per general method D, chlorinated (4-methoxyphenyl)(phenyl)methanol (9) (1 eq, 1.07 mmol, 0.25 g) was reacted with pyrrolidine (5 eq, 5.38 mmol, 0.38 g 0.45 mL) in dry ACN (50 mL) at reflux for 12 h. The product did not require any further purification. Yield: 83% (0.23 g) brown oil [30]. ^{1}H NMR (400 MHz, $CDCl_3$) δ 7.42 (d, *J* = 7.4 Hz, 2H, Ar-H), 7.34 (d, *J* = 8.7 Hz, 2H, Ar-H), 7.23 (d, *J* = 7.8 Hz, 2H, Ar-H), 7.13 – 7.15 (m, 1H, Ar-H), 6.78 (d, *J* = 8.6 Hz, 2H, Ar-H), 4.09 (s, 1H, CH-N-R), 3.73 (s, 3H, OCH_3), 2.42 – 2.36 (m, 4H, CH_2), 1.75 (dd, *J* = 6.4, 3.4 Hz, 4H, CH_2). ^{13}C NMR (101 MHz, $CDCl_3$) δ 158.33 (C-O), 144.61 (C), 136.63 (C), 128.43 (2xCH), 128.29 (2xCH), 127.32 (2xCH), 126.60 (CH), 113.65 (2xCH), 75.74 (CH-N-R), 55.15 ($2xCH_2$), 53.63 (OCH_3), 23.50 ($2xCH_2$). HRMS (EI): found 268.1692 $(M+H)^+$; $C_{18}H_{22}NO$ requires 268.1701. IR: ν_{max} (ATR) cm^{-1}: 3098, 3039, 2951, 1638, 1600, 1507, 1417, 1304, 1286, 1243, 1173, 1116, 1027, 966, 843, 724, 698.

1-(Bis(4-chlorophenyl)methyl)pyrrolidine (25e): As per general method D, chlorinated bis(4-chlorophenyl)methanol **(12f)** (1 eq, 2.7 mmol, 0.752 g) was reacted with pyrrolidine (5 eq, 13.5 mmol, 0.96 g 1.1 mL) in dry ACN (50 mL) at reflux for 12 h. The crude product was purified *via* flash chromatography (eluent: *n*-hexane/ethyl acetate 9:1). Yield: 89% (0.739 g) orange solid Mp: 69-70 °C [31]. ^{1}H NMR (400 MHz, $CDCl_3$) δ 1.74 – 1.76 (m, 4 H, CH_2), 2.32 - 2.40 (m, 4 H, CH_2), 4.10 (s, 1 H, CH-N-R), 7.21 (d, *J*=8.55 Hz, 4 H, Ar-H), 7.33 (d, *J*=7.93 Hz, 4 H, Ar-H). ^{13}C NMR (101 MHz, $CDCl_3$) δ

23.52 (2xCH$_2$), 53.45 (2xCH$_2$), 74.87 (CH-N-R), 128.63 (4xCH), 128.65 (4xCH), 132.59 (2xC-Cl), 142.40 (2xC). HRMS (EI): found 306.0816 $(M+H)^+$; $C_{17}H_{18}{}^{35}Cl_2N$ requires 306.0816 IR: ν_{max} (ATR) cm^{-1}: 2963, 2879, 2797, 1593, 1487, 1406, 1324, 1285, 1125, 1084, 1013, 804.

1-(Phenyl(*p*-tolyl)methyl)pyrrolidine (25f): As per general method D, chlorinated phenyl(*p*-tolyl)methanol **(12g)** (1 eq, 3.27 mmol, 0.71 g) was reacted with pyrrolidine (5 eq, 16.35 mmol, 1.16 g, 1.34 mL) in dry ACN (50 mL) at reflux for 12 h. The crude product was purified *via* flash chromatography (eluent: *n*-hexane/ethyl acetate 9:1). Yield: 57% (0.47 g) pale yellow solid Mp: 62-64 °C [29]. ^{1}H NMR (400 MHz, CDCl$_3$) δ 1.70 - 1.79 (m, 4 H, CH$_2$), 2.26 (s, 3 H, CH$_3$), 2.39 – 2.43 (m, 4 H, CH$_2$), 4.10 (s, 1 H, CH-N-R), 7.05 (d, *J*=7.93 Hz, 2 H, Ar-H), 7.10 - 7.15 (m, 1 H, Ar-H), 7.20 - 7.23 (m, 2 H, Ar-H), 7.32 (d, *J*=7.93 Hz, 2 H, Ar-H), 7.42 (d, *J*=7.32 Hz, 2 H, Ar-H). ^{13}C NMR (101 MHz, CDCl$_3$) δ 21.02 (CH$_3$), 23.53 (2xCH$_2$), 53.67 (2xCH$_2$), 76.21 (CH-N-R), 126.65 (CH), 127.35 (2xCH), 127.41 (2xCH), 128.32 (2xCH), 129.02 (2xCH), 136.27 (C-CH$_3$), 141.41 (C), 144.56 (C). HRMS (EI): found 252.1751 $(M+H)^+$; $C_{18}H_{22}N$ requires 252.1752. IR: ν_{max} (ATR) cm^{-1}: 3025 (C-H stretch of the aromatics), 2965, 2791, 1511, 1490, 1451, 1363, 1279, 1198, 1124, 895, 799, 740, 719, 695.

General method E: Benzhydryl-1*H*-imidazole derivatives

To a solution of the specific secondary alcohol (1 eq,) in acetonitrile (60 mL) CDI was added (1.3 eq,). The mixture was refluxed for 3 h, the acetonitrile was evaporated and the crude product was re-dissolved in DCM (30 mL) and washed with water (20 mL) and brine (10 mL). The product was dried over sodium sulphate, filtered and concentrated under reduced pressure. The crude product was purified *via* flash chromatography (*n*-hexane/ethyl acetate 1:1) over silica gel to afford the desired product.

1-Benzhydryl-1*H*-imidazole (20a): As per general method E, diphenylmethanol (**12a**) (1 eq, 2.93 mmol, 0.54 g) was reacted with CDI (1.3 eq, 3.8 mmol, 0.61 g) in dry ACN (50 mL) at reflux for 3 h under N$_2$. The crude product was then purified *via* flash chromatography (eluent: *n*-hexane/ethyl acetate 1:1). Yield: 20% (0.132 g) pale yellow solid Mp: 112-118 °C [32]. HPLC purity: 96%. ^{1}H NMR (400 MHz, CDCl$_3$) δ 5.31 (s, 1 H, CH-N-R), 7.06 (s, 1 H, CH-N), 7.10 (s, 1 H, Ar-H), 7.36 - 7.42 (m, 9 H, Ar-H), 7.51 (s, 1 H, CH-N), 8.23 (s, 1 H, CH-N). ^{13}C NMR (101 MHz, CDCl$_3$) δ 81.21 (CH-N-R),

117.16 (CH-N), 126.96 (2xCH), 127.13 (4xCH), 128.70 (CH-N), 128.80 (4xCH), 137.11 (2xC&CH-N). HRMS (EI): found 235.1229 $(M+H)^+$; $C_{16}H_{15}N_2$ requires 235.1235 IR: ν_{max} (ATR) cm^{-1}: 3132, 3063, 2972, 1749, 1494, 1454, 1395, 1318, 1294, 1253, 1175, 999, 922, 840, 766, 655.

1-((4-Nitrophenyl)(phenyl)methyl)-1*H*-imidazole (20b): As per general method E, (4-nitrophenyl)(phenyl)methanol **(12b)** (1 eq, 1.74 mmol, 0.40 g) was reacted with CDI (1.3 eq, 2.26 mmol, 0.36 g) in dry ACN (50 mL) at reflux for 3 h under N_2. The crude product was then purified *via* flash chromatography (eluent: *n*-hexane/ethyl acetate gradient 1:1 to 4:6). Yield: 53% (0.252 g) yellow oil [28]. ^1H NMR (400 MHz, $CDCl_3$) δ 7.10 (s, 1 H, CH-N-R), 7.13 (s, 1 H, CH-N), 7.38 - 7.45 (m, 5 H, Ar-H), 7.51 (s, 1 H, CH-N), 7.58 - 7.60 (m, 2 H, Ar-H), 8.23 (s, 1 H, CH-N), 8.27 (m, *J*=8.54 Hz, 2 H, Ar-H). ^{13}C NMR (101 MHz, $CDCl_3$) δ 80.31 (CH-N-R), 121.07 (CH-N), 123.90 (2xCH), 127.04 (CH), 127.16 (CH-N), 127.50 (2xCH), 128.95 (2xCH), 128.98 (2xCH), 137.86 (CH-N, C), 146.24 (C), 147.64 ($C-NO_2$). HRMS (EI): found 280.1074 $(M+H)^+$; $C_{16}H_{14}N_3O_2$ requires 280.1086 IR: ν_{max} (ATR) cm^{-1}: 3083, 2910, 2844, 1748, 1607, 1516, 1345, 1241, 1184, 1078, 988, 951, 936, 892, 842, 826, 743, 698.

1-(Bis(4-Chlorophenyl)methyl)-1*H*-imidazole (20f): As per general method E, bis(4-chlorophenyl)methanol (**12f**) (1 eq, 1.4 mmol, 0.35 g) was reacted with CDI (1.3 eq, 1.83 mmol, 0.29 g) in dry ACN (50 mL) at reflux for 3 h under N_2. The crude product was then purified *via* flash chromatography (eluent: *n*-hexane/ethyl acetate 1:1). Yield: 47% (0.2 g) white solid Mp: 154-158 °C [31]. HLPC purity: 98% ^1H NMR (400 MHz, $CDCl_3$) δ 6.96 (s, 1 H, CH-N-R), 7.08 (s, 1 H, N-CH), 7.28 (d, *J*=7.93 Hz, 4 H, Ar-H), 7.36 (d, *J*=8.54 Hz, 4 H, Ar-H), 7.45 (s, 1 H, N-CH), 8.17 (s, 1 H, N-CH). ^{13}C NMR (101 MHz, $CDCl_3$) δ 79.85 (CH-N-R), 117.09 (CH-N), 128.29 (4xCH), 128.49 (CH-N), 128.90 (4xCH), 131.00 (2xC-Cl), 134.42 (2xC), 137.36 (CH-N). HRMS (EI): found 303.0453 $(M+H)^+$; $C_{16}H_{13}{}^{35}Cl_2N_2$ requires 303.0456 IR: ν_{max} (ATR) cm^{-1}: 3125, 3017, 2790, 2862, 2682, 1747, 1541, 1490, 1260, 1088, 1054, 973.

General procedure F: Diarylmethanones 23a, 23b.

Eaton's reagent: Phosphorus pentoxide (P_2O_5) and methanesulfonic acid (CH_3SO_3H) (in a weight ratio P_2O_5:CH_3SO_3H 1:10) were mixed in a round bottomed flask and heated at 40 °C under nitrogen atmosphere until complete homogeneity. Carboxylic acid (1.5 eq,)

and aromatic derivative (1.0 eq,) were then added to Eaton's reagent. The mixture was heated at 60 °C under inert atmosphere for 3 h. After cooling to room temperature, the reaction medium was diluted with dichloromethane (60 mL) and carefully poured into a separatory funnel containing 50% aqueous solution of sodium bicarbonate (40 mL). The aqueous solution was extracted with dichloromethane, and the combined organic layers were dried over sodium sulphate, filtered and concentrated under reduced pressure.

(4-Methoxyphenyl)(3,4,5-trimethoxyphenyl)methanone (23a): Anisole (1 eq, 7.0 mmol, 0.75 g 0.75 mL) was reacted with 3,4,5-trimethoxybenzoic acid (1.5 eq, 10.5 mmol, 2.23 g) in Eaton's reagent (0.99 g P_2O_5 / 6.69 mL CH_3SO_3H). The mixture was stirred at 60 °C for 3 h under N_2. The product was diluted in DCM (60 mL) and poured in a separatory funnel containing $NaHCO_3$ 50% (40 mL) and extracted. The crude product was purified *via* flash chromatography (eluent: *n*-hexane/ethyl acetate 5:4). Yield: 60% (1.26 g) pink solid Mp: 76-81 °C [21]. ^{1}H NMR (400 MHz, $CDCl_3$) δ 3.88 (s, 6 H, OCH_3), 3.90 (s, 3 H, OCH_3), 3.94 (s, 3 H, OCH_3), 6.98 (d, *J*=8.53 Hz, 2 H, Ar-H), 7.02 (s, 2 H, Ar-H), 7.83 (d, *J*=9.03 Hz, 2 H, Ar-H). ^{13}C NMR (101 MHz, $CDCl_3$) δ 55.46 (OCH_3), 56.25 (2xOCH_3), 60.92 (OCH_3), 107.40 (2xCH), 113.50 (2xCH), 130.23 (C), 132.34 (2xCH), 133.30 (C), 141.54 (C-O), 152.78 (2xC-O), 163.08 (C-O), 194.61 (C=O). HRMS (EI): Found 325.1056 $(M+Na)^+$; $C_{17}H_{18}NaO_5$ requires 325.1052. IR: ν_{max} (ATR) cm^{-1}: 3401, 2951, 2837, 1641, 1600, 1510, 1494, 1445, 1332, 1305, 1233, 1250, 1111, 1018, 1025, 922, 840, 696, 761, 738, 696, 611.

(3,4-Dimethoxyphenyl)(3,4,5-trimethoxyphenyl)methanone (23b): 1,2-Dimethoxybenzene (1 eq, 7.24 mmol, 1 g) was reacted with 3,4,5-trimethoxybenzoic acid (1.5 eq, 10.86 mmol, 2.30 g) in Eaton's reagent (1.02 g P_2O_5 / 7.24 mL CH_3SO_3H). The mixture was stirred at 60 °C for 3 h under N_2. The product was diluted in DCM (60 mL) and poured in a separatory funnel containing $NaHCO_3$ 50% (40 mL) and extracted. The crude product was purified *via* flash chromatography (eluent: *n*-hexane/ethyl acetate 5:4). Yield: 57% (1.37 g) orange solid Mp: 128-131 °C [22]. ^{1}H NMR (400 MHz, $CDCl_3$) δ 3.89 (s, 6 H, OCH_3), 3.94 (s, 3 H, OCH_3), 3.95 (s, 3 H, OCH_3), 3.98 (s, 3 H, OCH_3), 6.92 (d, *J*=8.29 Hz, 1 H, CH), 7.04 (s, 2 H, Ar-H), 7.40 (d, *J*=2.07 Hz, 1 H, Ar-H), 7.47 (d, *J*=2.07 Hz, 1 H, Ar-H). ^{13}C NMR (101 MHz, $CDCl_3$) δ 56.07 (2xOCH_3), 56.30 (2xOCH_3), 60.96 (OCH_3), 107.44 (2xCH), 109.76 (CH), 112.25 (CH), 125.04 (CH),

130.30 (2xC), 148.95 (2xC-O), 152.80 (3xC-O), 194.62 (C=O). HRMS (EI): Found 333.1330 $(M+H)^+$; $C_{18}H_{21}O_6$ requires 333.1332. IR: ν_{max} (ATR) cm^{-1}: 2942, 1640, 1599, 1576, 1411, 1330, 1256, 1232, 1118, 1026, 996, 865, 835, 763, 613.

References

[1] Miyano M, Deason JR, Nakao A, et al. (Acyloxy)benzophenones and (acyloxy)-4-pyrones. A new class of inhibitors of human neutrophil elastase. *J Med Chem*. 1988;31(5):1052-61. doi:10.1021/jm00400a030.

[2] Silvestri R, Artico M, Martino G De, et al. Synthesis, Biological Evaluation, and Binding Mode of Novel 1-[2-(Diarylmethoxy)ethyl]-2-methyl-5-nitroimidazoles Targeted at the HIV-1 Reverse Transcriptase. *J Med Chem*. 2002;45:1567-1576.

[3] Olsson R, Hyldtoft L, Gustafsson M. Condensed compounds with activity at estrogen receptors. 2009:47-48.

[4] Mukhopadhyay A, Maka VK, Moorthy JN. Fluoride-Triggered Ring-Opening of Photochromic Diarylpyrans into Merocyanine Dyes: Naked-Eye Sensing in Subppm Levels. *J Org Chem*. 2016;81(17):7741-7750. doi:10.1021/acs.joc.6b01361.

[5] Meshram HM, Goud PR, Reddy BC, Kumar DA. Triton B–Mediated Efficient and Convenient Alkoxylation of Activated Aryl and Heteroaryl Halides. *Synth Commun*. 2010;40(14):2122-2129. doi:10.1080/00397910903219518.

[6] Davis RE, Gottbrath JA. Boron Hydrides. V. Methanolysis of Sodium Borohydride. *J Am Chem Soc*. 1962;84(Table I):895-898. doi:10.1021/ja00865a003.

[7] Lee CT, Lipshutz BH. Nonracemic diarylmethanols from CuH-catalyzed hydrosilylation of diaryl ketones. *Org Lett*. 2008;10(19):4187-4190. doi:10.1021/ol801590j.

[8] Li J, Zhang X, Shen H, et al. Boron Trifluoride.Diethyl Ether-Catalyzed Etherification of Alcohols: A Metal-Free Pathway to Diphenylmethyl Ethers. *Adv Synth Catal*. 2015;357(14-15):3115-3120. doi:10.1002/adsc.201500663.

[9] Ironside MD, Sugathapala PM, Robertson J, Darey MCP, Zhang J. Scale-Up Synthesis of the Dopamine Uptake Inhibitor GBR-12909. *Org Process Res Dev*. 2002;6(5):621-627.

[10] Brine GA, Boldt KG, Prakash D, et al. p-Hydroxymethadone: synthesis, crystal structure and CD properties. *J Chem Soc, Perkin Trans*. 1991;1(1):1809-1814.

[11] Kharul RK, Goswami A, Gite A, Godha AK, Jain M, Patel PR. Convenient Synthesis of Structurally Novel 1,3-Disubstituted Azetidine Derivatives. *Synth Commun*. 2008;38(11):1703-1717. doi:10.1080/00397910801982340.

[12] Yasuhara T, Manse Y, Morimoto T, et al. Acetoxybenzhydrols as highly active and stable analogues of 1'S-1'-acetoxychavicol, a potent antiallergic principal from Alpinia galanga. *Bioorganic Med Chem Lett*. 2009;19(11):2944-2946. doi:10.1016/j.bmcl.2009.04.065.

[13] Yan C, Zeng X, Zhang W, Luo M. Polymer-supported N-heterocyclic carbene-rhodium complex catalyst for the addition of arylboronic acids to aldehydes. *J Organomet Chem*. 2006;691(15):3391-3396. doi:10.1016/j.jorganchem.2006.02.021.

[14] Misawa T, Aoyama H, Furuyama T, et al. Structural development of benzhydrol-type 1'-acetoxychavicol acetate (ACA) analogs as human leukemia cell-growth inhibitors based on quantitative structure-activity relationship (QSAR) analysis. *Chem Pharm Bull (Tokyo)*. 2008;56:1490-1495. doi:10.1248/cpb.56.1490.

[15] Cunha RLOR, Omori ÁT, Castelani P, Toledo FT, Comasseto J V. One-pot

synthesis of aryl butyl tellurides from tellurium tetrachloride and activated aromatics through a solventless step. *J Organomet Chem*. 2004;689(22):3631-3636. doi:10.1016/j.jorganchem.2004.08.041.
[16] Doiron J, Soultan AH, Richard R, et al. Synthesis and structure-activity relationship of 1- and 2-substituted-1,2,3-triazole letrozole-based analogues as aromatase inhibitors. *Eur J Med Chem*. 2011;46(9):4010-4024. doi:10.1016/j.ejmech.2011.05.074.
[17] Zhang X, Xia A, Chen H, Liu Y. General and Mild Nickel-Catalyzed Cyanation of Aryl/Heteroaryl Chlorides with Zn(CN)2: Key Roles of DMAP. *Org Lett*. 2017;19(8):2118-2121. doi:10.1021/acs.orglett.7b00732.
[18] Wood PM, Woo LWL, Humphreys A, et al. A letrozole-based dual aromatase-sulphatase inhibitor with in vivo activity. *J Steroid Biochem Mol Biol*. 2005;94(1-3 SPEC. ISS.):123-130. doi:10.1016/j.jsbmb.2004.12.028.
[19] Bang JS, Kim YJ, Song J, et al. Small molecules that regulate zymosan phagocytosis of macrophage through deactivation of Rho GTPases. *Bioorganic Med Chem*. 2012;20(17):5262-5268. doi:10.1016/j.bmc.2012.06.043.
[20] Epple, R. Xie, Y Wang, X Russo, R Cow, C Azimioara M. Compounds and compositions as PPAR modulators. 2007.
[21] Cushman M. Synthesis and Evaluation of Analogues of (Z)-l-(4-Methoxyphenyl)-2-(3,4,5-trimethoxyphenyl)ethene as Potential Cytotoxic and Antimitotic Agents. *J Med Chem*. 1992;23(1):2293-2306.
[22] Ghinet A, Rigo B, Hénichart J-P, et al. Synthesis and biological evaluation of phenstatin metabolites. *Bioorg Med Chem*. 2011;19(20):6042-54. doi:10.1016/j.bmc.2011.08.047.
[23] Tanpure RP, Harkrider AR, Strecker TE, Hamel E, Trawick ML, Pinney KG. Application of the McMurry coupling reaction in the synthesis of tri- and tetra-arylethylene analogues as potential cancer chemotherapeutic agents. *Bioorganic Med Chem*. 2009;17(19):6993-7001. doi:10.1016/j.bmc.2009.08.011.
[24] L'Hermite N, Giraud A, Provot O, Peyrat JF, Alami M, Brion JD. Disproportionation reaction of diarylmethylisopropyl ethers: a versatile access to diarylmethanes from diarylcarbinols speeded up by the use of microwave irradiation. *Tetrahedron*. 2006;62(51):11994-12002. doi:10.1016/j.tet.2006.09.083.
[25] Gautret P, El-ghammarti S, Legrand A, Couturier D, Rigo B. On the silylation of diarylcarbinols. *Synth Commun*. 1996;26(4):707-713. doi:10.1080/00397919608086745.
[26] Maojiang, W Qinggang, J Chunhao Y. A novel and convenient method for the sinthesis of phenstatin. *Org Prep Proced Inc*. 2005;37(3):272-275. doi:10.1080/00304948909356417.
[27] Sakai N, Hori H, Yoshida Y, Konakahara T, Ogiwara Y. Copper(I)-catalyzed coupling reaction of aryl boronic acids with N,O-acetals and N,N-aminals under atmosphere leading to α-aryl glycine derivatives and diarylmethylamine derivatives. *Tetrahedron*. 2015;71(29):4722-4729. doi:10.1016/j.tet.2015.05.068.
[28] Le Gall E, Gosmini C, Troupel M. Aromatic organozinc reagents as nucleophiles in the α-arylation of piperidine and tetrahydropyran. *Tetrahedron Lett*. 2006;47(4):455-458. doi:10.1016/j.tetlet.2005.11.070.
[29] Chang S, Koh HJ, Lee B, Lee I. Kinetics and Mechanism of Reactions of

Benzhydryl Chlorides with Anilines. *J Org Chem*. 1995;(148):7760-7768.
[30] Le Gall E, Troupel M, Nédélec JY. One-step three-component coupling of aromatic organozinc reagents, secondary amines, and aromatic aldehydes into functionalized diarylmethylamines. *Tetrahedron*. 2006;62(42):9953-9965. doi:10.1016/j.tet.2006.08.008.
[31] Jones C, Winter M, Hirsch K, et al. Estrogen synthetase inhibitors. 2. Comparison of the in vitro aromatase inhibitory activity for a variety of nitrogen heterocycles substituted with diarylmethane or Diarylmethanol Groups. *J Med Chem*. 1990;33(Chart I):416-429. doi:10.1021/jm00163a065.
[32] Doiron J, Soultan AH, Richard R, et al. Synthesis and structure-activity relationship of 1- and 2-substituted-1,2,3-triazole letrozole-based analogues as aromatase inhibitors. *Eur J Med Chem*. 2011;46(9):4010-4024. doi:10.1016/j.ejmech.2011.05.074.
[33] Katritzky, A.R.Y., B.; Pleynet, D.P.M.; Wang, J. Novel heterocyclic analogs of trityl radicals: Synthesis and dimerization of diarylmethyl-1h-1,2,4-triazoles and diarylmethyl-2h-phenanthro[9,10-d]-1,2,3-triazoles. *Heterocycles* **2000** *52* 203-214.
[34] Dankwardt, J.W. Nickel-catalyzed cross-coupling of aryl grignard reagents with aromatic alkyl ethers: An efficient synthesis of unsymmetrical biaryls. *Angew Chem Int Ed Engl* **2004**, *43*, 2428-2432.
[35] Sengmany, S.L., E.; LeJean, C.; Troupel, M.; Nedelec, J. Straightforward three-component synthesis of diarylmethylpiperazines and 1,2-diarylethylpiperazines. *Tetrahedron* **2007** *63*, 3672-3681.
